# Tramtrack Is Genetically Upstream of Genes Controlling Tracheal Tube Size in *Drosophila*

**DOI:** 10.1371/journal.pone.0028985

**Published:** 2011-12-22

**Authors:** Barbara Rotstein, David Molnar, Boris Adryan, Marta Llimargas

**Affiliations:** 1 Institut de Biologia Molecular de Barcelona, CSIC, Barcelona, Spain; 2 Department of Genetics, Cambridge Systems Biology Centre, University of Cambridge, Cambridge, United Kingdom; Stockholm University, Sweden

## Abstract

The *Drosophila* transcription factor Tramtrack (Ttk) is involved in a wide range of developmental decisions, ranging from early embryonic patterning to differentiation processes in organogenesis. Given the wide spectrum of functions and pleiotropic effects that hinder a comprehensive characterisation, many of the tissue specific functions of this transcription factor are only poorly understood. We recently discovered multiple roles of Ttk in the development of the tracheal system on the morphogenetic level. Here, we sought to identify some of the underlying genetic components that are responsible for the tracheal phenotypes of Ttk mutants. We therefore profiled gene expression changes after Ttk loss- and gain-of-function in whole embryos and cell populations enriched for tracheal cells. The analysis of the transcriptomes revealed widespread changes in gene expression. Interestingly, one of the most prominent gene classes that showed significant opposing responses to loss- and gain-of-function was annotated with functions in chitin metabolism, along with additional genes that are linked to cellular responses, which are impaired in *ttk* mutants. The expression changes of these genes were validated by quantitative real-time PCR and further functional analysis of these candidate genes and other genes also expected to control tracheal tube size revealed at least a partial explanation of Ttk's role in tube size regulation. The computational analysis of our tissue-specific gene expression data highlighted the sensitivity of the approach and revealed an interesting set of novel putatively tracheal genes.

## Introduction

Transcription factors play critical roles in all aspects of development. They control the gene batteries that lead to cellular events such as proliferation, cell fate specification and differentiation, cell migration, cell morphological changes and apoptosis. Given this wide spectrum of functions, very often transcription factors exhibit a high degree of pleiotropy that hinders a comprehensive functional characterisation in a given tissue or developmental stage. Thus, identifying the plethora of target genes regulated by transcription factors is key to disentangling their multiple and complex activities. Nowadays, the combination of genome-wide approaches with cell type- or stage-specific isolation provides a powerful strategy to understand how the function of transcription factors is mediated.

Our previous work indicated that the transcription factor Ttk plays multiple relevant roles in the formation of the *Drosophila* tracheal system [1]. For a more mechanistic view, in the present work we aimed to map the downstream mediator targets of *ttk* during embryogenesis with a particular emphasis in the developing tracheal system. Ttk is a zinc-finger transcription factor widely used during the development of a number of different organ systems [2]–[15], with a pivotal role in many different morphogenetic events. In the trachea, we observed that Ttk is not only required for tracheal cell identity specification, but in addition it enables several morphogenetic changes, including the cell rearrangements of tube formation, and the proper setting of tube sizes [1]. Thus Ttk may govern a complex hierarchy of downstream mediators that execute cellular changes in tracheal development. We aimed to identify these downstream targets using microarray transcriptome profiling. This enabled us to find direct and indirect transcriptional targets, including those which are difficult to identify in traditional mutant screens due to pleiotropy and/or functional redundancy. To identify targets specific to the tracheal system, and to separate the specifically tracheal action of Ttk from action on other target systems, we combined whole-embryo expression profiles with transcription profiles of embryonic cell isolates, which were enriched for tracheal cells by fluorescence-activated cell sorting (FACS).

In this work we compared microarray expression profiles of wild-type embryos with different *ttk* mutant conditions, as well as expression profiles of tracheal cell isolates from both wild-type and mutant embryos. This analysis provided an *in vivo* census of genes whose transcription responds to Ttk loss-of-function or over-expression. Several of these genes have predicted Ttk binding sites in their regulatory regions, which make them candidates for direct regulation. To validate our experimental approach, we further analysed effects on the mechanism of tube size regulation, as we established the role of Ttk in this process before [1]. The size control of tracheal tubes is complex and involves several molecular mechanisms. It has been shown earlier that the transient assembly of a chitin filament inside the tracheal tubes, and epithelial septate junctions (SJs) play critical roles in the process (reviewed in [16]–[18]). Our current analysis of microarray data from whole embryos and isolated cells pointed to changes in the expression of chitin metabolism and SJ-related genes in *ttk* mutants, which were confirmed by quantitative real-time PCR (qPCR) profiling. In further tests we found that several of these targets are functionally required in tube size control. Thus we could confirm the involvement of Ttk in tube size regulation, and connect Ttk regulation to several downstream genes, which are involved in the control of tube size.

Our results provide entry points for the investigation of the targets of Ttk regulation in further processes. The results also show that our method of cell enrichment is a powerful tool in the search for transcription factor targets.

## Results

### Microarray analysis of ttk mis-expression mutants

**Figure 1:**
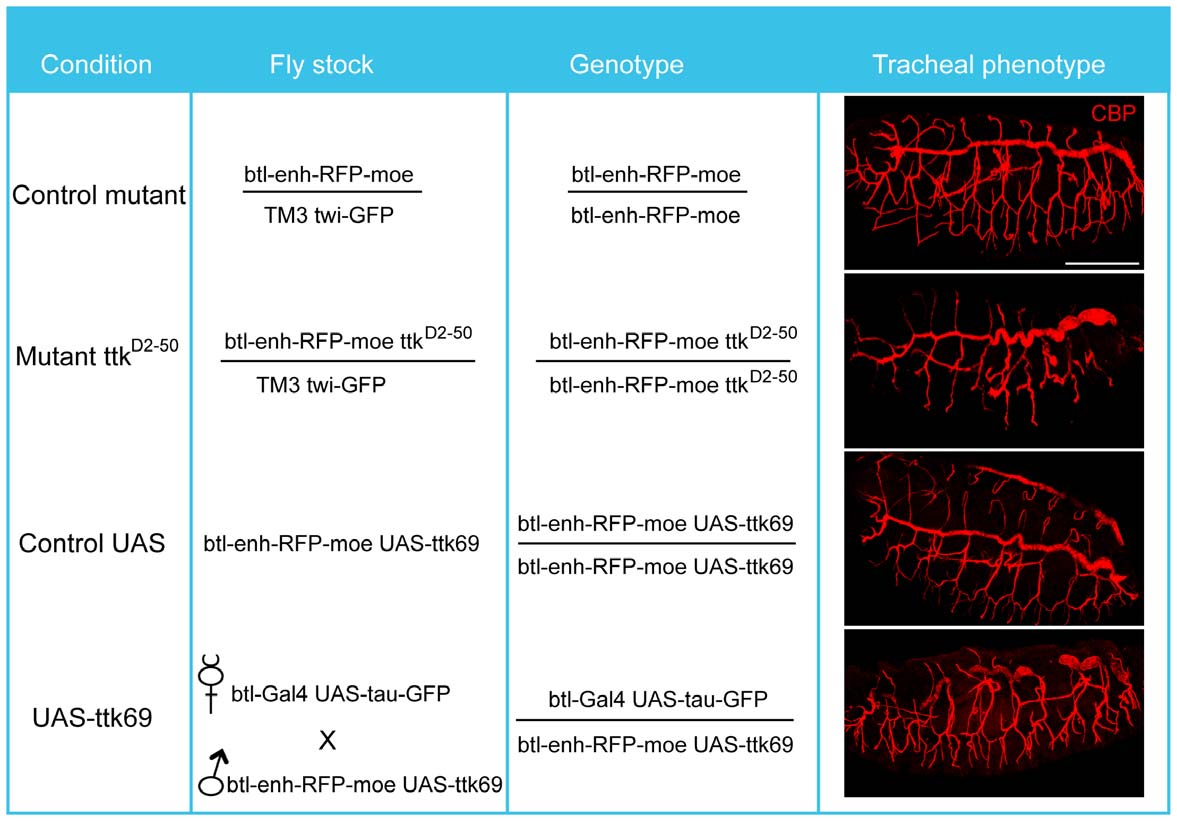
Experimental conditions, fly stocks and genotypes analysed in this study. The sample genotype and tracheal phenotype refer to the experimental condition that was analysed by microarray gene expression profiling. The tracheal pattern was visualised by the accumulation of chitin in the lumen using CBP. Immunostainings show projections of confocal sections of embryos of the indicated genotypes at embryonic stage 16 in lateral or dorsolateral views. In this and all figures anterior is to the left and dorsal is up. Scale bar 100 µm.

In order to identify direct or indirect Ttk target genes that could explain the diverse tracheal phenotypes seen in mutant embryos, we pursued RNA profiling experiments on embryos that are homozygous for the mutant *ttk* ^D2-50^ allele and control embryos at developmental stages 11 to 16. Furthermore we profiled embryos of the same age range that over-express *ttk* in the domain of the *btl*-GAL4 driver line, which includes the tracheal system and the midline, together with controls. All embryos, mutants and controls, expressed a membrane tethered RFP under the control of the *btl* enhancer from the Red Fluorescent Protein-moesin (btl-enh-RFP-moe) construct. This enabled a FACS strategy, which enabled us to obtain a cell population that was enriched for tracheal cells that were then subject to microarray gene expression analysis. The genotypes of these embryos and cells are detailed in Figure 1.

RNA was extracted in three biological replicates from each experimental group, with the exception of the experiments on the sorted cell populations in the over-expression group, where only two replicates were performed. The samples were hybridised to Affymetrix *Drosophila* Genome 2.0 Arrays. Following a rigorous protocol for quality control (see Text S1 and Figure S1), we decided to exclude one experiment from the embryo control for the mutant situation, one from embryo mutant, one from cell mutant. All experiments for the over-expression situation could be used. The microarray dataset is available from the Gene Expression Omnibus (http://www.ncbi.nlm.nih.gov/geo/) database under accession code GSE30239.

Two parameters primarily determine the number of candidate genes that can be obtained from a microarray experiment: the expected expression change (as indicated by the fold change in signal) and the reliability for this observation (implied in the p-value in the comparison between two different groups, under consideration of the noise structure within each of these groups). We therefore explored the parameter space from log_2_ fold change (logFC) 0.7 to 4 in increments of 0.1 between 0.7 and 1.5, and larger increments above 1.5, along with the p-value at 0.001, 0.005, 0.01 and 0.05 (Supplementary Table S1). For further analysis, we opted to define primary candidate genes as those with a logFC> = 1 (translating to an at least two-fold expression change) at a p-value< = 0.05. This parameter set identified 761 candidates in the mutant cell experiment and 676 genes in the mutant embryo experiment, with an overlap of 182 genes (24%). The identical parameter set was used for the over-expression experiments, identifying 322 genes in cells and 397 genes in embryos, with an overlap of 72 genes (22%).

The *btl* gene is expressed both in the tracheal system and in the ventral midline [19]. Manual inspection of the btl-enh-RFP-moe construct further confirmed detectable levels of expression in various other cell types (e.g. lateral ectoderm, endoderm, amnioserosa, others) (data not shown). The employed cell sorting strategy relied on the appropriate expression of marker fluorescence, and furthermore, cell sorting can be highly subjective to choosing appropriate gate settings (Figure S2). It was therefore unclear whether the sorted cell population was enriched for tracheal cells. We assessed the merit of the cell sorting strategy using a computational approach, making use of the high-throughput *in situ* hybridisation database at the BDGP [20]. We binned the signal intensity data from the control cell population in steps of approximately 0.5 (covering the dynamic range of 0 to16 of the microarray), and for each bin determined from the genes with BDGP information what fraction was known to be expressed in the tracheal system or midline, ubiquitously expressed, expressed elsewhere in the embryo, or not expressed (Figure S3). Surprisingly, we found a large number of genes that are supposedly not expressed in embryogenesis, accounting for almost half of the genes detected in the sorted cell population. While signal amplification issues in the pre-processing and detection of our microarray data are possible and may lead to considerable signals also for non-existent mRNA, it cannot be ruled out that a good proportion of the *in situ*-negative genes are indeed expressed. Of the group of genes known to be expressed during the relevant stages of embryogenesis, about half are expressed in the midline and/or trachea (and probably elsewhere), while the other half should not be present if our preparations were pure. Using two different normalization strategies to account for the differences in the RNA sources and microarray experiments, we found that the tracheal marker gene *trachealess* shows a two-fold enrichment in the sorted cell population in comparison to whole embryos, while e.g. a marker for mesoderm (*twist*) is reduced to about one third (Table S2 and Table S3). These results also hold for the over-expression experiments. In summary, while it was not possible to isolate a pure population of tracheal cells, we were confident that these experiments still provide valuable information for candidate gene prioritisation.

### The *in vivo* transcriptome confirms in vitro results and is backed by known biology

Recently, Reddy et al. published an expression profile of the S2 *Drosophila* cell line after *ttk* knockdown by RNA interference [21]. Using our criteria for candidate genes, their screen yielded 1,380 candidate genes. Although the absolute overlap between their candidate gene list and our lists is small, the degree of overlap between these experiments is statistically significant (Table 1) indicating that similar trends were detected in both datasets (see Discussion for a more detailed comparison).

**Table 1:** Overlap in candidate gene lists from different transcriptomes.

		S2 mutant		UAS, cells		UAS, embryos		mutant, cells		mutant, embryos	
		down	up	down	up	down	up	down	up	down	up
	FlyMine genes	620	760	96	246	73	326	254	515	220	422
**S2 cells**	**down**	-	1	1	22 **(9.7×10^−4^)**	3	7	21 **(3.3×10^−3^)**	14	16 **(2.5×10^−2^)**	18
	**up**	-	-	5	12	2	9	10	79 **(<10^−16^)**	13	35 **(8.4×10^−3^)**
**UAS, cells**	**down**	-	-	-	0 **(<10^−16^)**	21 **(<10^−16^)**	2	7 **(4.5×10^−4^)**	13 **(1.6×10^−5^)**	4 **(2.0×10^−2^)**	7 **(1.0×10^−2^)**
	**up**	-	-	-	-	0	51 **(<10^−16^)**	17 **(9.4×10^−7^)**	30 **(4.7×10^−9^)**	14 **(1.2×10^−5^)**	21 **(9.6×10^−6^)**
**UAS, embryos**	**down**	-	-	-	-	-	0	4 **(1.2×10^−2^)**	10 **(9.3×10^−5^)**	1	2
	**up**	-	-	-	-	-	-	18 **(1.3×10^−5^)**	23 **(1.6×10^−3^)**	10 **(1.8×10^−2^)**	7
**mutant, cells**	**down**	-	-	-	-	-	-	-	0	35 **(<10^−16^)**	0
	**up**	-	-	-	-	-	-	-	-	4	143 **(<10^−16^)**
**mutant, embryos**	**down**	-	-	-	-	-	-	-	-	-	0
	**up**	-	-	-	-	-	-	-	-	-	-

The table shows the number of candidate genes observed under each experimental condition. The degree of overlap is shown in absolute numbers, overlaps with statistically significant p-values are highlighted in bold. Note that the FlyMine list comparison tool considers splits and merges in gene models, which explains the slight offset between Table 1 and Table S1.

We were also interested in genome-wide trends in gene expression that are probably better reflected on a functional rather than per-gene level. For each gene list in Table 1 we calculated Gene Ontology (GO) over-representation statistics. The variety of functional classes that show over-representation in these gene lists was diverse, with no clearly defined general function for Ttk action (Supplementary Table S4). While the analysis of the S2 data confirmed previously detected GO terms involved in developmental processes [21], our mutant data revealed not immediately conclusive terms such as up-regulation of genes involved in ‘oocyte axis specification’ (GO:0007309) or ‘G-protein coupled receptor protein signaling pathway’ (GO:0007186), while no GO over-representation could be identified for genes down-regulated in the mutant. Equally remarkable, there was very little overlap in GO terms between the different experiments, ‘cell fate commitment’ (GO:0045165) being one of them that was shared between the S2 cell-based experiments and the genes down-regulated in embryos after *ttk* over-expression. Interestingly, as our work had previously implied a connection between Ttk function and cuticle formation [1], we found a range of GO terms involved in the ‘chitin metabolic process’ being over-represented amongst the genes up-regulated after Ttk over-expression in embryos.

### Identification of putative Ttk target genes involved in chitin metabolism

**Figure 2:**
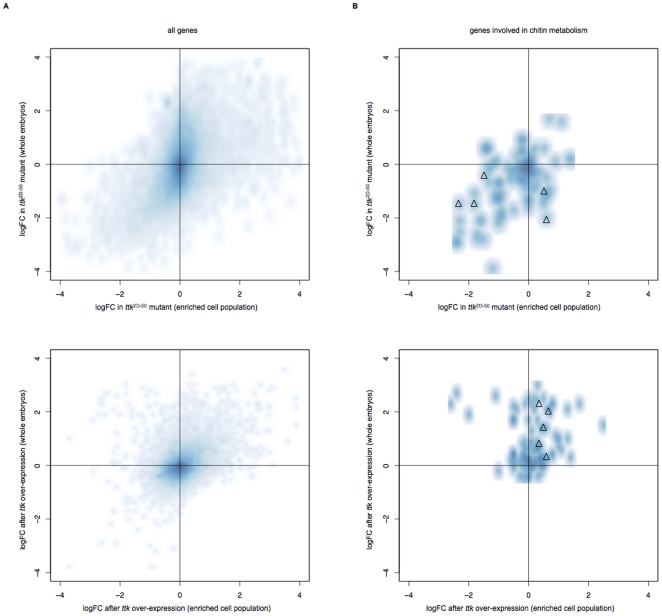
Gene expression changes in *ttk* mis-expression experiments. (A) Upper panel: *ttk* ^D2-50^ mutant; lower panel: *ttk* over-expression. The density plots show the expression changes of all genes on the microarray, in direct comparison of the enriched cell population (along the x-axis) to whole embryos (along the y-axis). Most genes do not change in expression (darker shade of blue around 0;0). If genes respond to *ttk* mutation, the change follows the same trend in embryos and sorted cells (roughly describing a diagonal), however there are outliers that behave counter-intuitively. Similar results can be observed after ttk over-expression. (B) The same plot as in (A), but restricted to genes annotated with GO term “chitin metabolism”. In contrast to all genes, the expression changes are much more pronounced and suggest a systematic response to *ttk* mis-expression. Triangles indicate genes further investigated in the course of this study.

In order to prioritize target genes for further validation, we visually inspected scatter plots in which we plotted the logFC in whole embryos versus our enriched (tracheal) cell population for both mutant and over-expression experiments (Figure 2A). As can be seen, the majority of genes cluster around the origin of the plots (i.e. coordinate 0;0), indicating they are not affected by the mis-expression condition. Importantly, the majority of genes with clear expression changes lie on the diagonal of the plot, indicating that direction and amplitude are the same for embryos and sorted cells, though this trend is less pronounced in the over-expression situation. We restricted the display to genes that were involved in GO biological processes with at least 50 annotated genes, with a closer look at those terms previously flagged as significantly enriched, one GO term at a time (File S1). As already suggested in the gene expression analysis of cell isolates, this analysis also showed a footprint of nervous system development. A large variety of GO terms implicated in central and peripheral nervous system development displayed converse expression behaviour in mutant and over-expression conditions. However, as we were particularly interested in genes involved in the tracheal system, genes annotated for functions in chitin metabolism (known to be involved in tracheal tube size control) came to our immediate attention, because in many cases they show inverse regulation in the mutant and over-expression situation, and consistently showed significant absolute logFC values (Figure 2B).

We therefore picked a few of the genes highlighted in this process for further validation of our microarray results.

### Chitin metabolic genes show tube length phenotypes consistent with *ttk* mis-expression

We previously described that *ttk* plays a role in controlling tracheal tube size [1]. Thus, we selected genes differentially expressed in our *ttk* experimental assays that participate in chitin metabolism and SJ organization (as identified by annotation with GO:0005918, ‘septate junction’) as they are involved in tube size control (review in [16]–[18]).

To validate the expression changes of these putative *ttk* targets obtained in the microarray experiment we performed quantitative real-time PCR analysis. The results confirmed the directional change in the levels of expression of all genes we tested (Table 2). Nine genes assigned to the GO term ‘chitin metabolism’ were analyzed. We confirmed the expected gain or loss of RNA abundance in eight candidate genes in mutant embryo conditions. Furthermore, we validated the directional change of six of them in *ttk* over-expression experiments. Remarkably, our qPCR experiments confirmed minor transcriptional changes (absolute logFC<1) for several candidate *ttk* target genes. Three genes annotated with ‘septate junction’ were also analysed by qPCR. Also here, the qPCR results confirmed the transcriptional changes and the direction in the change that were observed in the microarray analysis. This established that our microarray data can be used as a reliable platform for further downstream analysis.

**Table 2:** Validation of microarray candidates by quantitative PCR.

	*ttk* ^D2-50^ embryos				*ttk* over-expression embryos			
Gene name	Microarray log FC	p-value	qPCR FC	p-value	Microarray log FC	p-value	qPCR FC	p-value
*Cht2*	**−1.47 ↓**	1.65E^−01^	**−5.5 ↓**	**<0.05**	0.22	**2.53E^−02^**	**2.4 ↑**	**<0.05**
*Cht5*	**−1.27 ↓**	**2.64E^−02^**	**−10.0 ↓**	**<0.05**	**0.85 ↑**	**1.60E^−04^**	**2.1 ↑**	**<0.05**
*Cda4*	**−2.03 ↓**	1.59E^−01^	**−5.3 ↓**	**<0.05**	−0.02	9.36E^−01^	**−1.5 ↓**	**<0.05**
*Cpr78Cb*	**−2.08 ↓**	**3.20E^−02^**	**−15.0 ↓**	**<0.05**	0.65	3.37E^−01^	ND	
*Idgf3*	**−1.96 ↓**	**2.53E^−02^**	**−2.5 ↓**	**<0.05**	0.29	5.91E^−01^	ND	
*Cht9*	−0.61	2.58E^−01^	ND		**2.41 ↑**	**0.00E^+00^**	**4.7 ↑**	**<0.05**
*CG7715*	**−1.46 ↓**	2.68E^−01^	**−15.6 ↓**	**<0.05**	**1.42 ↑**	**4.44E^−02^**	**2.9 ↑**	**<0.05**
*CG8460*	**1.67 ↑**	**1.79E^−02^**	**5.4 ↑**	**<0.05**	0.18	8.11^−02^	ND	
*Idgf1*	−0.70	2.83E^−01^	**−9.00 ↓**	**<0.05**	**2.00 ↑**	**2.83E^−02^**	**10.0 ↑**	**<0.05**
*dlg1*	**1.57 ↑**	**9.15E^−03^**	**1.61↑**	**<0.05**	−0.015	1.03E^−01^	ND	
*Scrib*	0.50	5.00E^−01^	ND		**1.57 ↑**	**3.35E^−03^**	**1.3 ↑**	**<0.05**
*vari*	**−1.35 ↓**	**4.91E^−02^**	**−6.6 ↓**	**<0.05**	−0.015	2.32E^−01^	ND	
*Hdc*	**2.55 ↑**	**3.78E^−02^**	**10.0 ↑**	**<0.05**	0.25	1.00E^−01^	ND	
*sc*	**1.86 ↑**	**9.75E^−02^**	**1.6 ↑**	**<0.05**	**−1.29 ↓**	**4.36E^−02^**	**−2.8 ↓**	**<0.05**
*ac*	**1.21 ↑**	3.12E^−01^	**−2.6 ↓**	**<0.05**	**−1.80 ↓**	**4.88E^−02^**	**−2.0 ↓**	**<0.05**
*H*	**2.27 ↑**	**4.83E^−02^**	**1.5 ↑**	**<0.05**	−0.15	2.34E^−01^	ND	

Values obtained in the microarray and qPCR validation experiments performed on selected genes identified by microarray analysis in ttk mutant and ttk overexpression in whole embryo conditions, at developmental stages 11–16. qPCR analysis was performed using aliquots of cDNA pools prepared for microarray analysis. Note the correlation in the levels and direction of change between the two sets of experiments.

**Figure 3:**
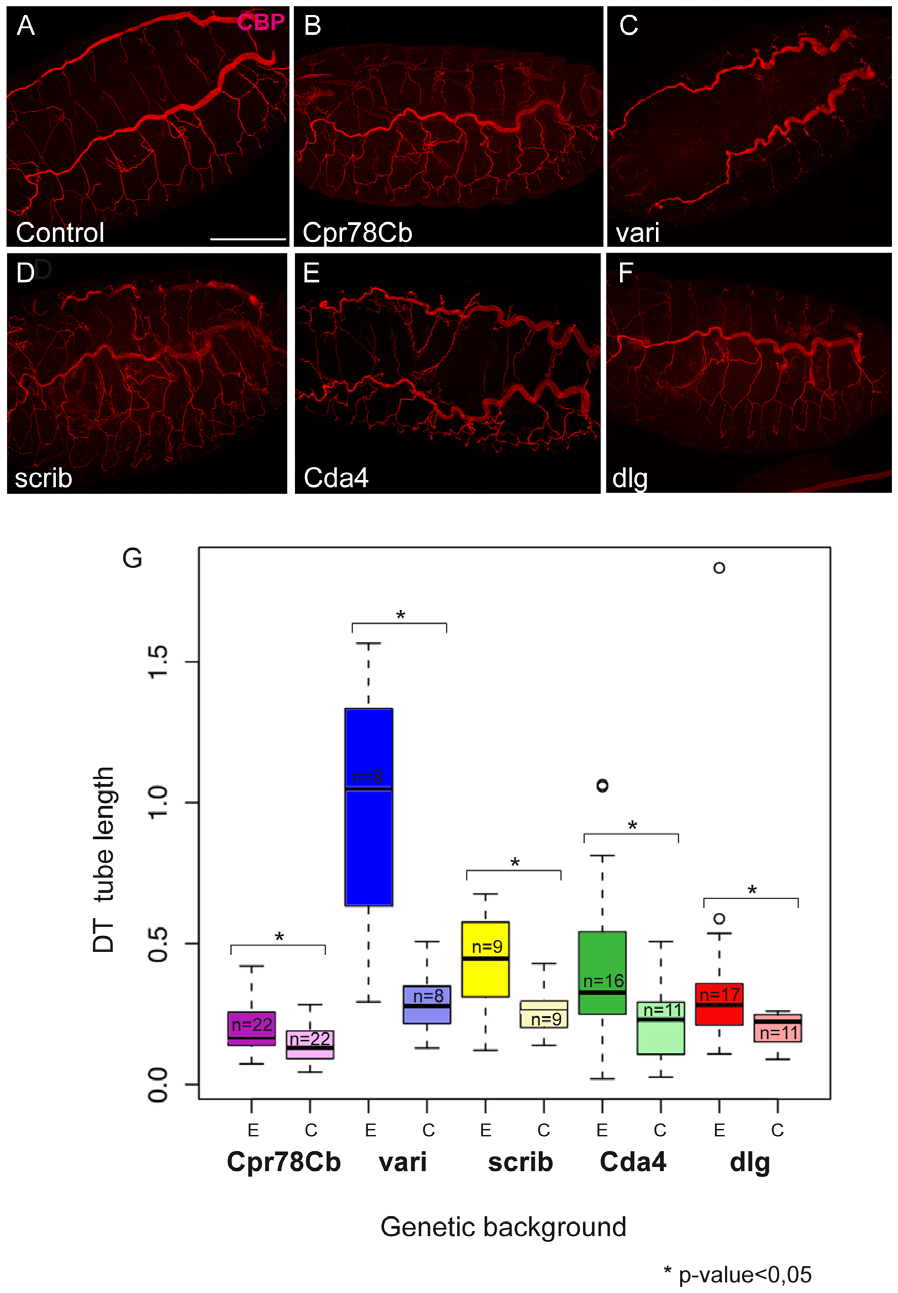
Analysis of the effect of putative *ttk* targets on tube length. (A–F) Projections of confocal sections showing dorso-lateral views of embryos at embryonic stage 16, labelled to visualise the chitin filament with CBP. Anterior is to the left and dorsal is up in all panels. The RNAi lines or loss-of-function alleles used were the following: (B) Cpr78Cb: RNAi 107767; (C) vari^3953^; (D) Scrib RNAi; (E) Cda4: RNAi 36106; (F) dlg1: RNAi 1076. RNAi lines were driven with *btl*-GAL4 UAS-tau-GFP. Note the enlarged DTs in embryos from B to F as compared to A (control). (G) Histogram showing tracheal tube lengths. For each gene we compared the experimental situation that we called E to its internal control that we called C. E corresponds to the loss-of-function situation, either RNAi expressed with *btl*-GAL4 UAS-tau-GFP, or the homozygote mutant in the case of loss-of-function alleles. C corresponds to heterozygote embryos in the case of loss-of-function alleles or to the RNAi line alone. n indicates the number of embryos that were measured. The differences in length between the experimental and control conditions are significant at p<0.05. Scale bar 100 µm.

To understand whether these *ttk* targets can mediate, at least in part, the function of *ttk* in tube size regulation we performed a functional analysis. We either expressed UAS-inducible RNAi in the tracheal system using the *btl*-GAL4 driver or used available loss-of-function alleles to down-regulate the activity of eleven selected *ttk* targets. We scored the tube size phenotype by staining embryos with chitin binding probe (CBP), a marker that allows visualizing the luminal chitin filament, which can be used as a read-out for tube length. We obtained normalized measures of dorsal trunk length and compared each mutant condition to an internal control. We found that five out of the eleven genes tested exhibited a tube size phenotype. Interestingly, the mutant situations achieved by loss-of-function alleles (the cases of *vari* and *dlg1*) proved positive. 4 out of 10 candidates tested by RNAi expression produced a tube size effect (*Cda4*, *Cpr78Cb*, *Scrib*, *dlg*1) indicating expected [22] or non-previously described requirements of these genes in controlling the growth of the tracheal tubes (Figure 3). The tracheal expression of RNAi lines of another 6 candidates did not result in tube size defects, indicating that either the gene is not involved in tube size regulation or that the strength of the interfering lines are not sufficient to generate a visible effect.

Together these results identify several direct or indirect target genes for *ttk* involved in chitin metabolism or SJ organization that mediate Ttk activity during tube size regulation.

### Tramtrack feeds into various developmental programmes in the tracheal system

Our previous work further found an involvement of Ttk in the fusion cell fate and a potential interaction with the Notch signaling cascade [1]. We examined genes with described functions in the fusion process (branch fusion, open tracheal system; GO:0035147) and the Dysfusion target gene CG15252 [23] for transcriptional changes in our microarray dataset (Supplementary Table S6). A key transcription factor gene (*escargot*) and *headcase* and CG15252 showed a significant transcriptional response in the enriched cell population after *ttk* over-expression (logFCs of −1.2, 1.1 and 2.3, respectively; all at p<0.02 or less). We observed changes in the same direction in mutant embryos (logFC>2.5 for *hdc*) that we could positively validate by qPCR (Table 2).

In a computational analysis of the genetic interaction network in *Drosophila*, we were searching for interacting gene pairs where both partners show a transcriptional response in either our or the S2 microarray datasets (data not shown). Interestingly, we found a local cluster of genes that are either targets of the Notch downstream effector Su(H) (*HLHmdelta*, *scute*, *achaete*, *E(spl)*, *HLHm4*) or known interactors of the transcription complex (Hairless), or factors downstream of H function (e.g. *Bearded*). Importantly, many of these genes show significantly decreased expression in the over-expression situation, and increased expression in the mutant situation, which could be confirmed by qPCR (Table 2). While the role and mechanism of Ttk in the regulation of these Notch target genes is not clear, this may provide a first explanation of our previous observations (see Discussion).

### Stage-specific effects of Ttk regulation

Our microarray analysis and the validations of several candidates by qPCR generated results not always easy to interpret. For instance, we previoulsy described a regulation of *branchless* (*bnl*) and *mummy* (*mmy*) by *in situ* hybridisation [1] that we could not confirm in our microarray experimental data. In addition, many genes showed unidirectional transcriptional responses (either up or down) to Ttk mis-expression conditions (either mutant or over-expression). For instance, the expression of *cda4* goes down in both mutant and over-expression conditions. Furthermore, we also found some inconsistencies in the mode of transcriptional regulation (either up or down) between the microarray data and the qPCR experiments, as in the case of *ac* (see Table 2). While over-compensation effects of the genetic interaction network could possibly explain these counterintuitive behaviors, another possibility is that Ttk itself exhibits different time-specific roles (e.g. first activating, then repressing), which cannot be resolved in our samples. We therefore selected three genes (*bnl*, *cda4* and *ac*) and probed their expression profile by stage-resolved qPCR. We performed qPCR experiments on Ttk mutant embryos and compared to their controls at stages 11 to 13 and at stages 14 to 16 (Table S7). We observed a downregulation of *cda4* and *bnl* at the different stages tested, in agreement with our previous observations. Interestingly, we found that *ac* is regulated differently by Ttk at early or late embryonic stages, indicating that Ttk can exert different stage-specific modes of action.

### Tramtrack might directly regulate the expression of tracheal target genes

**Figure 4:**
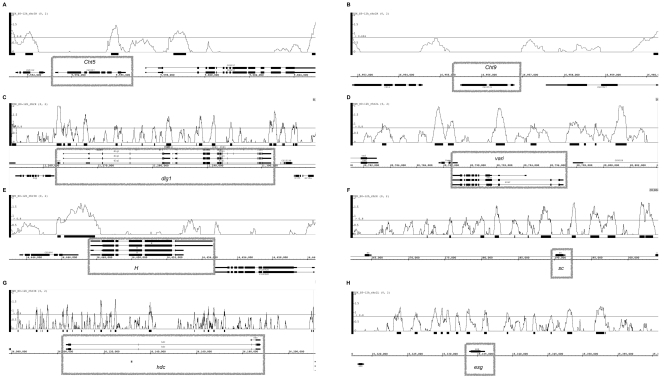
Chromatin immunoprecipitation (ChIP) enrichment for Ttk as determined by modENCODE. Panels (A–H) show ChIP profiles along the chromosomal axis for candidate genes (boxed) obtained by gene expression profiling. We assume measurable enrichment at lg2(0.8), or 1.5-fold enrichment over background levels (indicated by the gray horizontal line. Genes plotted above the coordinate line are encoded on the plus-strand, and those below the coordinate line are on the minus-strand. It is noteworthy that Ttk enrichment does not necessarily occur near the 5′-end of these genes, but can also occur in intronic regions as frequently seen also for transcription factors.

The broad range of biological functions could be either direct or indirect consequences of Ttk's role as a transcription factor. An initial step towards asserting this question is a look at genome-wide binding profiles of Ttk, and to see whether our candidate genes are positioned in the vicinity of Ttk binding sites. The modENCODE project [24] has recently published such data for *Drosophila* embryos between stages 1–15, thereby capturing a significant proportion of tracheal development. While many caveats apply in the interpretation from whole-embryo data at this broad time frame, the manual inspection of our candidate genes suggests that the majority of them possess significant Ttk binding (Figure 4). This direct input from Ttk concerns genes involved in chitin metabolism (Figure 4A), septate junction formation (Figure 4C and 4D), Notch signaling (Figure 4E and 4F) and fusion cell fate (Figure 4G and 4H). It is noteworthy that Ttk binding is not a default, and that some of our candidate genes do not show significant binding during embryonic development, e.g. *Cht9* (Figure 4B) or *m4* (data not shown). In respect to the apparent importance of Ttk in the regulation of genes involved in chitin metabolic processes, we performed a global analysis of Ttk binding using the modENCODE data (Figure S4). We found that 40 of 77 chitin metabolic genes showed Ttk ChIP enrichment> = lg2(1). Within this group of genes with Ttk enrichment, there is a higher proportion of genes with absolute logFC> = 1 as transcriptional response in at least one of the experimental conditions probed with our microarrays (70% versus 51%), although this difference is not statistically significant (χ^2^ test, p = 0.11). The observation that about half of the genes without Ttk enrichment show a transcriptional response suggests that Ttk may indirectly moderate their expression via yet unknown additional regulators. However, the result of many target genes having Ttk enrichment also suggests that Ttk mediates its function not only as a coordinator of other transcriptional regulators, but feeds in directly to the target genes immediately involved in the cellular response. It remains to be shown whether this role requires additional cofactors, or is primarily dependent on Ttk binding.

## Discussion

The study of transcriptional regulators involved in *Drosophila* tracheal development has thus far primarily focused on their characterisation on the morphological level. While tracheal phenotypes were reported for many transcription factors and their developmental role was assessed on the cellular level (see [25] for review), we still lack a deeper understanding why certain factors yield particular phenotypes, mostly for the lack of putative target genes. In the present paper, we used transcriptional profiling to show that mis-regulation of the transcription factor Ttk feeds into a variety of previously known tracheal regulatory processes. While it is not possible to infer whether this input is direct with Ttk activating or repressing the respective target genes, or indirect due to effects mediated by additional regulators, this analysis provides a first step to understand the molecular basis of the morphological abnormalities that can be seen in *ttk* mutant embryos [1]. We previously showed that Ttk has immediate impact on several tracheal morphogenetic events such as tube size regulation, cell rearrangements or intracellular lumen formation. Here, we provide first evidence that the effect on tube size is to a large degree dependent on genes we find transcriptionally downstream of Ttk (see further below).

### Microarray data quality, experimental limitations and interpretation

We used microarray gene expression profiling to identify putative targets of Ttk-dependent gene regulation. Data reproducibility in microarray experiments is typically a function of the number of replicates that are afforded. Given the number of experimental conditions we wanted to study, we compromised on a rather small number of replicates (three per condition attempted, in some cases only two were successful). Therefore, taking a rather conservative approach in the data analysis, we concentrated on genes with significant changes in gene expression as response to Ttk mis-regulation. Quite reassuringly, we found that the expression changes of most genes better our p-value cutoff (p<0.05) could be confirmed in our qPCR validation experiments. This implies that there may be many more transcriptional changes that we were not able to detect due to stringent cutoffs. There were a few counterintuitive results, which however were not statistically significant. For example, our previous *in situ* hybridization experiments showed *bnl* to go down in *ttk* mutants [1] while the microarray results could not reproduce this regulation. However, the variation for this gene between individual microarray replicates is large (resulting in a p-value≫0.5), meaning that the measurement for this gene is unreliable. Further analysis of *bnl* by stage-dependent qPCR validated the transcriptional change observed by *in situ* hybridisation. Interestingly, a stage-resolved qPCR analysis also helped to interpret contradictory results between microarray analysis and qPCR experiments, as it indicated stage-specific modes of Ttk transcriptional control.

We sought to obtain tissue-specific gene expression profiles to discern Ttk-responses that are general and those that are probably specific to the tracheal system. We therefore generated expression profiles from FACS-sorted cell populations that expressed a fluorescent reporter under the control of the *btl* enhancer (btl-enh-RFP-moe). The *btl* enhancer is widely used in the community for studies of the tracheal system, despite its well-known expression in the ventral midline [26] as well as other parts of the embryo. Importantly, we find similarly strong enrichment for RNAs for primarily tracheal genes (e.g. *trachealess*) and the midline marker *single-minded*, but also a strong transcriptional footprint for the amnioserosa and other tissues, for which thus far there was only anecdotal evidence for the activity of the *btl* enhancer. We therefore conclude that non-visual analyses of the tracheal system should therefore avoid the *btl* enhancer if tracheal specificity is desired. Interestingly, we also found genes expressed in the purified cell population that according to the BDGP gene expression database should not be expressed in the embryo. By comparison to temporal expression profiles from modENCODE (data not shown), we inferred that many of these are false-negative results of the *in situ* hybridisation experiments. Despite technical limitations, we would like to point out that our gene expression catalogue of btl-enh-RFP-moe positive cells might represent a valuable entry point for further analysis. While the interpretation of the numerical data may not be as straightforward as the *in situ* hybridisation results obtained in high-throughput by the BDGP, the expression values of the purified cell population allow an initial judgment if a gene might be expressed in the trachea or not.

Although not pure, our analysis indicates that the expression profiles of the sorted cell population are at least enriched for tracheal targets and therefore very useful. Using our parameter combination to identify “significantly changed” transcripts after Ttk mis-expression, we determined an overlap of about 25% between the whole embryo versus the purified cell population datasets. Can this degree of overlap be expected? While one may naively expect a higher degree of overlap as the molecular mechanisms of transcription factor function should be conserved, we argue that a versatile transcription factor such as Ttk likely depends on a wide range of different co-factors to exhibit its different functions. We therefore argue that the observed degree of overlap in the transcriptional response reflects the different transcriptional background (of active genes) of whole embryos and sorted cells. In addition, the assayed cell population accounts for an estimated 5–10% of the whole embryonic cell mass, and transcriptional changes in these cells may therefore be too dilute to be picked up in the whole-embryo expression profile. Furthermore, variations due to technical reasons may also account for, although minor, differences in the transcriptome profiles. We observe cases were the transcriptional response is opposite in whole embryos and cells. Again, this is probably due to different co-regulators that dictate the directionality of an expression change. Although Ttk is widely believed to act as transcriptional repressor [3], [5], [9], [14], the analysis of genome-wide binding data has suggested alternative roles for many transcription factors, and a recent theoretical study implies that most transcription factors may act in different directions [27].

Using our criteria to select candidate genes, the screen of Reddy et al. on the S2 *Drosophila* cell line after *ttk* knockdown by RNA interference [21] yielded 1,380 candidate genes as compared to 600–800 genes in our *ttk* ^2D-50^ mutant conditions. The larger number of genes is at least in part owed to the fact that their dataset comprises many more replicates and, therefore, allows for a greater number of genes to fulfill the p-value< = 0.05 criterion. Quite reassuringly, however, the degree of overlap between our and their experiments is statistically significant although the absolute overlap between their candidate gene list and our lists is small (Table 1). For example, the 620 genes down-regulated in S2 cells overlap with 22 of the 246 genes up-regulated in cells after *ttk* over-expression (p = 9.7×10^−4^) and 21 of 254 genes down-regulated in our cell population experiments in *ttk* ^2D-50^ mutants (p = 3.3×10^−3^). The majority of experiments that are expected to be conversely correlated (e.g. up in *ttk* ^2D-50^ mutant, down after *ttk* over-expression, or vice versa) show significant overlap. However, it should also be noted that in a few cases results emerged that were not intuitive, e.g. the overlap of 30 genes up-regulated in both mutant and over-expressing cells (p = 4.7×10^−9^). It may be assumed that, while the majority of direct or indirect Ttk target genes respond appropriately to the experimental situation, the over-expression in particular provokes unexpected responses: the 246 up-regulated genes in cells after *ttk* over-expression overlap significantly with almost all other experimental conditions. We argue that the mutant situation is likely to represent the biologically better interpretable response, as the over-expression situation may see many more ectopic downstream effects.

### The microarray analysis validate previous functional analysis

Ttk plays multiple roles during *Drosophila* development. In the case of tracheal development we previously observed requirements for Ttk at several steps. Some of these requirements are particularly interesting from the morphogenetic point of view because they involve cellular responses downstream of cell fate specification [1]. Thus, we expected Ttk to control several target genes that impinge directly on cell behaviour, affecting, for instance, the cytoskeletal network, intracellular trafficking or cell junctions. In line with our previous assumptions, we find several Ttk-regulated genes that belong to the GO categories of “vesicle-mediated transport”, “cytoskeleton”, or “plasma membrane” (Table S6). Very interestingly, we find many genes involved in the Microtubule cytoskeleton, which has been involved in several tracheal events also affected by Ttk activity, like branch fusion, terminal branch formation, or luminal chitin deposition [28]–[32]. Immunostainings with several microtubule markers further indicate that the microtubule network is not properly organized in Ttk mutants (ML, unpublished data). Future work will help to determine how Ttk controls microtubule activity and organization, and how this contributes to Ttk phenotype. It is noteworthy that the broadest range of GO terms shared between genes that come up after *ttk* knockdown in cells and in the *ttk* mutant are involved in ‘female gamete generation’ (GO:0007292). Ttk has previously been implicated in ovarian follicle development [12], [33], and it appears that this function is probably linked to a gene set that may also exhibit broader functionality in the embryonic or cell culture context. This may also be true for an extensive list of genes involved in embryonic axis determination, one of the first functions described for Ttk [2].

Remarkably, our microarray results confirm previous observations and provide new data for the different Ttk tracheal requirements. For instance, we described that the transcription factor Esg, which plays a pivotal role in fusion cell identity specification [34], [35] is lost when Ttk is over-expressed, but still present in Ttk loss-of-function conditions [1]. The microarray data confirm this regulation, and in addition identifies other genes already shown to directly or indirectly modulate fusion fate as Ttk targets, like *hdc* [36], CG15252 [28], or *pnt* [37]. Similarly, we find that *polychaetoid* (*pyd*), which we identified as a Ttk target in *in situ* hybridisation analysis [1], is differentially expressed in our microarray conditions (it should be noted however that *pyd* is not formally a candidate due to inconsistencies between microarray replicates; in fact only splice variant *pyd*-RE shows a response), explaining in part the requirement of Ttk in tracheal cell intercalation. In addition, it is tempting to speculate about other candidate targets to mediate this function of Ttk in intercalation, like *canoe* (*cno*) for instance, which have been recently shown to act with *pyd* during embryogenesis [38].

We have focused on the role of Ttk in tube size to validate our experimental approach. To date, several factors have been shown to control tube growth, the septate junctions (SJs) and a transient intraluminal chitin filament being two key elements (review in [16]–[18]). Among Ttk-regulated genes in our screen we have identified several with known or potential functions in chitin metabolism (included in the GO term ‘chitin metabolism’) and SJ organisation. We confirmed by qPCR the predicted transcriptional regulation observed in the microarray analysis. Remarkably, the functional requirement of some of these genes in tube size regulation could also be confirmed in our or other's experiments. Our functional approach based on the expression in the tracheal tissue of RNAi lines of chitin metabolism and SJ organisation targets of Ttk identified 40% of these genes as modulators of tube size. For instance, we identify such a role in the chitin-binding protein Cda4 which might act as a chitin deacetylase. However, we did not observe an effect on tube size using RNAi lines of several genes with expected roles in this process, like for instance *Cht2* (BR and ML, unpublished data), which has been shown to generate tube size defects upon overexpression [39]. The lack of effect of several RNAi lines tested indicate that either the downregulation of the gene does not affect the control of tube size (because the gene is not required or due to functional redundancy), or that the strength of the interfering lines is not sufficient to generate a visible effect. Further experiments would be required to distinguish between these two possibilities. Nevertheless, the fact that some RNAi lines produce extra long tracheal tubes already establishes the link between Ttk and (at least some) tracheal tube size genes. Finally, it is important to note that besides the organisation and assembly of a transient luminal chitin filament other mechanisms have also been reported to regulate the size of the tracheal tubes (reviews in [18]). Importantly, we observe that Ttk may be also controlling some of these other mechanisms, like apical secretion through Gef64C [40], apical cell membrane growth through Mmp1 [41] or planar cell polarity through ft [42] (Supplementary Table S6). Thus, Ttk would feed into a general and complex program of tube size control involving different mechanisms.

Altogether our results validate previous functional analysis and identify several targets that we show or we propose can mediate the different activities of Ttk during tracheal development.

### Ttk and Notch interactions

Our microarray analysis pointed to a regulation of the Notch signalling pathway or its activity by Ttk, likely acting as a negative regulator. On the other hand, we had previously observed that Ttk acts as a downstream effector of N activity in the specification of different tracheal identitites [1]. Indeed, we showed that Ttk levels depend on N activity in such a way that when N is active, Ttk levels are high, whereas when N is not active, Ttk levels are low. Thus, we observed lower levels of Ttk in tracheal fusion cells due to the inactivity of N there [37], [43], [44]. Therefore, Ttk acts as a target of N in fusion cell determination. Now, the results of the microarray add an extra level of complexity to the Ttk-N interaction. The observation that in turn Ttk also transcriptionally regulates several N pathway components suggests that Ttk is involved in a feedback mechanism that could play a pivotal role in biasing or amplifying N signalling outcome.

Interactions between Ttk and N have been observed in different developmental contexts, emphasising the importance of such regulations. Several examples illustrate the regulation, either positive or negative, of Ttk expression by N activity [45]–[49]. In addition, a recent report provides evidence of a regulation of N activity by Ttk and proposes a mutually repressive relationship between N and Ttk which would also involve Ecdysone signalling [15]. Our results are consistent with many of these observations, indicating that they could represent general molecular mechanisms of morphogenesis. Thus, tracheal cell specification could serve as an ideal scenario to investigate the intricate, and often contradictory, interactions between N and Ttk and the complexity of N signaling.

## Materials and Methods

### *Drosophila* stocks

In this work we use the loss-of-function allele *ttk* ^D2-50^ balanced over a TM3 Twi-GFP balancer, which allows the identification of mutant embryos by the absence of fluorescence. All gain-of-function experiments were performed using the GAL4/UAS system [50]. All the *Drosophila* stocks used are described in FlyBase. We used a transgenic line containing the btl-enh-RFP-moe insertion [51] that enables the visualization of the shape of all *btl* positive cells *in vivo*. We generated recombinants carrying btl-enh-RFP-moe and the *ttk* mutant conditions, in particular: btl-enh-RFP-moe *ttk* ^D2-50^/TM3 Twi-GFP for the mutant, and btl-enh-RFP-moe UAS-*ttk69* for the over-expression condition. We used *btl*-GAL4 UAS-tau-GFP [52] to drive *ttk* over-expression.

### Embryo and cell sorting strategy

To obtain homogeneous-as-possible embryos for RNA extraction, we devised a two-step sorting strategy for first embryos and then cells. In order to enrich the embryo collection for embryos undergoing tracheal development, we allowed flies to lay eggs for 7,40 hr at 25°C, and subsequently let the embryos age for another 5,20 hr at 25°C. This enabled us to maximize the number of embryos at the appropriate developmental stages 11–16.

Embryos were first dechorionated and washed 3× with PBS-Triton (0.1%). They were then collected into an Eppendorff with cold Schneider's medium (S2) (GIBCO). We used a COPAS PLUS Embryo Sorter (Union Biometrica) following the manufacturer's recommendations to obtain homozygous mutants that were characterised by the absence of balancer-GFP. The appropriate embryo population was selected according to their extinction/absortion profile. The gated population was analysed and sorted according to the lack of GFP expression (blue excitation, green emission). Manual examination confirmed the success of this sorting step.

For several rounds, pools of 600–1000 sorted embryos were homogenised in cold S2 medium in a crystal douncer (KONTES Gerresheimer) with three strokes without rotating the pestle. Thereafter, 60 µl of trypsin-EDTA 9X (SIGMA) was added to 1.5 ml of S2 medium and shaken for 15 minutes at room temperature to the homogenised embryonic sample. 5 µl of Hoechst-33342 nuclear marker per 600–1000 embryos was added to the trypsinisation reaction. Subsequently, the reaction was stopped with a 1∶1 PBS∶BSA buffer.

Ultimately, dissociated cells were sorted using a FacsAria SORP sorter (Beckton Dickinson, San Jose, California). Excitation of the sample was done using a blue (488 nm) laser for forward-scatter (FSC) and GFP green fluorescence; green (532 nm) laser for RFP fluorescence, and a UV laser for Hoechst-33342 fluorescence. A combination of excitation wavelengths was used to distinguish autofluorescence from fats from actual cells. Those enriched for RFP (by their expression of the btl-enh-RFP-moe construct) were selected according to their forward-scatter/side-scatter (SSC) signal. The Hoechst-33342 signal was used to eliminate debris and broken cells. The gating strategy is shown in Figure S2.

### RNA extraction

Total RNA from pools of about 1,000 embryos from the four genotypes (control, *ttk* ^D2-50^ mutant, *btl*-GAL4; btl-enh-RFP-moe and *btl*-GAL4; btl-enh-RFP-moe UAS-*ttk69*) was extracted using the GibcoBRL Trizol protocol. RNA extraction from the purified cell samples from the four genotypes were performed using the RNeasy QIAGEN RNA Extraction Kit, starting with 100,000 isolated cells of each condition. RNA was quantified using the Nanodrop ND-1000 spectrophotometer (Fisher Scientific) and integrity was evaluated on the Bioanalyzer 2100 (Agilent) using the RNA 6000 Nano Kit.

### Microarray experiments

We pooled multiple RNA collections from different cell sorting runs from the same week to minimise the variation inherent among collections and to obtain adequate RNA from each genotype. 5 ng total RNA per sample was processed using the isothermal amplification SPIA Biotin System (NuGEN Technologies, Inc.).

2.2 µg of cDNA from each genotype and for each sample condition were hybridized to GeneChip 2.0 *Drosophila* Genome Arrays (Affymetrix, Santa Clara, CA) in triplicate. The Functional Genomics Core Facility at the IRB provided all the reagents, equipment and expertise for hybridisation and scanning.

Raw microarray data has been deposited in the MIAME compliant GEO database, series GSE30239.

### Computational analysis

Raw microarray data were processed in *R* and Bioconductor [53] using the Brainarray ENSEMBL Custom v12 CDF mapping [54]. For further details on normalisation and scaling see GEO [55] data series GSE30239. All other data analysis was performed using custom-written Perl scripts, using BDGP gene expression database (March 2007) [20] and BioGrid genetic interactions (early 2011) [56] for comparison. Gene lists were managed in FlyMine [57] and analysed for Gene Ontology-enrichment using their implementation of the hypergeometric test with Benjamini-Hochberg multiple testing correction. The Ttk chromatin immunopurification data was obtained from the modENCODE website and visualised using the Integrated Genome Browser (http://bioviz.org/igb/).

### Quantitative real time qPCR

Equal amounts of total RNA of embryos at stages 11 to 16 from each condition and genotype were used to synthesise complementary cDNA by random-hexamer-priming (RevertAid H Minus First Strand cDNA Synthesis FERMENTAS Kit) in triplicate. A LightCycler 480 Real-Time PCR System and the SYBR Green PCR Master Mix (Roche) were used to amplify cDNAs. Relative quantities were normalized to expression of CG13167, a mitochondrial ATPase that showed stable expression throughout all experimental conditions. All PCR reactions were carried out in triplicate in 20 µl reaction volumes containing 2 µl cDNA template. Samples were analyzed using the LightCycler 480 Real-Time PCR System software (Roche). All PCR primers were designed using the MacVector 11.1 software, aimed to amplify a product size of 75–150 bp, and are listed in Supplementary Table S5. Expression changes were deemed significant at p<0.05.

### Stage-specific qPCR

To compare the expression profile of embryos at different stages, we collected embryos at stages 11 to 13 and embryos at stages 14 to 16. For the collection of the younger embryos we allowed flies to lay eggs for 5,20 hr at 25°C and subsequently allowed embryos to develop for another 5 hr at 25°C. For the older embryo collection we allowed flies to lay eggs for 5,20 hr at 25°C and subsequently allowed embryos to develop for another 10,20 hr at 25°C.

Embryos were dechorionated and sorted by absence of GFP using the COPAS PLUS Sorter to obtain the homozygous mutants and control embryos. Total RNA from pools of about 1,000 embryos from each genotype (control and *ttk* ^D2-50^ mutant) at each developmental stage was extracted using the GibcoBRL Trizol standard protocol. RNA was quantified using the Nanodrop ND-1000 spectrophotometer (Fisher Scientific).

Equal amounts of total RNA were used to synthesise complementary cDNA by random-hexamer-priming (RevertAid H Minus First Strand cDNA Synthesis FERMENTAS Kit) in triplicate, to perform and analyse the qPCR experiments as we describe above.

### Immunohistochemistry

Embryos were stained following standard protocols. Immunostainings were performed on embryos fixed in 4% formaldehyde for 20′. The following primary antibodies were used: anti-GFP, 1∶600 (Molecular Probes and Roche) and anti-βGal, 1∶600 (Cappel and Promega). Cy2 and Cy3-conjugated secondary antibodies (Jackson ImmunoResearch) were used at 1∶300 in PBT+0.5%BSA. Chitin was visualised with Chitin-binding protein CBP (NEB) at 1∶500. Confocal images were obtained with a Leica TCS-SPE system. Images were imported into Adobe Photoshop and assembled into figures using the Adobe Illustrator software.

### Tube length measurements

*Drosophila* embryos from mid-late stage 16 and stage 17 were stained with CBP and GFP to visualise their tracheal system. Digital micrographs of dorsal trunks were used to measure their length adapting a previously published method [22]. The significance of tracheal length differences between controls, RNAi knockdown mutants and the available loss-of-function alleles, measured in ImageJ, was determined using Wilcoxon's test in *R*.

## Supporting Information

Figure S1 Quality control plots. Microarray quality control before normalization. Panel (A) shows histograms and panel (B) shows box plots, each grouped by experiment. The outcomes of cell sorting experiments are generally close to the corresponding whole-embryo outcomes. The MA plots in panel (C) indicate that the arrays are well comparable within each experiment.
(TIF)

Figure S2 Gating strategy for FACS-assisted cell sorting. Examples for the isolation of RFP positive cells from a whole embryo cell suspension. Regions are drawn to include the population of interest. (Panel 1) Data for mutant control cell suspension. (1A) Region in A is drawn and placed to include most of the events that exhibit similar SSC and FSC values. (1B) P5 region is drawn and placed to include live cells with only 2C DNA content, which are the cells at G0/G1 cell cycle phase. (1C) P4 region is drawn and placed to distinguish RFP-positive from RFP-negative cells with respect to relative cell size and fluorescence. (Panels 2–4) show equivalent plots. (Panel 2) Data for *ttk* ^D2-50^ mutant cell suspension. (Panel 3) Data for control UAS-experiment cell suspension. (Panel4) Data for UAS-*ttk69* cell suspension.
(TIF)

Figure S3 Spatial gene expression representation in microarray signal categories. Spatial gene expression pattern representation in different microarray signal strength categories. Gene expression profiles from enriched cell populations for the control group in the (A) mutant and (B) over-expression are shown. Within the signal intensity histogram (x-axis: bins of log_2_ signal strength, y-axis: number of genes), the proportion of genes falling into one of four different spatial gene expression categories is shown: genes with tracheal or midline expression (red), genes with ubiquitous expression (blue), genes with expression elsewhere (light gray), genes not expressed according to the BDGP *in situ* database (dark gray).
(TIF)

Figure S4 Cluster diagram of genes involved in chitin metabolic processes. Genes are clustered according to the gene expression changes in response to Ttk-mis-expression (from bottom to top: over-expression in cells, over-expression in embryos, mutant cells and mutant embryos) as well as Ttk occupancy in the modENCODE dataset (ChIP enrichment> = lg2(1) over the gene body +/−1 kb; blue indicates occupancy and yellow non-occupancy).
(PDF)

Table S1 Number of putative candidate genes after *ttk* mis-expression.
(XLS)

Table S2 Comparison of raw signal intensities for selected marker genes.
(XLS)

Table S3 Expression enrichment in FACS-sorted cell populations.
(XLS)

Table S4 Over-represented Gene Ontology categories.
(XLS)

Table S5 Real-time PCR primer sequences for target validation.
(DOC)

Table S6 Selected Gene Ontology terms with putative involvement in tracheal development, and genes with that annotation which are mis-regulated either in our microarray dataset or in S2 cells.
(XLS)

Table S7 Analysis of stage-specific regulation by quantitative PCR.
(DOC)

Text S1 Quality control of microarrays.
(DOC)

File S1 Archive (gzip2 compressed tar ball format) with 82 PDF files showing scatter plots for GO biological process terms with more than 50 annotated genes. Legend analogous to Figure 2.
(BZ2)

## References

[1] Araújo SJ, Cela C, Llimargas M (2007). Tramtrack regulates different morphogenetic events during Drosophila tracheal development.. Development.

[2] Harrison SD, Travers AA (1990). The tramtrack gene encodes a Drosophila finger protein that interacts with the ftz transcriptional regulatory region and shows a novel embryonic expression pattern.. EMBO J.

[3] Brown JL, Sonoda S, Ueda H, Scott MP, Wu C (1991). Repression of the Drosophila fushi tarazu (ftz) segmentation gene.. EMBO J.

[4] Read D, Manley JL (1992). Alternatively spliced transcripts of the Drosophila tramtrack gene encode zinc finger proteins with distinct DNA binding specificities.. EMBO J.

[5] Brown JL, Wu C (1993). Repression of Drosophila pair-rule segmentation genes by ectopic expression of tramtrack.. Development.

[6] Guo M, Bier E, Jan LY, Jan YN (1995). tramtrack acts downstream of numb to specify distinct daughter cell fates during asymmetric cell divisions in the Drosophila PNS.. Neuron.

[7] Lai ZC, Li Y (1999). Tramtrack69 is positively and autonomously required for Drosophila photoreceptor development.. Genetics.

[8] Badenhorst P (2001). Tramtrack controls glial number and identity in the Drosophila embryonic CNS.. Development.

[9] Chen Y-J, Chiang C-S, Weng L-C, Lengyel JA, Liaw G-J (2002). Tramtrack69 is required for the early repression of tailless expression.. Mech Dev.

[10] Baonza A, Murawsky CM, Travers AA, Freeman M (2002). Pointed and Tramtrack69 establish an EGFR-dependent transcriptional switch to regulate mitosis.. Nat Cell Biol.

[11] French RL, Cosand KA, Berg CA (2003). The Drosophila female sterile mutation twin peaks is a novel allele of tramtrack and reveals a requirement for Ttk69 in epithelial morphogenesis.. Dev Biol.

[12] Althauser C, Jordan KC, Deng W-M, Ruohola-Baker H (2005). Fringe-dependent notch activation and tramtrack function are required for specification of the polar cells in Drosophila oogenesis.. Dev Dyn.

[13] Audibert A, Simon F, Gho M (2005). Cell cycle diversity involves differential regulation of Cyclin E activity in the Drosophila bristle cell lineage.. Development.

[14] Siddall NA, Hime GR, Pollock JA, Batterham P (2009). Ttk69-dependent repression of lozenge prevents the ectopic development of R7 cells in the Drosophila larval eye disc.. BMC Dev Biol.

[15] Boyle MJ, Berg CA (2009). Control in time and space: Tramtrack69 cooperates with Notch and Ecdysone to repress ectopic fate and shape changes during Drosophila egg chamber maturation.. Development.

[16] Wu VM, Beitel GJ (2004). A junctional problem of apical proportions: epithelial tube-size control by septate junctions in the Drosophila tracheal system.. Curr Opin Cell Biol.

[17] Swanson LE, Beitel GJ (2006). Tubulogenesis: an inside job.. Curr Biol.

[18] Schottenfeld J, Song Y, Ghabrial AS (2010). Tube continued: morphogenesis of the Drosophila tracheal system.. Curr Opin Cell Biol.

[19] Glazer L, Shilo BZ (1991). The Drosophila FGF-R homolog is expressed in the embryonic tracheal system and appears to be required for directed tracheal cell extension.. Genes Dev.

[20] Tomancak P, Berman BP, Beaton A, Weiszmann R, Kwan E (2007). Global analysis of patterns of gene expression during Drosophila embryogenesis.. Genome Biol.

[21] Reddy BA, Bajpe PK, Bassett A, Moshkin YM, Kozhevnikova E (2010). Drosophila transcription factor Tramtrack69 binds MEP1 to recruit the chromatin remodeler NuRD.. Mol Cell Biol.

[22] Laprise P, Paul SM, Boulanger J, Robbins RM, Beitel GJ (2010). Epithelial polarity proteins regulate Drosophila tracheal tube size in parallel to the luminal matrix pathway.. Curr Biol.

[23] Jiang L, Pearson JC, Crews ST (2010). Diverse modes of Drosophila tracheal fusion cell transcriptional regulation.. Mech Dev.

[24] Roy S, Ernst J, Kharchenko PV, Kheradpour P, Negre N (2010). Identification of functional elements and regulatory circuits by Drosophila modENCODE.. Science.

[25] Ghabrial A, Luschnig S, Metzstein MM, Krasnow MA (2003). Branching morphogenesis of the Drosophila tracheal system.. Annu Rev Cell Dev Biol.

[26] Ohshiro T, Saigo K (1997). Transcriptional regulation of breathless FGF receptor gene by binding of TRACHEALESS/dARNT heterodimers to three central midline elements in Drosophila developing trachea.. Development.

[27] Bauer DC, Buske FA, Bailey TL (2010). Dual-functioning transcription factors in the developmental gene network of Drosophila melanogaster.. BMC Bioinformatics.

[28] Jiang L, Rogers SL, Crews ST (2007). The Drosophila Dead end Arf-like3 GTPase controls vesicle trafficking during tracheal fusion cell morphogenesis.. Dev Biol.

[29] Gervais L, Casanova J (2010). In vivo coupling of cell elongation and lumen formation in a single cell.. Curr Biol.

[30] Lee M, Lee S, Zadeh AD, Kolodziej PA (2003). Distinct sites in E-cadherin regulate different steps in Drosophila tracheal tube fusion.. Development.

[31] Kakihara K, Shinmyozu K, Kato K, Wada H, Hayashi S (2008). Conversion of plasma membrane topology during epithelial tube connection requires Arf-like 3 small GTPase in Drosophila.. Mech Dev.

[32] Brodu V, Baffet AD, Le Droguen P-M, Casanova J, Guichet A (2010). A developmentally regulated two-step process generates a noncentrosomal microtubule network in Drosophila tracheal cells.. Dev Cell.

[33] Jordan KC, Schaeffer V, Fischer KA, Gray EE, Ruohola-Baker H (2006). Notch signaling through tramtrack bypasses the mitosis promoting activity of the JNK pathway in the mitotic-to-endocycle transition of Drosophila follicle cells.. BMC Dev Biol.

[34] Tanaka-Matakatsu M, Uemura T, Oda H, Takeichi M, Hayashi S (1996). Cadherin-mediated cell adhesion and cell motility in Drosophila trachea regulated by the transcription factor Escargot.. Development.

[35] Samakovlis C, Manning G, Steneberg P, Hacohen N, Cantera R (1996). Genetic control of epithelial tube fusion during Drosophila tracheal development.. Development.

[36] Steneberg P, Englund C, Kronhamn J, Weaver TA, Samakovlis C (1998). Translational readthrough in the hdc mRNA generates a novel branching inhibitor in the drosophila trachea.. Genes Dev.

[37] Llimargas M (1999). The Notch pathway helps to pattern the tips of the Drosophila tracheal branches by selecting cell fates.. Development.

[38] Choi W, Jung K-C, Nelson KS, Bhat MA, Beitel GJ (2011). The single Drosophila ZO-1 protein Polychaetoid regulates embryonic morphogenesis in coordination with Canoe/afadin and Enabled.. Mol Biol Cell.

[39] Tonning A, Hemphälä J, Tång E, Nannmark U, Samakovlis C (2005). A transient luminal chitinous matrix is required to model epithelial tube diameter in the Drosophila trachea.. Dev Cell.

[40] Massarwa R, Schejter ED, Shilo B-Z (2009). Apical secretion in epithelial tubes of the Drosophila embryo is directed by the Formin-family protein Diaphanous.. Dev Cell.

[41] Page-McCaw A, Serano J, Santé JM, Rubin GM (2003). Drosophila matrix metalloproteinases are required for tissue remodeling, but not embryonic development.. Dev Cell.

[42] Chung S, Vining MS, Bradley PL, Chan C-C, Wharton KA (2009). Serrano (sano) functions with the planar cell polarity genes to control tracheal tube length.. PLoS Genet.

[43] Steneberg P, Hemphälä J, Samakovlis C (1999). Dpp and Notch specify the fusion cell fate in the dorsal branches of the Drosophila trachea.. Mech Dev.

[44] Ikeya T, Hayashi S (1999). Interplay of Notch and FGF signaling restricts cell fate and MAPK activation in the Drosophila trachea.. Development.

[45] Guo M, Jan LY, Jan YN (1996). Control of daughter cell fates during asymmetric division: interaction of Numb and Notch.. Neuron.

[46] Okabe M, Imai T, Kurusu M, Hiromi Y, Okano H (2001). Translational repression determines a neuronal potential in Drosophila asymmetric cell division.. Nature.

[47] Ward EJ, Zhou X, Riddiford LM, Berg CA, Ruohola-Baker H (2006). Border of Notch activity establishes a boundary between the two dorsal appendage tube cell types.. Dev Biol.

[48] Sun J, Smith L, Armento A, Deng W-M (2008). Regulation of the endocycle/gene amplification switch by Notch and ecdysone signaling.. J Cell Biol.

[49] Mourikis P, Lake RJ, Firnhaber CB, DeDecker BS (2010). Modifiers of notch transcriptional activity identified by genome-wide RNAi.. BMC Dev Biol.

[50] Brand AH, Perrimon N (1993). Targeted gene expression as a means of altering cell fates and generating dominant phenotypes.. Development.

[51] Cabernard C, Affolter M (2005). Distinct roles for two receptor tyrosine kinases in epithelial branching morphogenesis in Drosophila.. Dev Cell.

[52] Cela C, Llimargas M (2006). Egfr is essential for maintaining epithelial integrity during tracheal remodelling in Drosophila.. Development.

[53] Gentleman RC, Carey VJ, Bates DM, Bolstad B, Dettling M (2004). Bioconductor: open software development for computational biology and bioinformatics.. Genome Biol.

[54] Wang P, Ding F, Chiang H, Thompson RC, Watson SJ (2002). ProbeMatchDB–a web database for finding equivalent probes across microarray platforms and species.. Bioinformatics.

[55] Barrett T, Troup DB, Wilhite SE, Ledoux P, Evangelista C (2011). NCBI GEO: archive for functional genomics data sets–10 years on.. Nucleic Acids Res.

[56] Stark C, Breitkreutz B-J, Chatr-Aryamontri A, Boucher L, Oughtred R (2011). The BioGRID Interaction Database: 2011 update.. Nucleic Acids Res.

[57] Lyne R, Smith R, Rutherford K, Wakeling M, Varley A (2007). FlyMine: an integrated database for Drosophila and Anopheles genomics.. Genome Biol.

