# Supplementary figures and images for: Tramtrack Is Genetically Upstream of Genes Controlling Tracheal Tube Size in Drosophila
Source: PLoS One. 2011 Dec 22;6(12):e28985. doi: 10.1371/journal.pone.0028985 (PMC3245245; doi:10.1371/journal.pone.0028985)

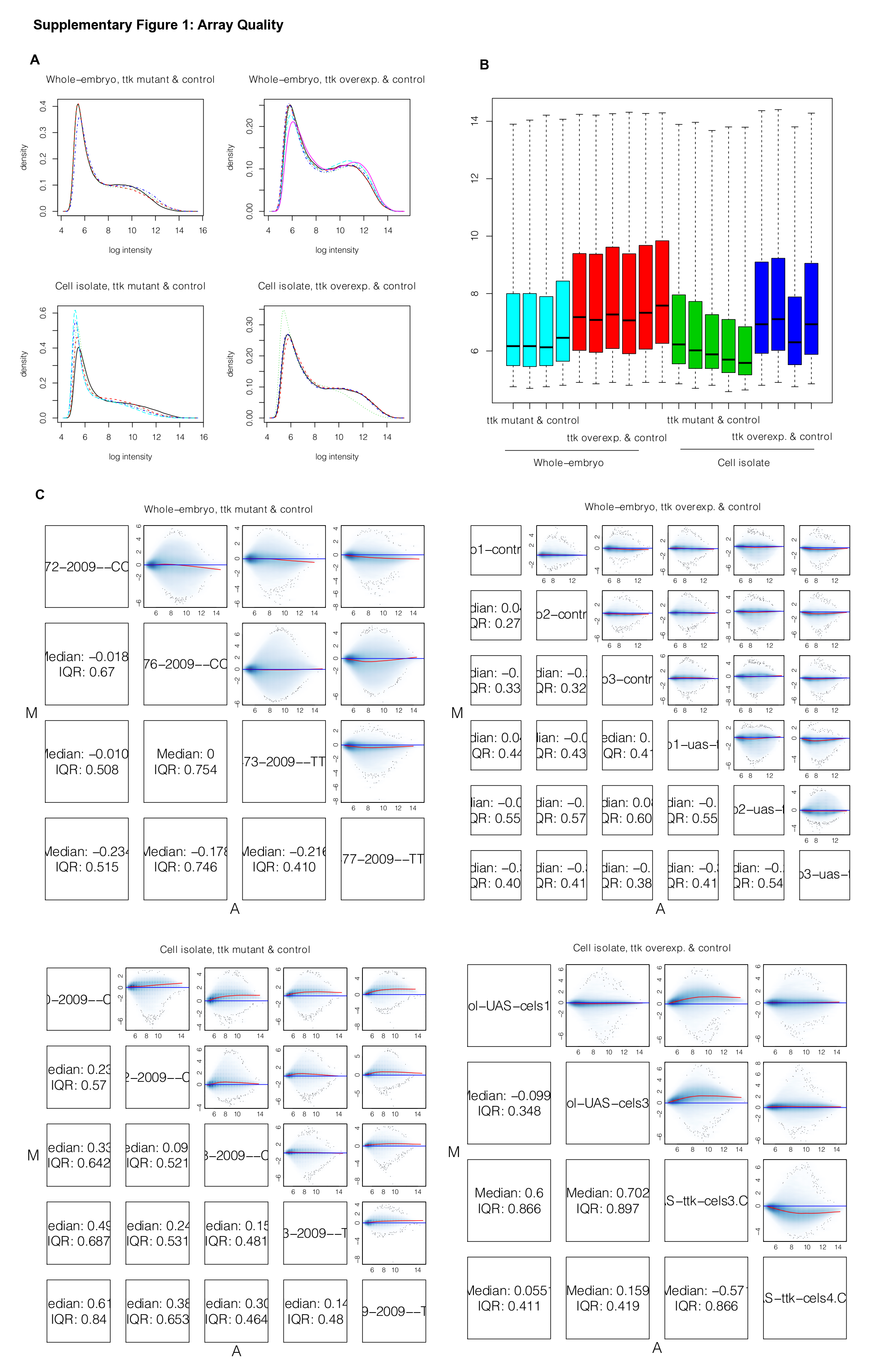

Supplement: Figure S1 — Quality control plots. Microarray quality control before normalization. Panel (A) shows histograms and panel (B) shows box plots, each grouped by experiment. The outcomes of cell sorting experiments are generally close to the corresponding whole-embryo outcomes. The MA plots in panel (C) indicate that the arrays are well comparable within each experiment. (TIF) [file pone.0028985.s001.tif]

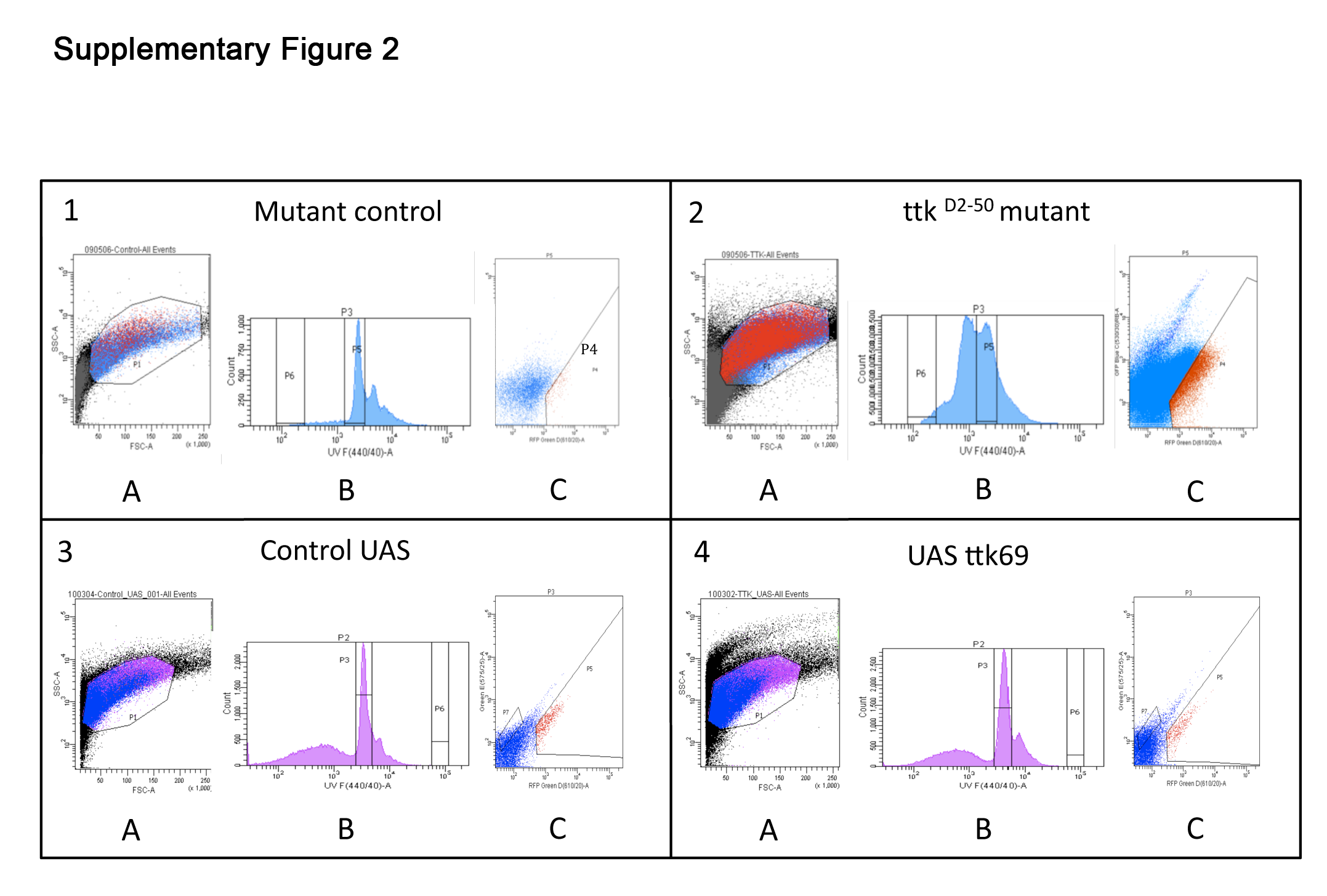

Supplement: Figure S2 — Gating strategy for FACS-assisted cell sorting. Examples for the isolation of RFP positive cells from a whole embryo cell suspension. Regions are drawn to include the population of interest. (Panel 1) Data for mutant control cell suspension. (1A) Region in A is drawn and placed to include most of the events that exhibit similar SSC and FSC values. (1B) P5 region is drawn and placed to include live cells with only 2C DNA content, which are the cells at G0/G1 cell cycle phase. (1C) P4 region is drawn and placed to distinguish RFP-positive from RFP-negative cells with respect to relative cell size and fluorescence. (Panels 2–4) show equivalent plots. (Panel 2) Data for ttk D2-50 mutant cell suspension. (Panel 3) Data for control UAS-experiment cell suspension. (Panel4) Data for UAS-ttk69 cell suspension. (TIF) [file pone.0028985.s002.tif]

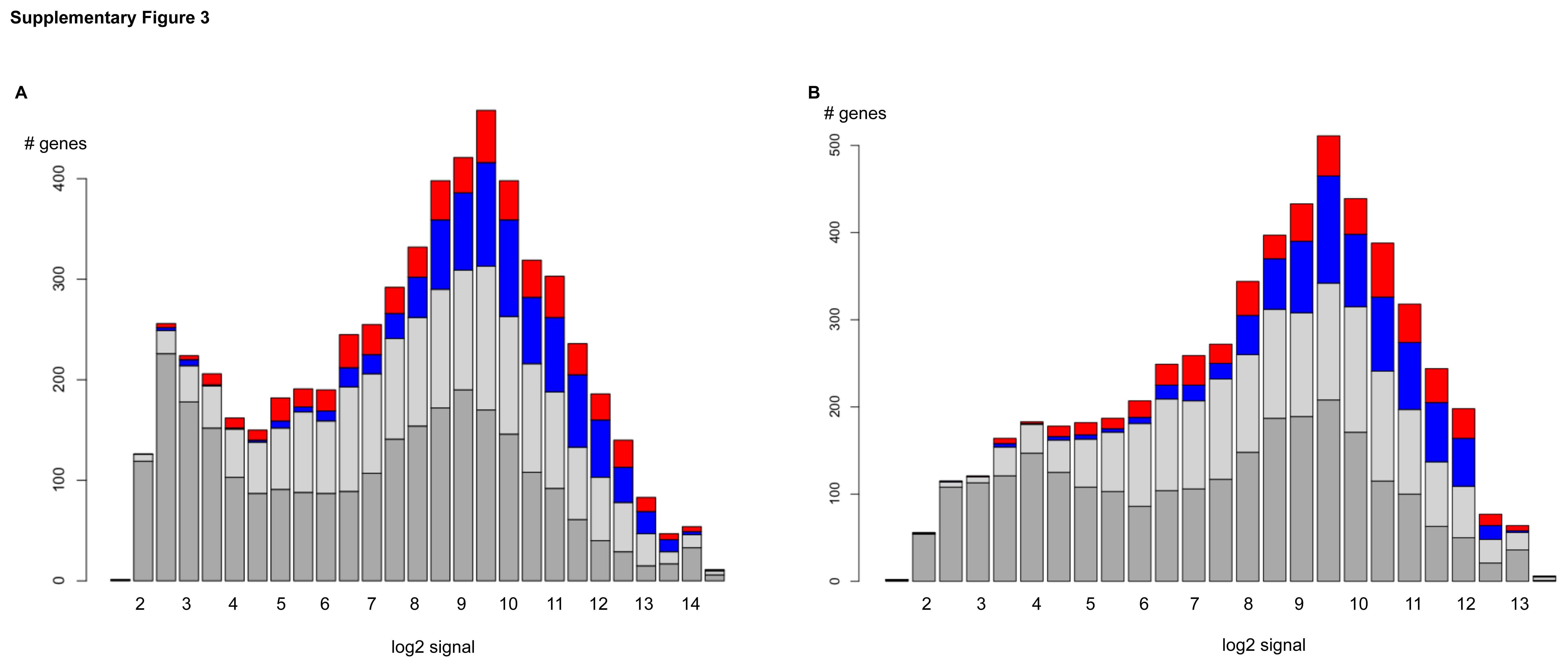

Supplement: Figure S3 — Spatial gene expression representation in microarray signal categories. Spatial gene expression pattern representation in different microarray signal strength categories. Gene expression profiles from enriched cell populations for the control group in the (A) mutant and (B) over-expression are shown. Within the signal intensity histogram (x-axis: bins of log2 signal strength, y-axis: number of genes), the proportion of genes falling into one of four different spatial gene expression categories is shown: genes with tracheal or midline expression (red), genes with ubiquitous expression (blue), genes with expression elsewhere (light gray), genes not expressed according to the BDGP in situ database (dark gray). (TIF) [file pone.0028985.s003.tif]

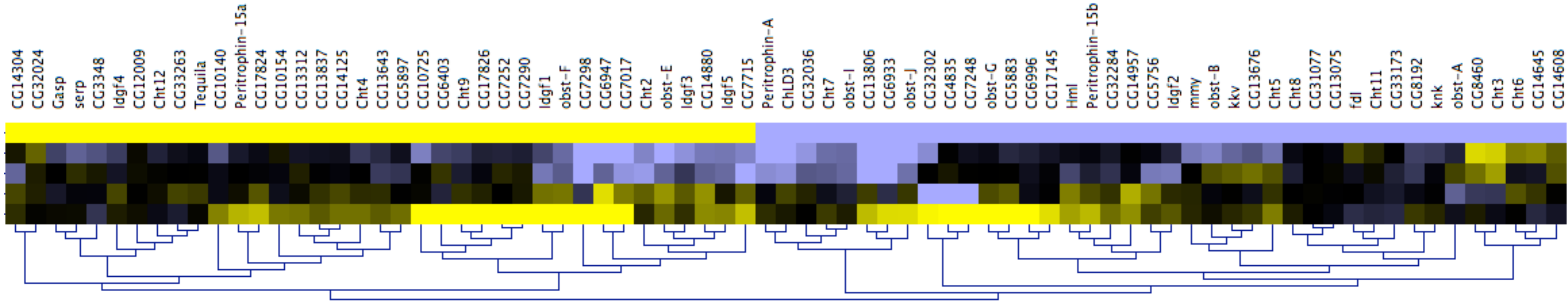

Supplement: Figure S4 — Cluster diagram of genes involved in chitin metabolic processes. Genes are clustered according to the gene expression changes in response to Ttk-mis-expression (from bottom to top: over-expression in cells, over-expression in embryos, mutant cells and mutant embryos) as well as Ttk occupancy in the modENCODE dataset (ChIP enrichment> = lg2(1) over the gene body +/−1 kb; blue indicates occupancy and yellow non-occupancy). (PDF) [file pone.0028985.s004.pdf]

### genes in actin filament organization

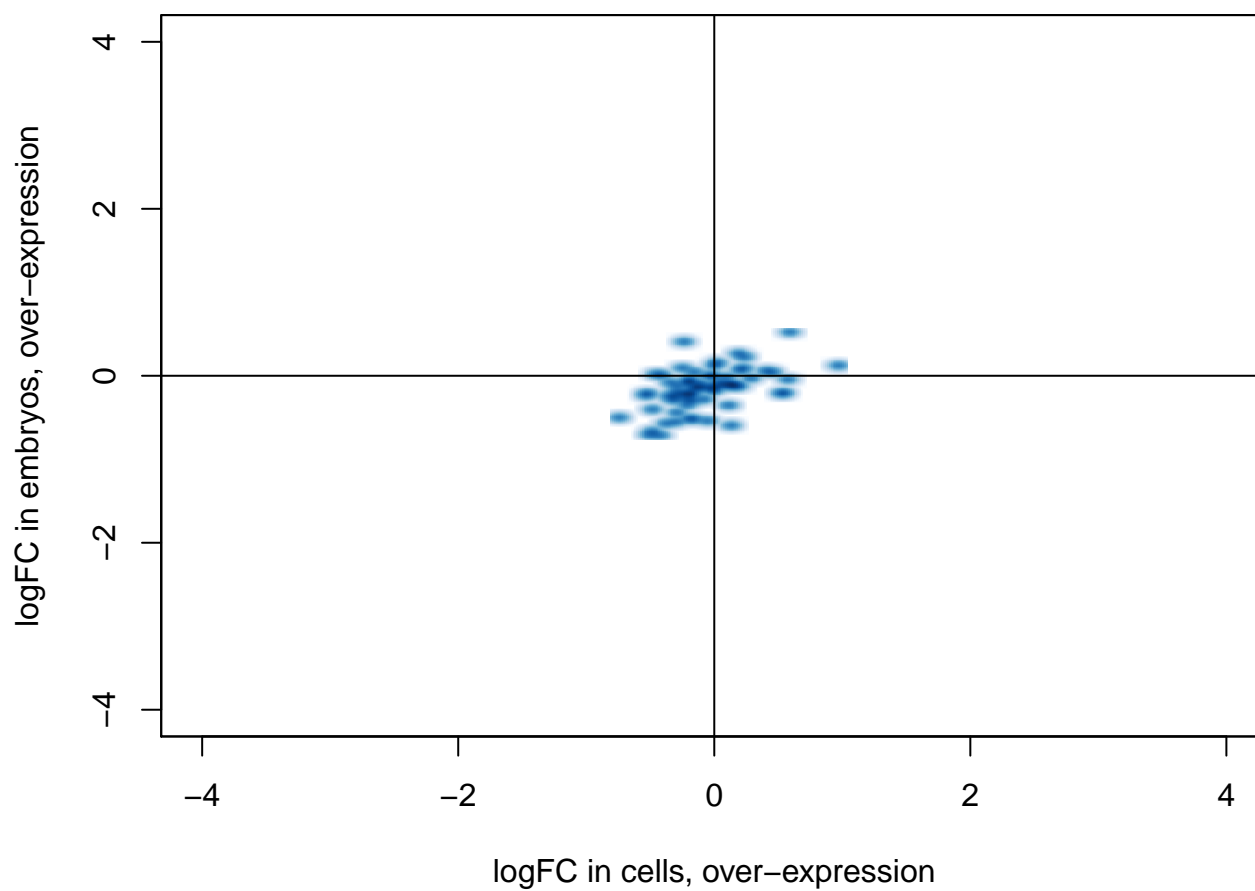

### genes in actin filament organization

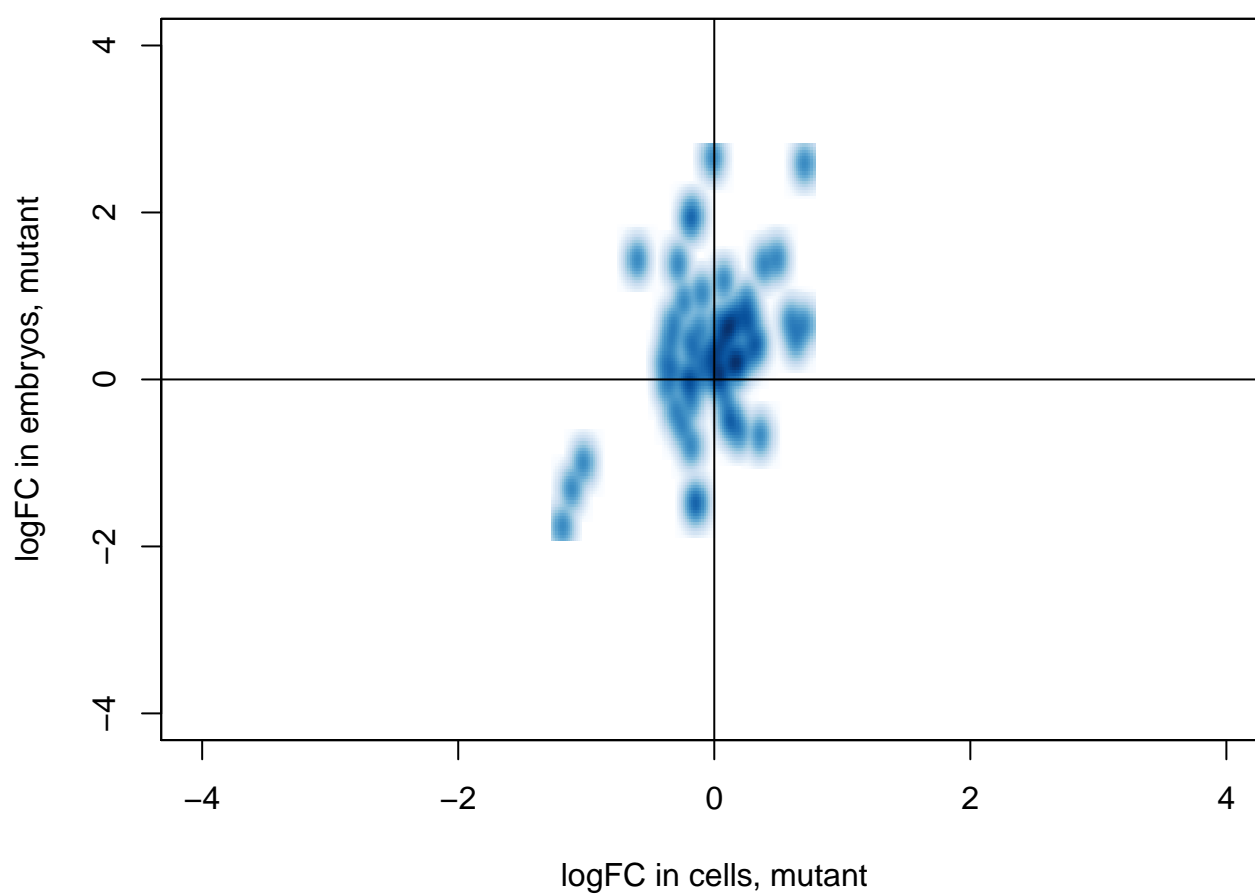

Supplement: File S1 — Archive (gzip2 compressed tar ball format) with 82 PDF files showing scatter plots for GO biological process terms with more than 50 annotated genes. Legend analogous to Figure 2. (BZ2) [file pone.0028985.s013.bz2 › scatterplots for 82 GO process terms with more than 50 genes/actin filament organization-results.pdf]

### genes in apoptosis

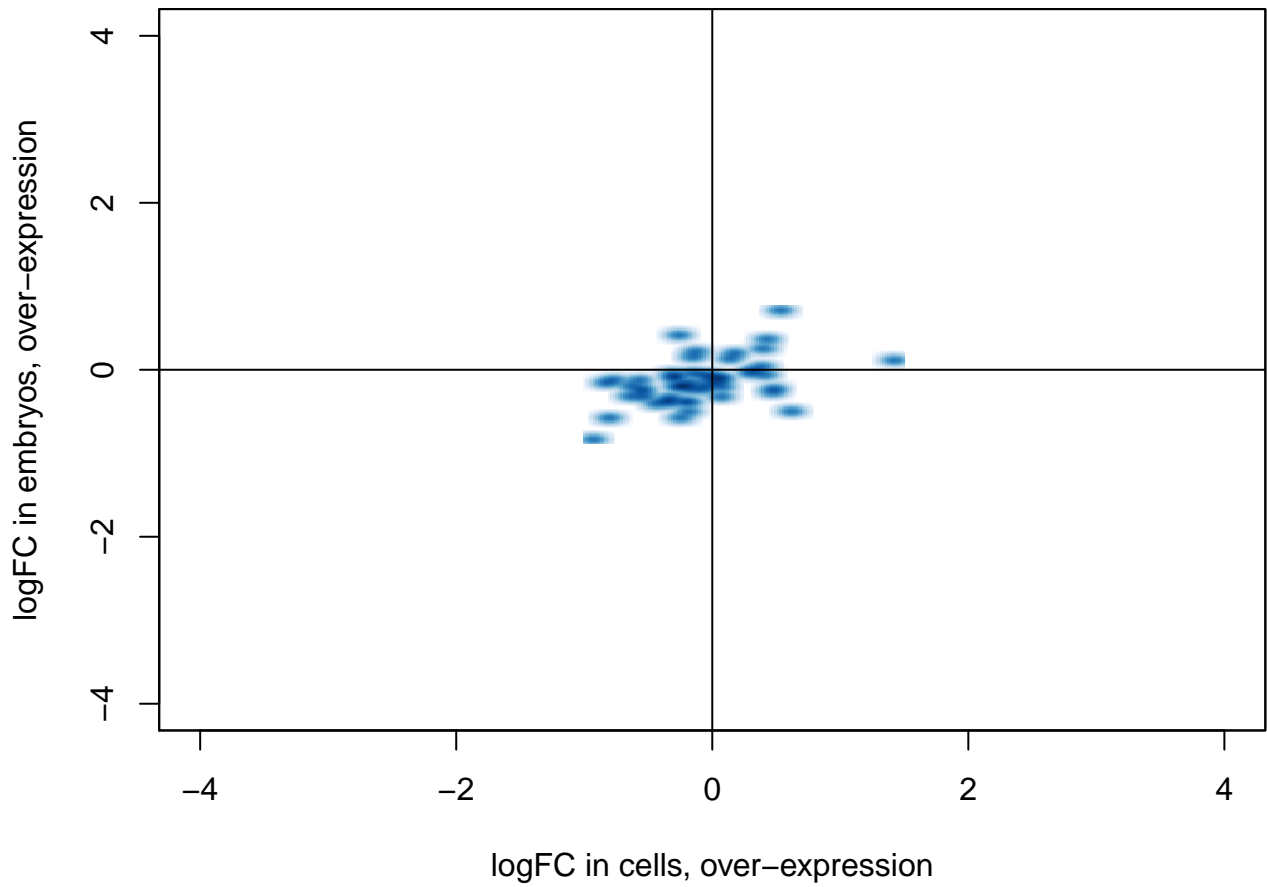

### genes in apoptosis

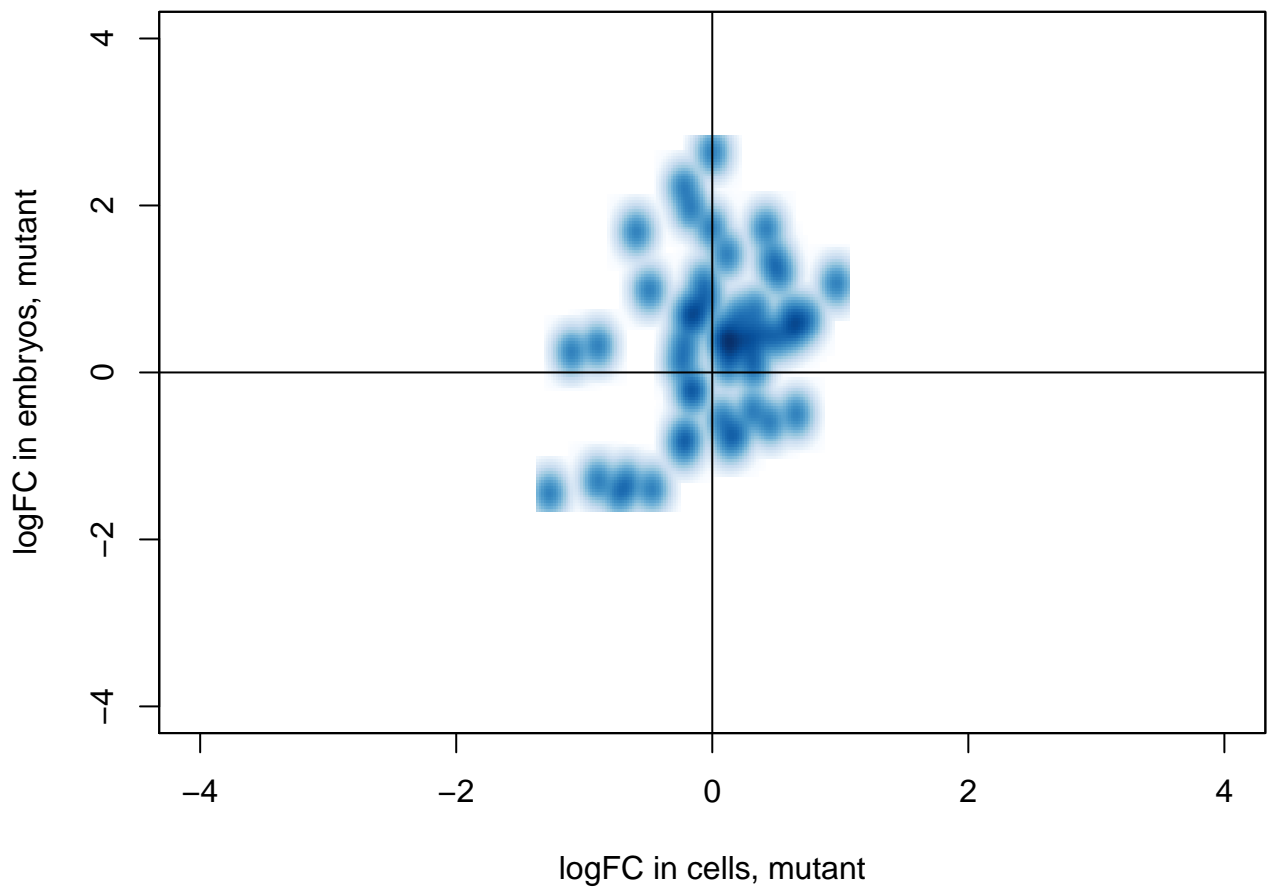

Supplement: File S1 — Archive (gzip2 compressed tar ball format) with 82 PDF files showing scatter plots for GO biological process terms with more than 50 annotated genes. Legend analogous to Figure 2. (BZ2) [file pone.0028985.s013.bz2 › scatterplots for 82 GO process terms with more than 50 genes/apoptosis-results.pdf]

**genes in autophagic cell death**

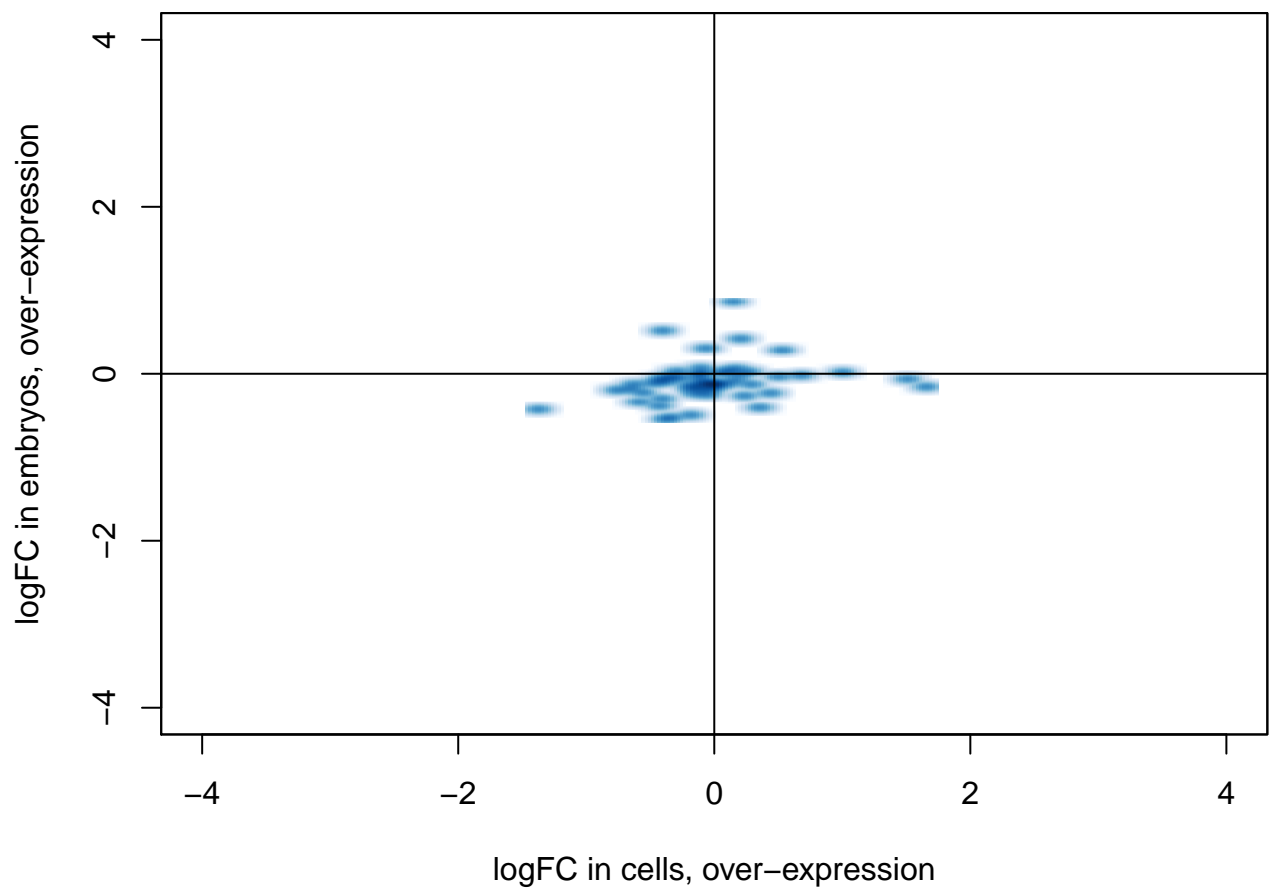

**genes in autophagic cell death**

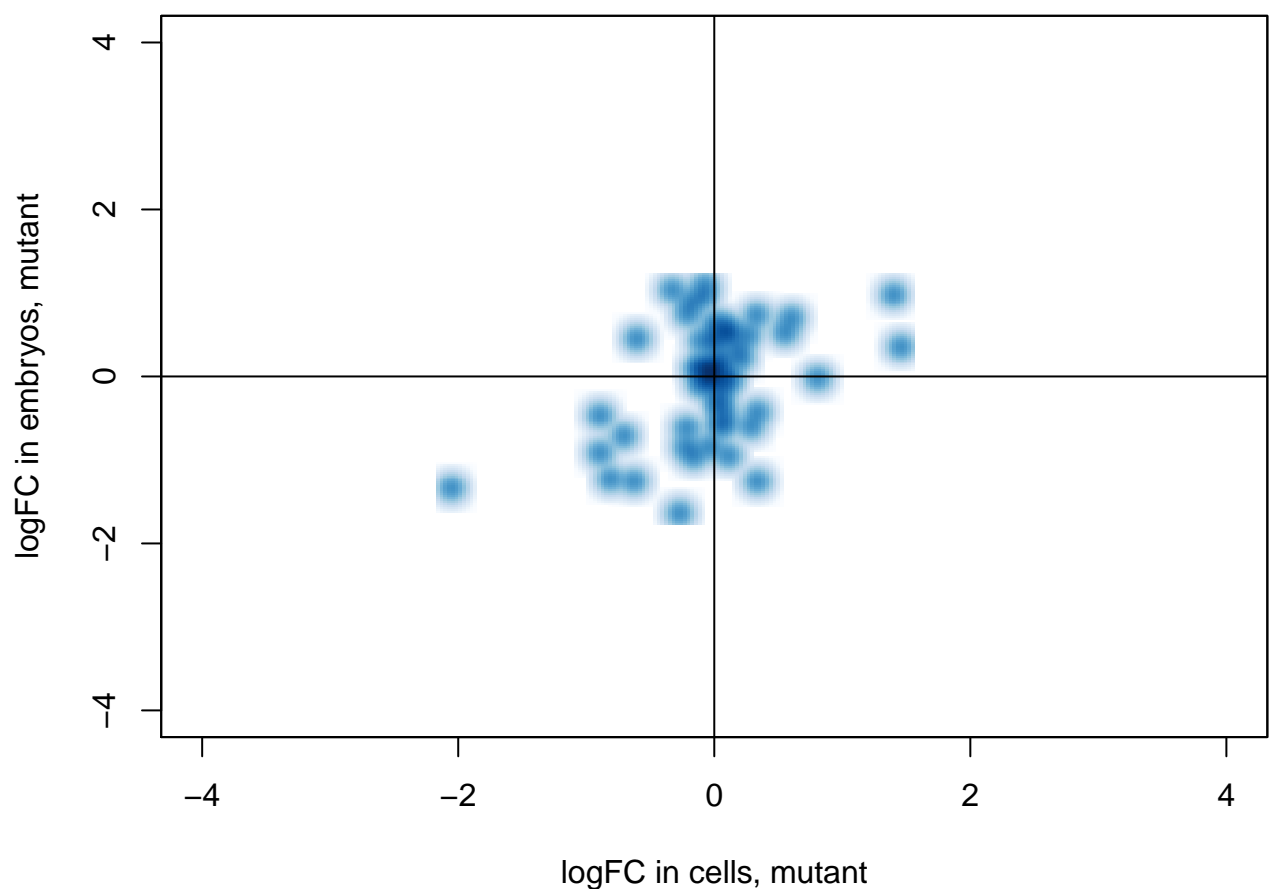

Supplement: File S1 — Archive (gzip2 compressed tar ball format) with 82 PDF files showing scatter plots for GO biological process terms with more than 50 annotated genes. Legend analogous to Figure 2. (BZ2) [file pone.0028985.s013.bz2 › scatterplots for 82 GO process terms with more than 50 genes/autophagic cell death-results.pdf]

### genes in axon guidance

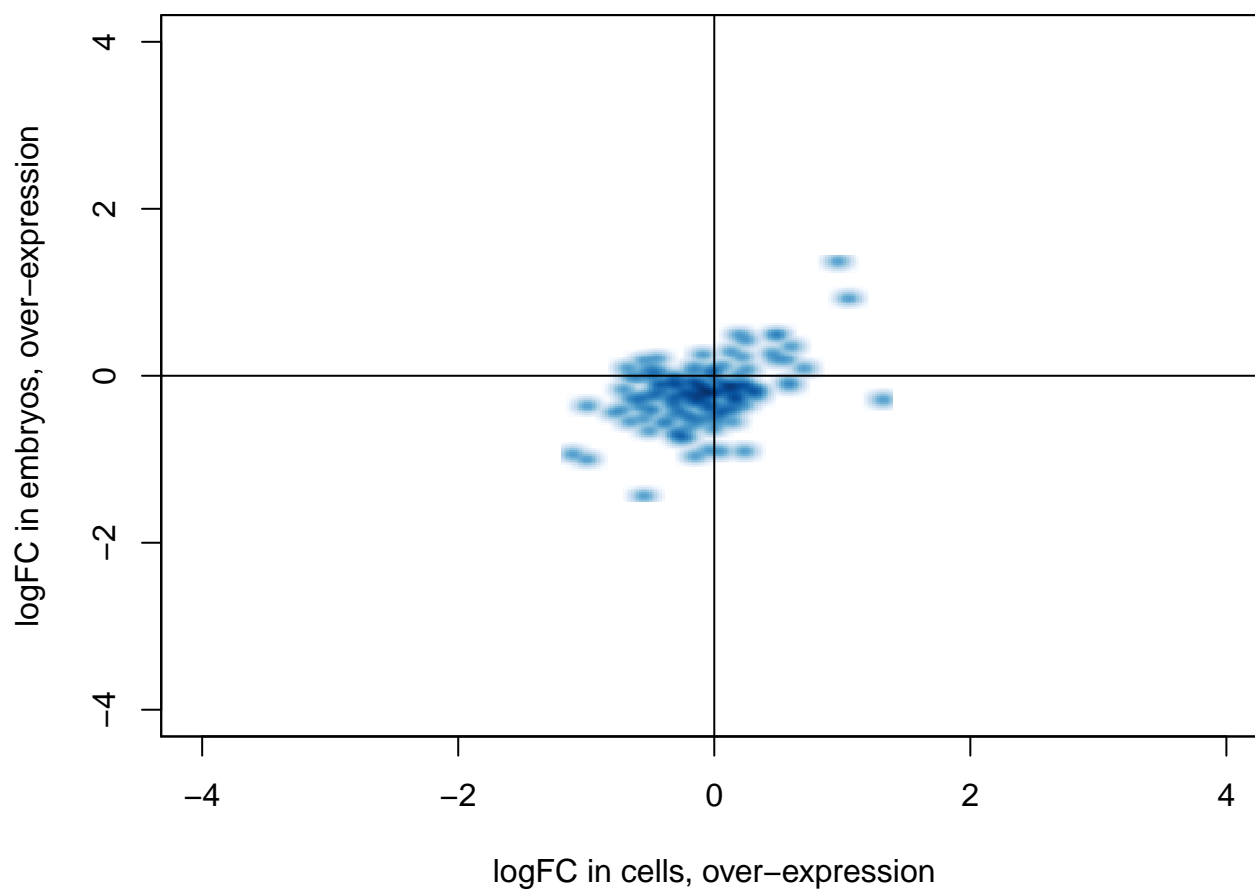

### genes in axon guidance

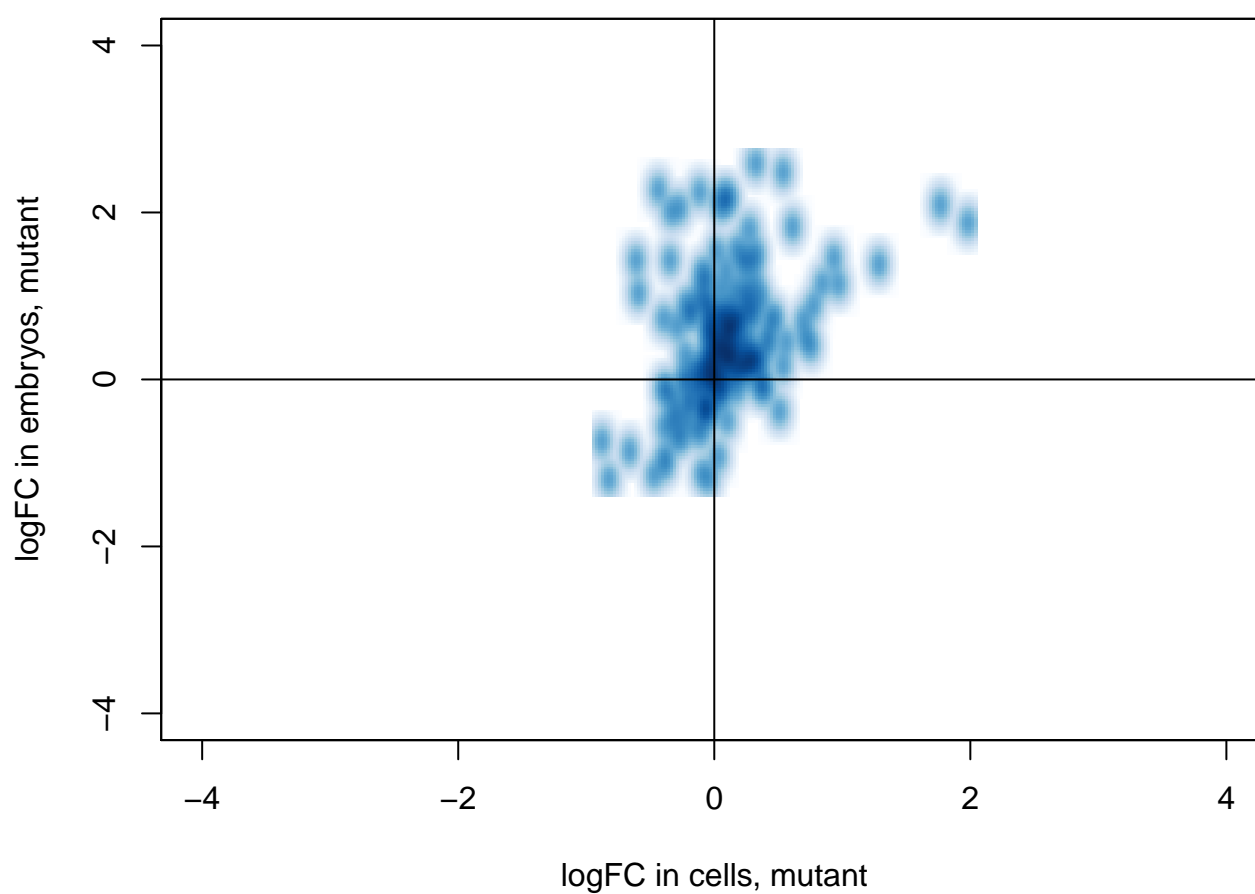

Supplement: File S1 — Archive (gzip2 compressed tar ball format) with 82 PDF files showing scatter plots for GO biological process terms with more than 50 annotated genes. Legend analogous to Figure 2. (BZ2) [file pone.0028985.s013.bz2 › scatterplots for 82 GO process terms with more than 50 genes/axon guidance-results.pdf]

### genes in biological\_process

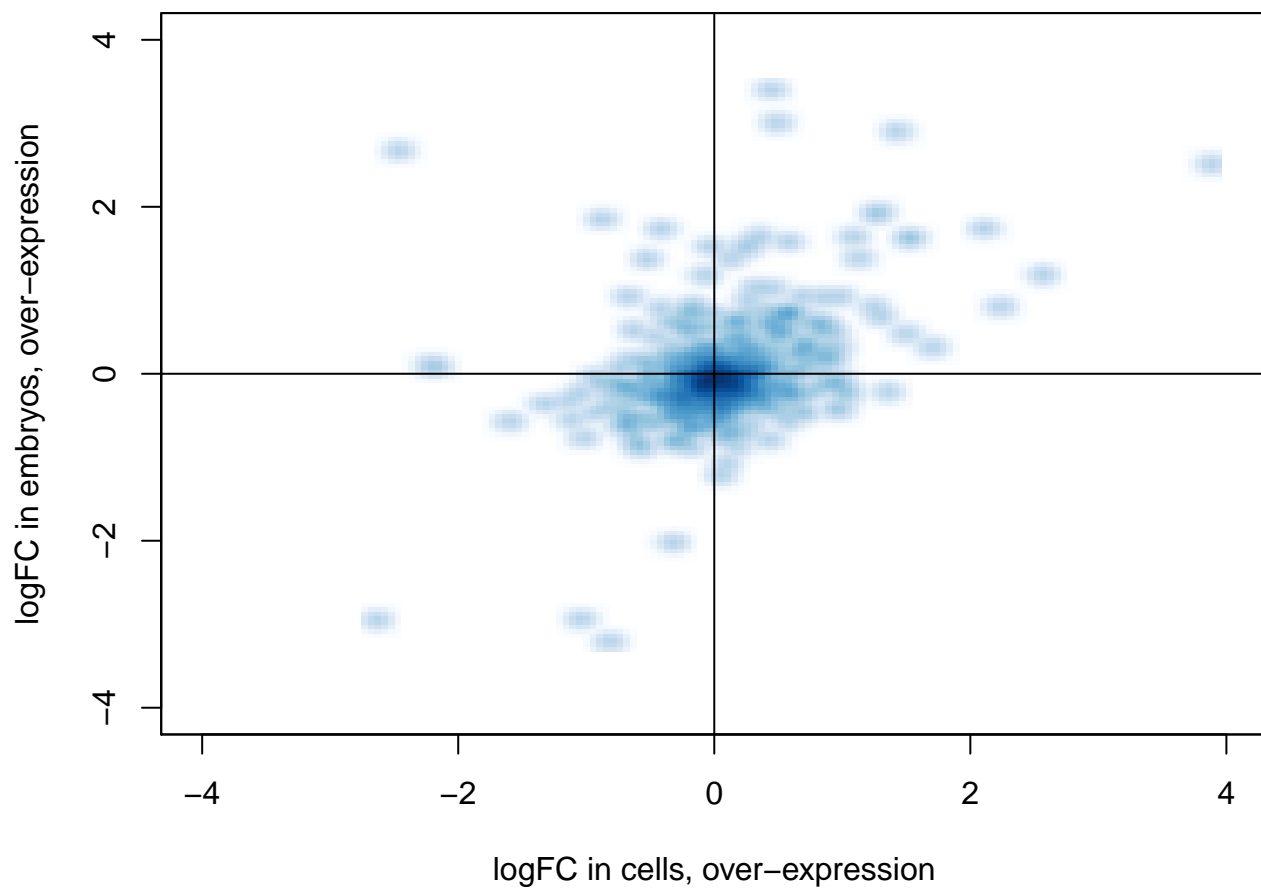

### genes in biological\_process

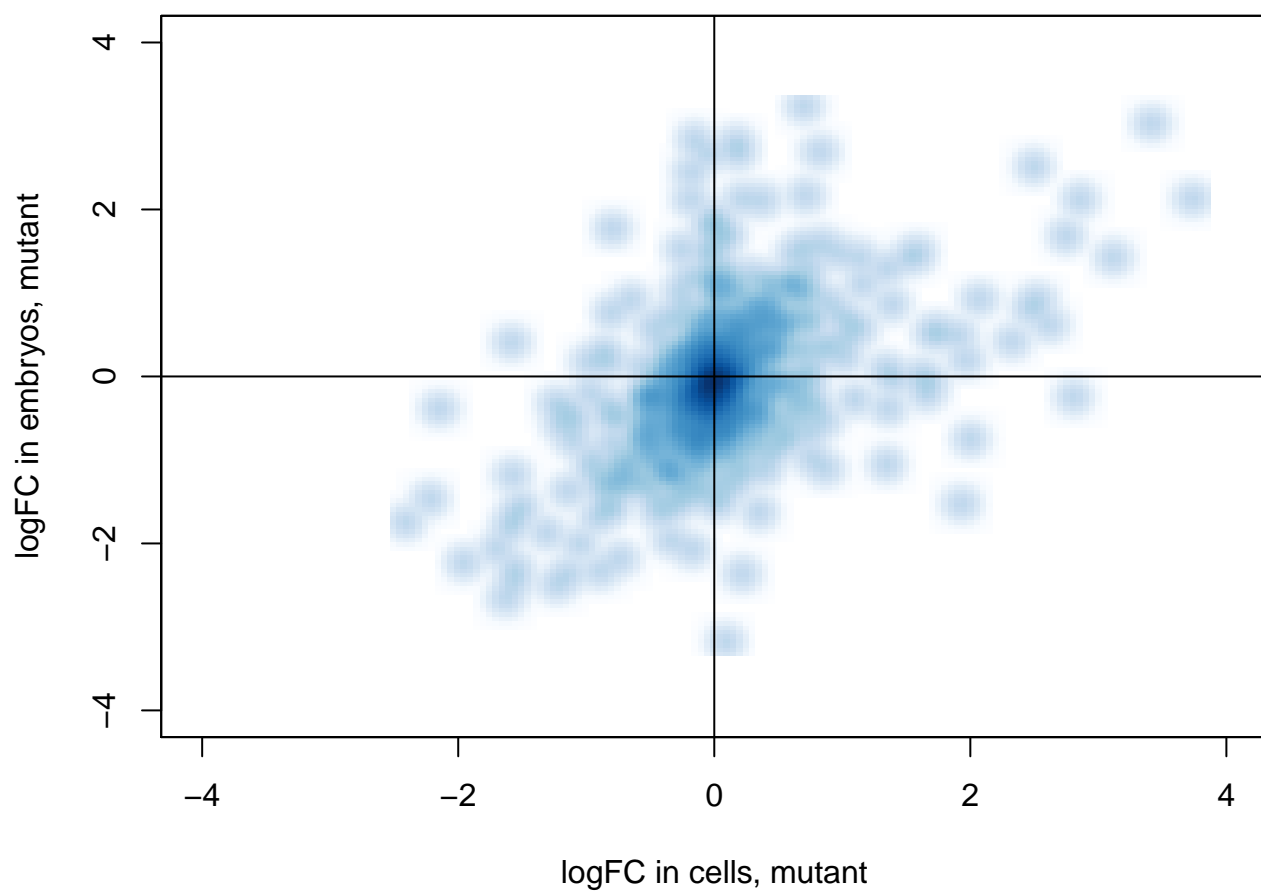

Supplement: File S1 — Archive (gzip2 compressed tar ball format) with 82 PDF files showing scatter plots for GO biological process terms with more than 50 annotated genes. Legend analogous to Figure 2. (BZ2) [file pone.0028985.s013.bz2 › scatterplots for 82 GO process terms with more than 50 genes/biological_process-results.pdf]

### genes in border follicle cell migration

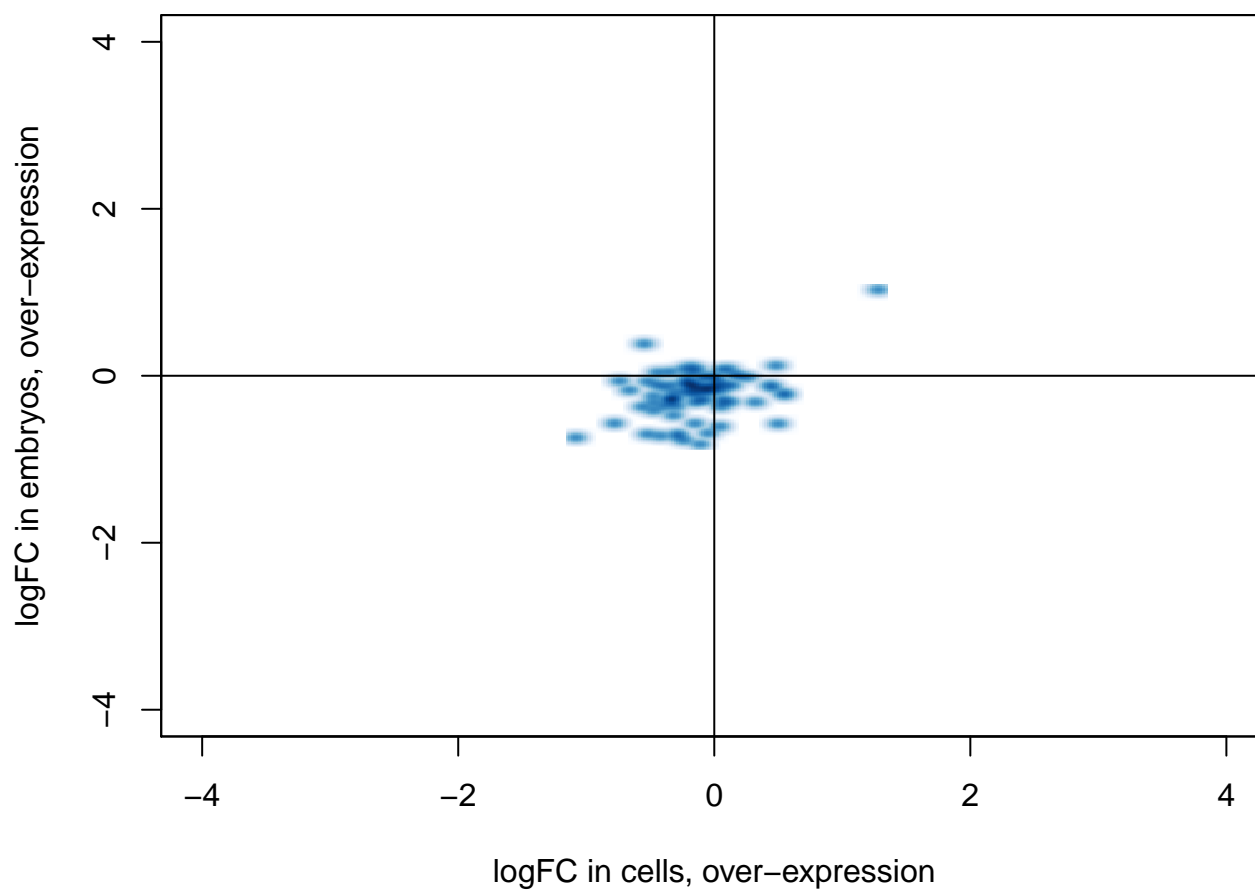

### genes in border follicle cell migration

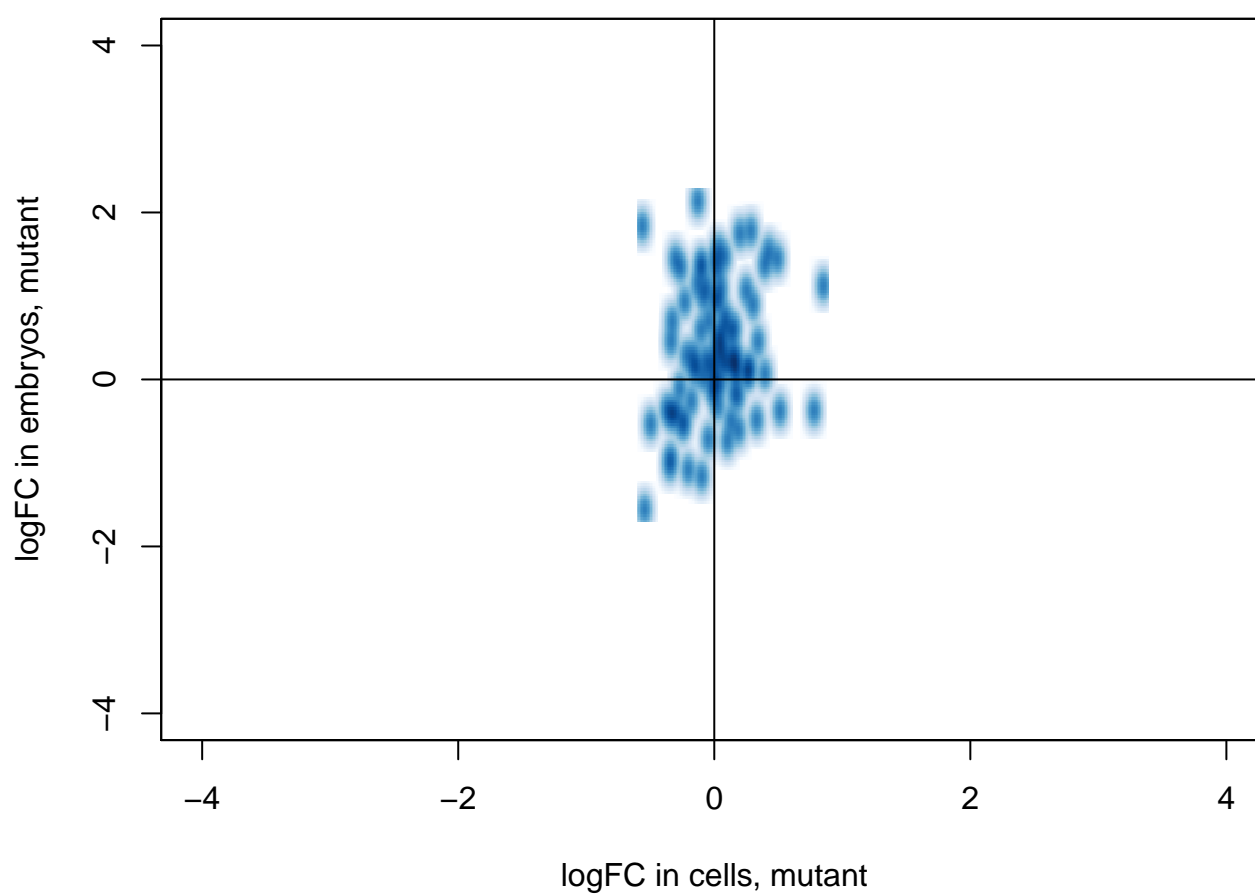

Supplement: File S1 — Archive (gzip2 compressed tar ball format) with 82 PDF files showing scatter plots for GO biological process terms with more than 50 annotated genes. Legend analogous to Figure 2. (BZ2) [file pone.0028985.s013.bz2 › scatterplots for 82 GO process terms with more than 50 genes/border follicle cell migration-results.pdf]

### genes in carbohydrate metabolic process

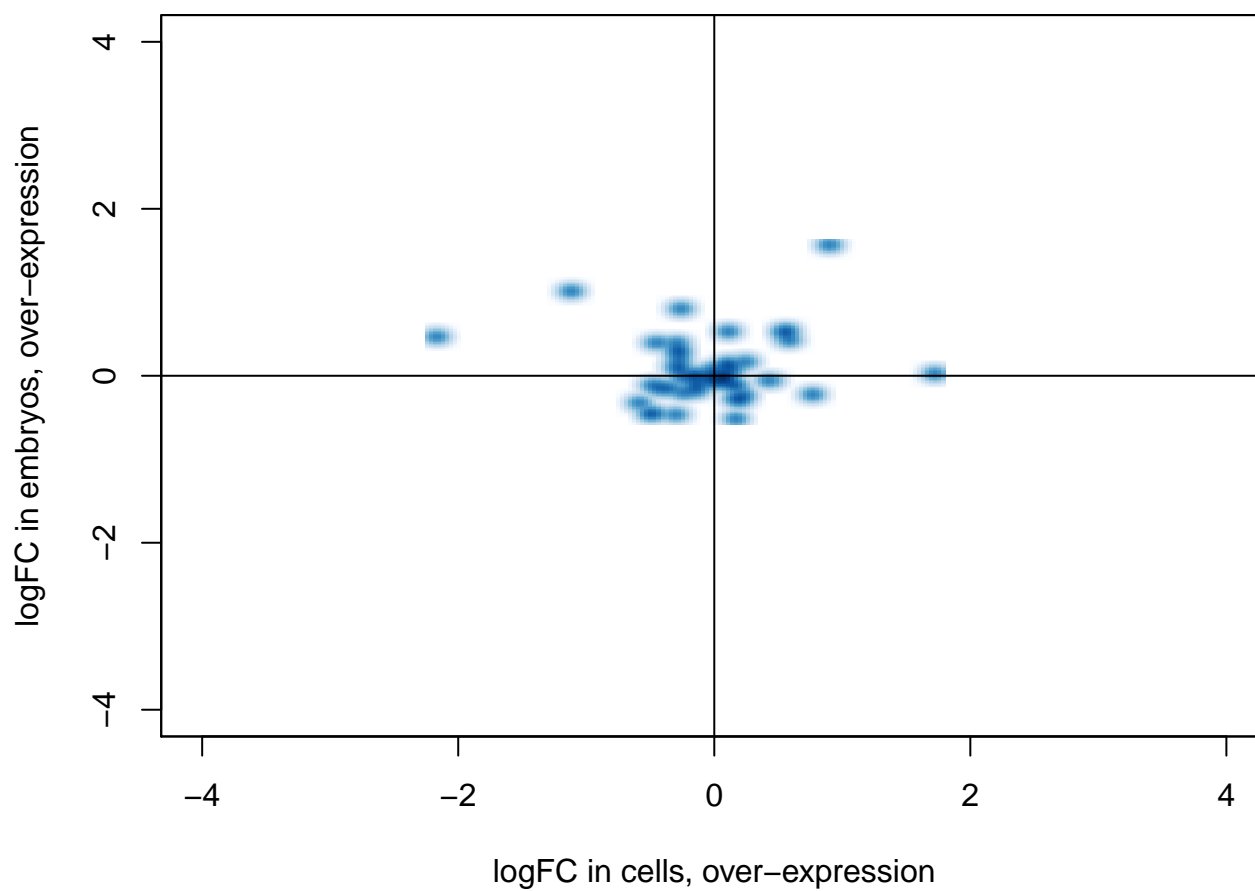

### genes in carbohydrate metabolic process

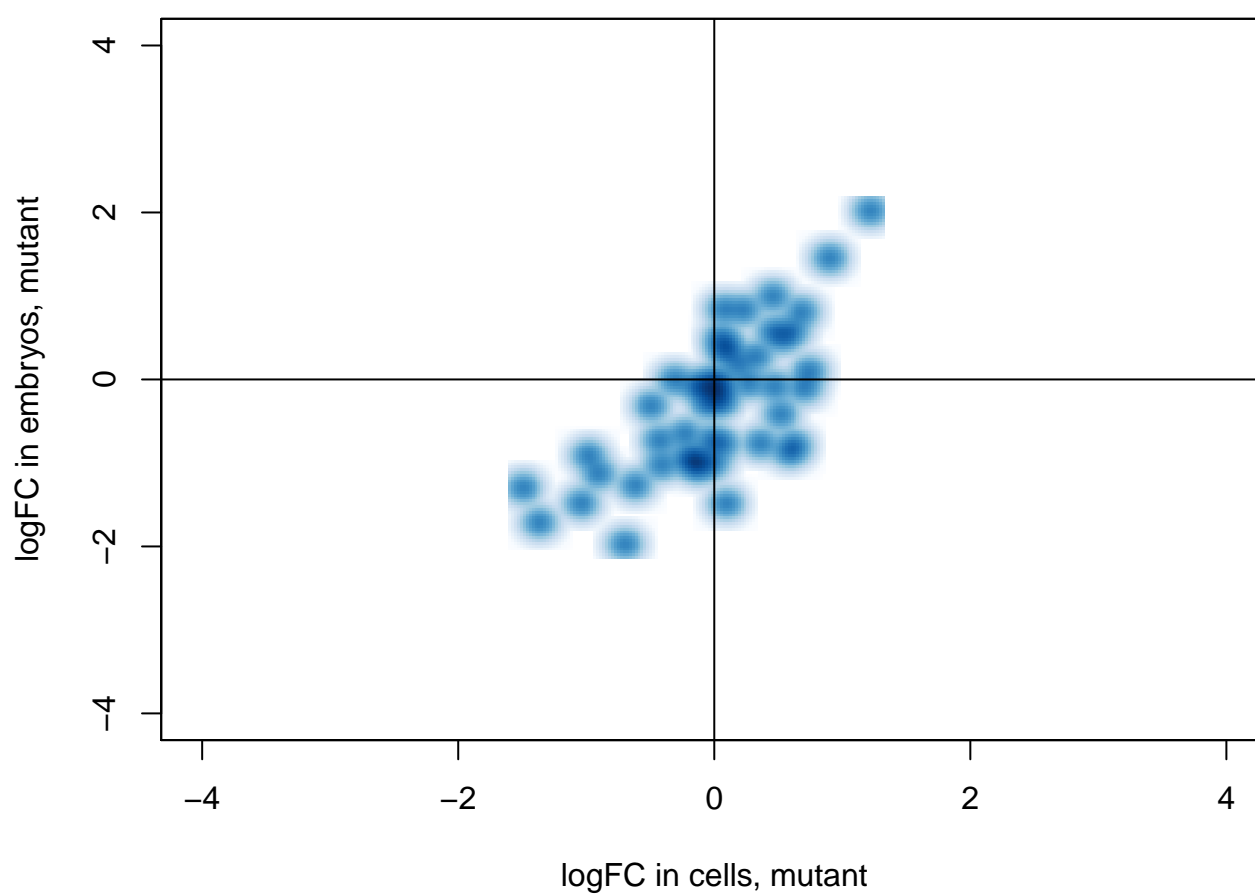

Supplement: File S1 — Archive (gzip2 compressed tar ball format) with 82 PDF files showing scatter plots for GO biological process terms with more than 50 annotated genes. Legend analogous to Figure 2. (BZ2) [file pone.0028985.s013.bz2 › scatterplots for 82 GO process terms with more than 50 genes/carbohydrate metabolic process-results.pdf]

### genes in cell adhesion

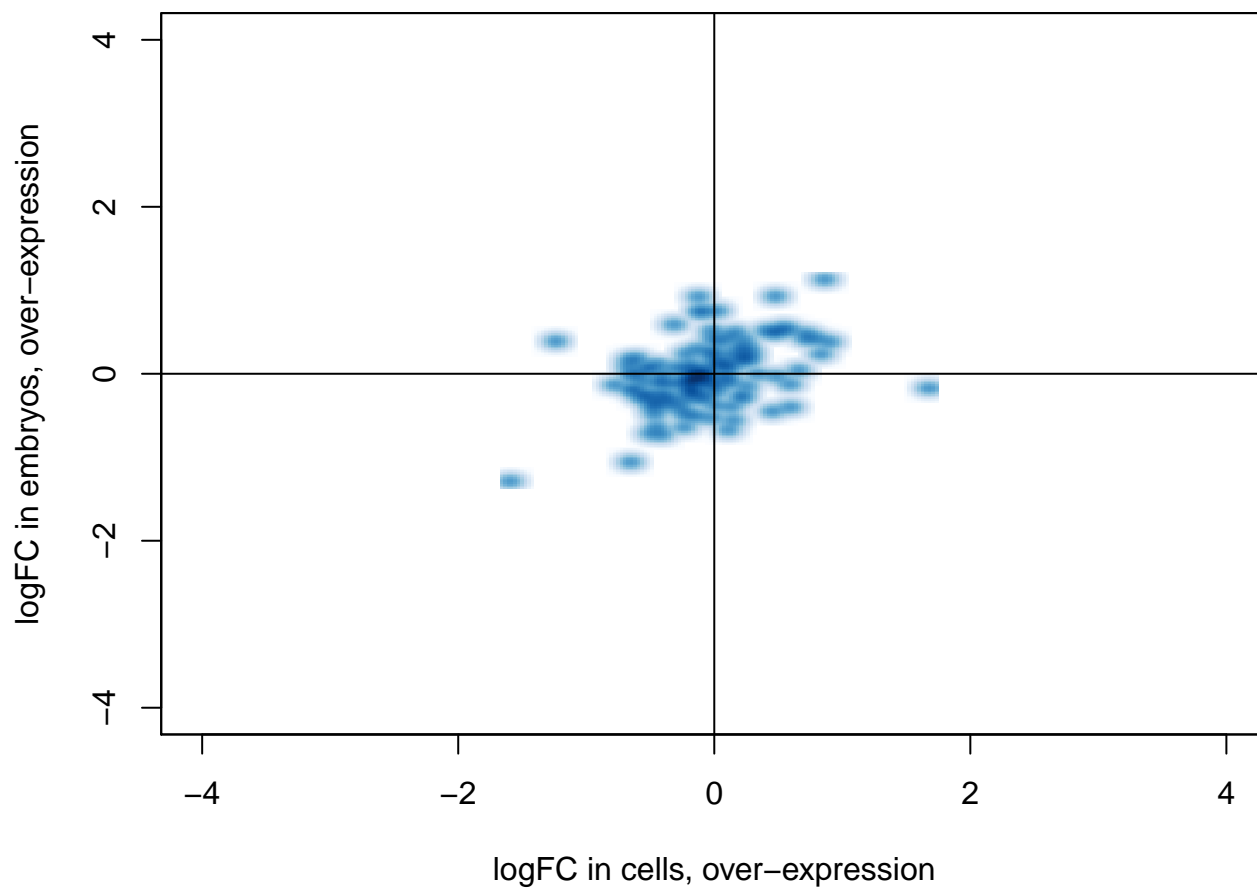

### genes in cell adhesion

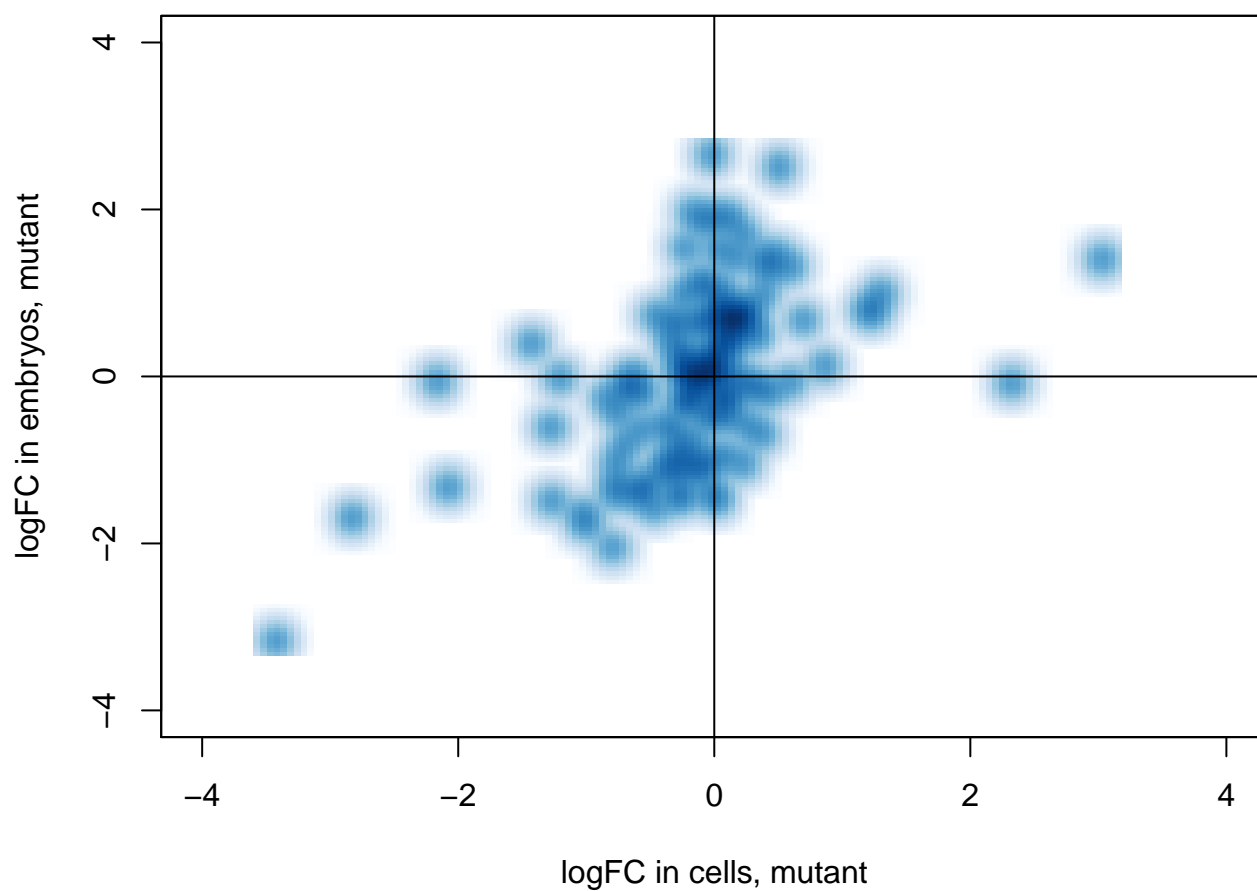

Supplement: File S1 — Archive (gzip2 compressed tar ball format) with 82 PDF files showing scatter plots for GO biological process terms with more than 50 annotated genes. Legend analogous to Figure 2. (BZ2) [file pone.0028985.s013.bz2 › scatterplots for 82 GO process terms with more than 50 genes/cell adhesion-results.pdf]

### genes in cell proliferation

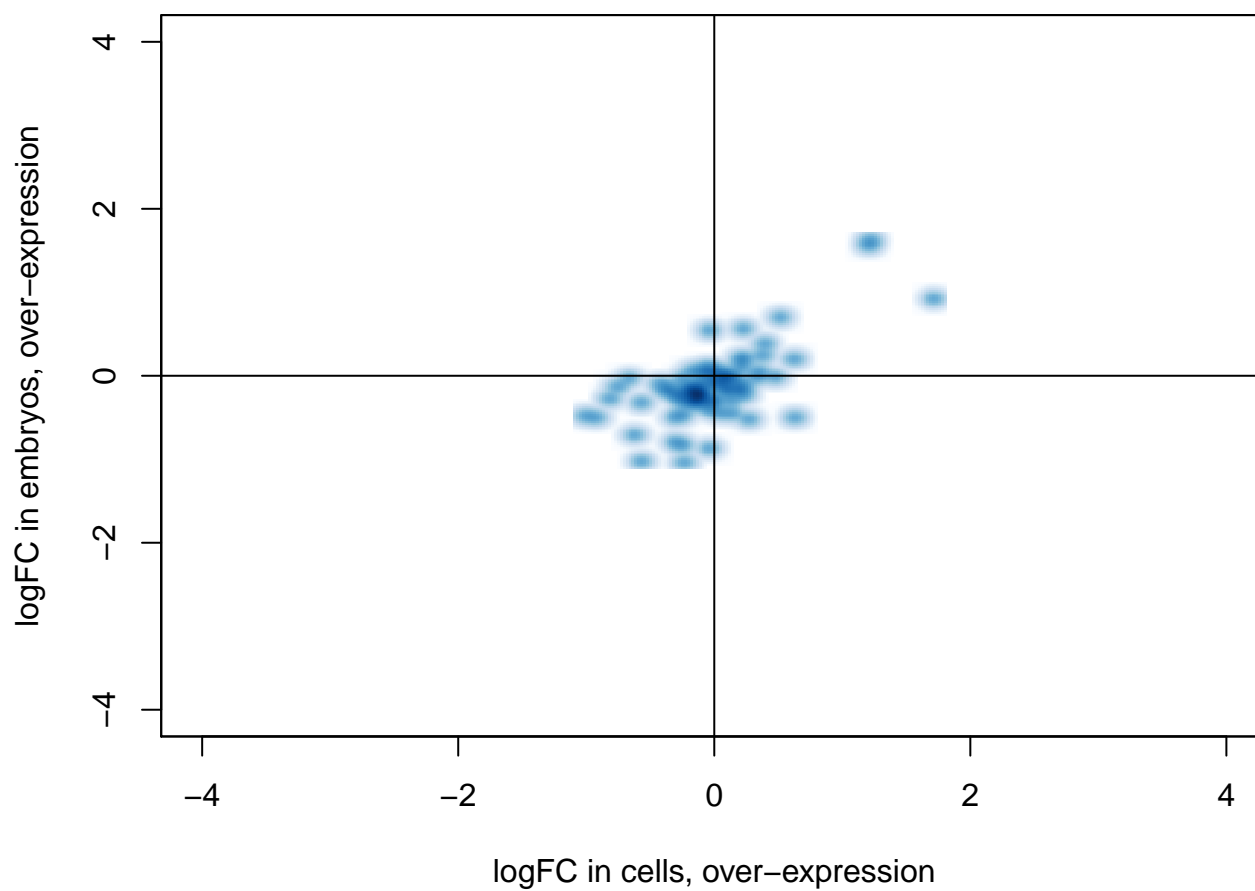

### genes in cell proliferation

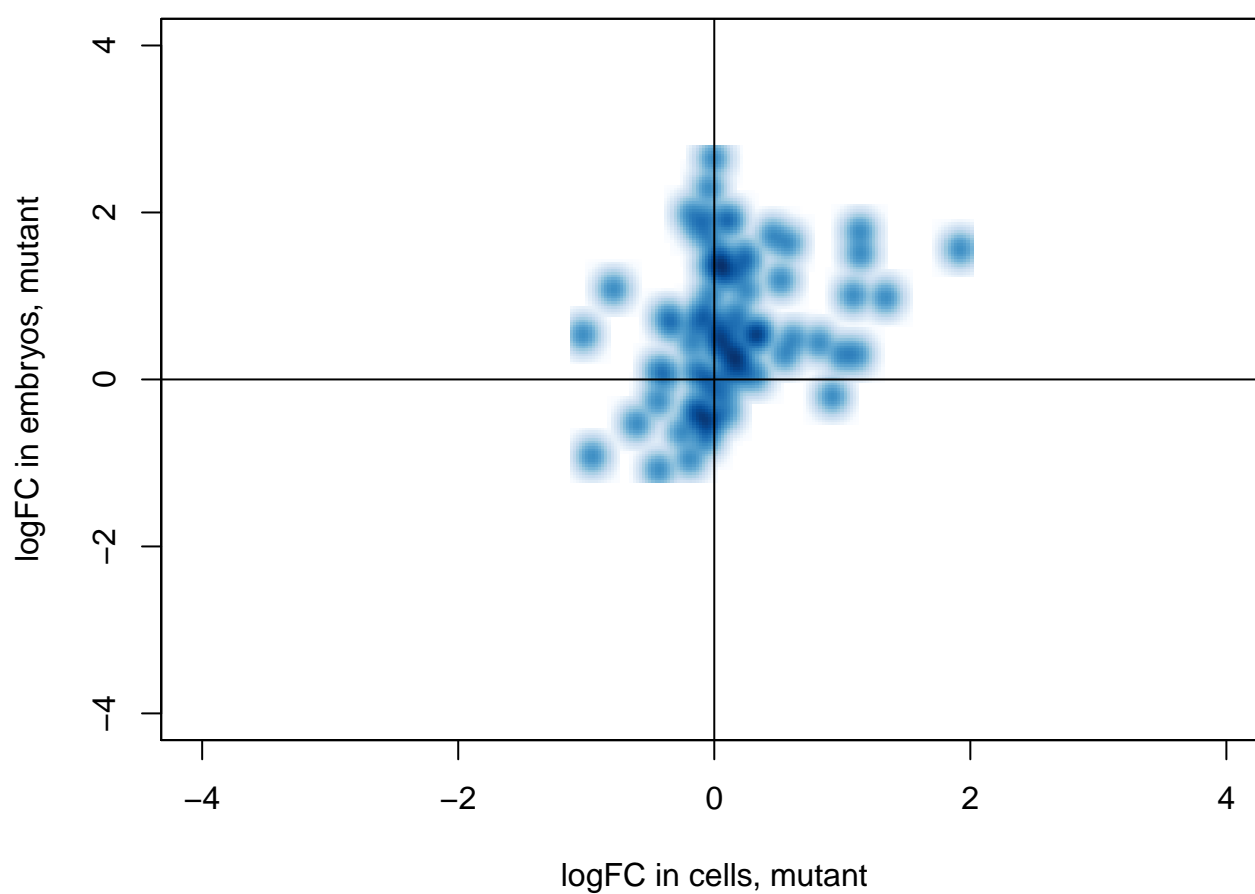

Supplement: File S1 — Archive (gzip2 compressed tar ball format) with 82 PDF files showing scatter plots for GO biological process terms with more than 50 annotated genes. Legend analogous to Figure 2. (BZ2) [file pone.0028985.s013.bz2 › scatterplots for 82 GO process terms with more than 50 genes/cell proliferation-results.pdf]

### genes in cell redox homeostasis

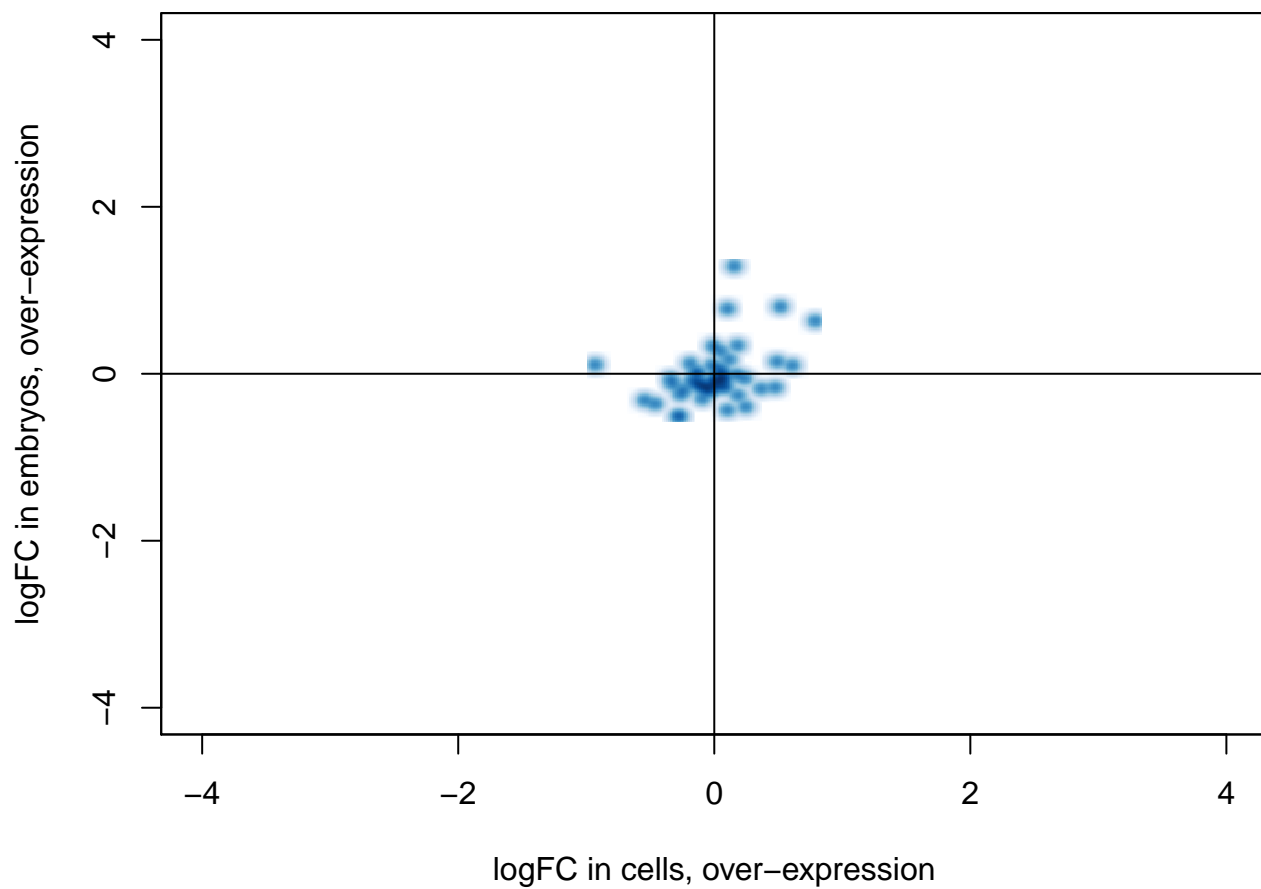

### genes in cell redox homeostasis

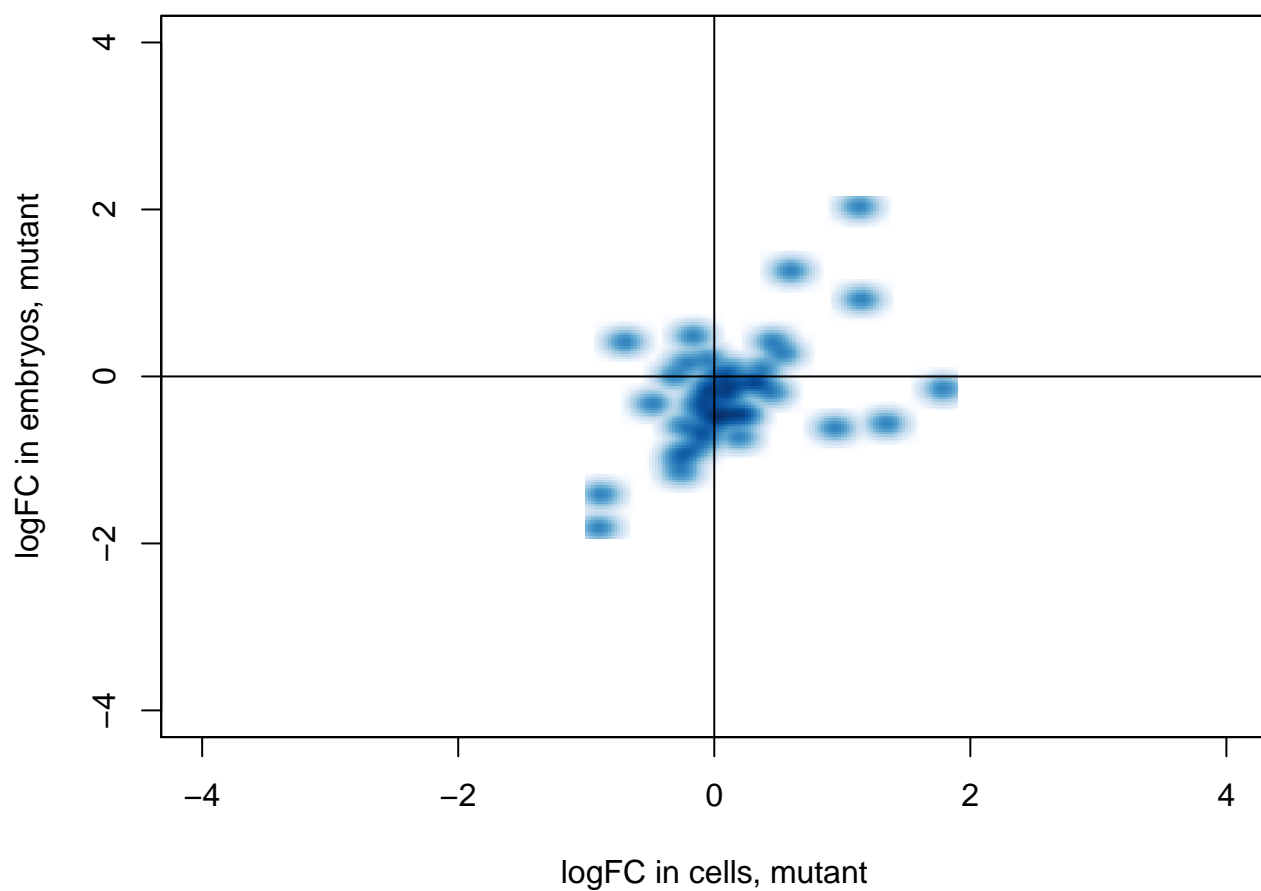

Supplement: File S1 — Archive (gzip2 compressed tar ball format) with 82 PDF files showing scatter plots for GO biological process terms with more than 50 annotated genes. Legend analogous to Figure 2. (BZ2) [file pone.0028985.s013.bz2 › scatterplots for 82 GO process terms with more than 50 genes/cell redox homeostasis-results.pdf]

### genes in cellular process

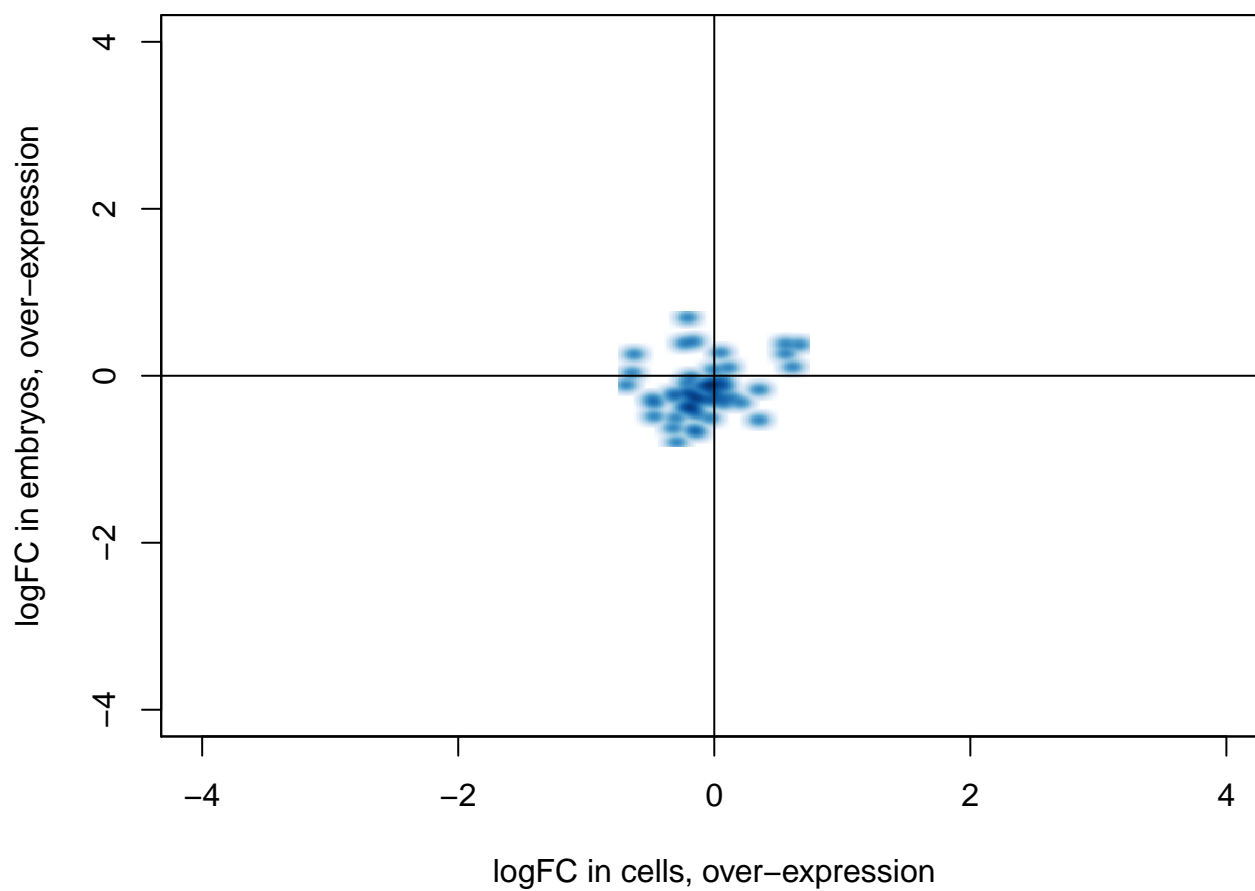

### genes in cellular process

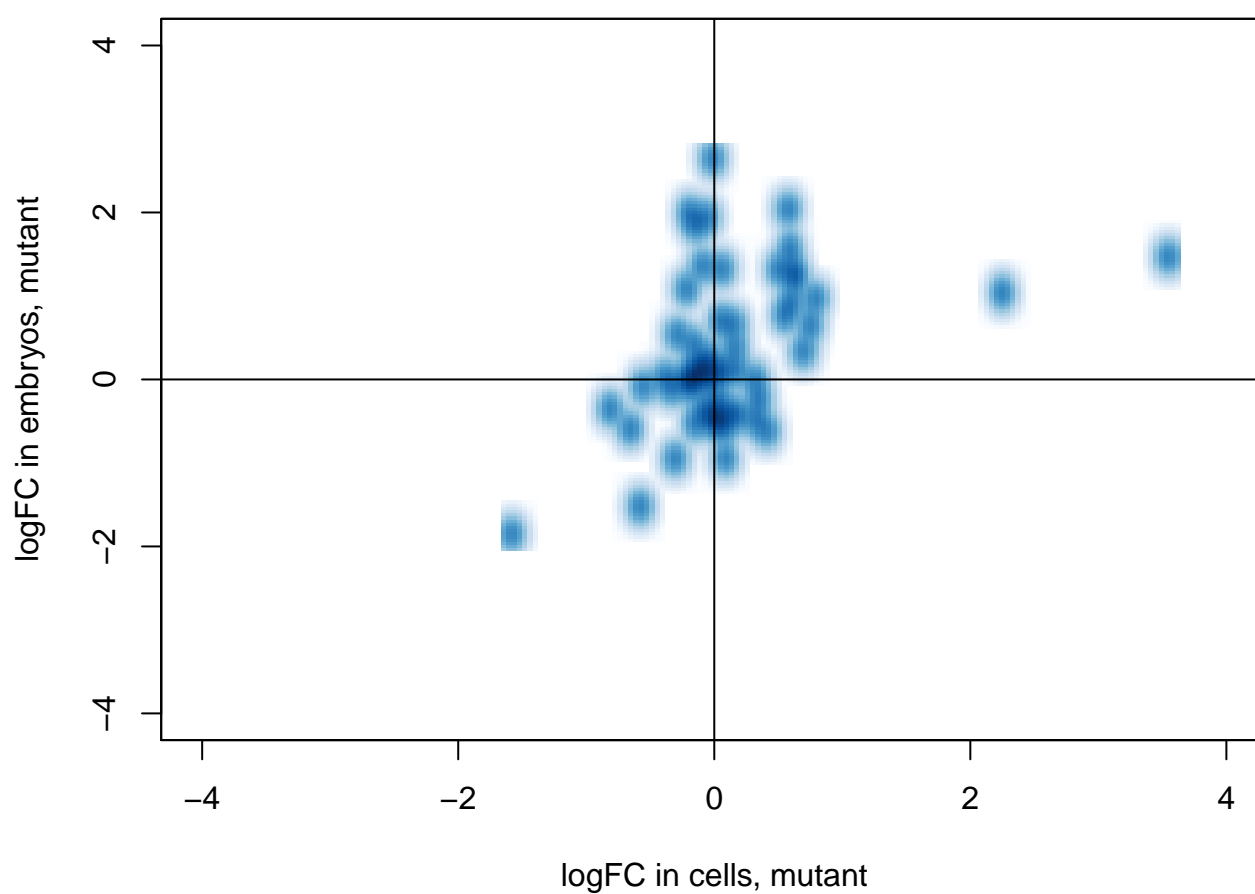

Supplement: File S1 — Archive (gzip2 compressed tar ball format) with 82 PDF files showing scatter plots for GO biological process terms with more than 50 annotated genes. Legend analogous to Figure 2. (BZ2) [file pone.0028985.s013.bz2 › scatterplots for 82 GO process terms with more than 50 genes/cellular process-results.pdf]

### genes in central nervous system development

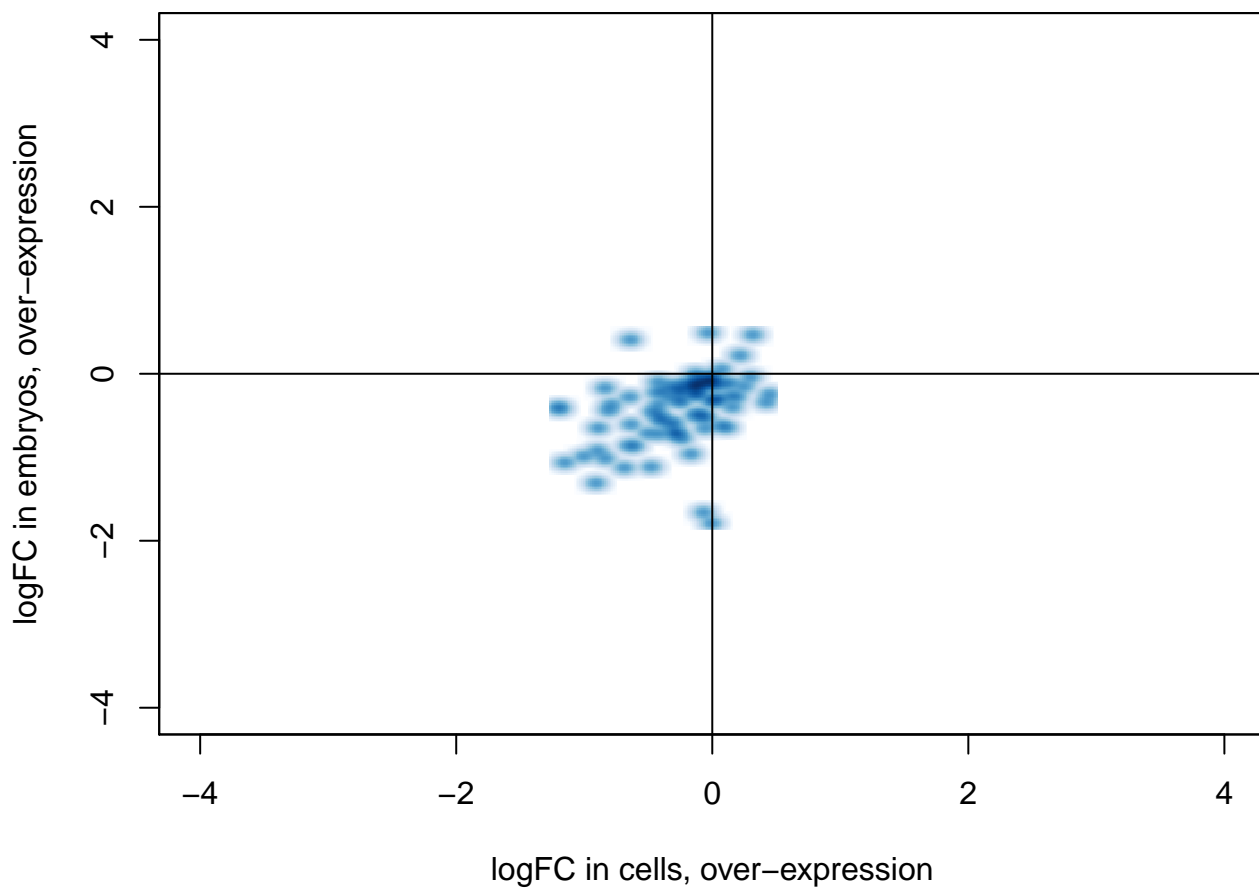

### genes in central nervous system development

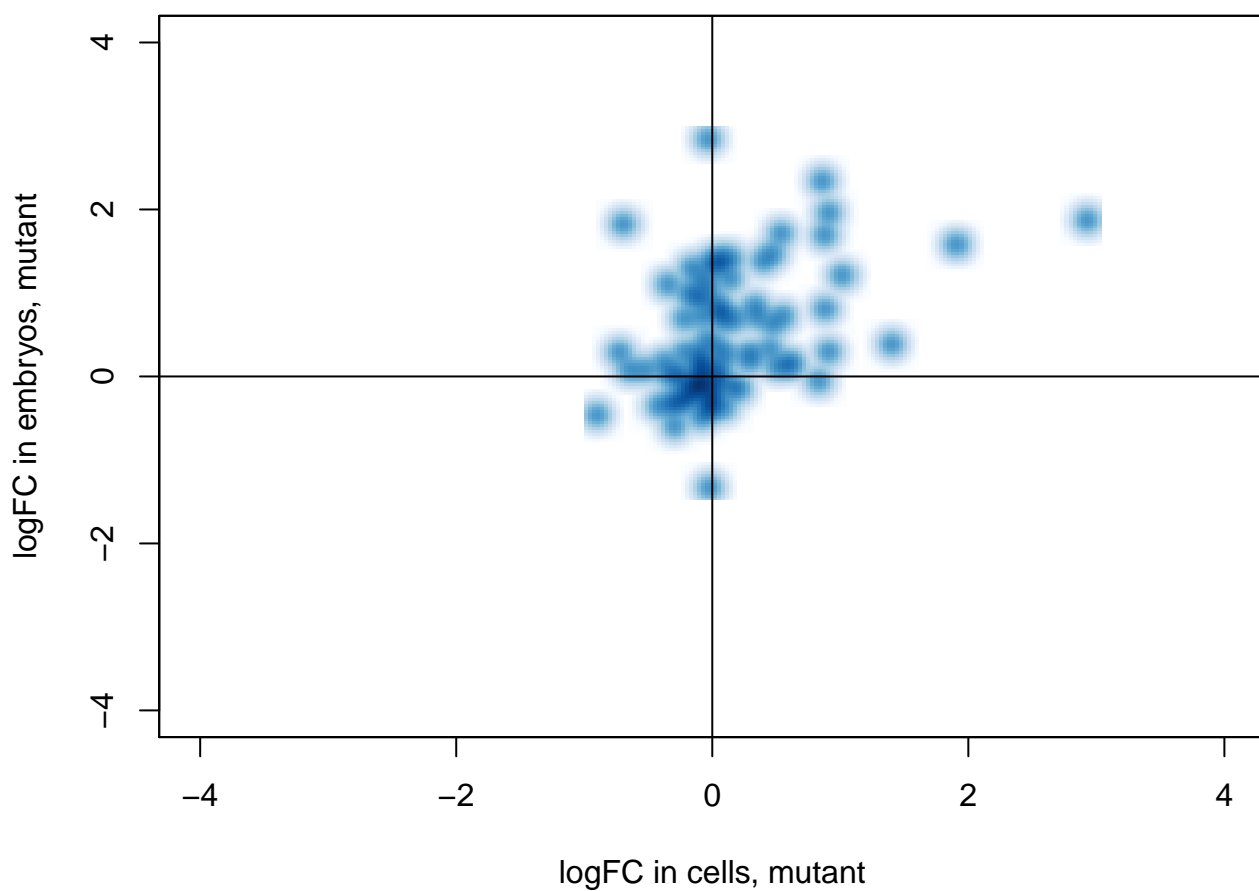

Supplement: File S1 — Archive (gzip2 compressed tar ball format) with 82 PDF files showing scatter plots for GO biological process terms with more than 50 annotated genes. Legend analogous to Figure 2. (BZ2) [file pone.0028985.s013.bz2 › scatterplots for 82 GO process terms with more than 50 genes/central nervous system development-results.pdf]

### genes in chitin metabolic process

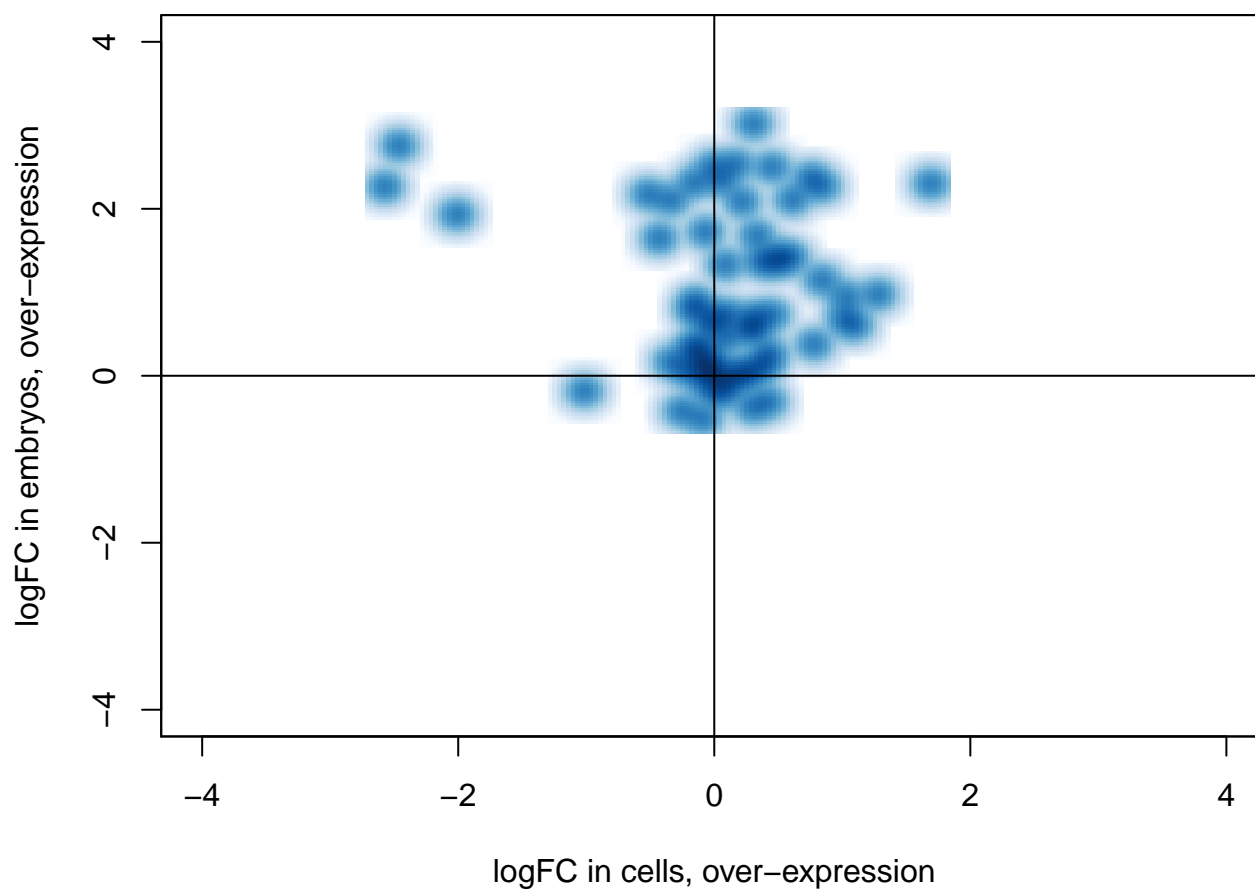

### genes in chitin metabolic process

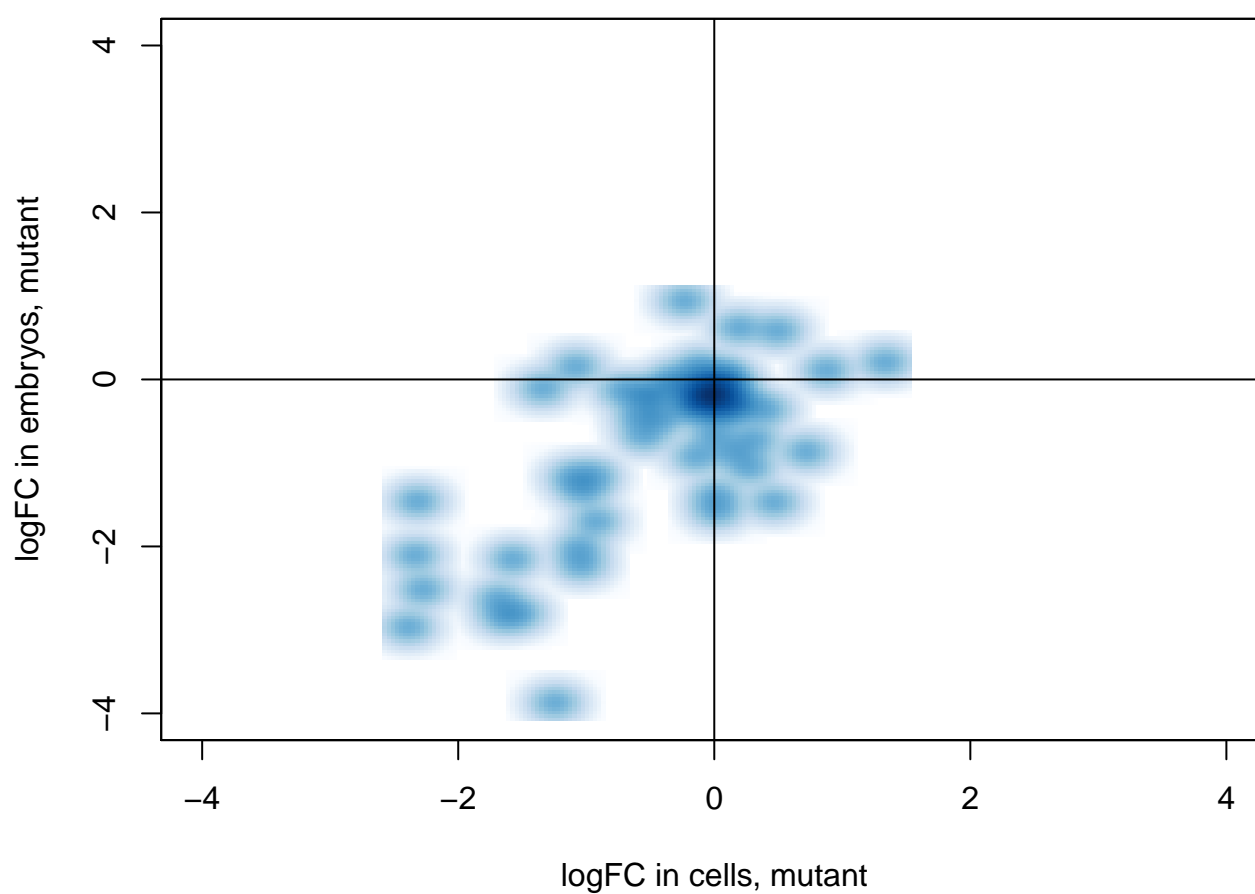

Supplement: File S1 — Archive (gzip2 compressed tar ball format) with 82 PDF files showing scatter plots for GO biological process terms with more than 50 annotated genes. Legend analogous to Figure 2. (BZ2) [file pone.0028985.s013.bz2 › scatterplots for 82 GO process terms with more than 50 genes/chitin metabolic process-results.pdf]

### genes in chromatin assembly or disassembly

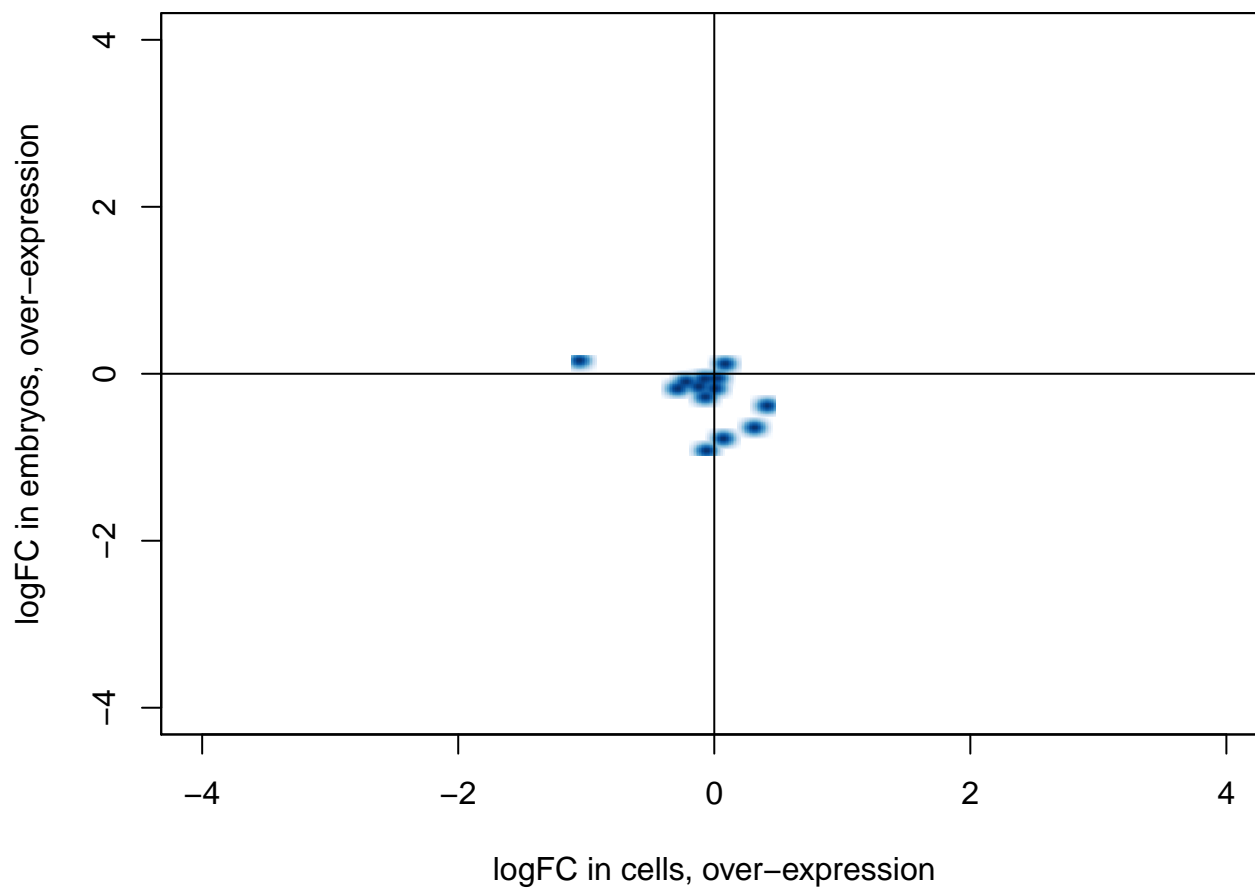

### genes in chromatin assembly or disassembly

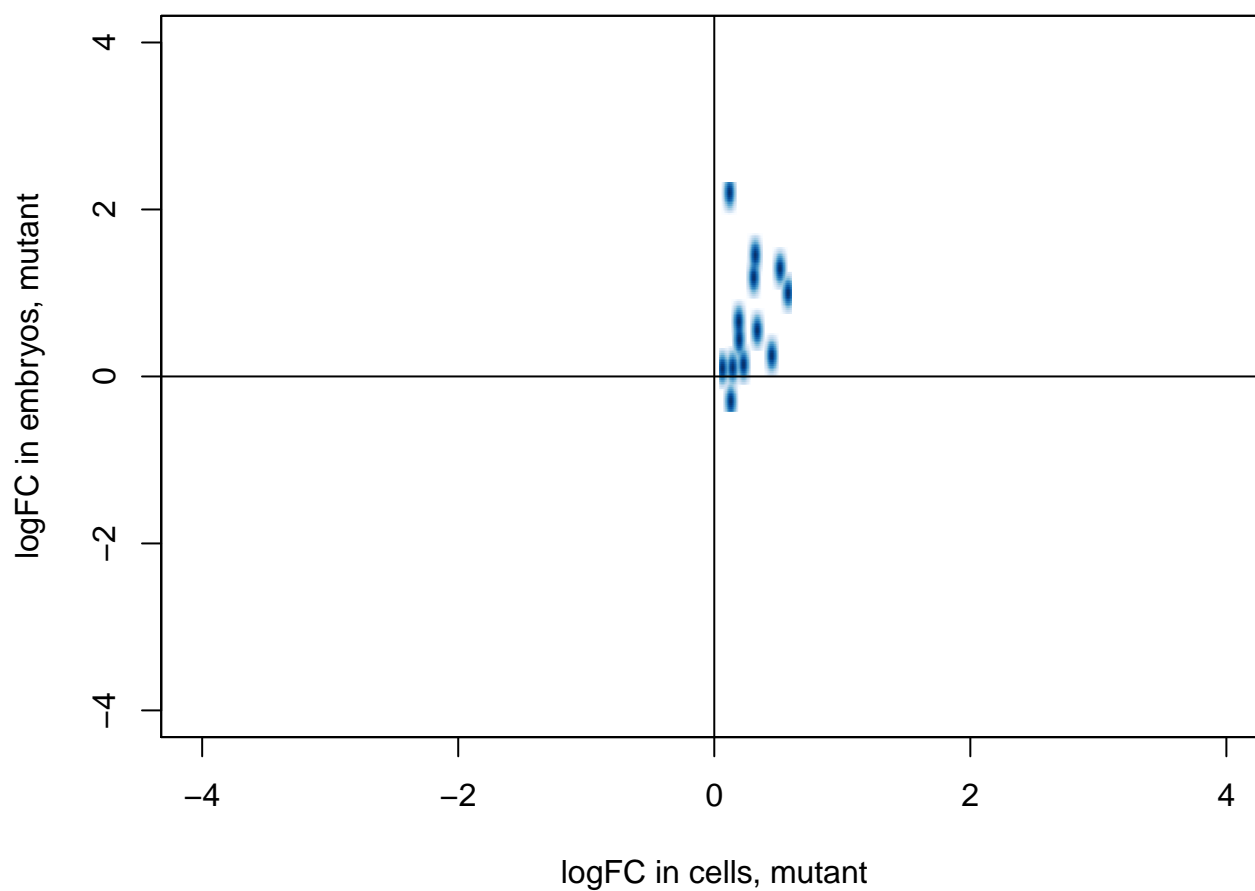

Supplement: File S1 — Archive (gzip2 compressed tar ball format) with 82 PDF files showing scatter plots for GO biological process terms with more than 50 annotated genes. Legend analogous to Figure 2. (BZ2) [file pone.0028985.s013.bz2 › scatterplots for 82 GO process terms with more than 50 genes/chromatin assembly or disassembly-results.pdf]

### genes in compound eye development

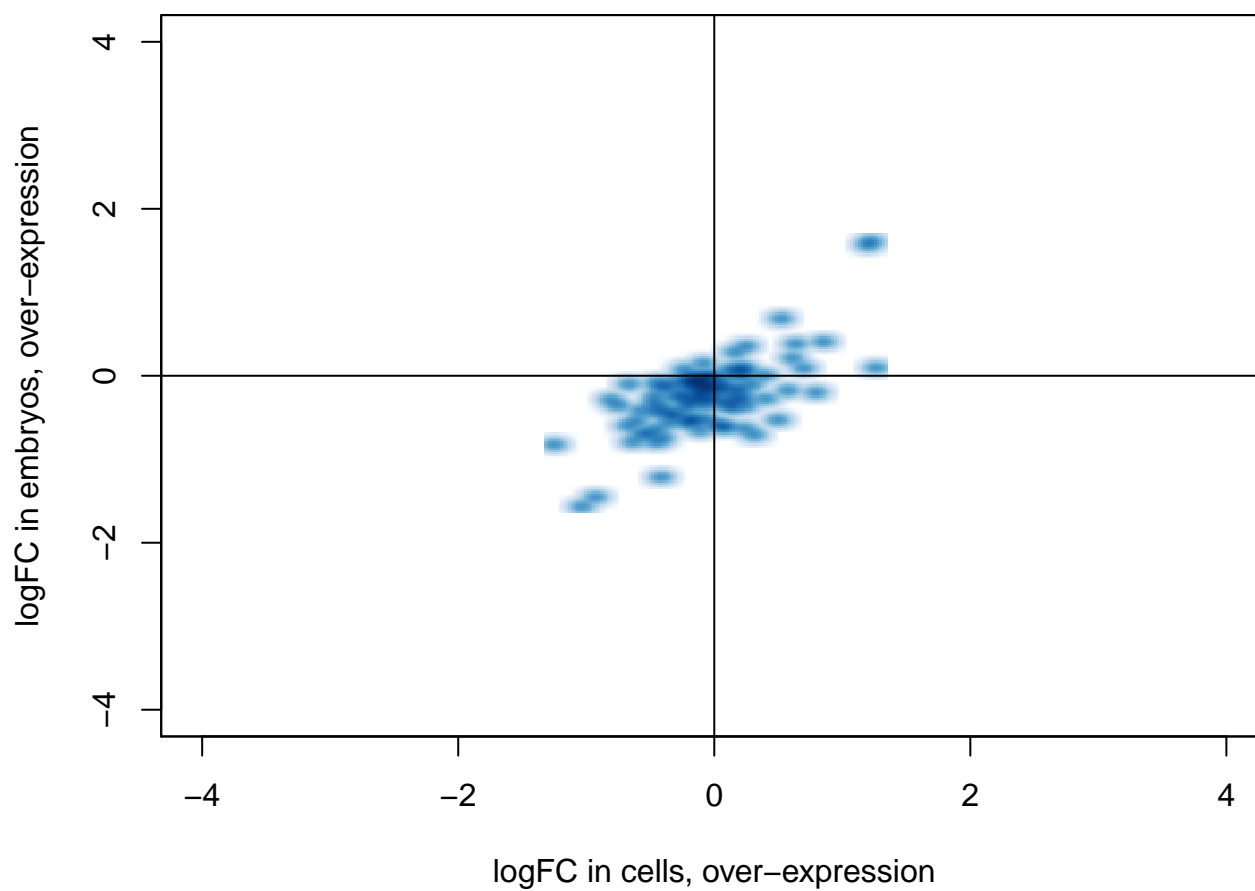

### genes in compound eye development

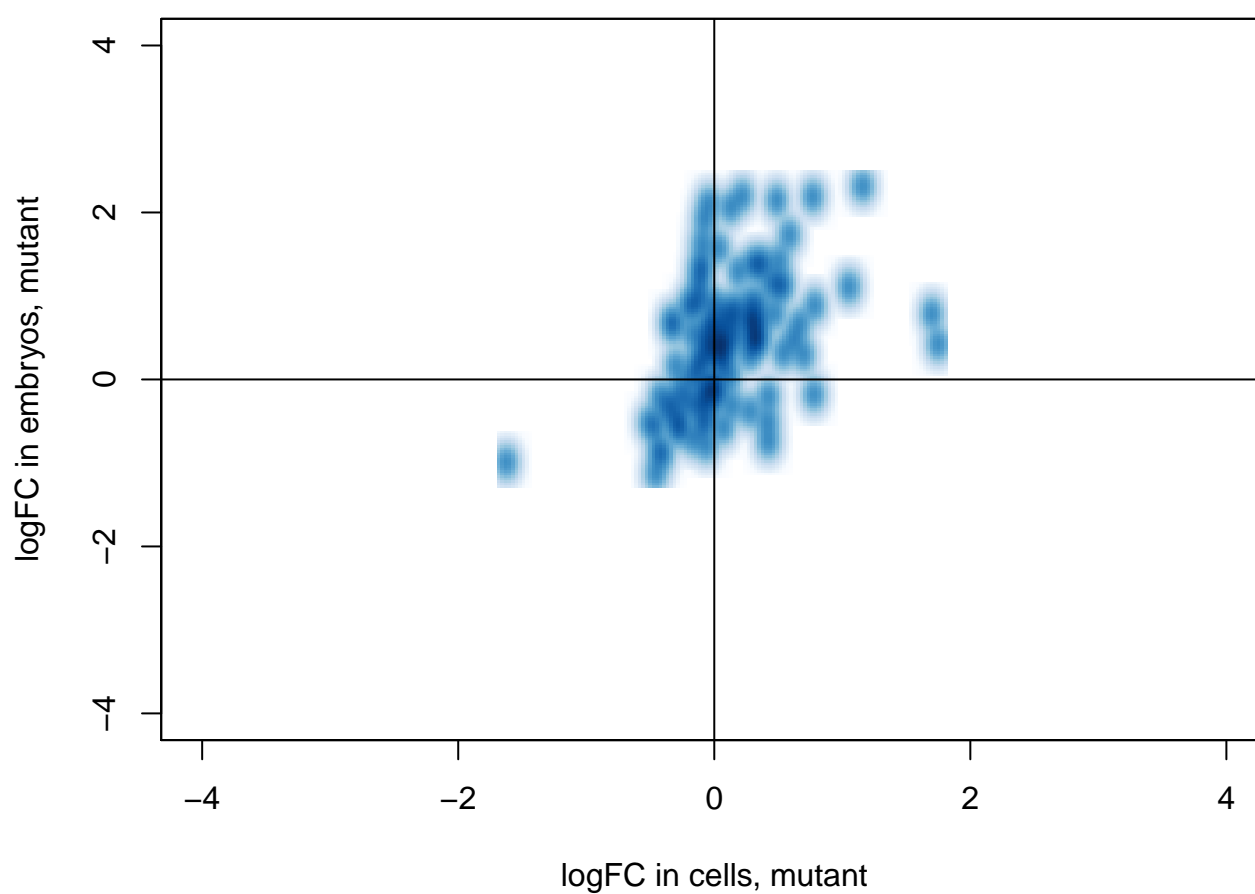

Supplement: File S1 — Archive (gzip2 compressed tar ball format) with 82 PDF files showing scatter plots for GO biological process terms with more than 50 annotated genes. Legend analogous to Figure 2. (BZ2) [file pone.0028985.s013.bz2 › scatterplots for 82 GO process terms with more than 50 genes/compound eye development-results.pdf]

### genes in compound eye morphogenesis

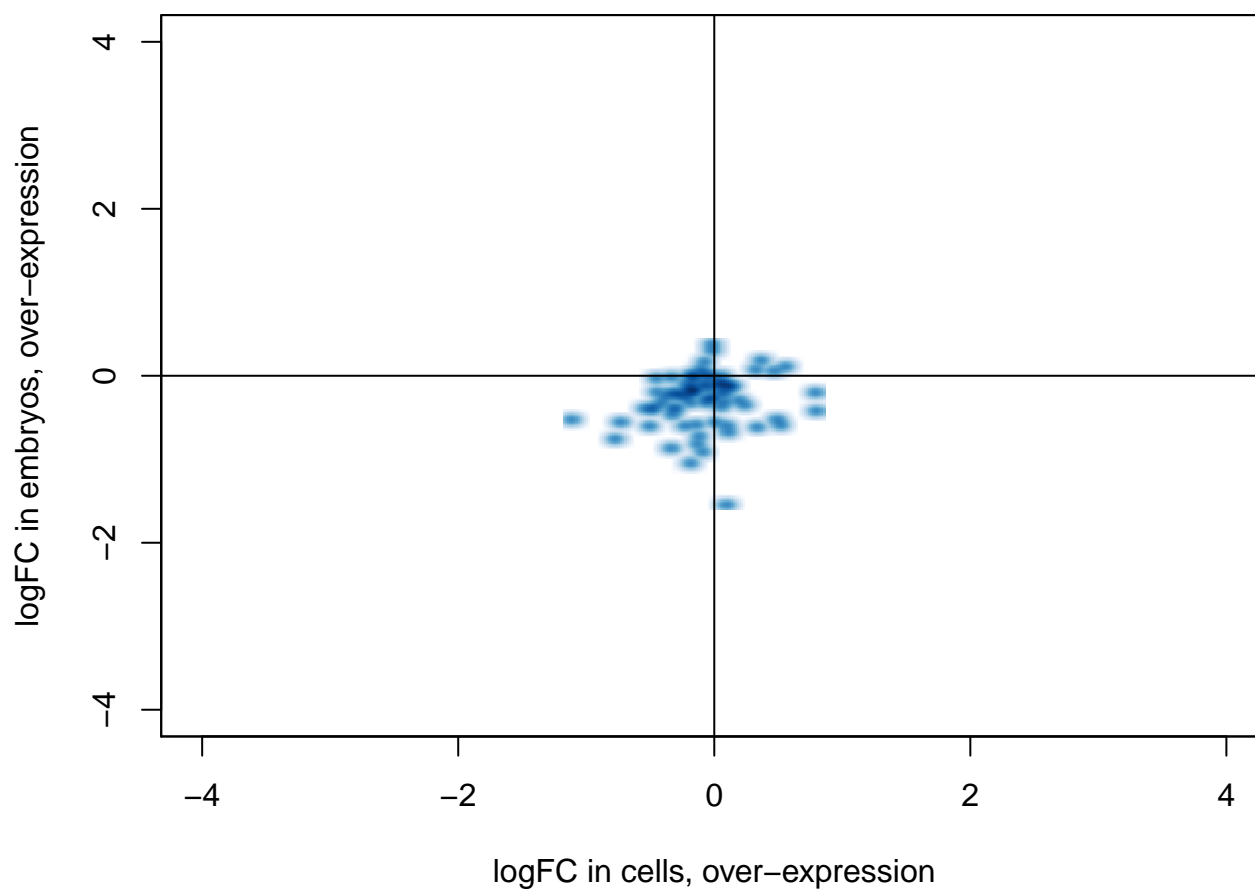

### genes in compound eye morphogenesis

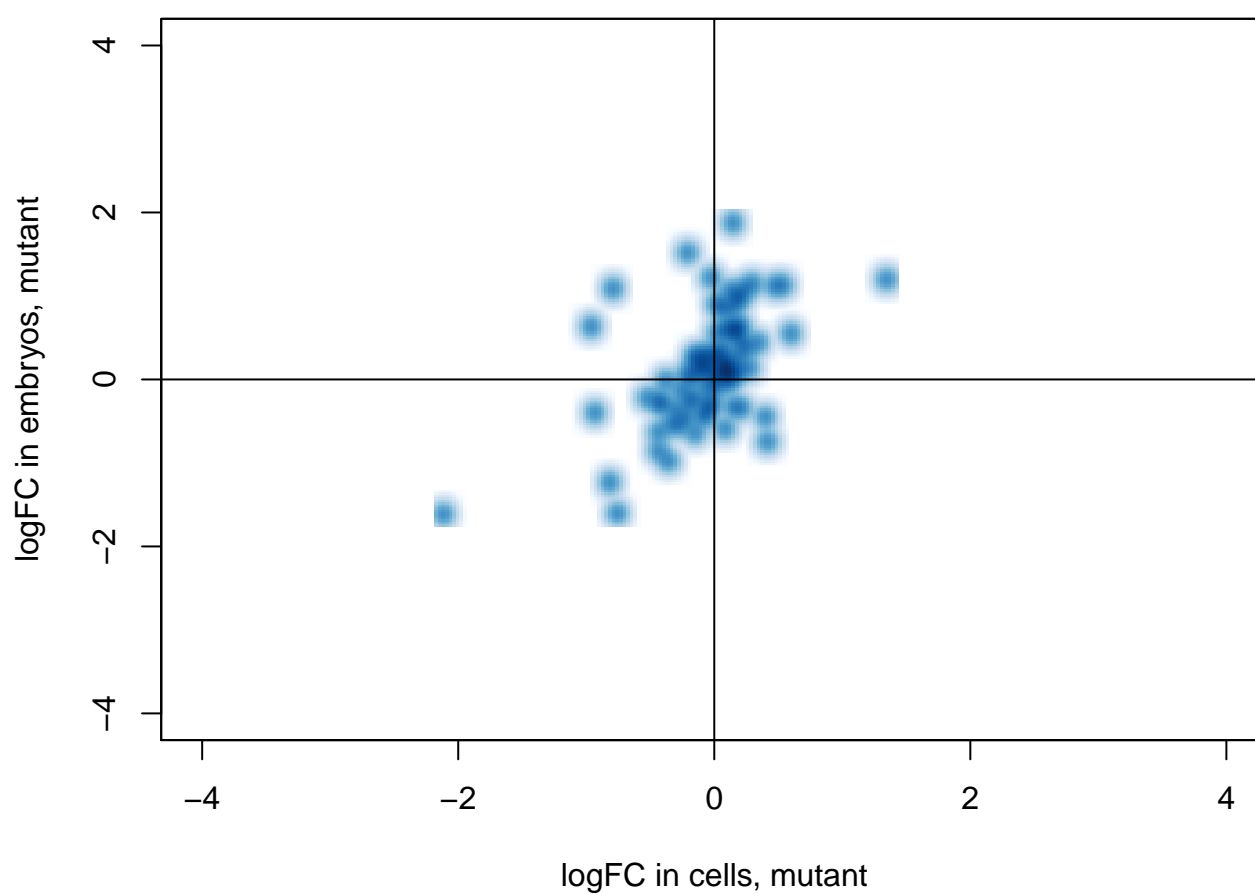

Supplement: File S1 — Archive (gzip2 compressed tar ball format) with 82 PDF files showing scatter plots for GO biological process terms with more than 50 annotated genes. Legend analogous to Figure 2. (BZ2) [file pone.0028985.s013.bz2 › scatterplots for 82 GO process terms with more than 50 genes/compound eye morphogenesis-results.pdf]

### genes in cytokinesis

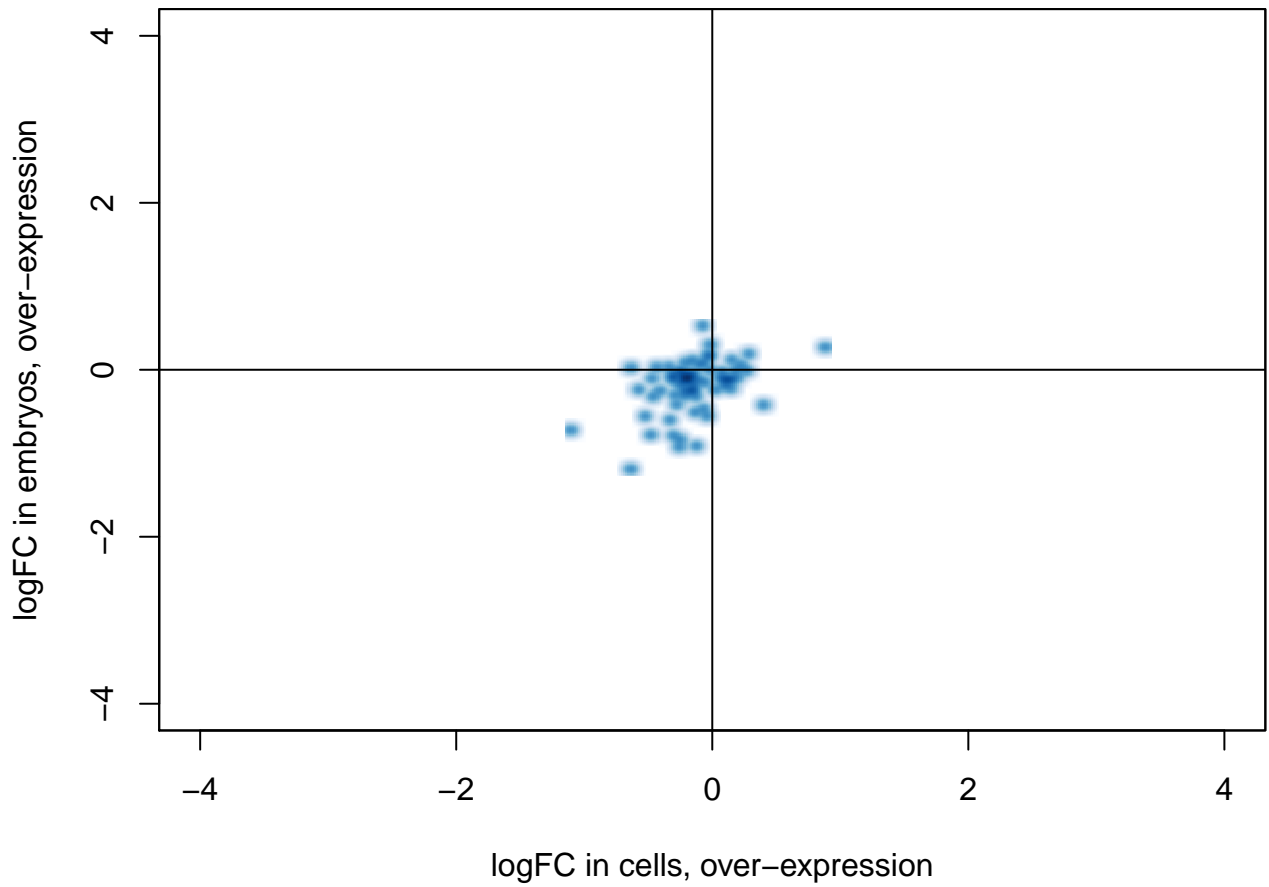

### genes in cytokinesis

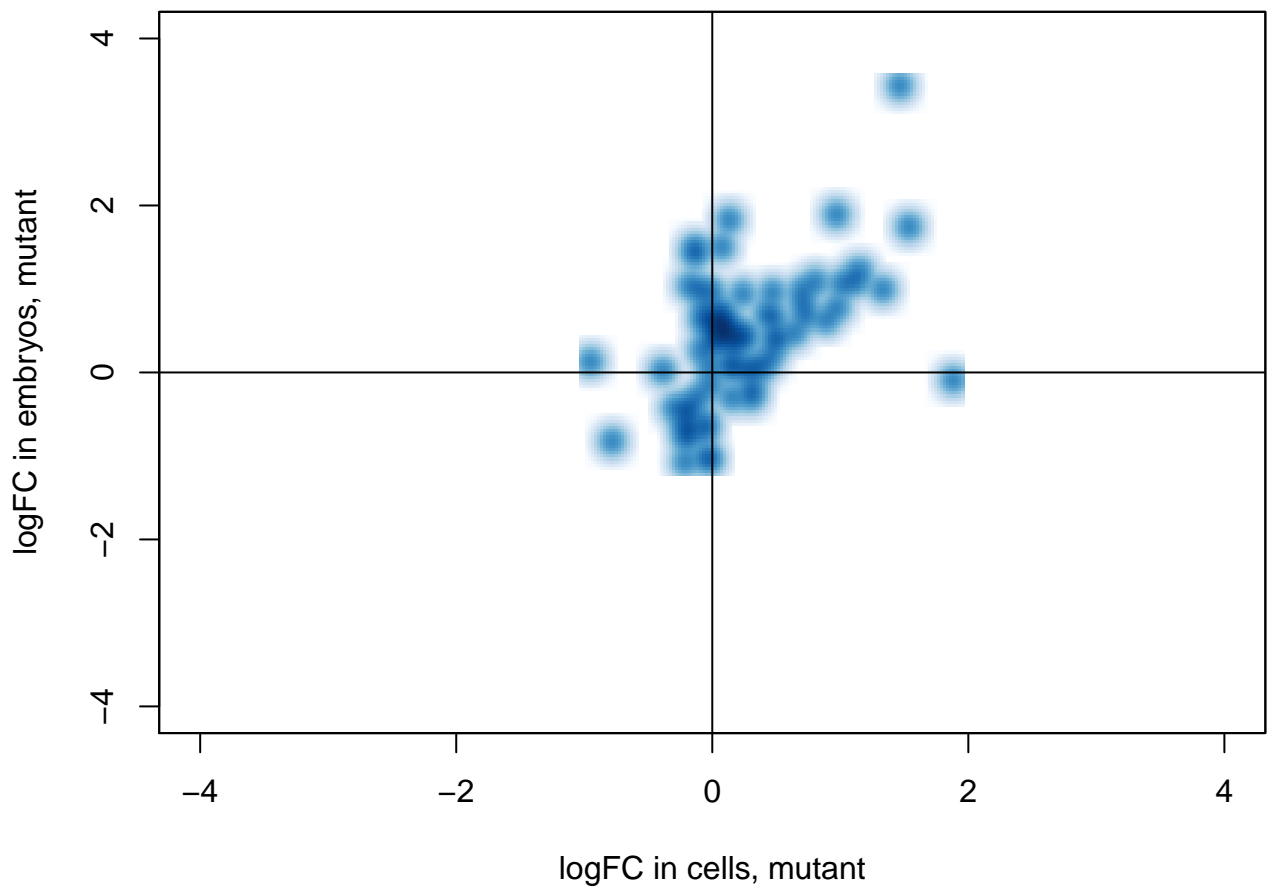

Supplement: File S1 — Archive (gzip2 compressed tar ball format) with 82 PDF files showing scatter plots for GO biological process terms with more than 50 annotated genes. Legend analogous to Figure 2. (BZ2) [file pone.0028985.s013.bz2 › scatterplots for 82 GO process terms with more than 50 genes/cytokinesis-results.pdf]

**genes in defense response**

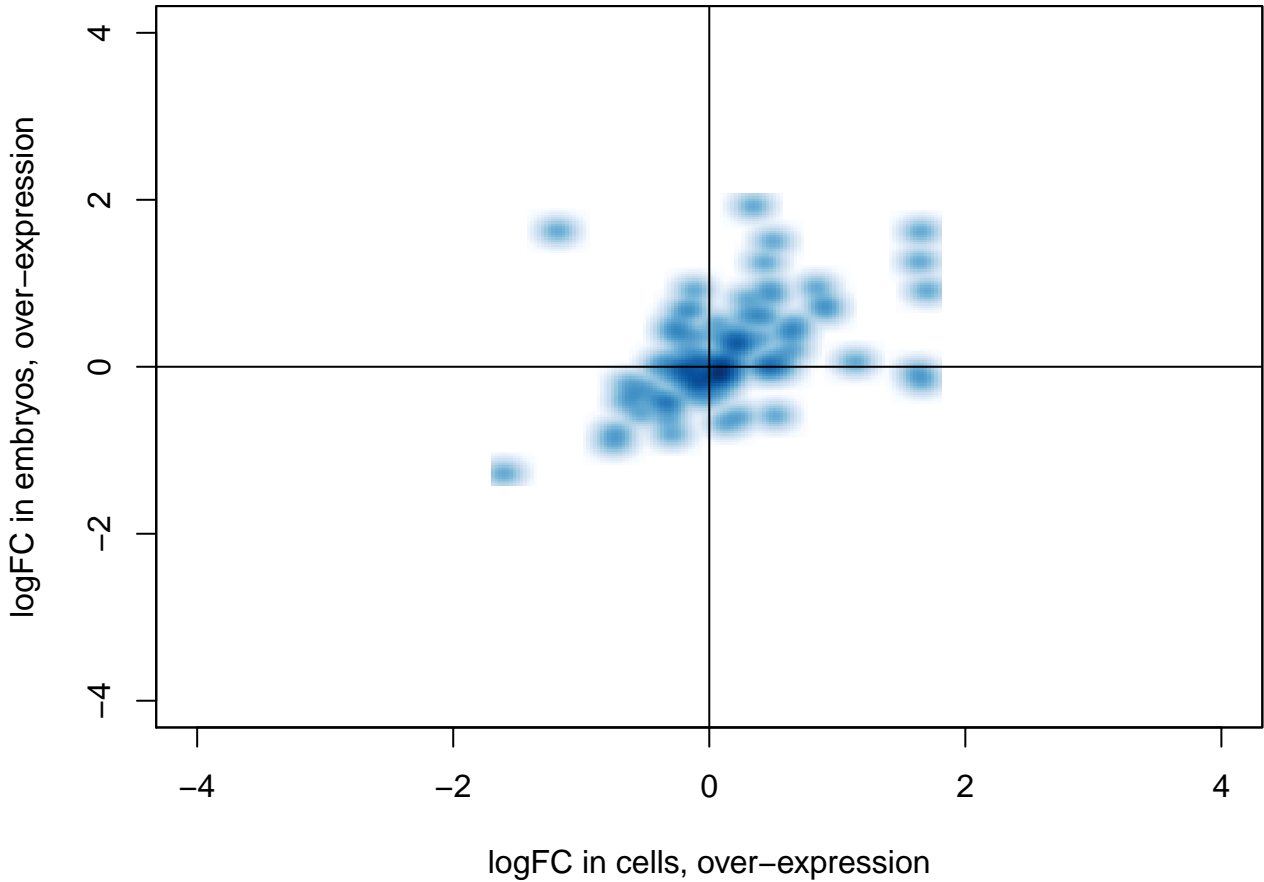

**genes in defense response**

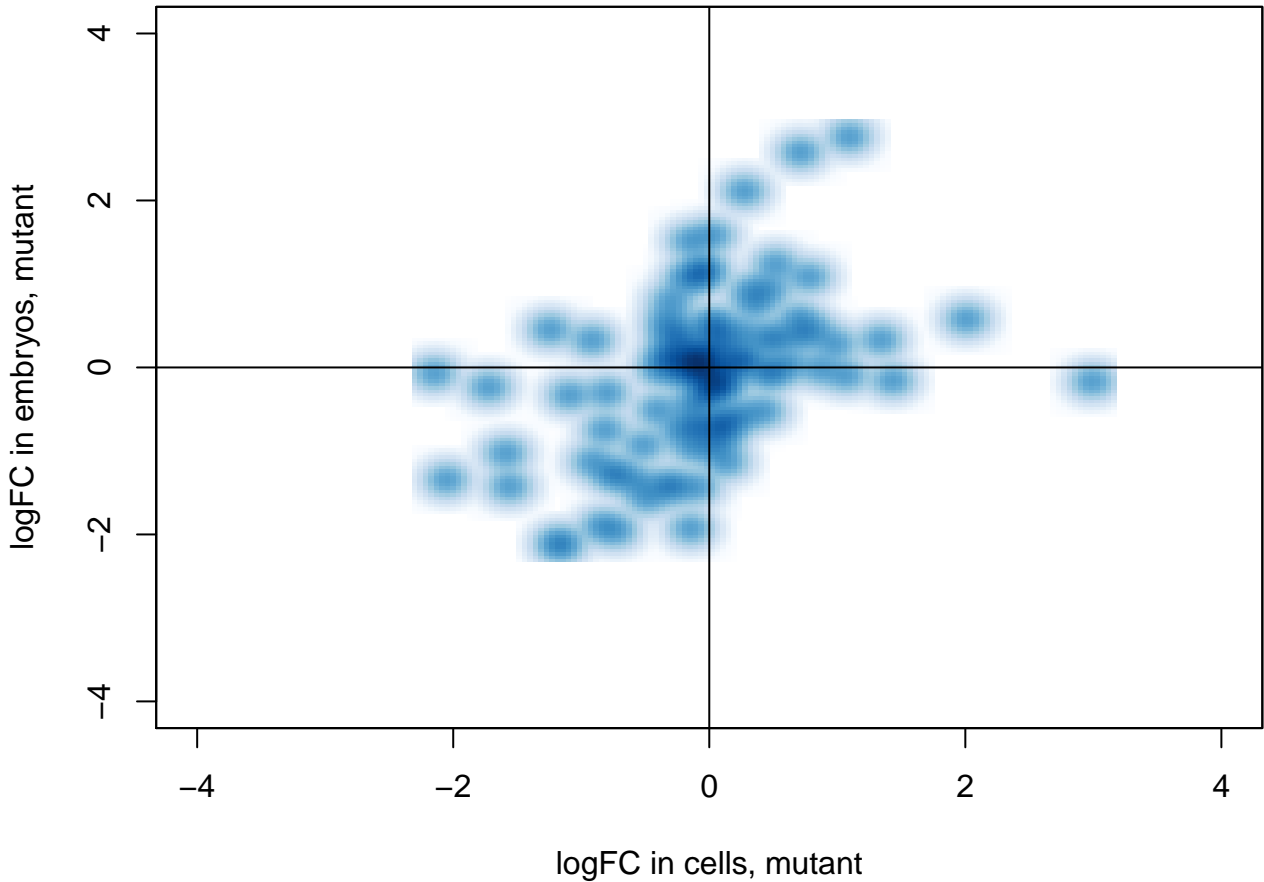

Supplement: File S1 — Archive (gzip2 compressed tar ball format) with 82 PDF files showing scatter plots for GO biological process terms with more than 50 annotated genes. Legend analogous to Figure 2. (BZ2) [file pone.0028985.s013.bz2 › scatterplots for 82 GO process terms with more than 50 genes/defense response-results.pdf]

### genes in dendrite morphogenesis

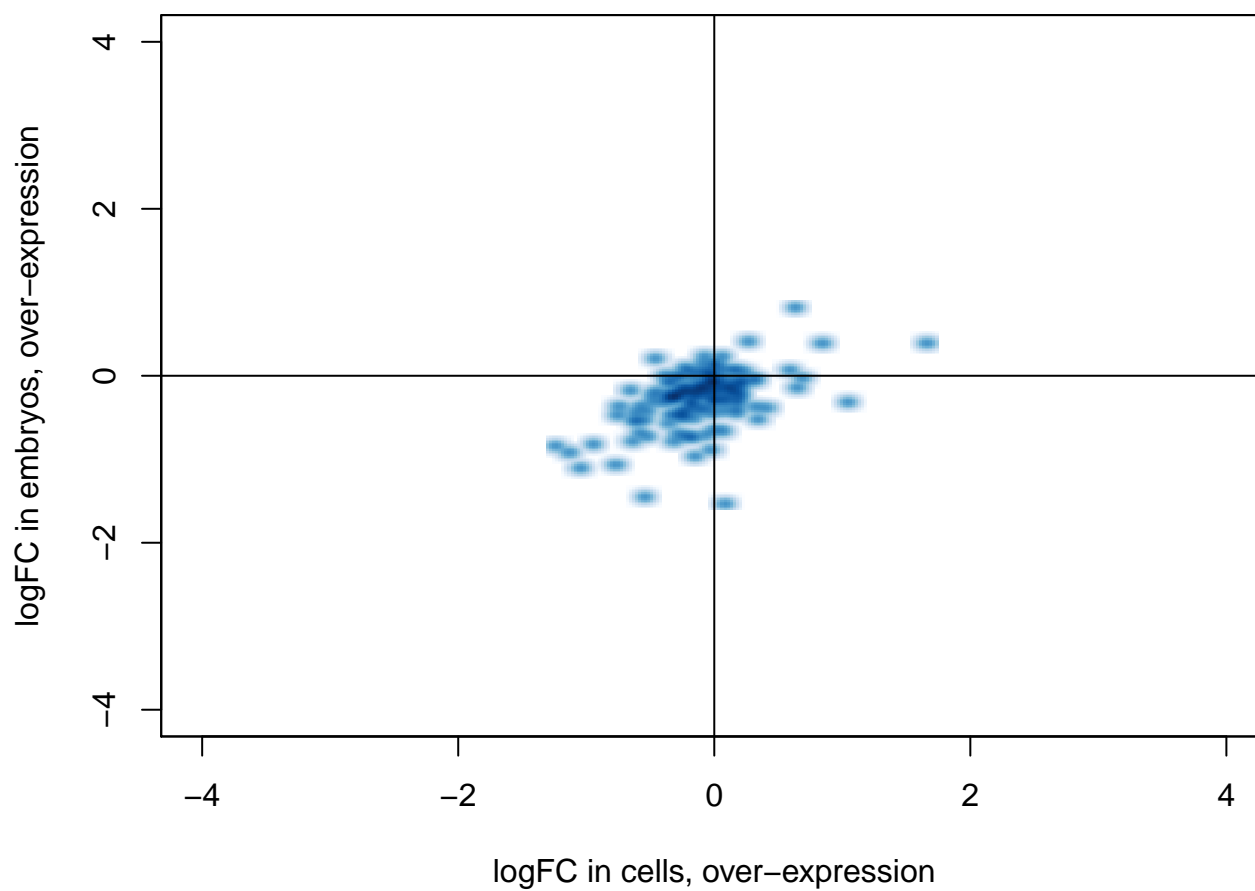

### genes in dendrite morphogenesis

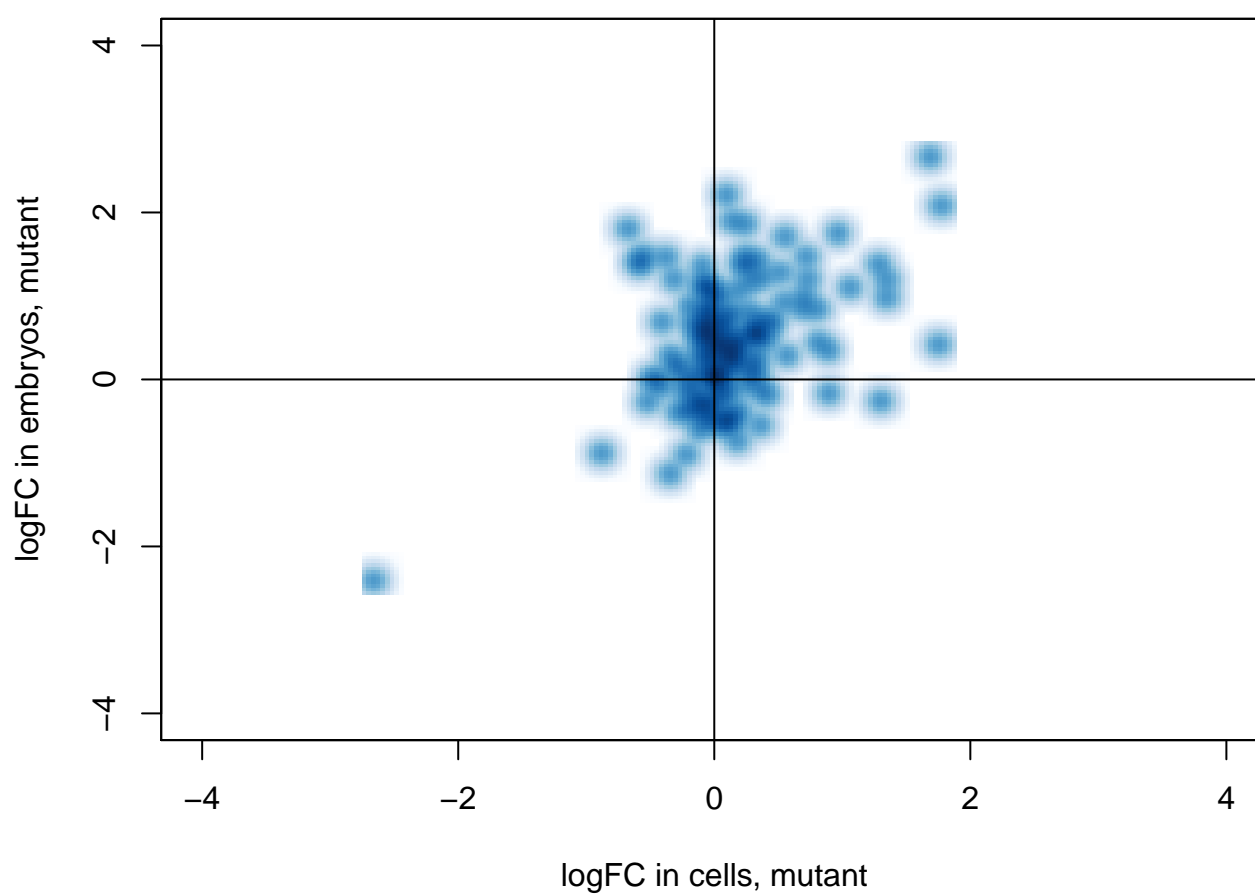

Supplement: File S1 — Archive (gzip2 compressed tar ball format) with 82 PDF files showing scatter plots for GO biological process terms with more than 50 annotated genes. Legend analogous to Figure 2. (BZ2) [file pone.0028985.s013.bz2 › scatterplots for 82 GO process terms with more than 50 genes/dendrite morphogenesis-results.pdf]

### genes in determination of adult lifespan

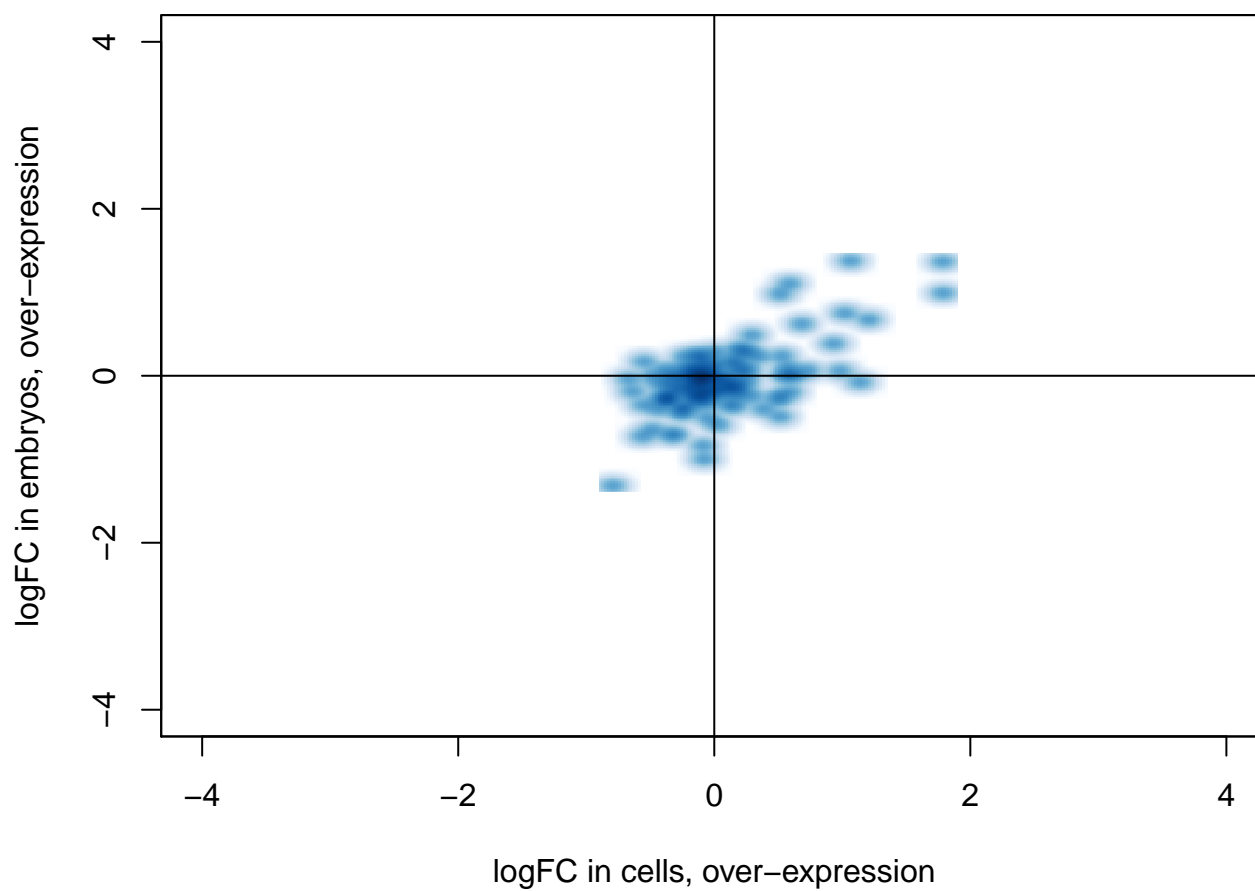

### genes in determination of adult lifespan

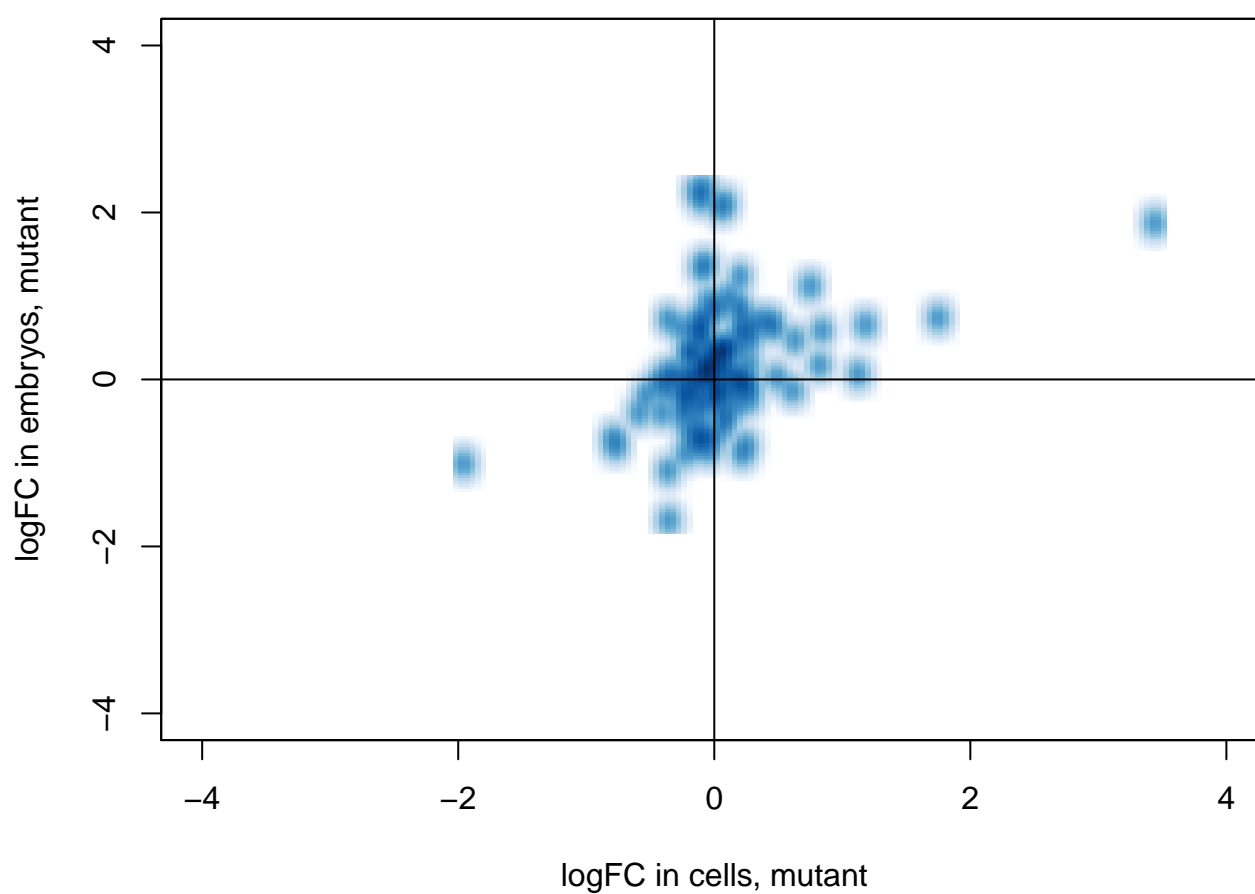

Supplement: File S1 — Archive (gzip2 compressed tar ball format) with 82 PDF files showing scatter plots for GO biological process terms with more than 50 annotated genes. Legend analogous to Figure 2. (BZ2) [file pone.0028985.s013.bz2 › scatterplots for 82 GO process terms with more than 50 genes/determination of adult lifespan-results.pdf]

### genes in DNA repair

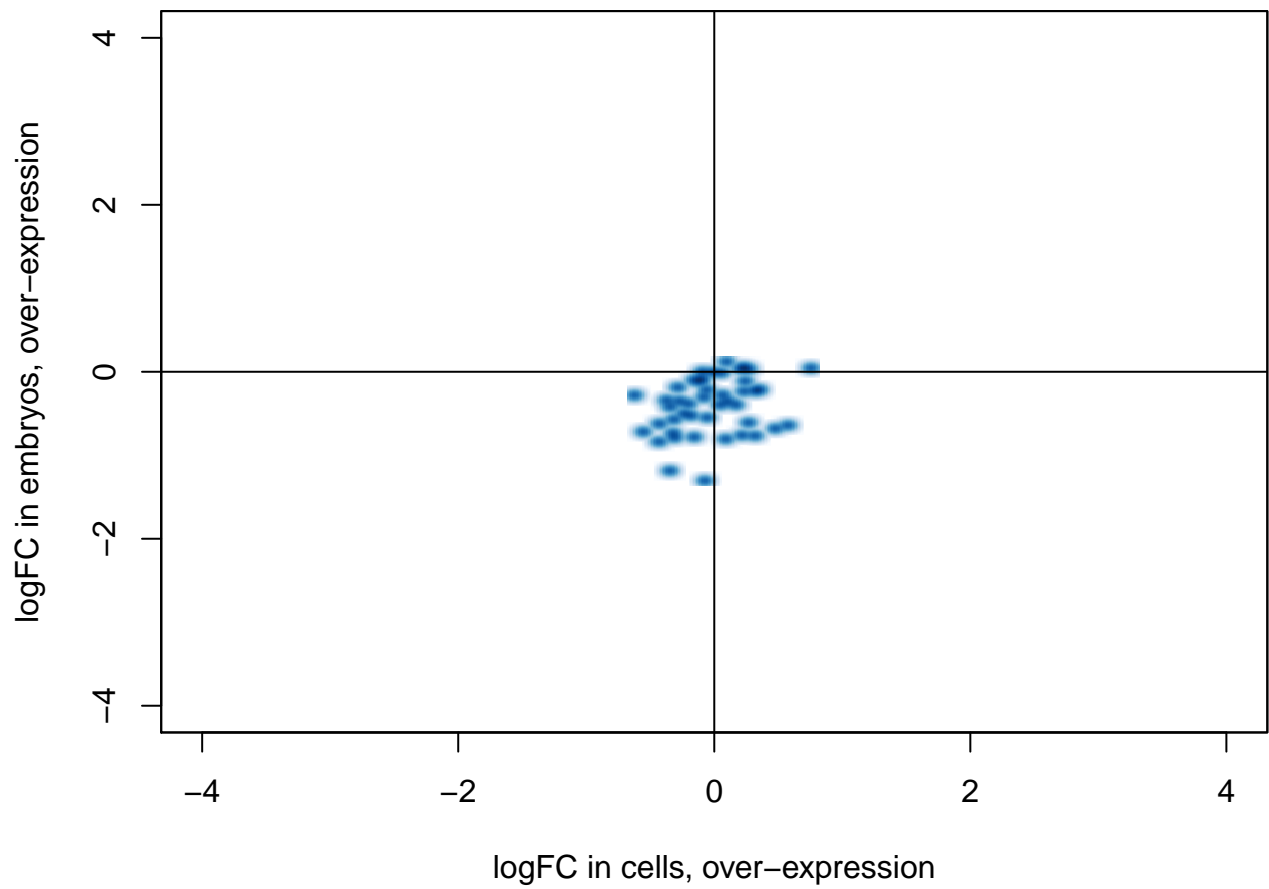

### genes in DNA repair

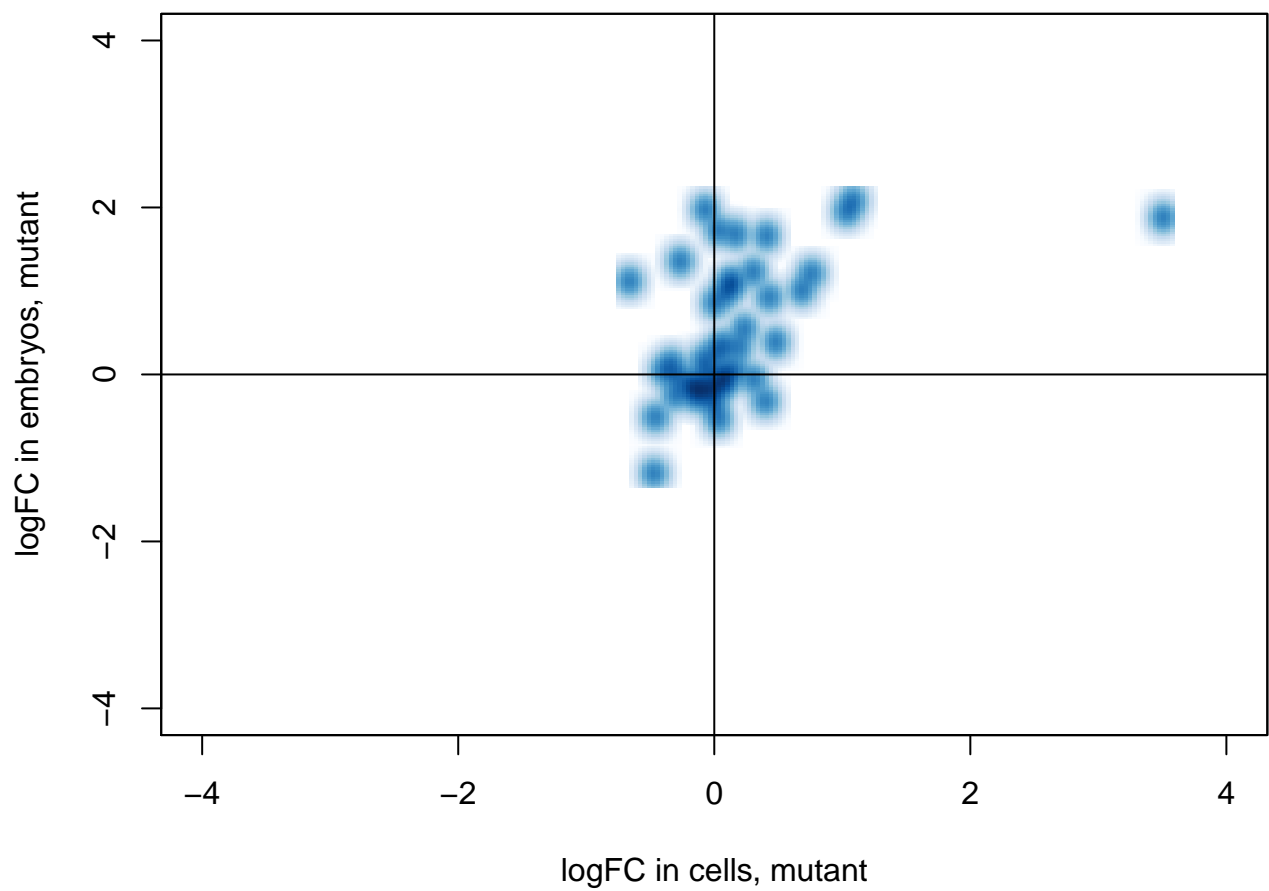

Supplement: File S1 — Archive (gzip2 compressed tar ball format) with 82 PDF files showing scatter plots for GO biological process terms with more than 50 annotated genes. Legend analogous to Figure 2. (BZ2) [file pone.0028985.s013.bz2 › scatterplots for 82 GO process terms with more than 50 genes/DNA repair-results.pdf]

### genes in dorsal closure

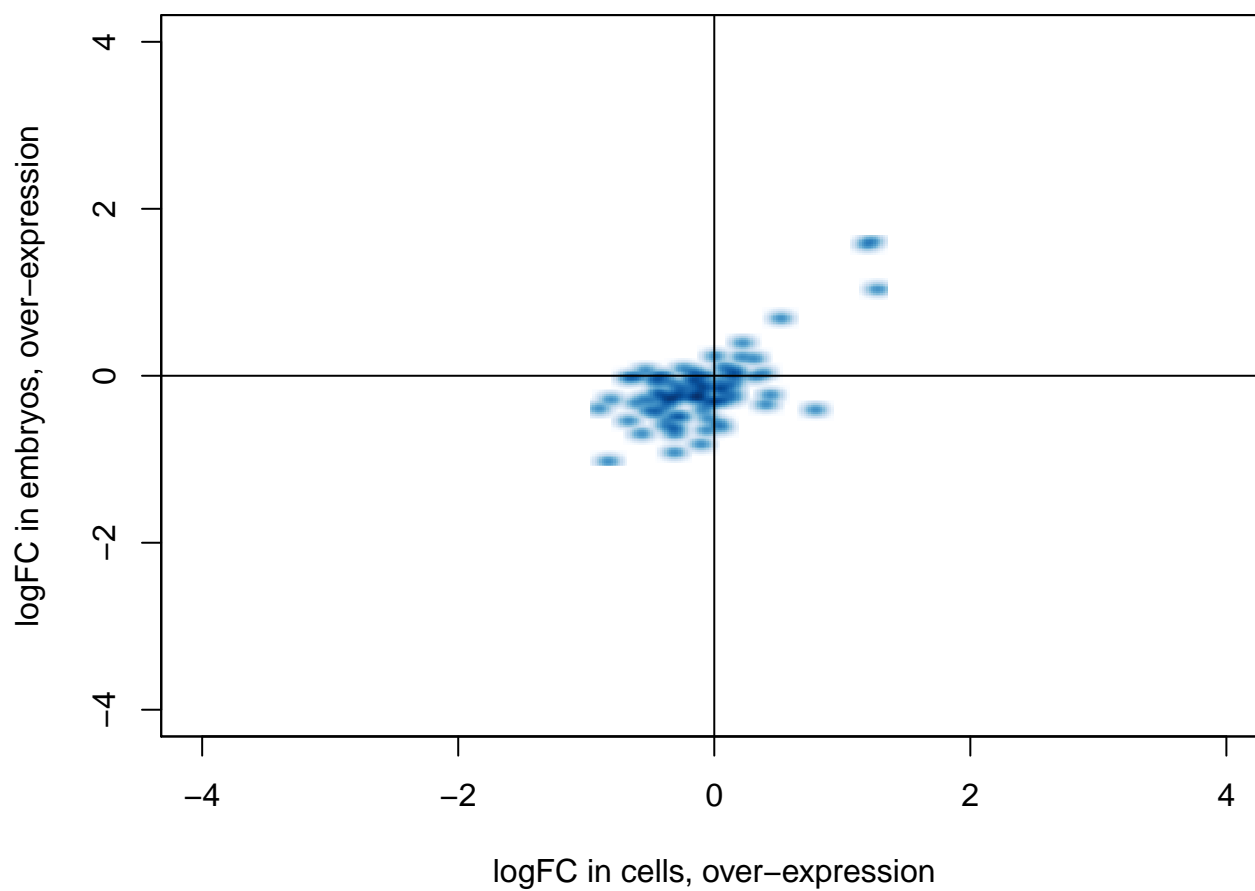

### genes in dorsal closure

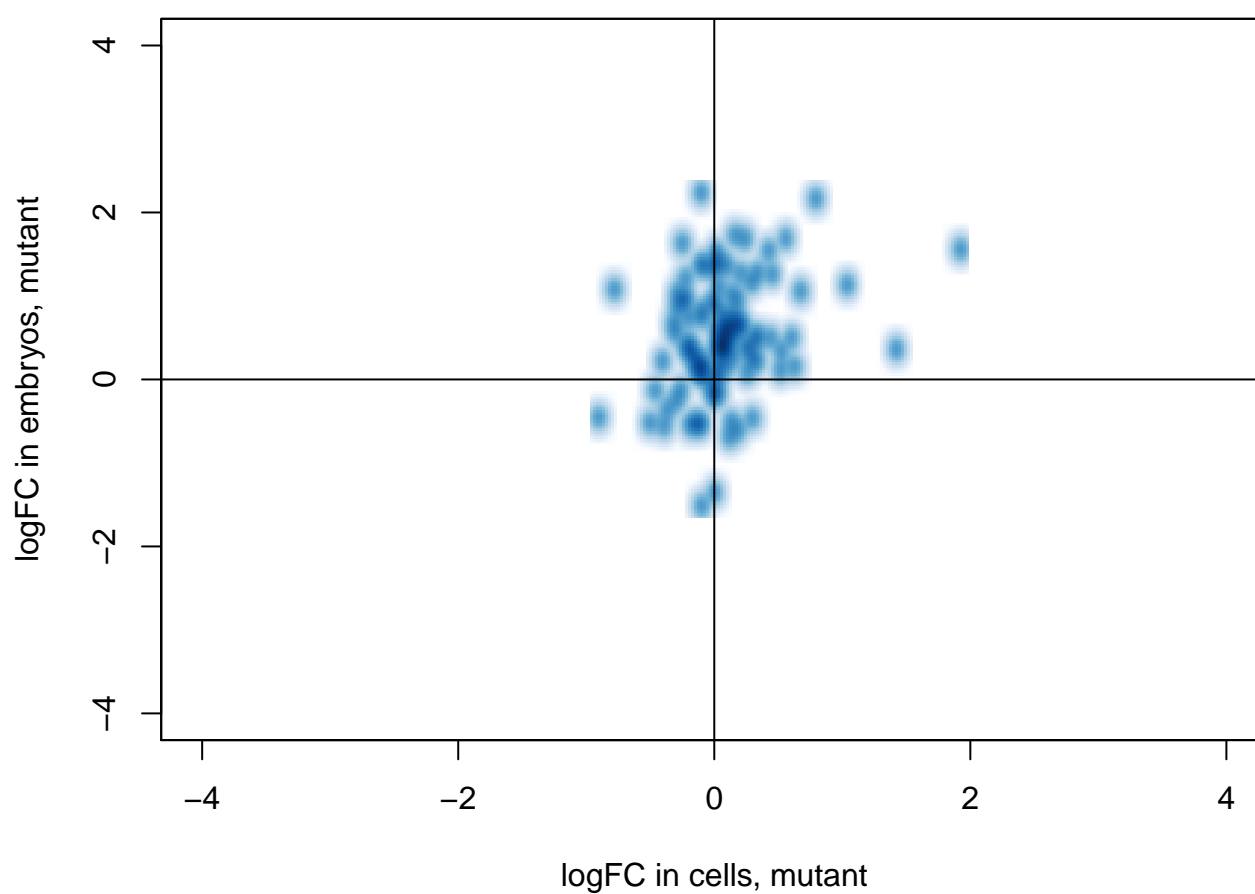

Supplement: File S1 — Archive (gzip2 compressed tar ball format) with 82 PDF files showing scatter plots for GO biological process terms with more than 50 annotated genes. Legend analogous to Figure 2. (BZ2) [file pone.0028985.s013.bz2 › scatterplots for 82 GO process terms with more than 50 genes/dorsal closure-results.pdf]

**genes in heart development**

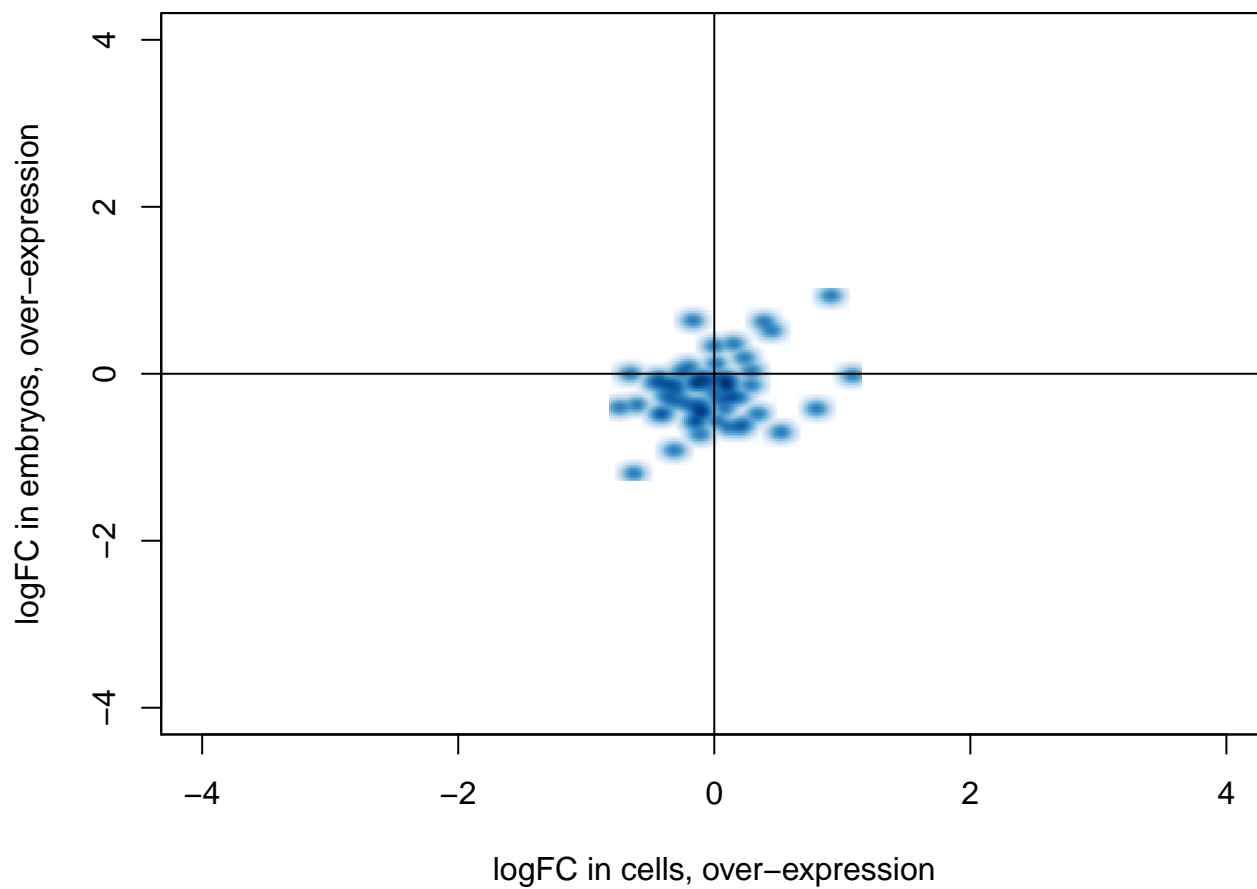

**genes in heart development**

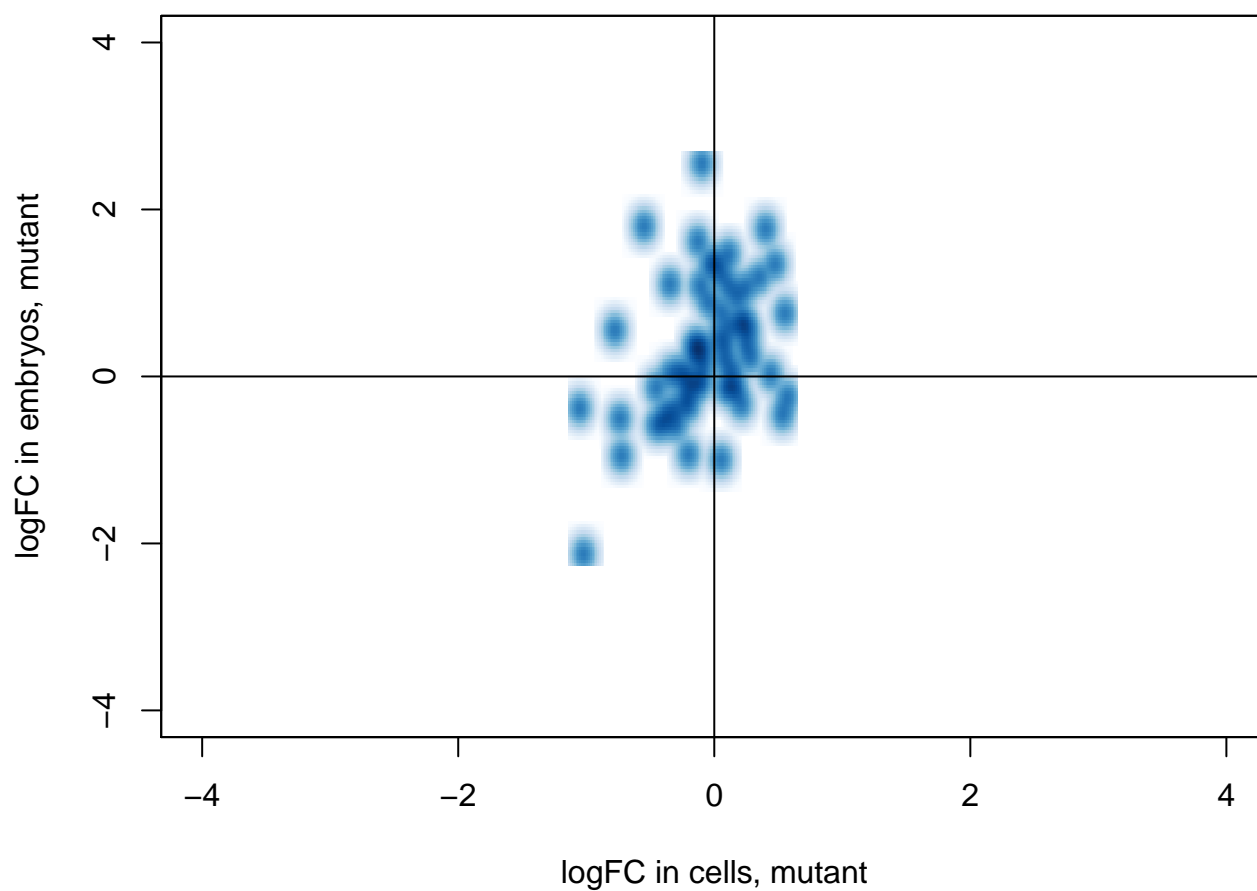

Supplement: File S1 — Archive (gzip2 compressed tar ball format) with 82 PDF files showing scatter plots for GO biological process terms with more than 50 annotated genes. Legend analogous to Figure 2. (BZ2) [file pone.0028985.s013.bz2 › scatterplots for 82 GO process terms with more than 50 genes/heart development-results.pdf]

### genes in intracellular protein transport

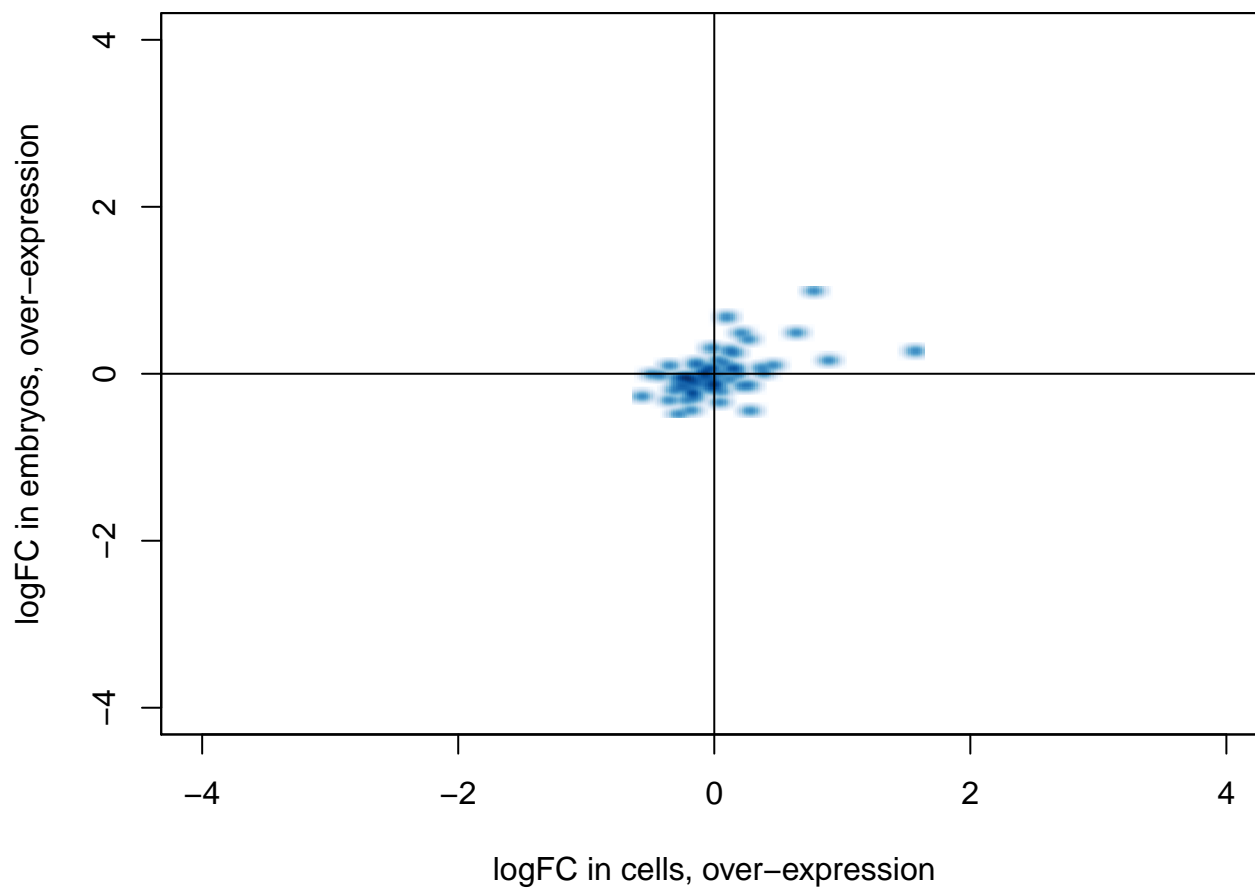

### genes in intracellular protein transport

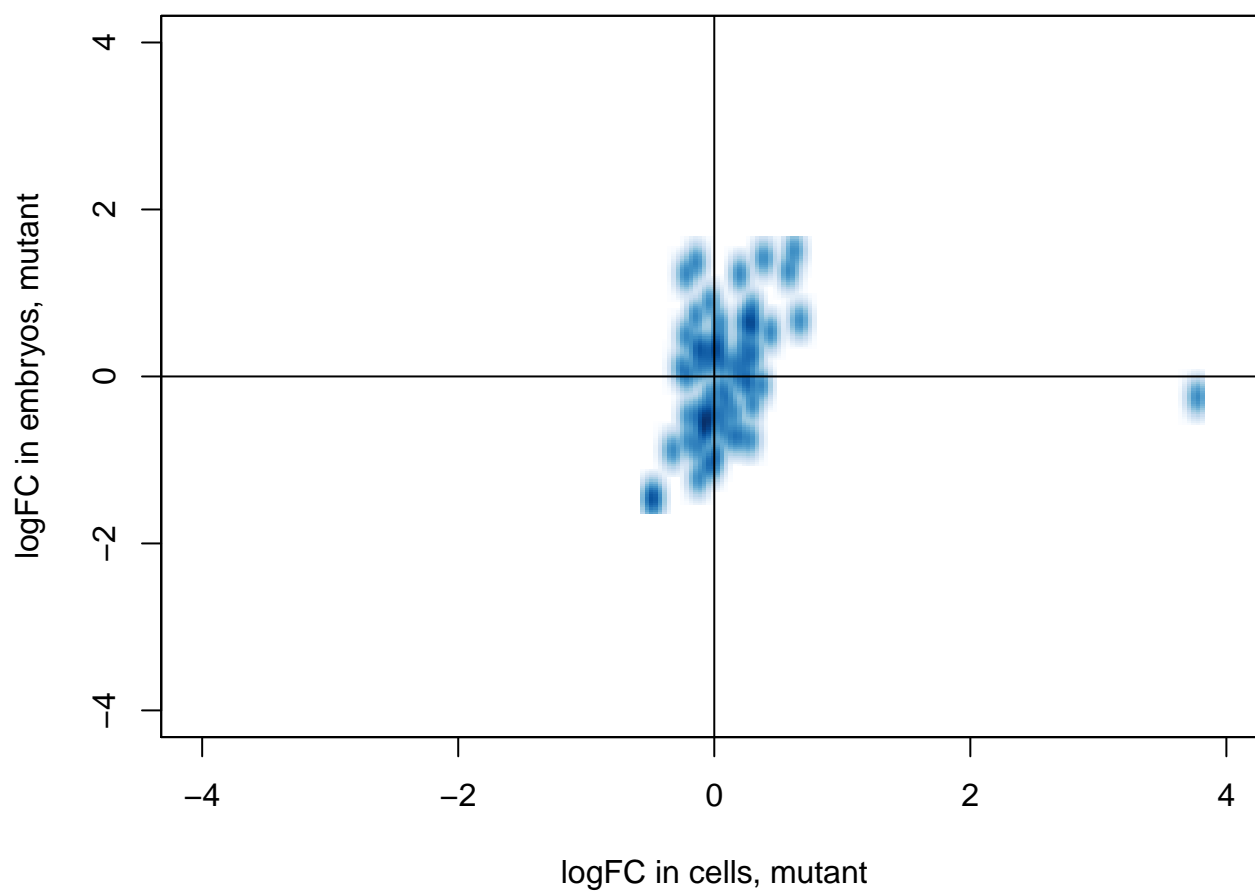

Supplement: File S1 — Archive (gzip2 compressed tar ball format) with 82 PDF files showing scatter plots for GO biological process terms with more than 50 annotated genes. Legend analogous to Figure 2. (BZ2) [file pone.0028985.s013.bz2 › scatterplots for 82 GO process terms with more than 50 genes/intracellular protein transport-results.pdf]

### genes in intracellular signal transduction

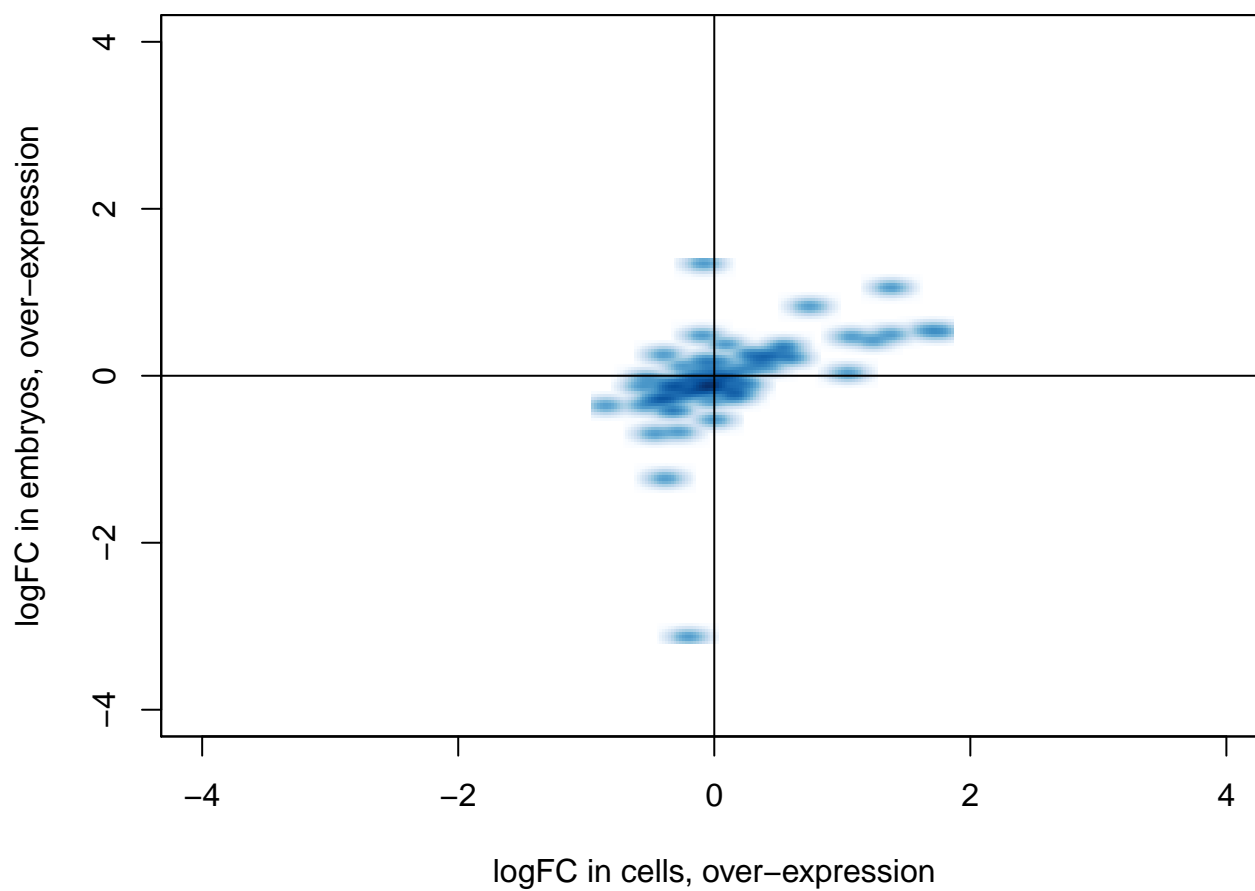

### genes in intracellular signal transduction

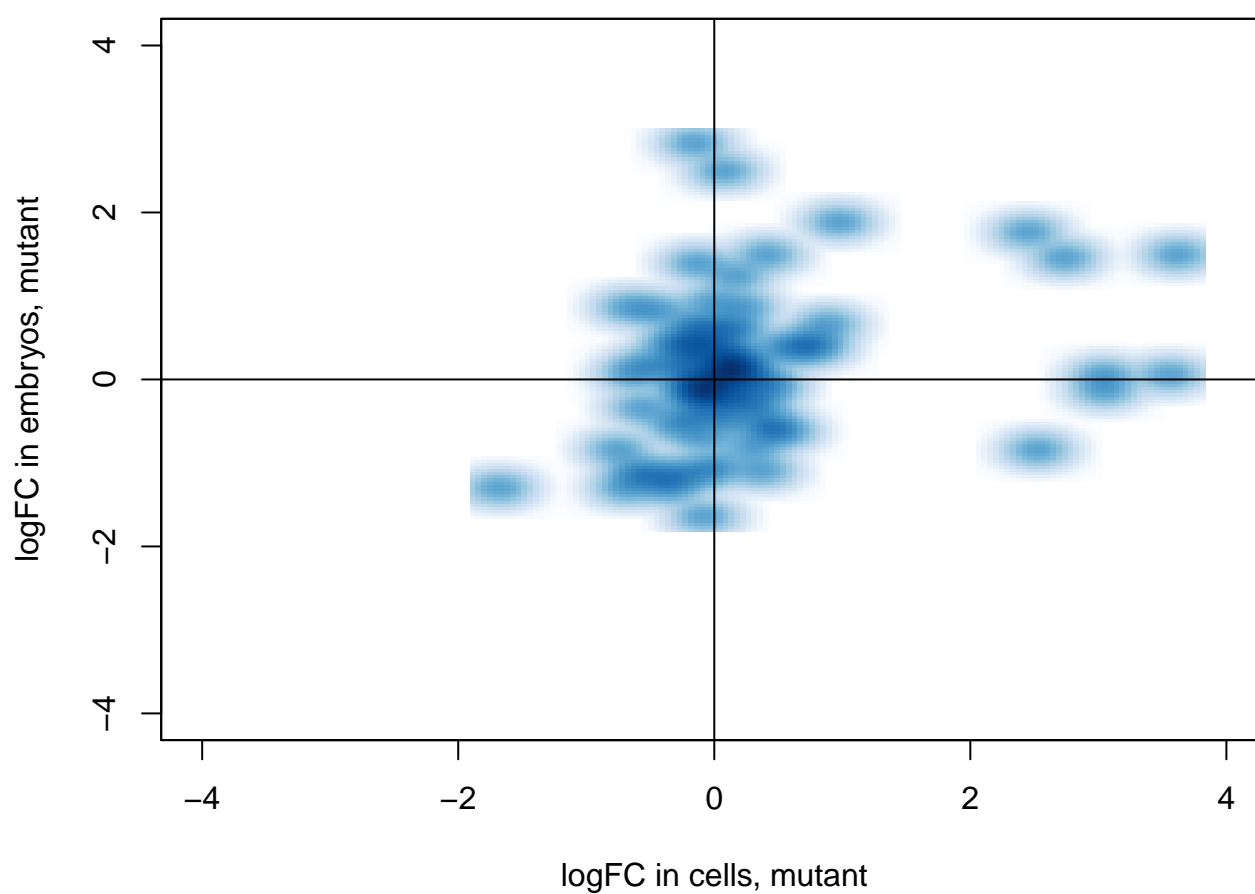

Supplement: File S1 — Archive (gzip2 compressed tar ball format) with 82 PDF files showing scatter plots for GO biological process terms with more than 50 annotated genes. Legend analogous to Figure 2. (BZ2) [file pone.0028985.s013.bz2 › scatterplots for 82 GO process terms with more than 50 genes/intracellular signal transduction-results.pdf]

**genes in ion transport**

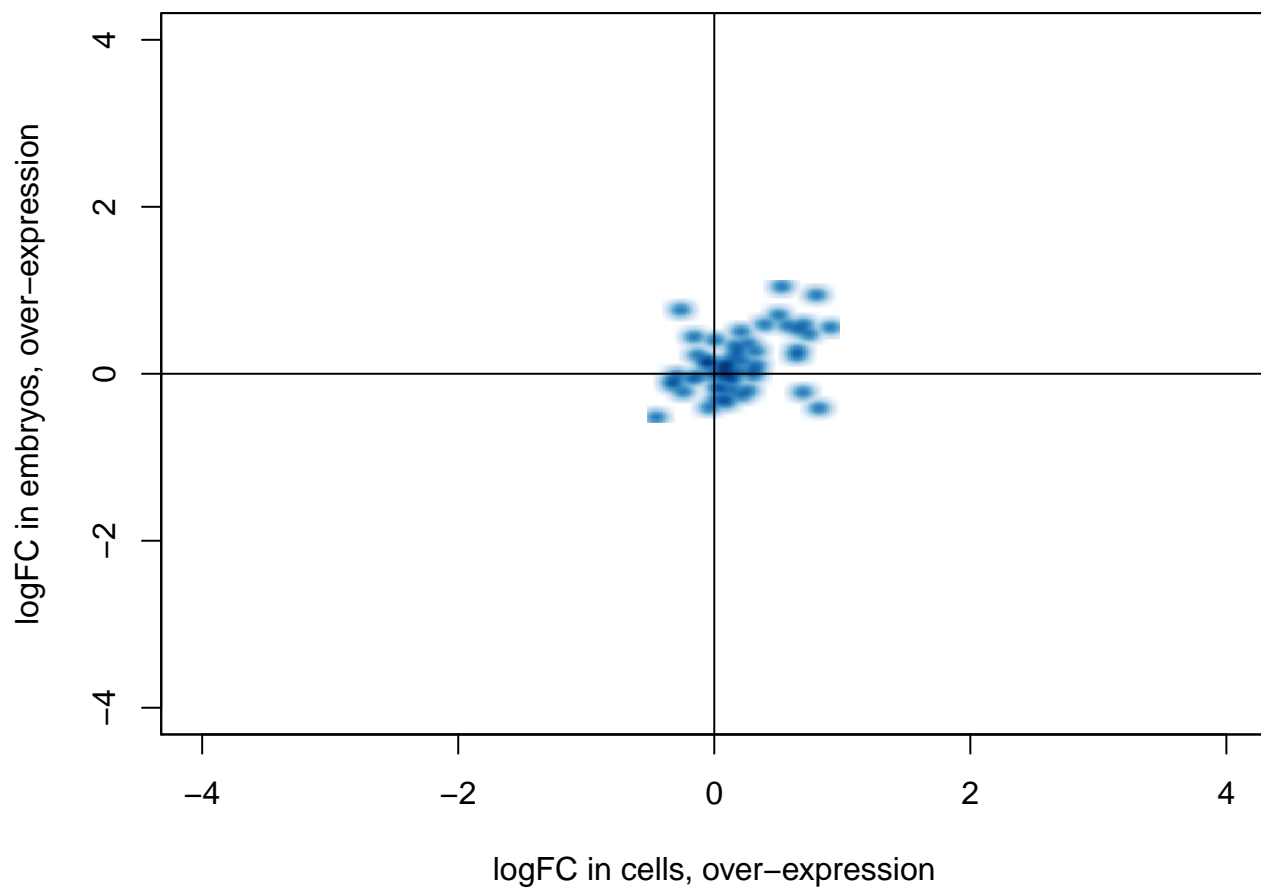

**genes in ion transport**

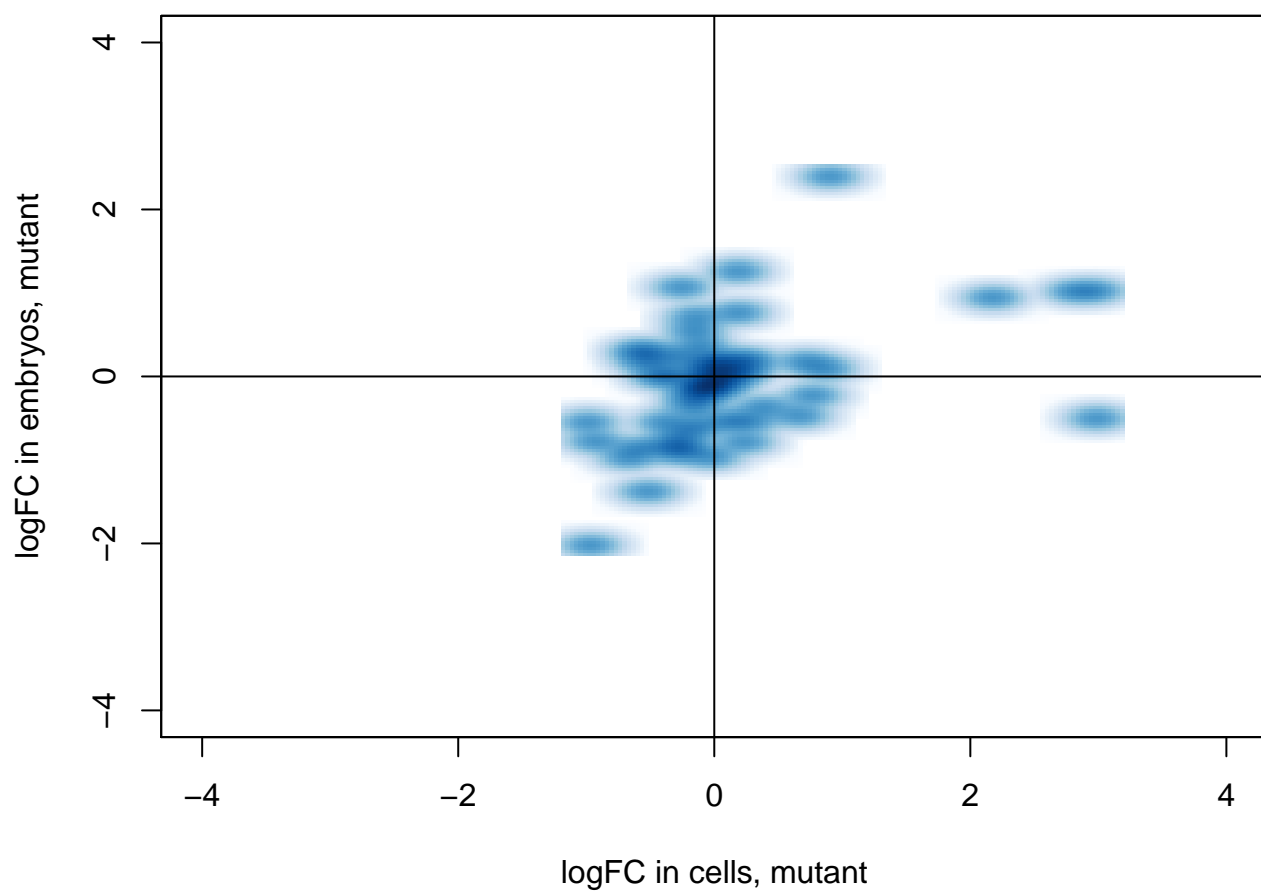

Supplement: File S1 — Archive (gzip2 compressed tar ball format) with 82 PDF files showing scatter plots for GO biological process terms with more than 50 annotated genes. Legend analogous to Figure 2. (BZ2) [file pone.0028985.s013.bz2 › scatterplots for 82 GO process terms with more than 50 genes/ion transport-results.pdf]

### genes in lipid metabolic process

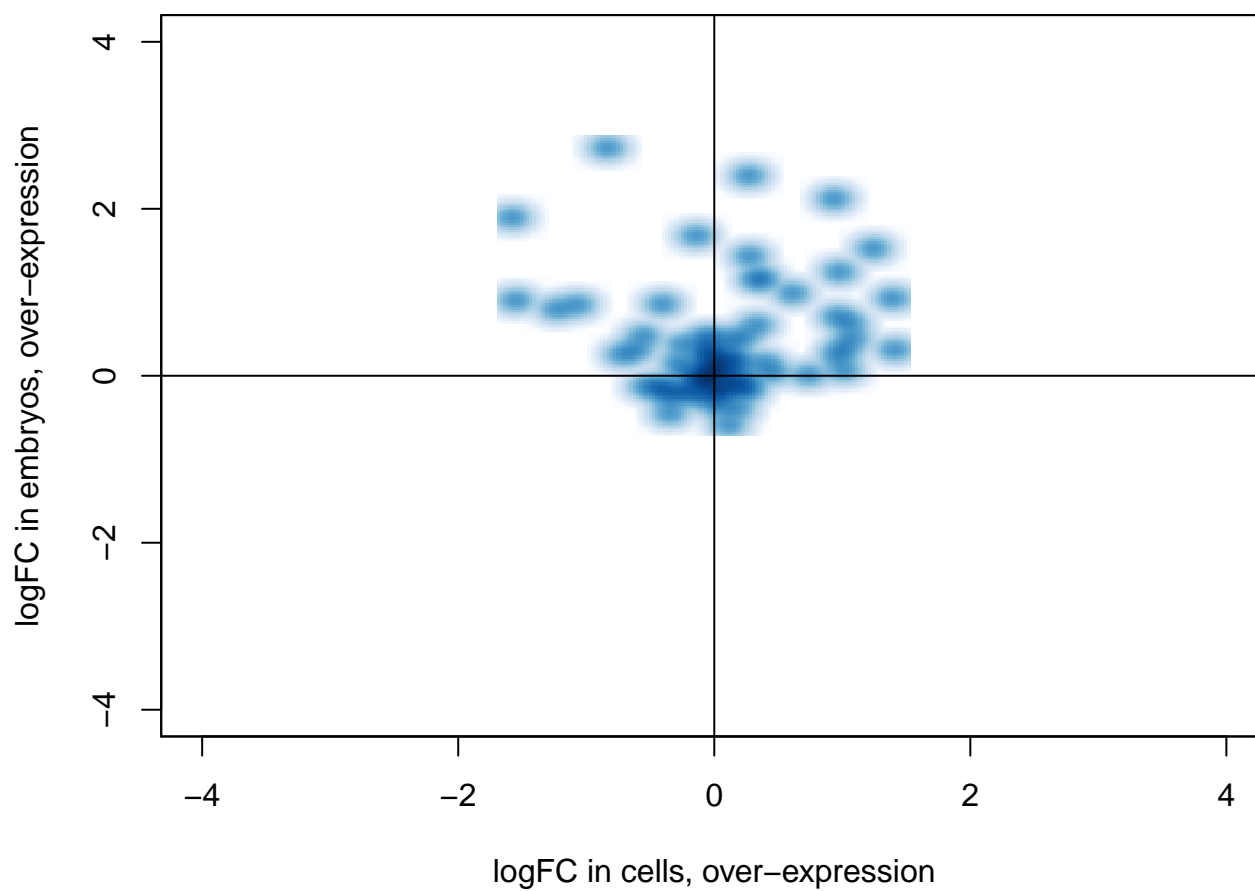

### genes in lipid metabolic process

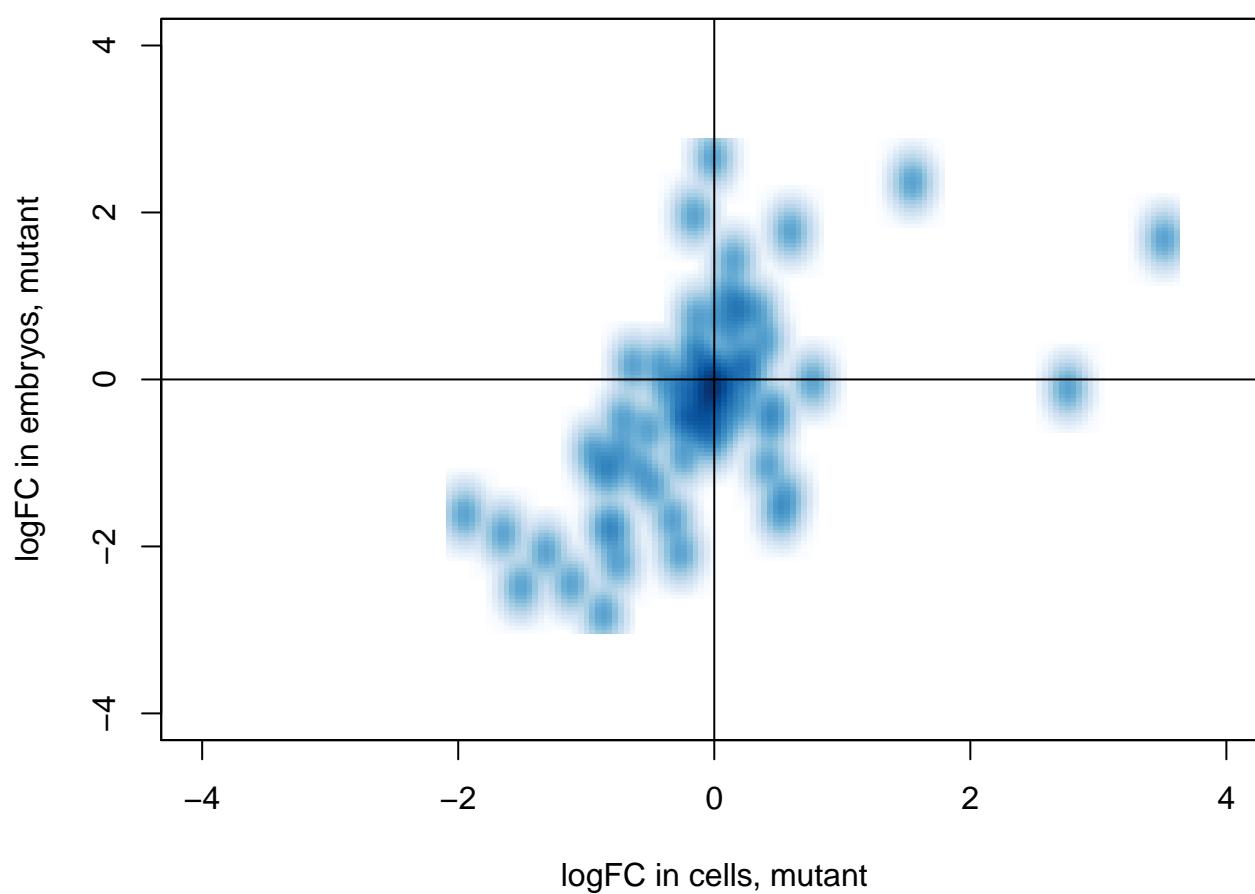

Supplement: File S1 — Archive (gzip2 compressed tar ball format) with 82 PDF files showing scatter plots for GO biological process terms with more than 50 annotated genes. Legend analogous to Figure 2. (BZ2) [file pone.0028985.s013.bz2 › scatterplots for 82 GO process terms with more than 50 genes/lipid metabolic process-results.pdf]

### genes in mesoderm development

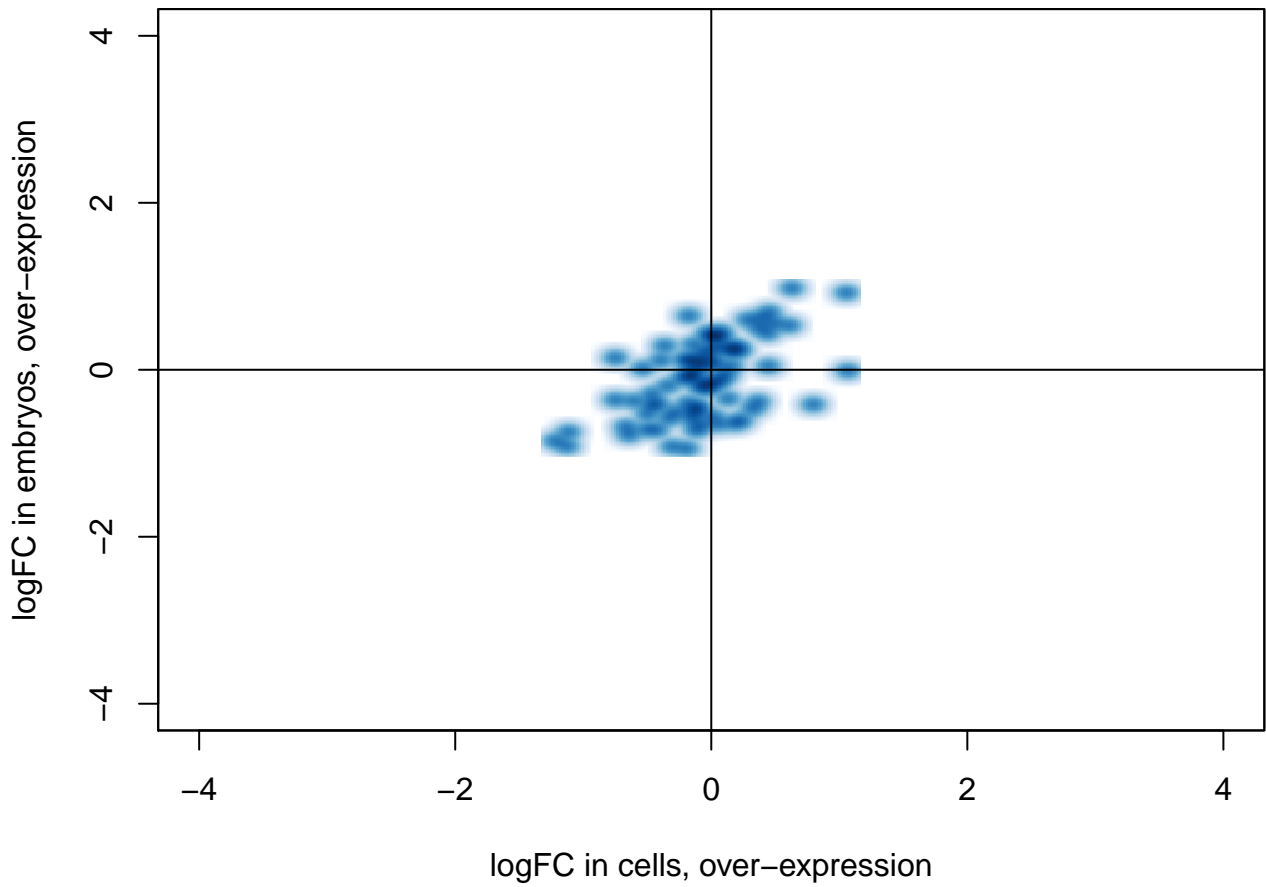

### genes in mesoderm development

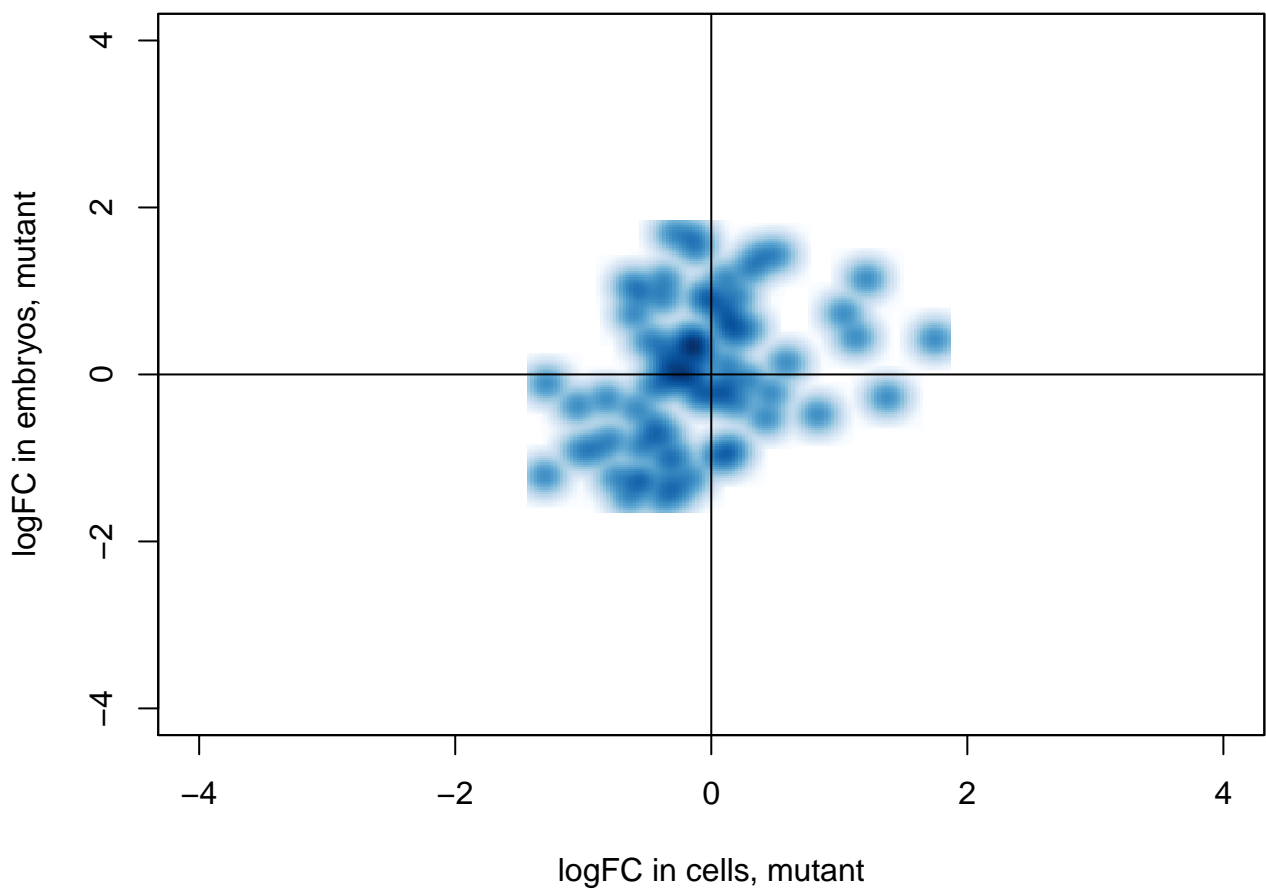

Supplement: File S1 — Archive (gzip2 compressed tar ball format) with 82 PDF files showing scatter plots for GO biological process terms with more than 50 annotated genes. Legend analogous to Figure 2. (BZ2) [file pone.0028985.s013.bz2 › scatterplots for 82 GO process terms with more than 50 genes/mesoderm development-results.pdf]

### genes in metabolic process

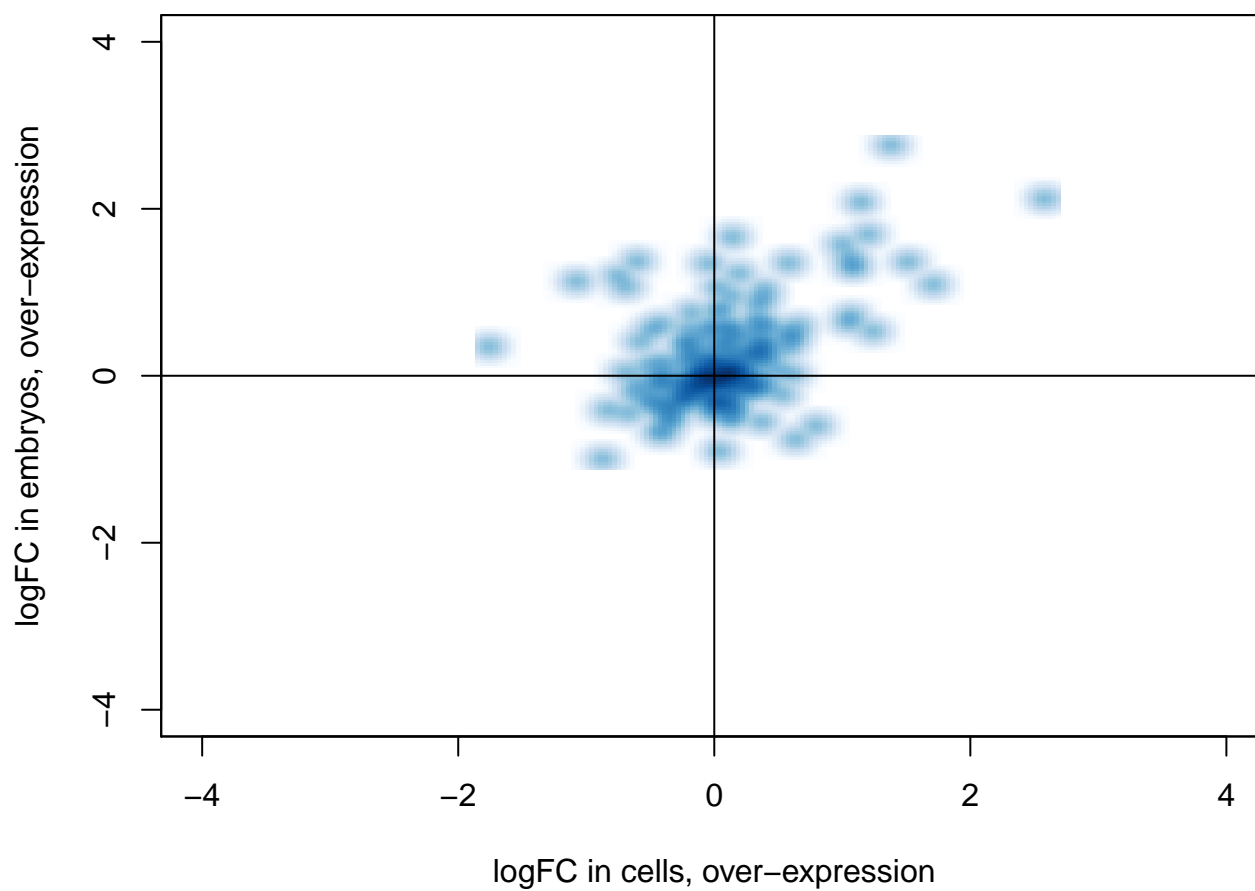

### genes in metabolic process

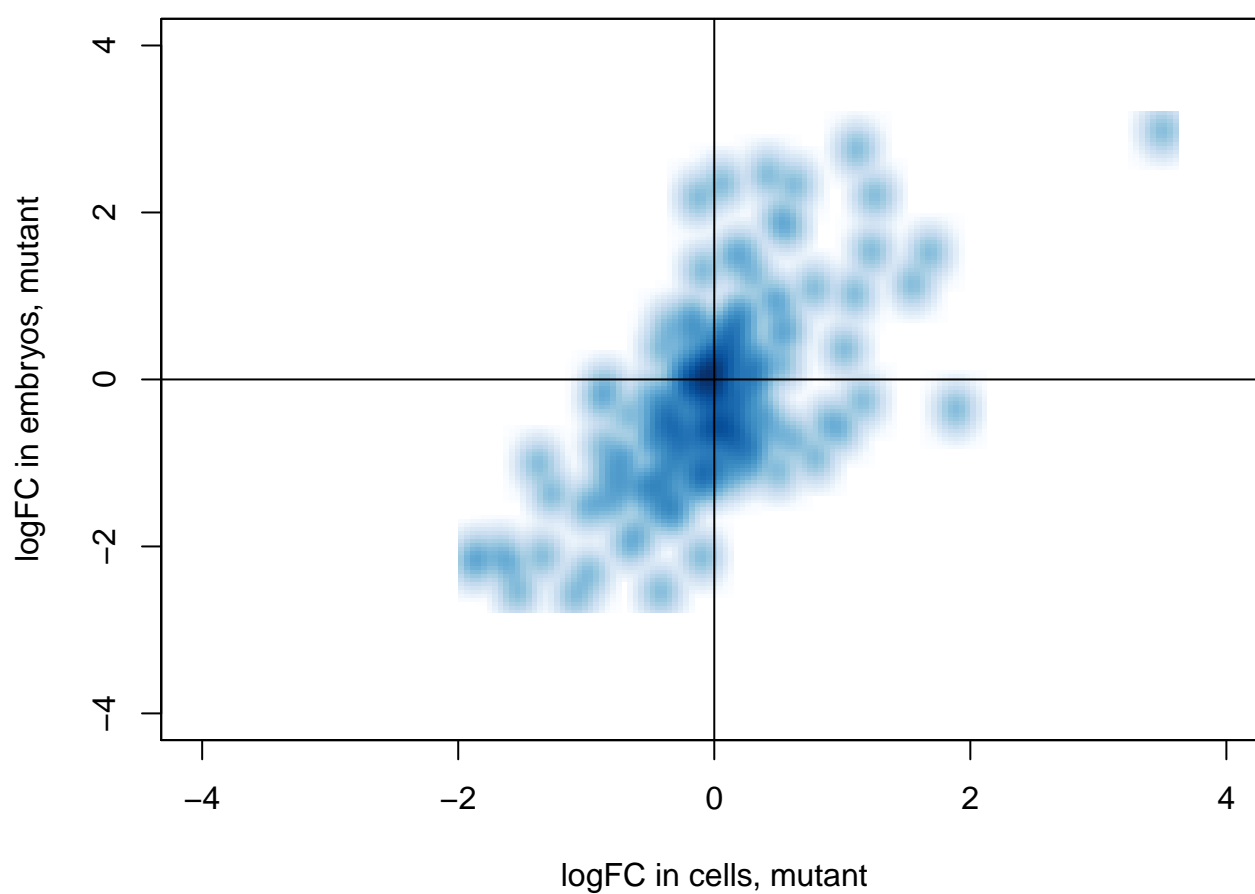

Supplement: File S1 — Archive (gzip2 compressed tar ball format) with 82 PDF files showing scatter plots for GO biological process terms with more than 50 annotated genes. Legend analogous to Figure 2. (BZ2) [file pone.0028985.s013.bz2 › scatterplots for 82 GO process terms with more than 50 genes/metabolic process-results.pdf]

### genes in microtubule-based movement

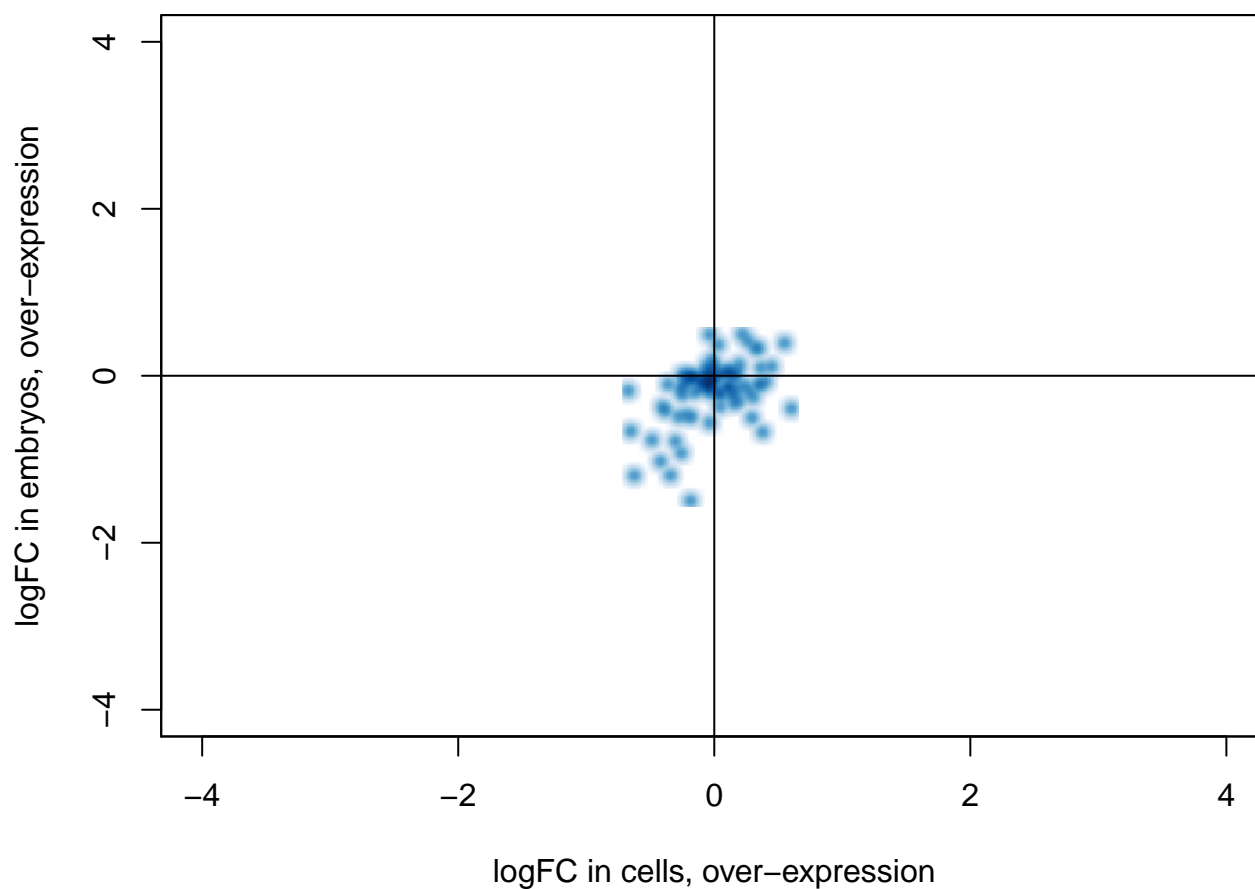

### genes in microtubule-based movement

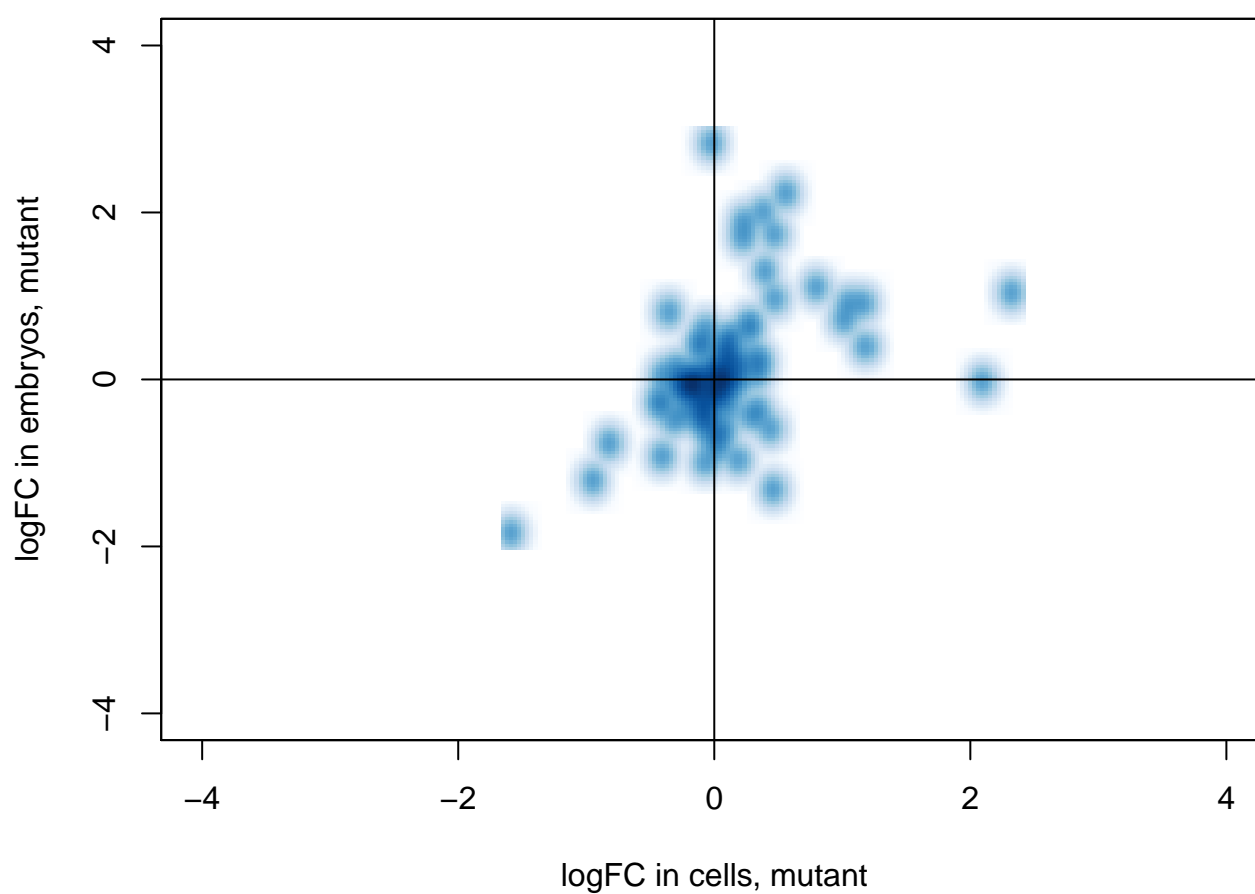

Supplement: File S1 — Archive (gzip2 compressed tar ball format) with 82 PDF files showing scatter plots for GO biological process terms with more than 50 annotated genes. Legend analogous to Figure 2. (BZ2) [file pone.0028985.s013.bz2 › scatterplots for 82 GO process terms with more than 50 genes/microtubule-based movement-results.pdf]

### genes in mitosis

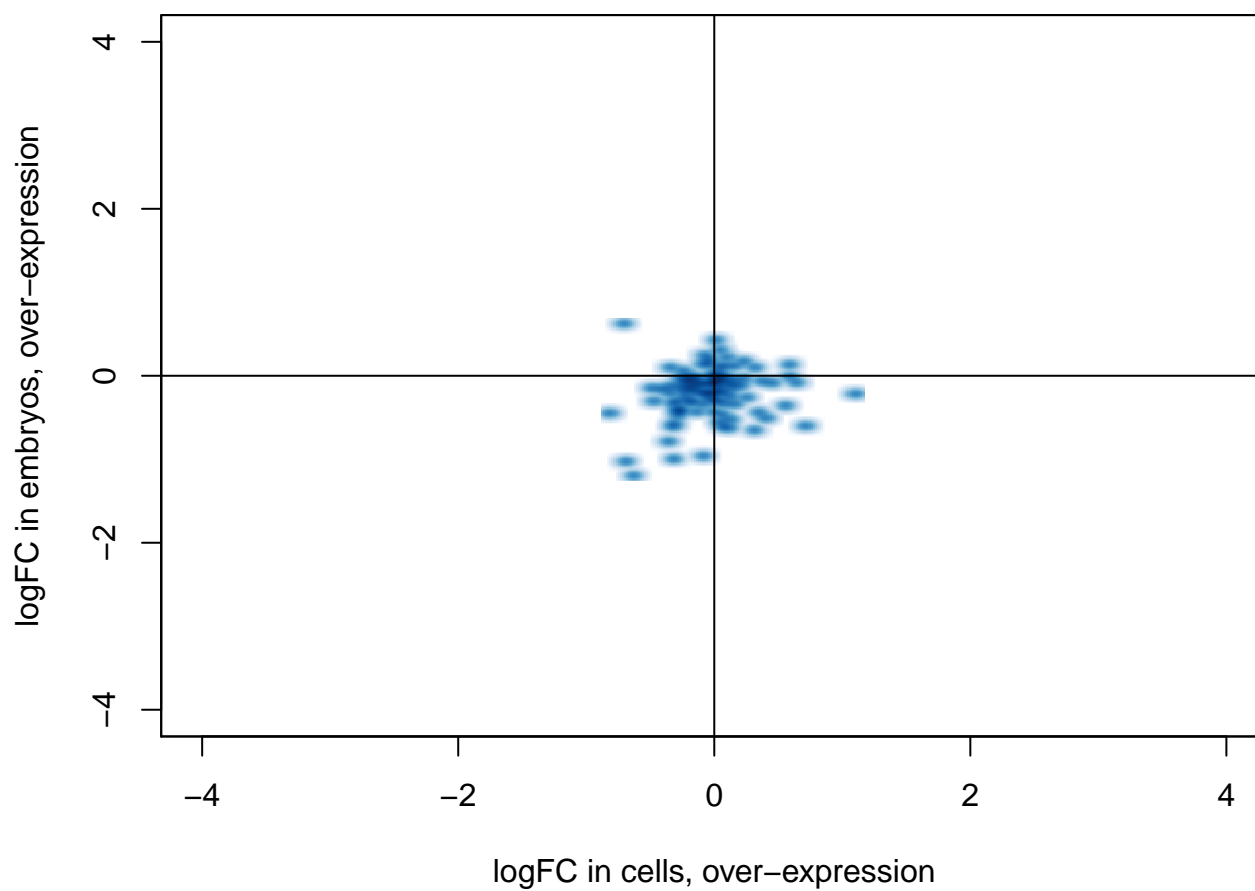

### genes in mitosis

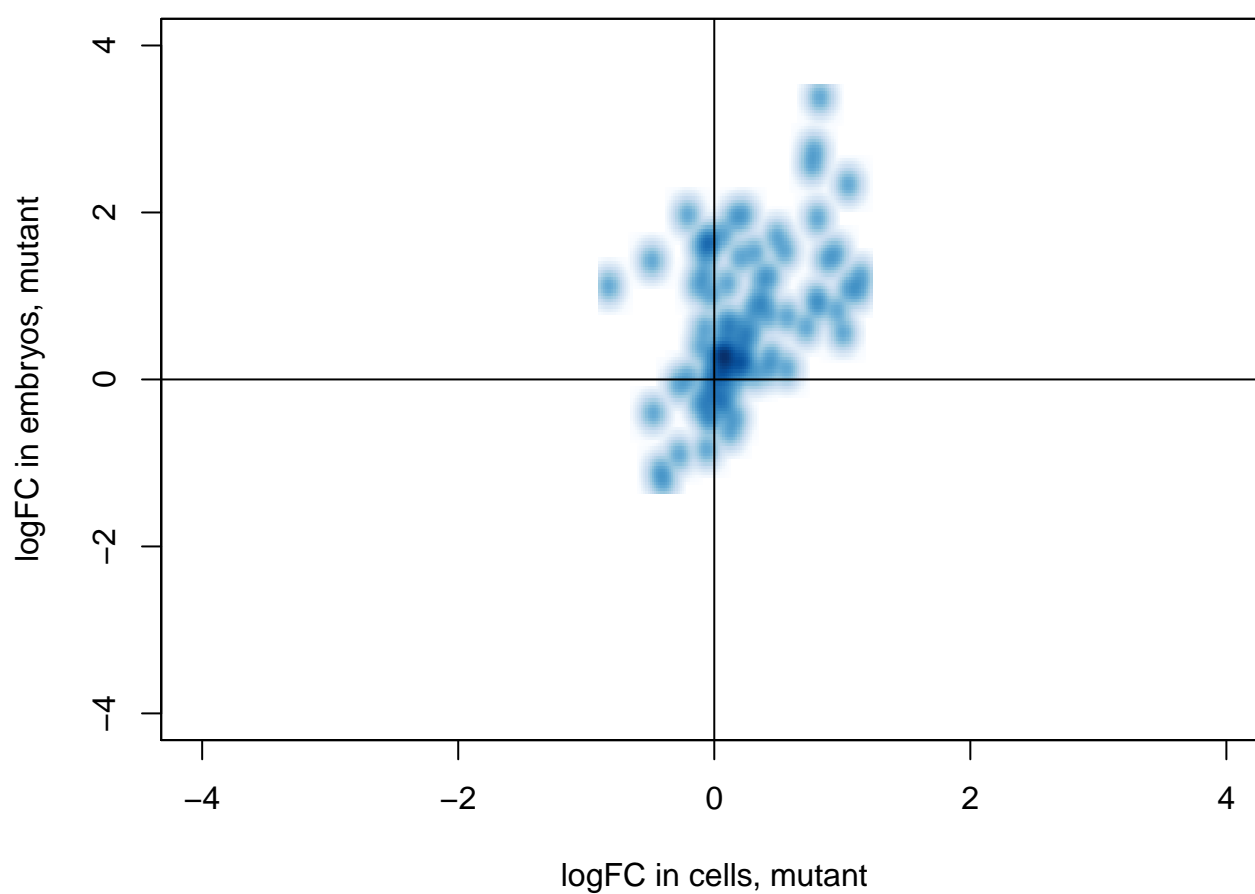

Supplement: File S1 — Archive (gzip2 compressed tar ball format) with 82 PDF files showing scatter plots for GO biological process terms with more than 50 annotated genes. Legend analogous to Figure 2. (BZ2) [file pone.0028985.s013.bz2 › scatterplots for 82 GO process terms with more than 50 genes/mitosis-results.pdf]

### genes in mitotic spindle elongation

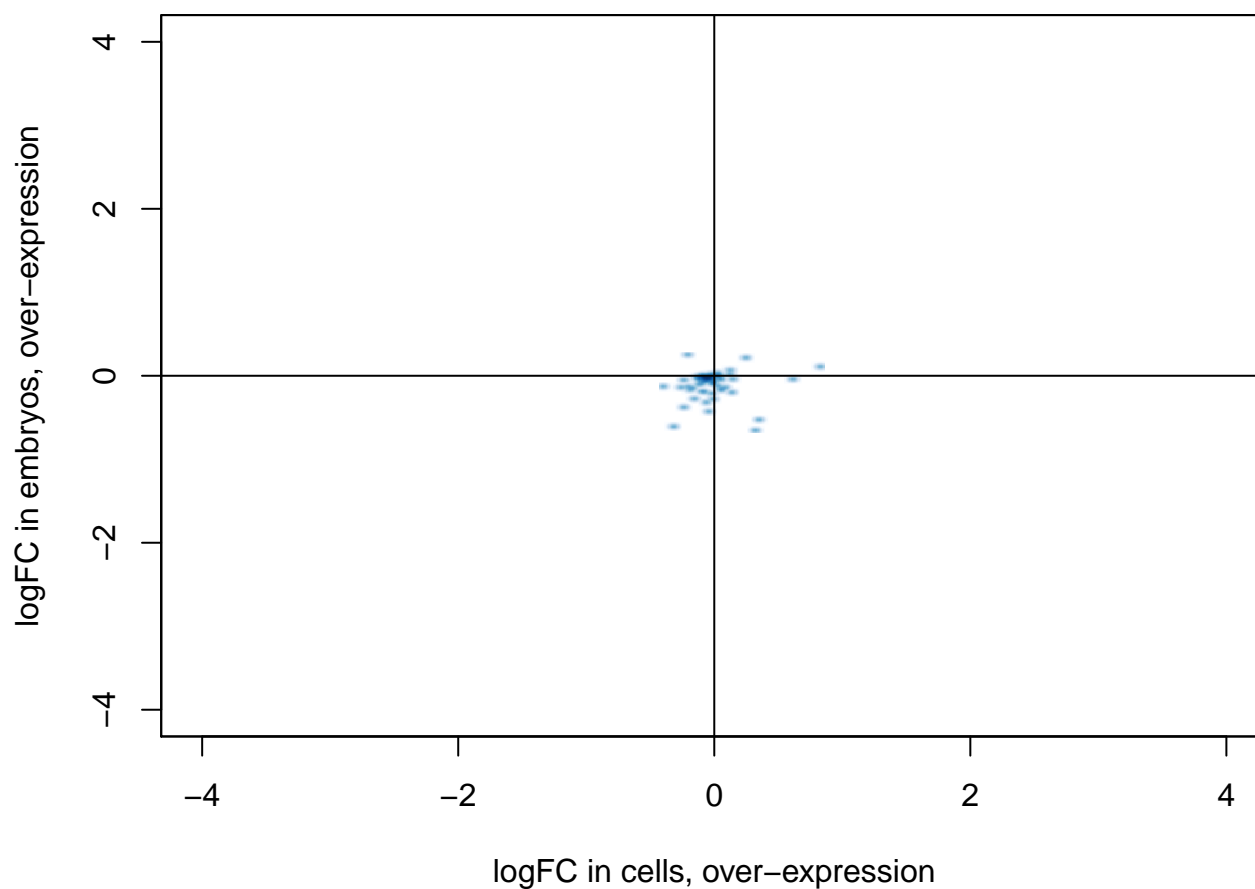

### genes in mitotic spindle elongation

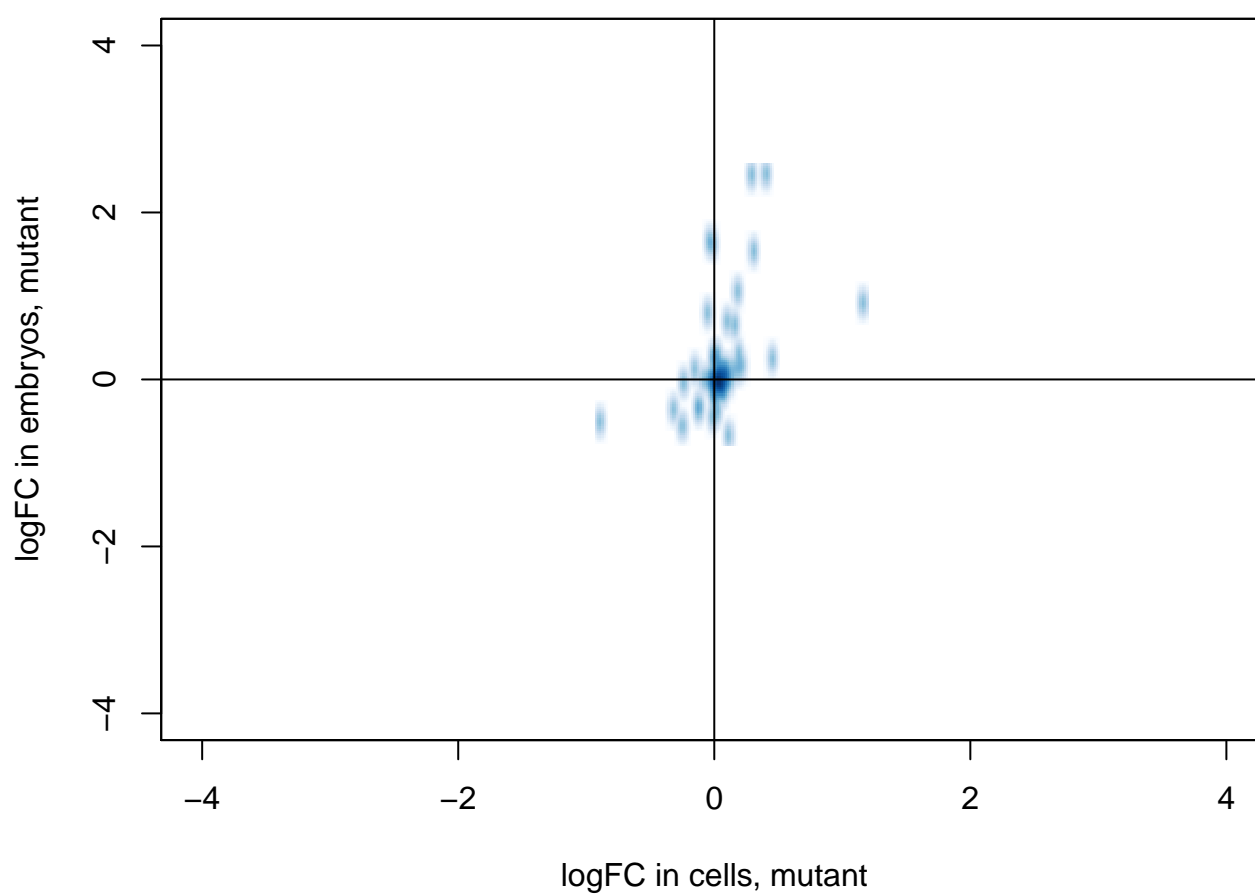

Supplement: File S1 — Archive (gzip2 compressed tar ball format) with 82 PDF files showing scatter plots for GO biological process terms with more than 50 annotated genes. Legend analogous to Figure 2. (BZ2) [file pone.0028985.s013.bz2 › scatterplots for 82 GO process terms with more than 50 genes/mitotic spindle elongation-results.pdf]

### genes in mitotic spindle organization

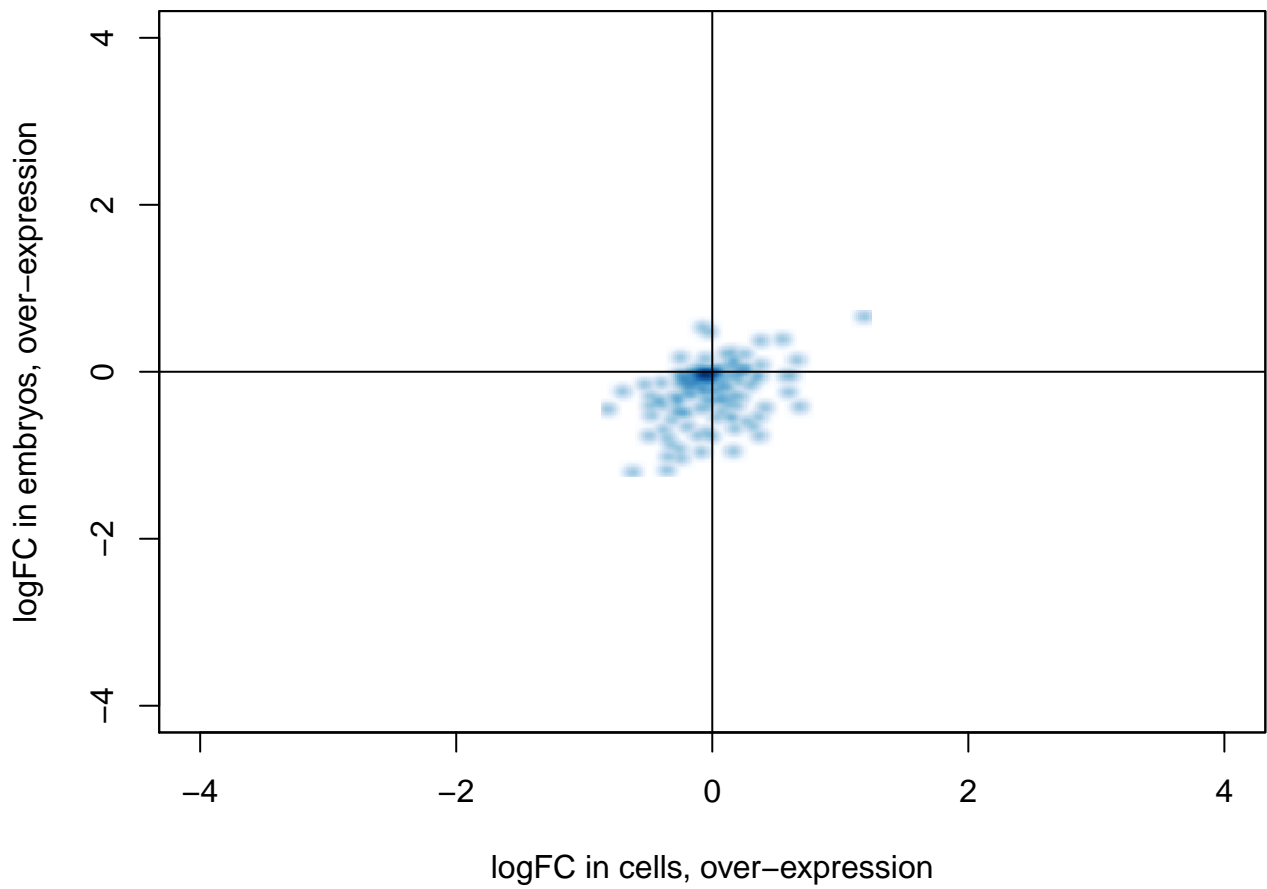

### genes in mitotic spindle organization

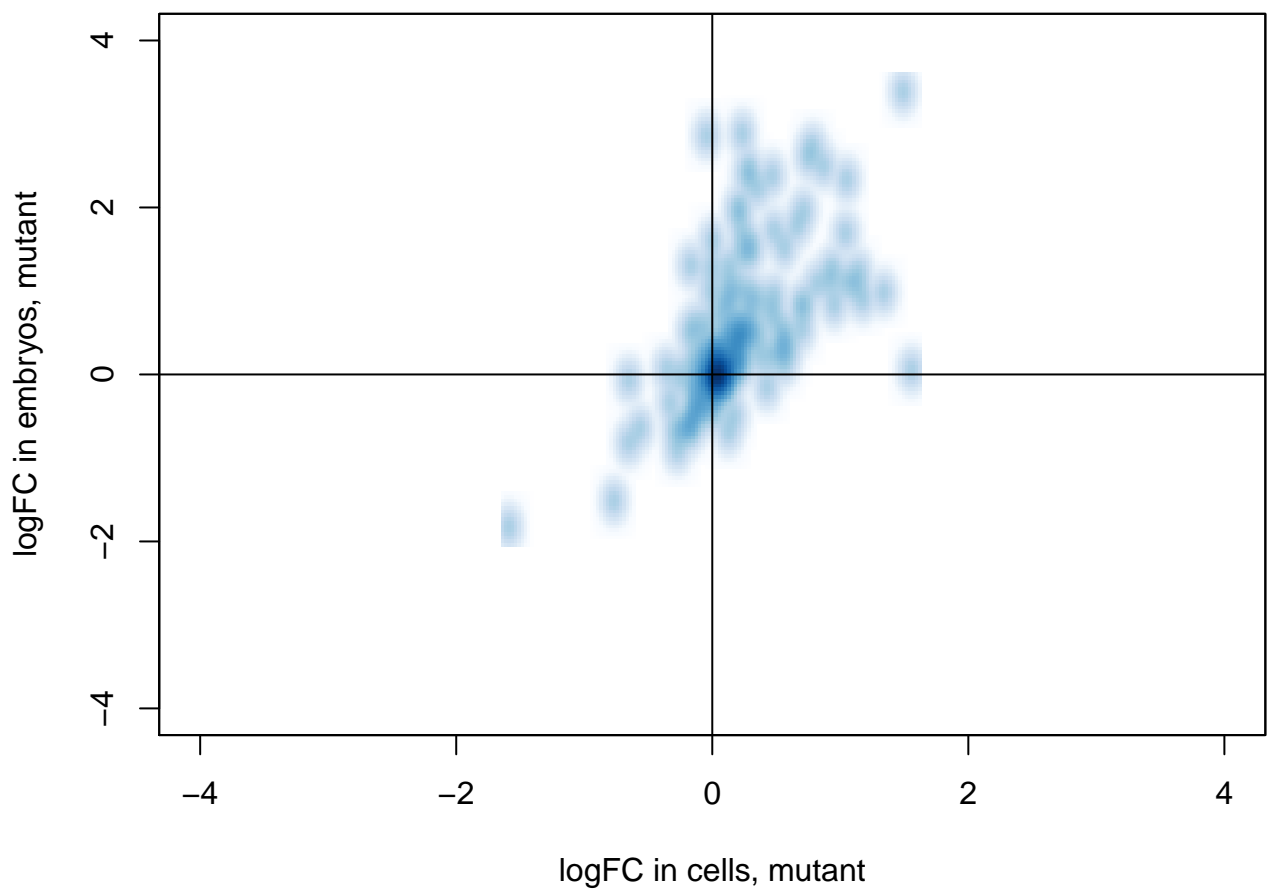

Supplement: File S1 — Archive (gzip2 compressed tar ball format) with 82 PDF files showing scatter plots for GO biological process terms with more than 50 annotated genes. Legend analogous to Figure 2. (BZ2) [file pone.0028985.s013.bz2 › scatterplots for 82 GO process terms with more than 50 genes/mitotic spindle organization-results.pdf]

### genes in muscle organ development

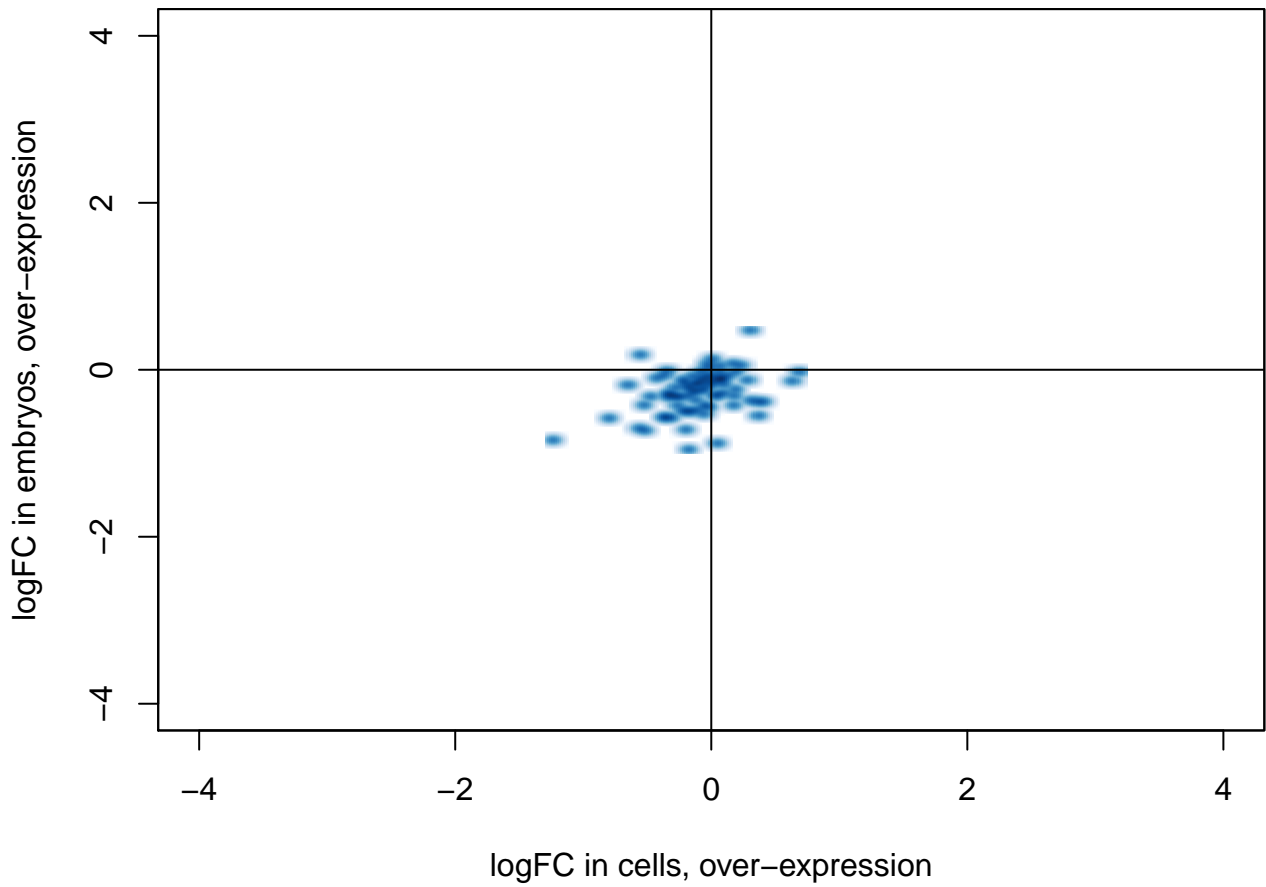

### genes in muscle organ development

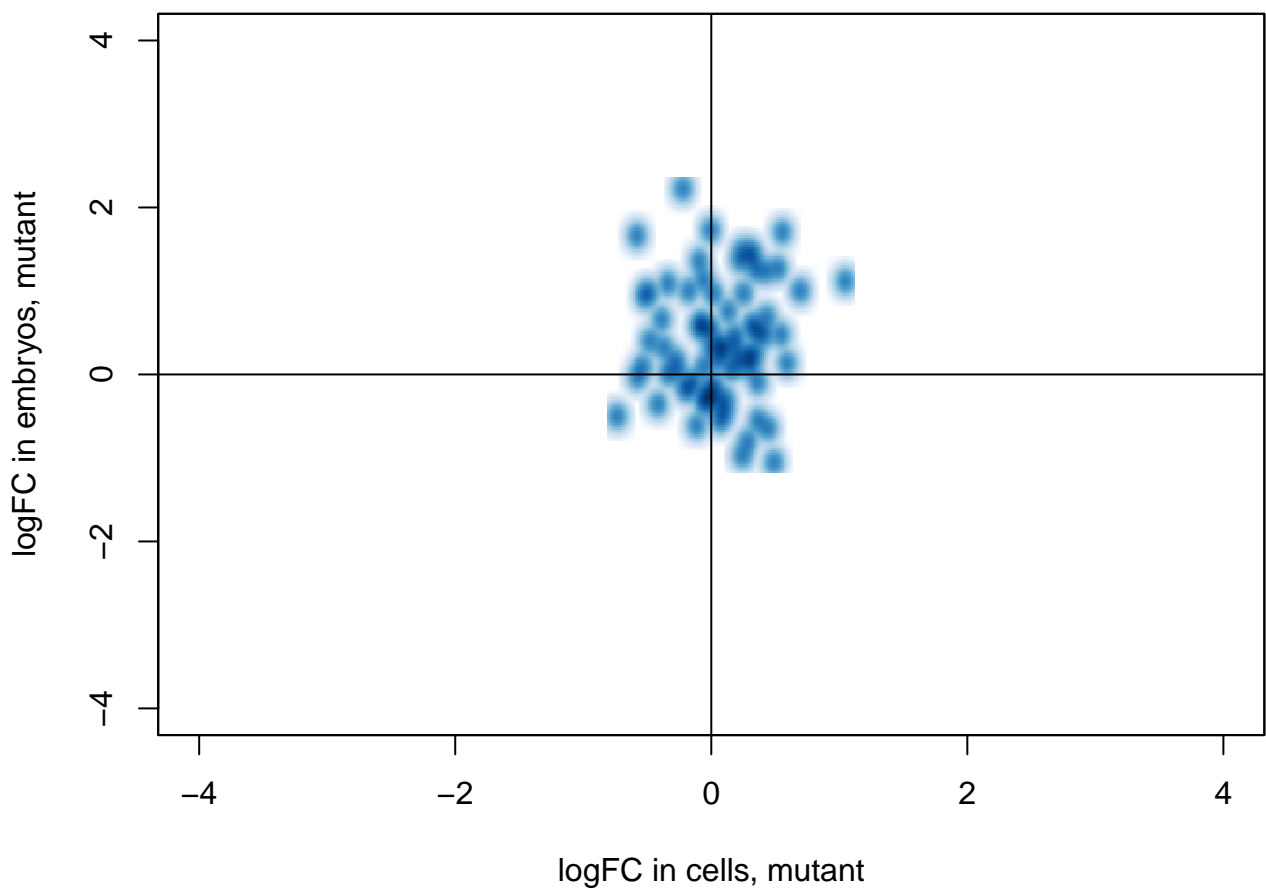

Supplement: File S1 — Archive (gzip2 compressed tar ball format) with 82 PDF files showing scatter plots for GO biological process terms with more than 50 annotated genes. Legend analogous to Figure 2. (BZ2) [file pone.0028985.s013.bz2 › scatterplots for 82 GO process terms with more than 50 genes/muscle organ development-results.pdf]

### genes in mushroom body development

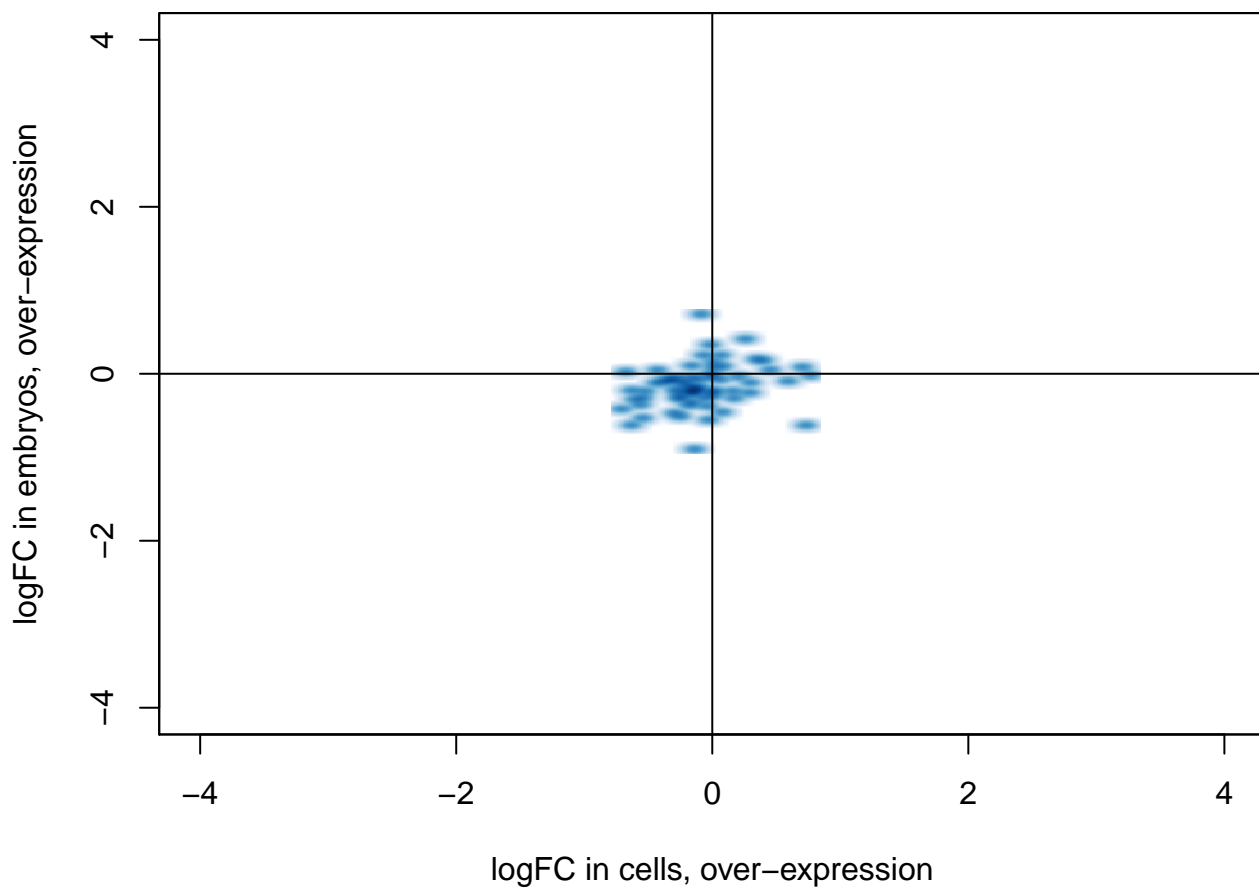

### genes in mushroom body development

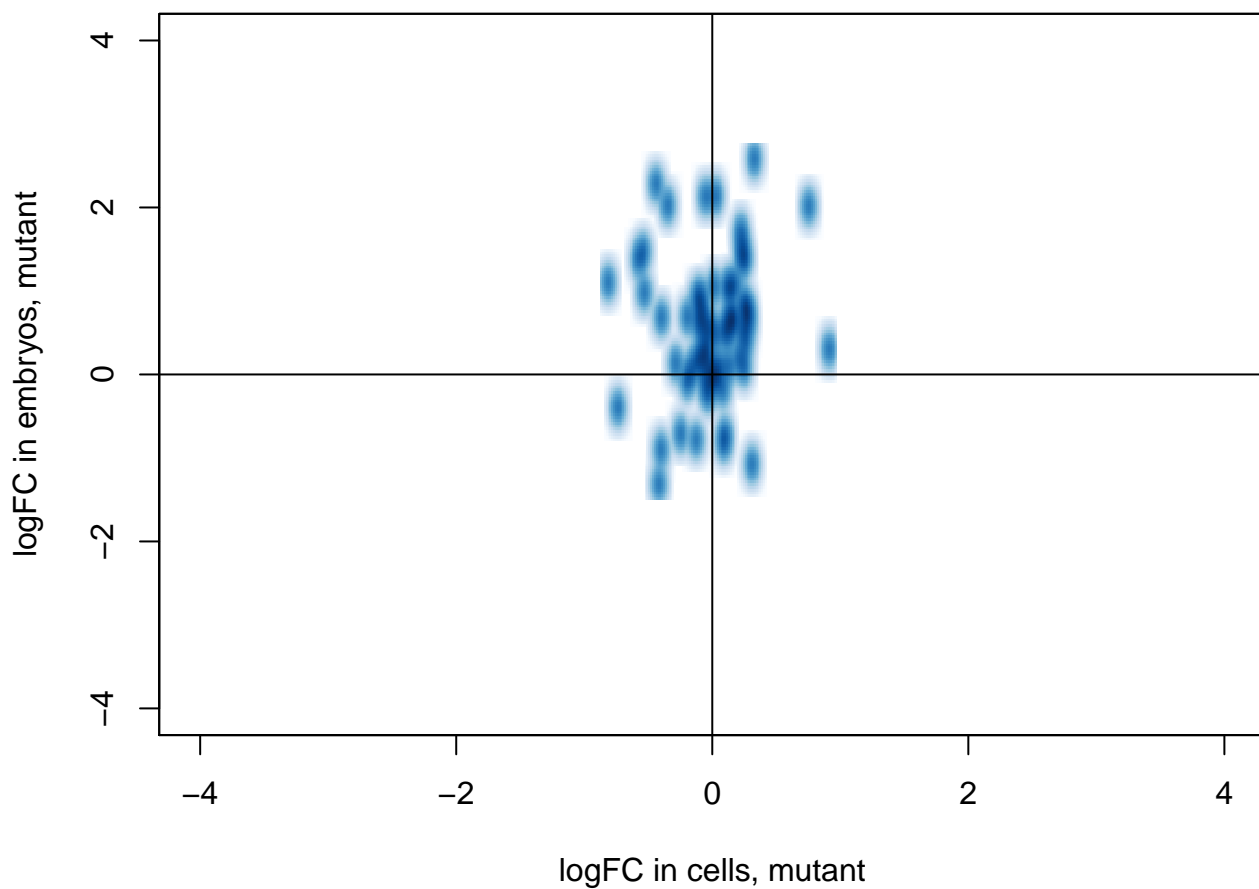

Supplement: File S1 — Archive (gzip2 compressed tar ball format) with 82 PDF files showing scatter plots for GO biological process terms with more than 50 annotated genes. Legend analogous to Figure 2. (BZ2) [file pone.0028985.s013.bz2 › scatterplots for 82 GO process terms with more than 50 genes/mushroom body development-results.pdf]

### genes in nervous system development

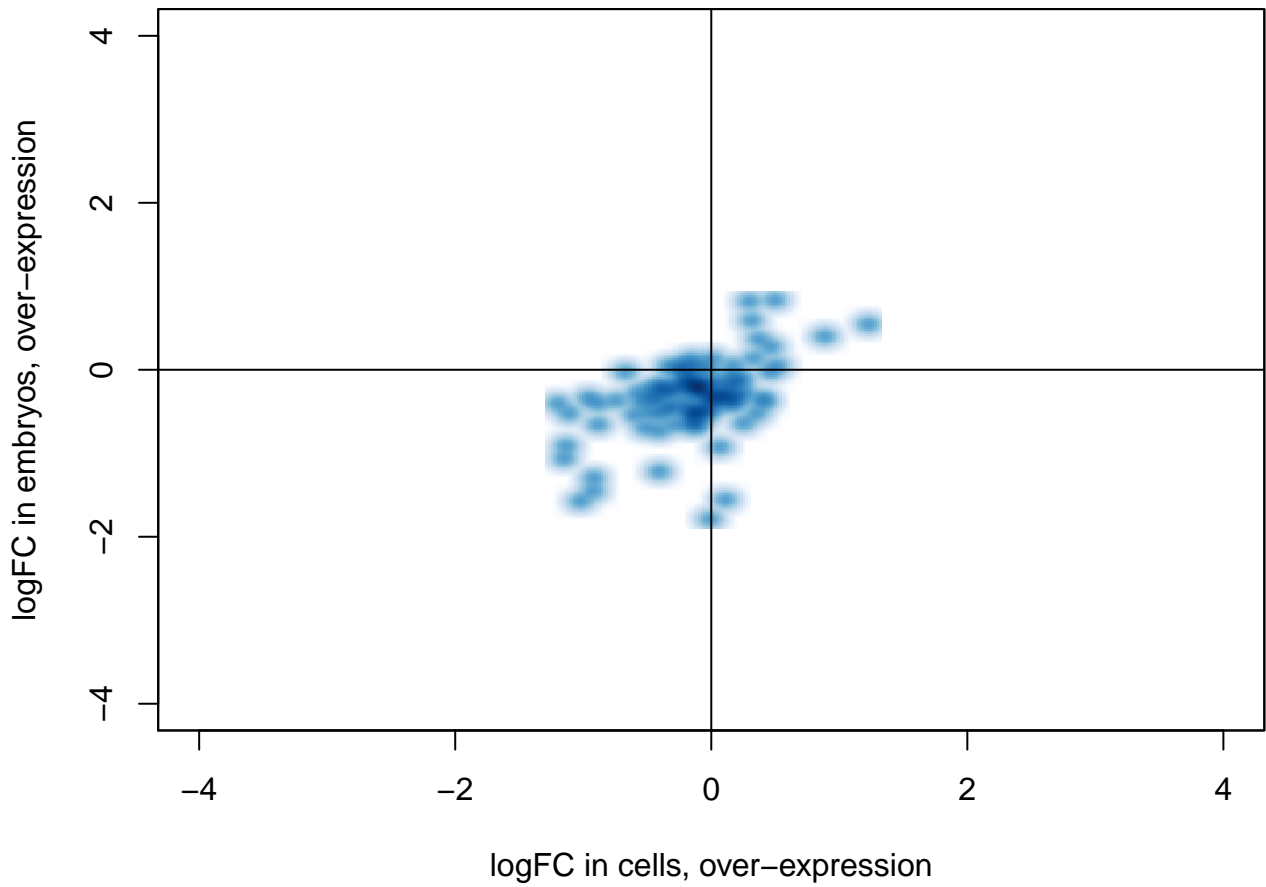

### genes in nervous system development

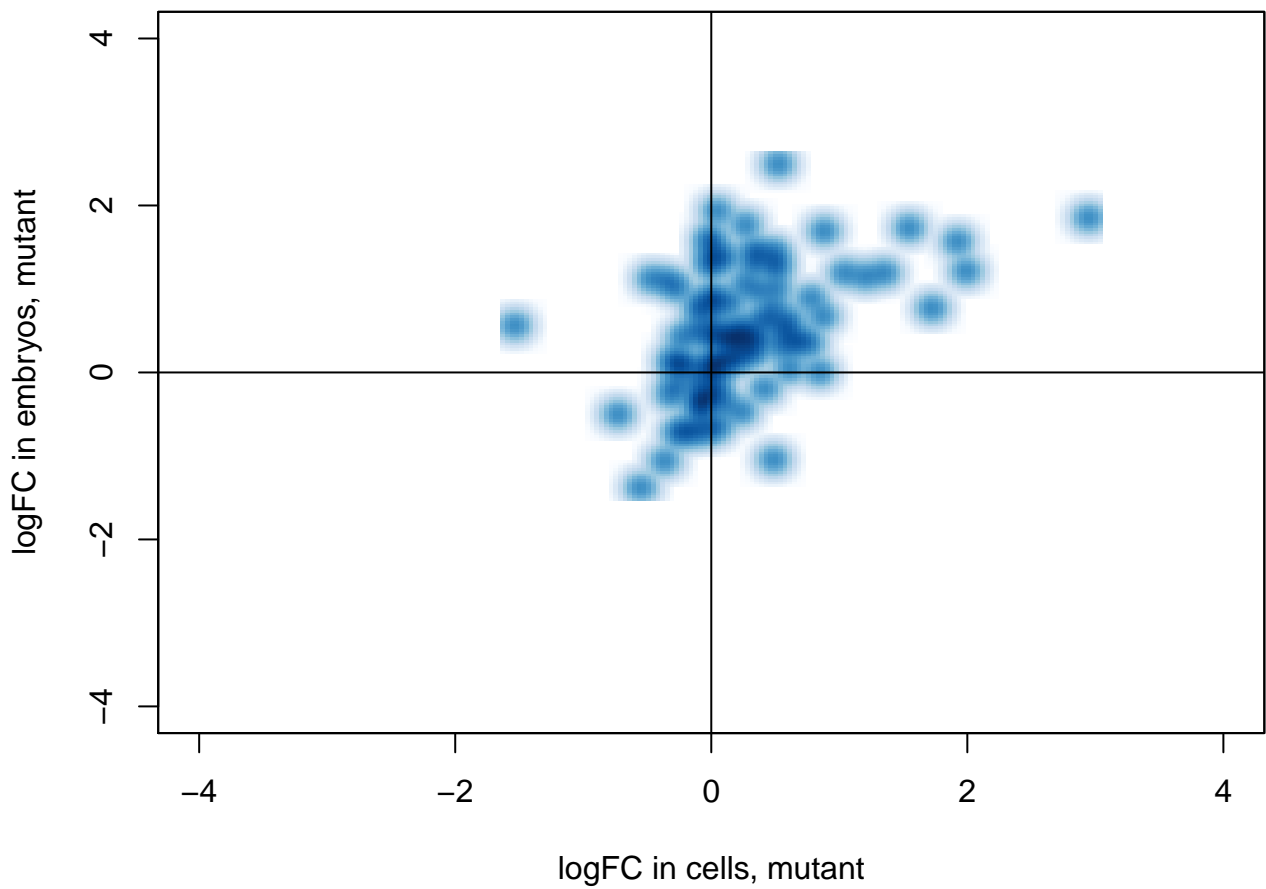

Supplement: File S1 — Archive (gzip2 compressed tar ball format) with 82 PDF files showing scatter plots for GO biological process terms with more than 50 annotated genes. Legend analogous to Figure 2. (BZ2) [file pone.0028985.s013.bz2 › scatterplots for 82 GO process terms with more than 50 genes/nervous system development-results.pdf]

### genes in neurotransmitter secretion

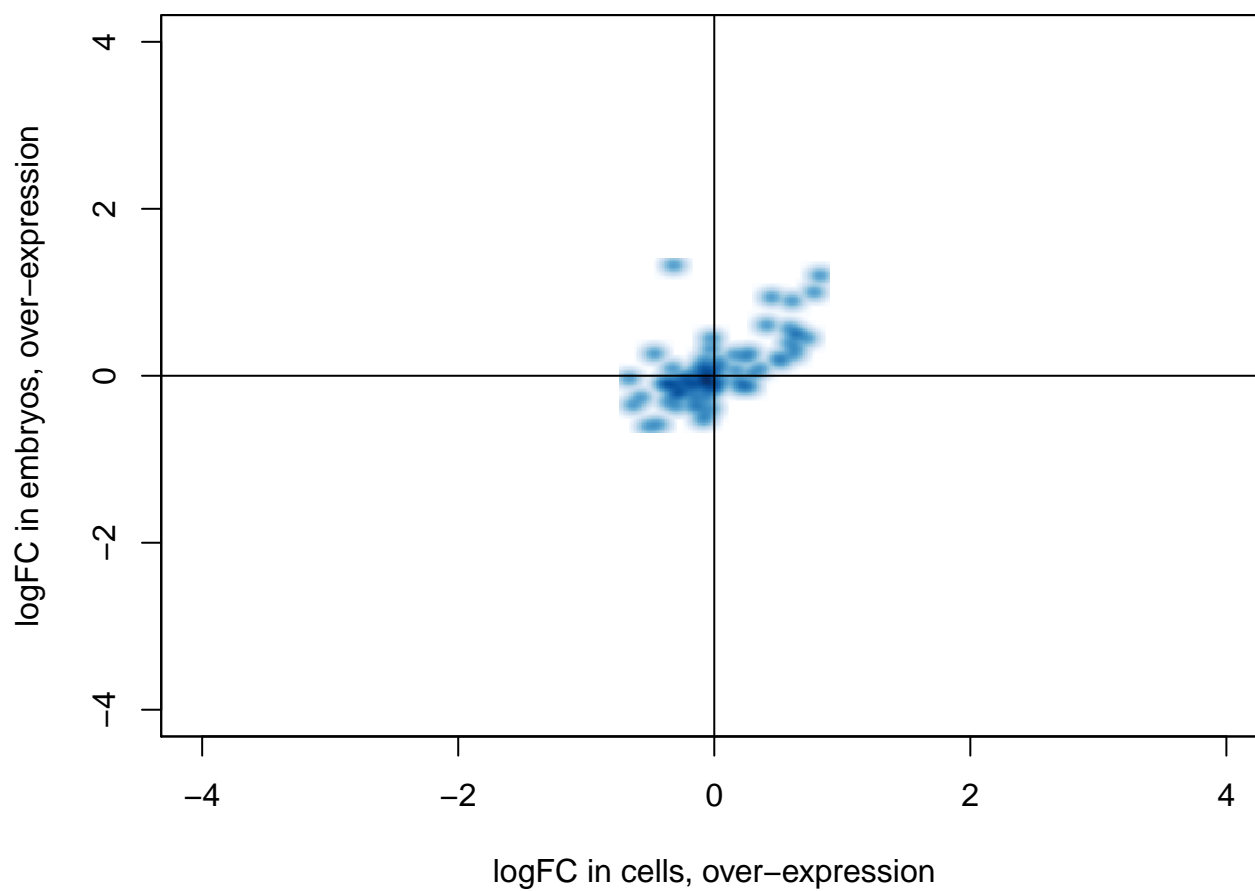

### genes in neurotransmitter secretion

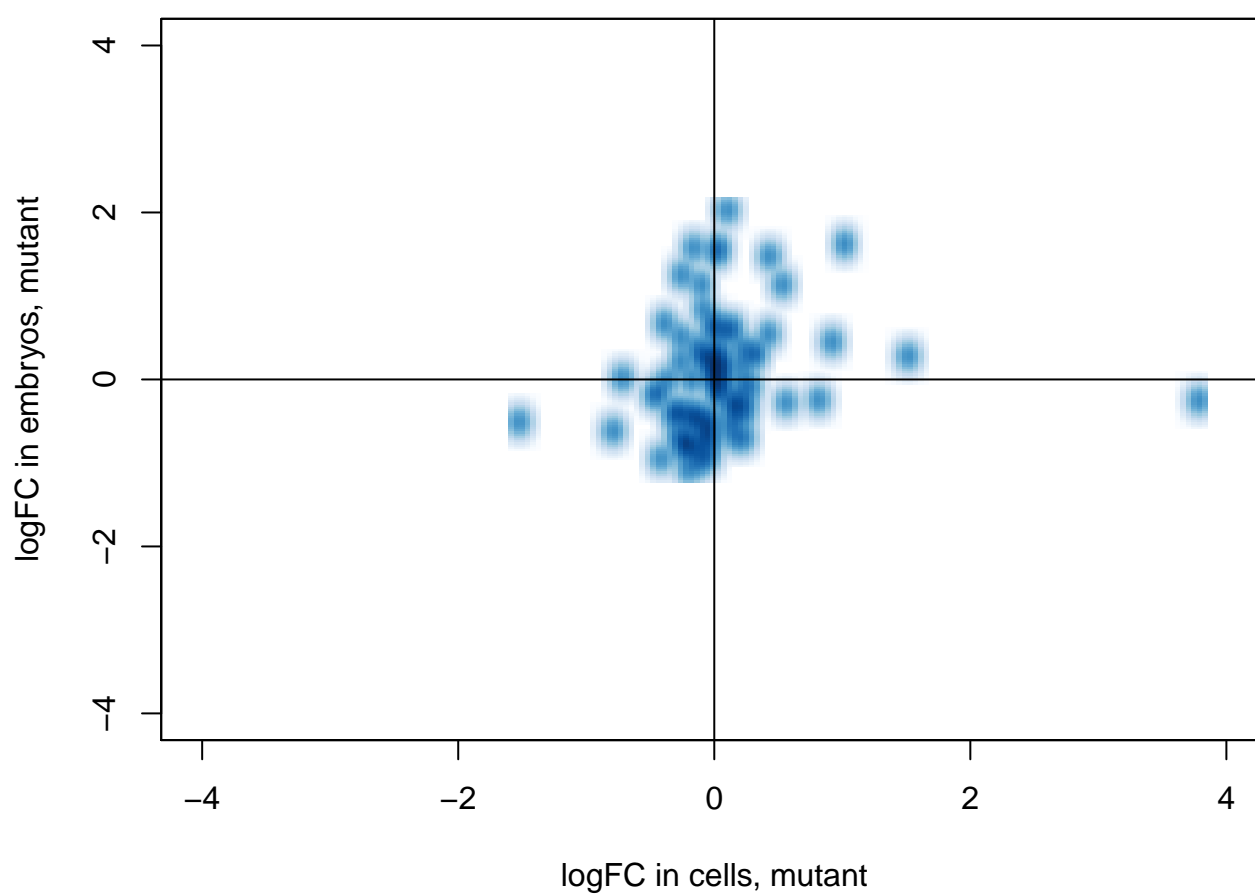

Supplement: File S1 — Archive (gzip2 compressed tar ball format) with 82 PDF files showing scatter plots for GO biological process terms with more than 50 annotated genes. Legend analogous to Figure 2. (BZ2) [file pone.0028985.s013.bz2 › scatterplots for 82 GO process terms with more than 50 genes/neurotransmitter secretion-results.pdf]

### genes in oogenesis

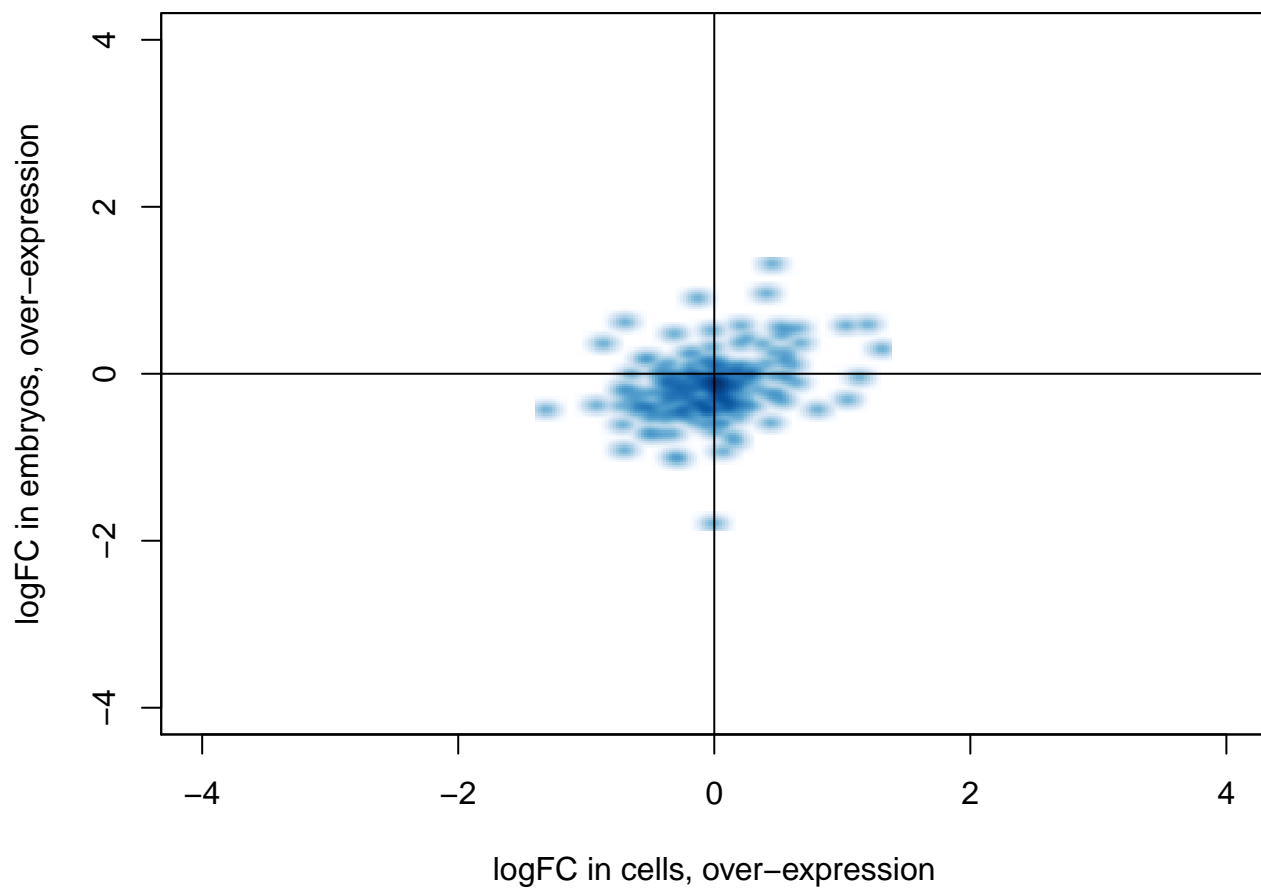

### genes in oogenesis

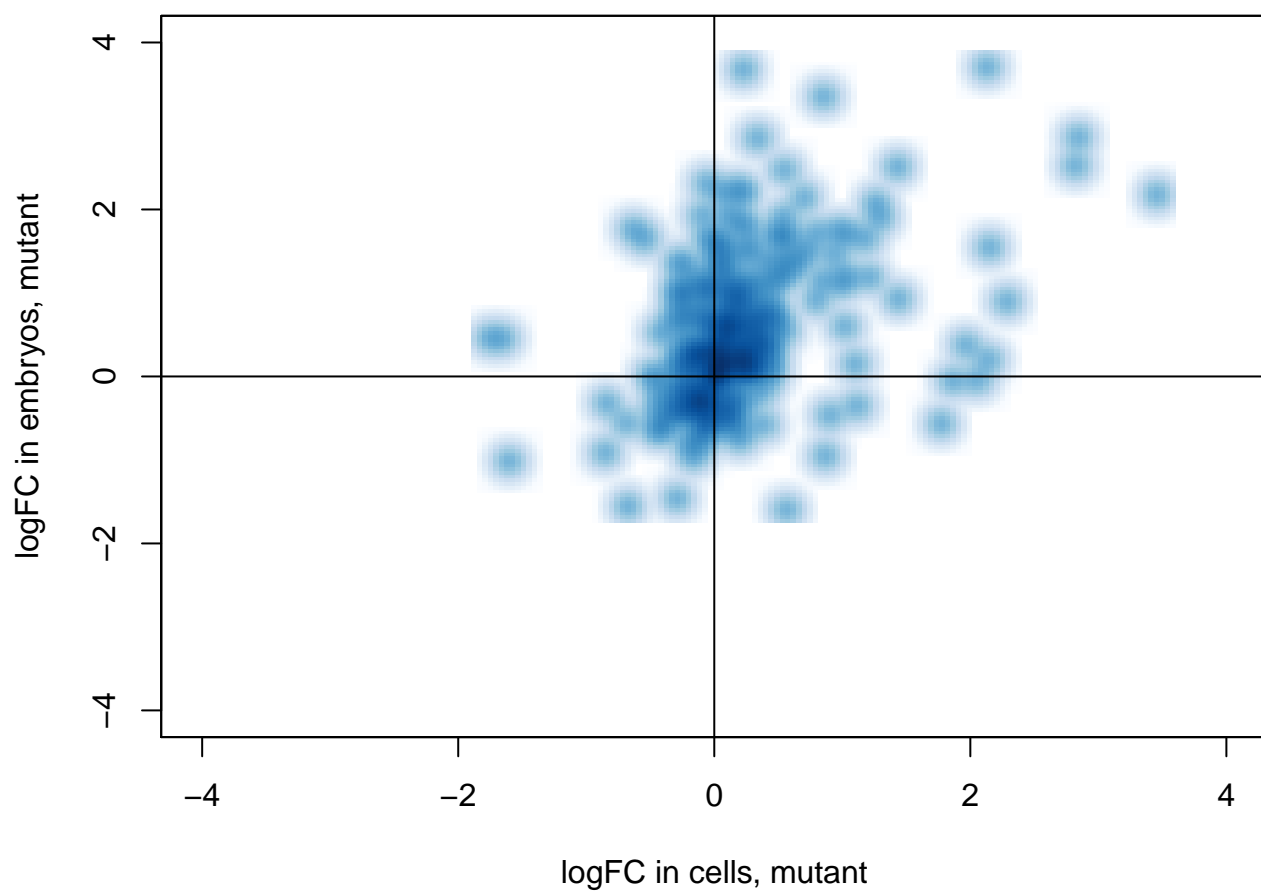

Supplement: File S1 — Archive (gzip2 compressed tar ball format) with 82 PDF files showing scatter plots for GO biological process terms with more than 50 annotated genes. Legend analogous to Figure 2. (BZ2) [file pone.0028985.s013.bz2 › scatterplots for 82 GO process terms with more than 50 genes/oogenesis-results.pdf]

### genes in open tracheal system development

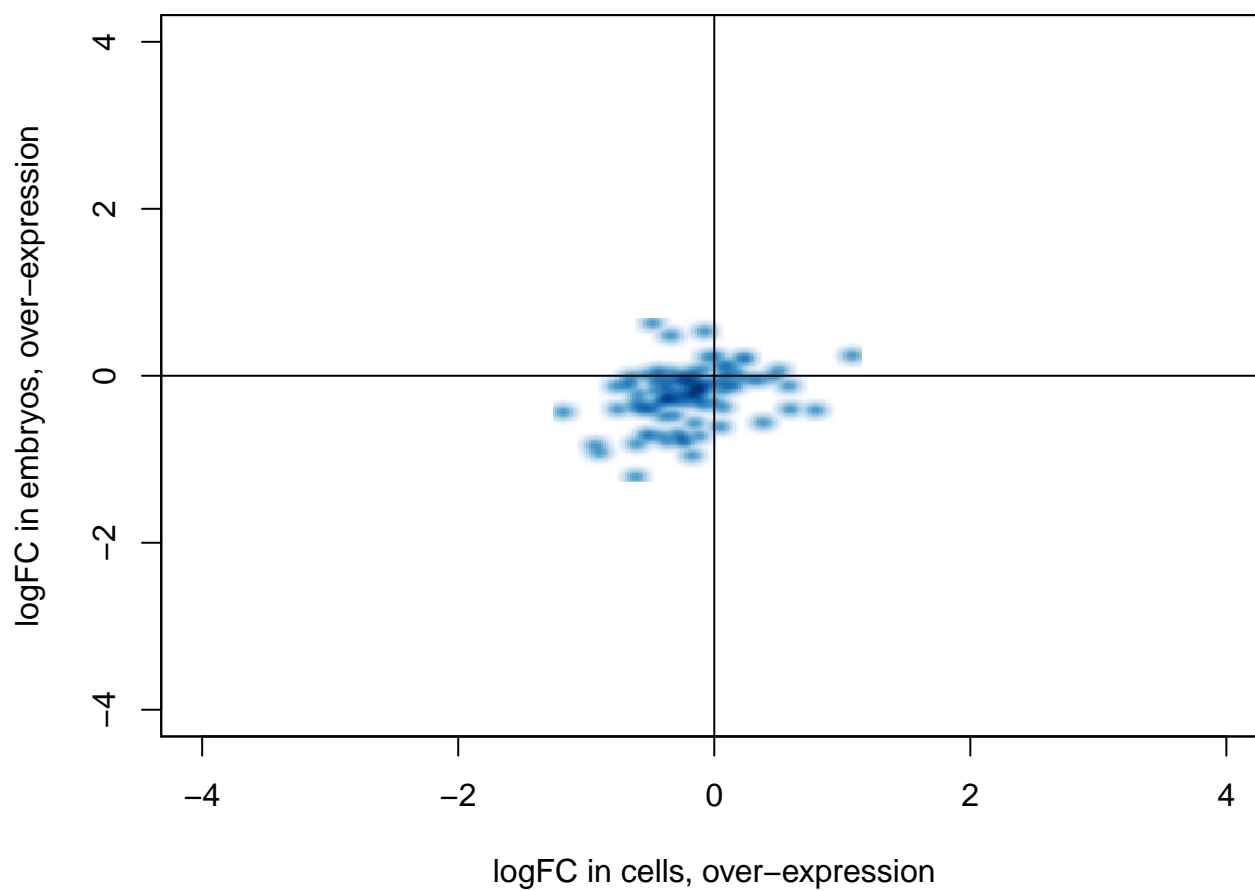

### genes in open tracheal system development

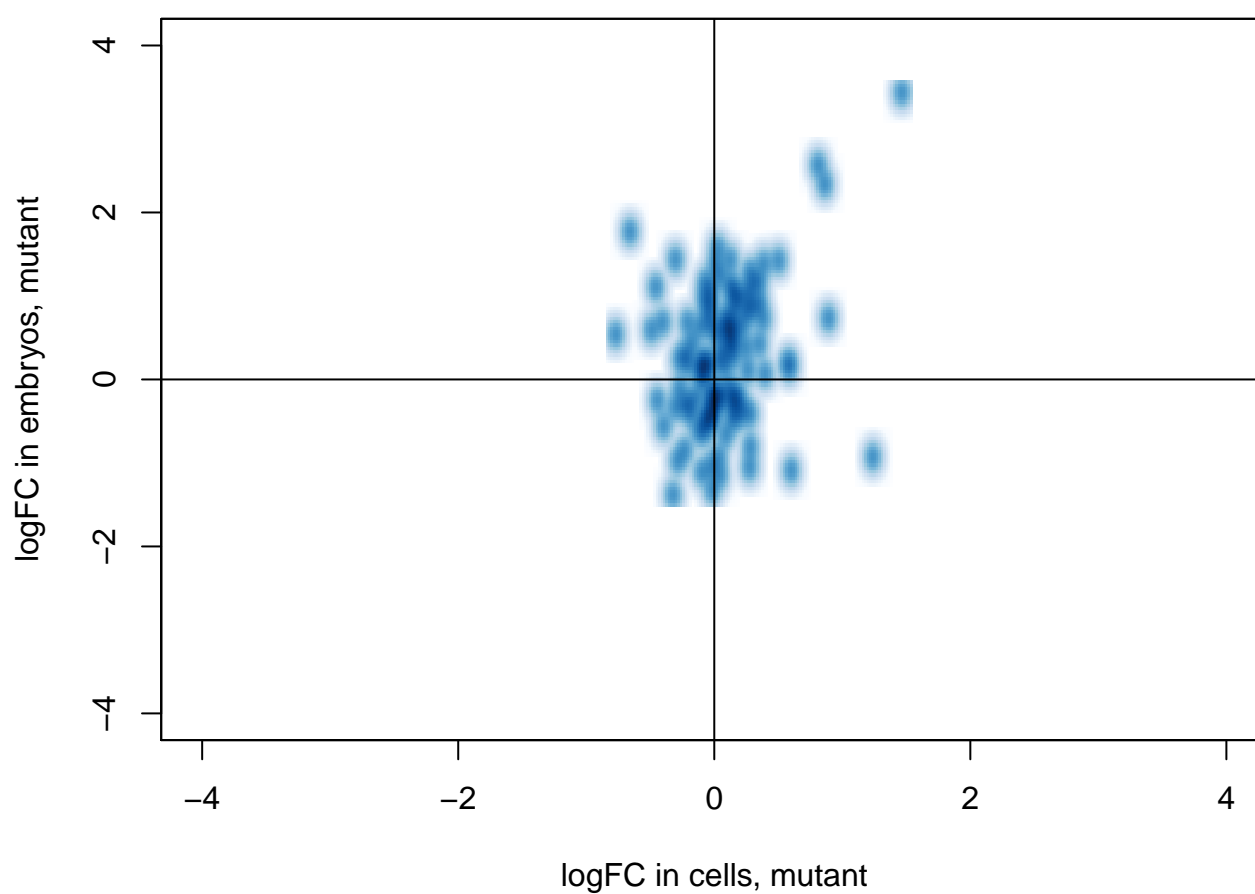

Supplement: File S1 — Archive (gzip2 compressed tar ball format) with 82 PDF files showing scatter plots for GO biological process terms with more than 50 annotated genes. Legend analogous to Figure 2. (BZ2) [file pone.0028985.s013.bz2 › scatterplots for 82 GO process terms with more than 50 genes/open tracheal system development-results.pdf]

### genes in ovarian follicle cell development

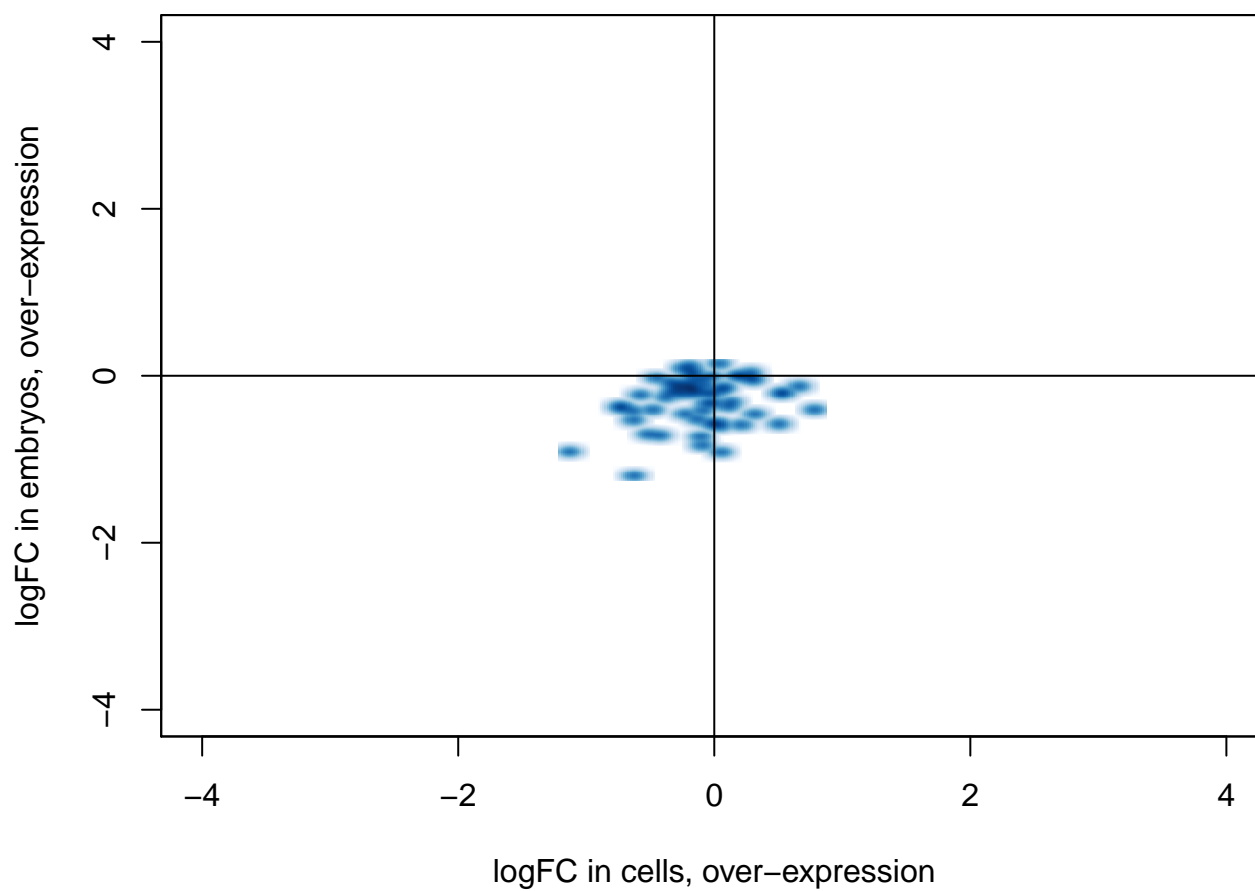

### genes in ovarian follicle cell development

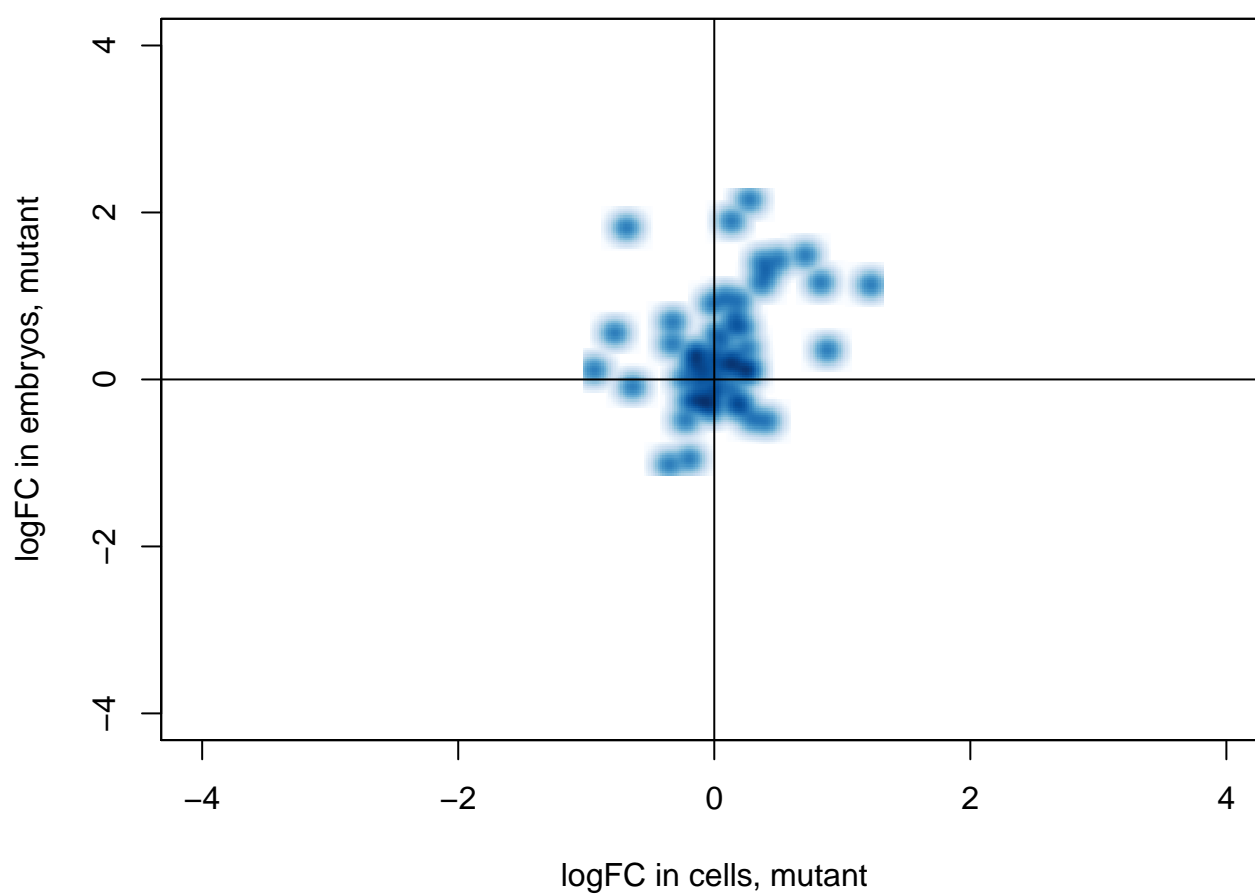

Supplement: File S1 — Archive (gzip2 compressed tar ball format) with 82 PDF files showing scatter plots for GO biological process terms with more than 50 annotated genes. Legend analogous to Figure 2. (BZ2) [file pone.0028985.s013.bz2 › scatterplots for 82 GO process terms with more than 50 genes/ovarian follicle cell development-results.pdf]

### genes in oxidation–reduction process

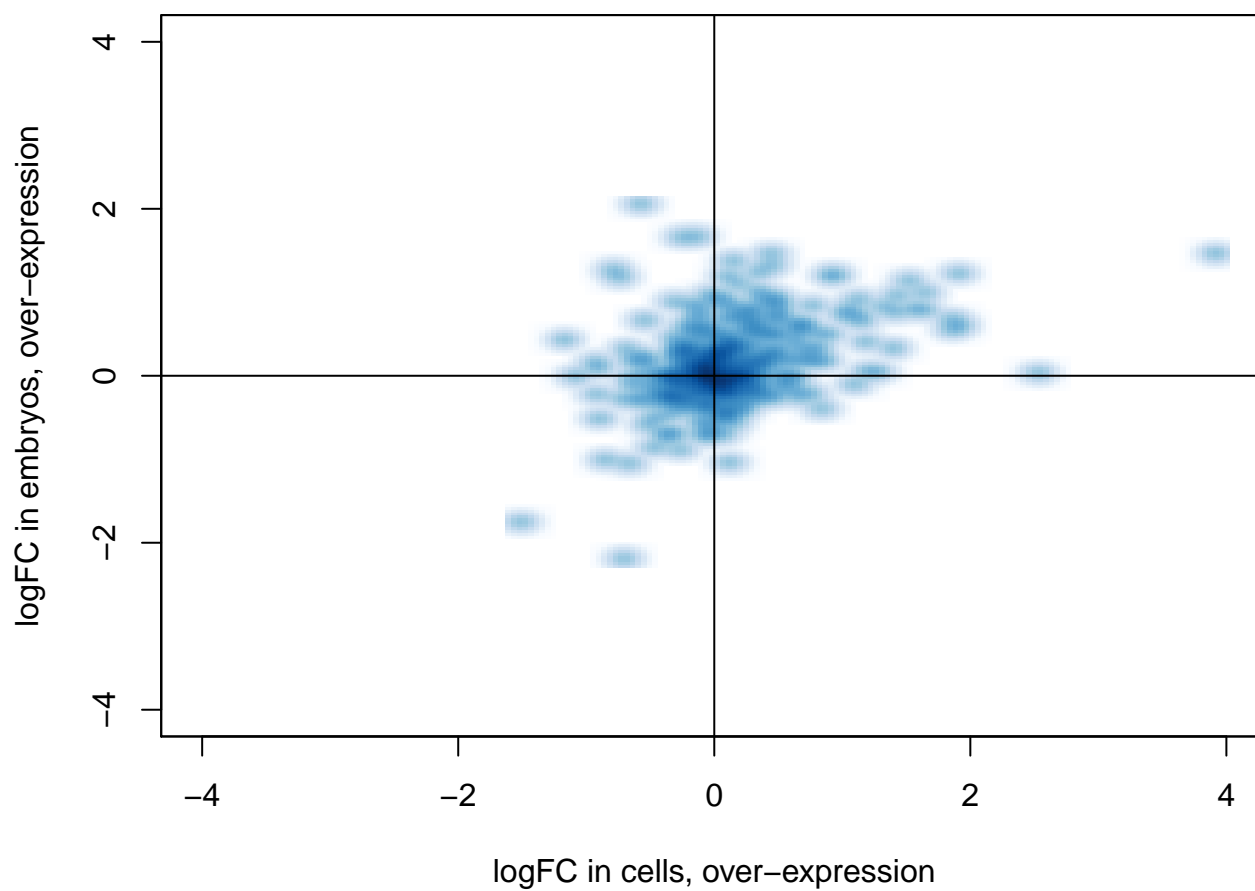

### genes in oxidation–reduction process

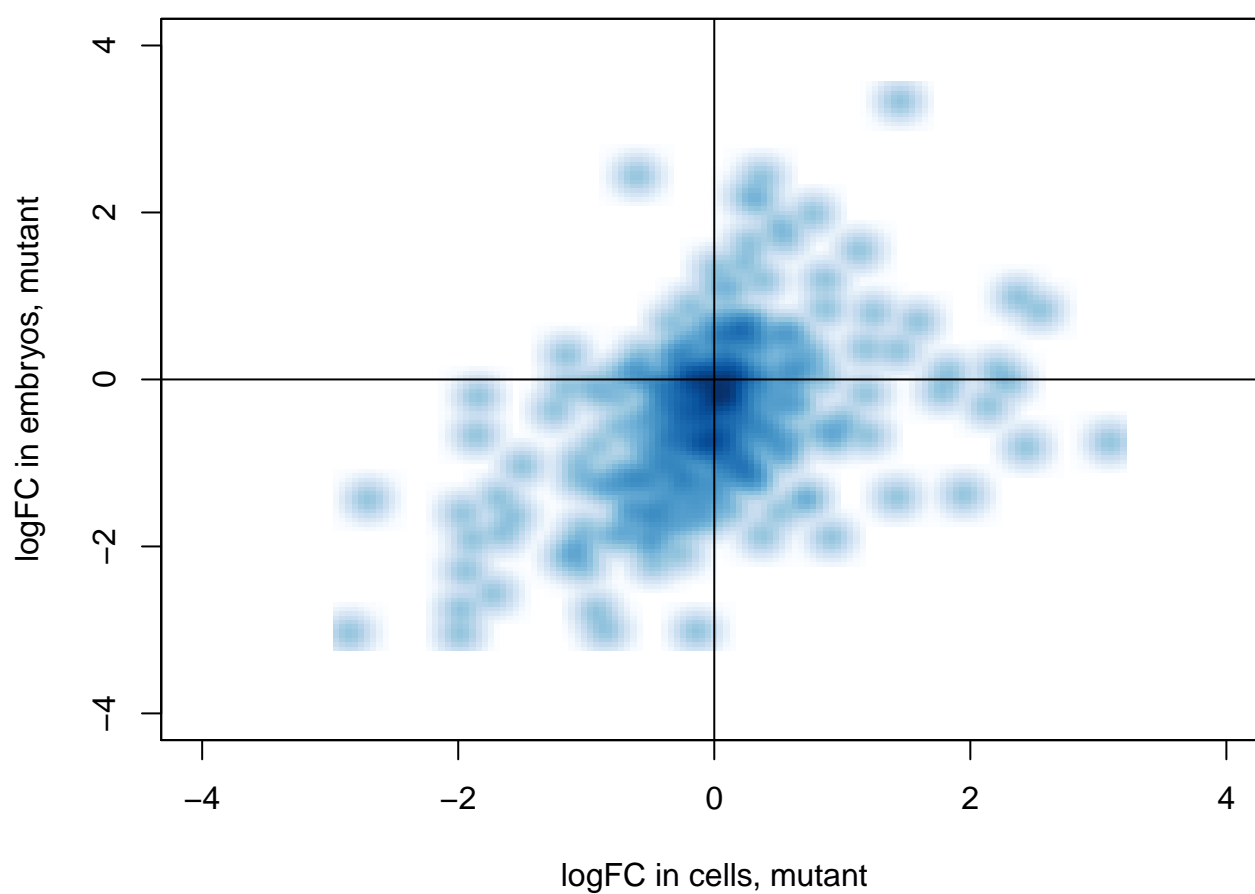

Supplement: File S1 — Archive (gzip2 compressed tar ball format) with 82 PDF files showing scatter plots for GO biological process terms with more than 50 annotated genes. Legend analogous to Figure 2. (BZ2) [file pone.0028985.s013.bz2 › scatterplots for 82 GO process terms with more than 50 genes/oxidation-reduction process-results.pdf]

### genes in phagocytosis, engulfment

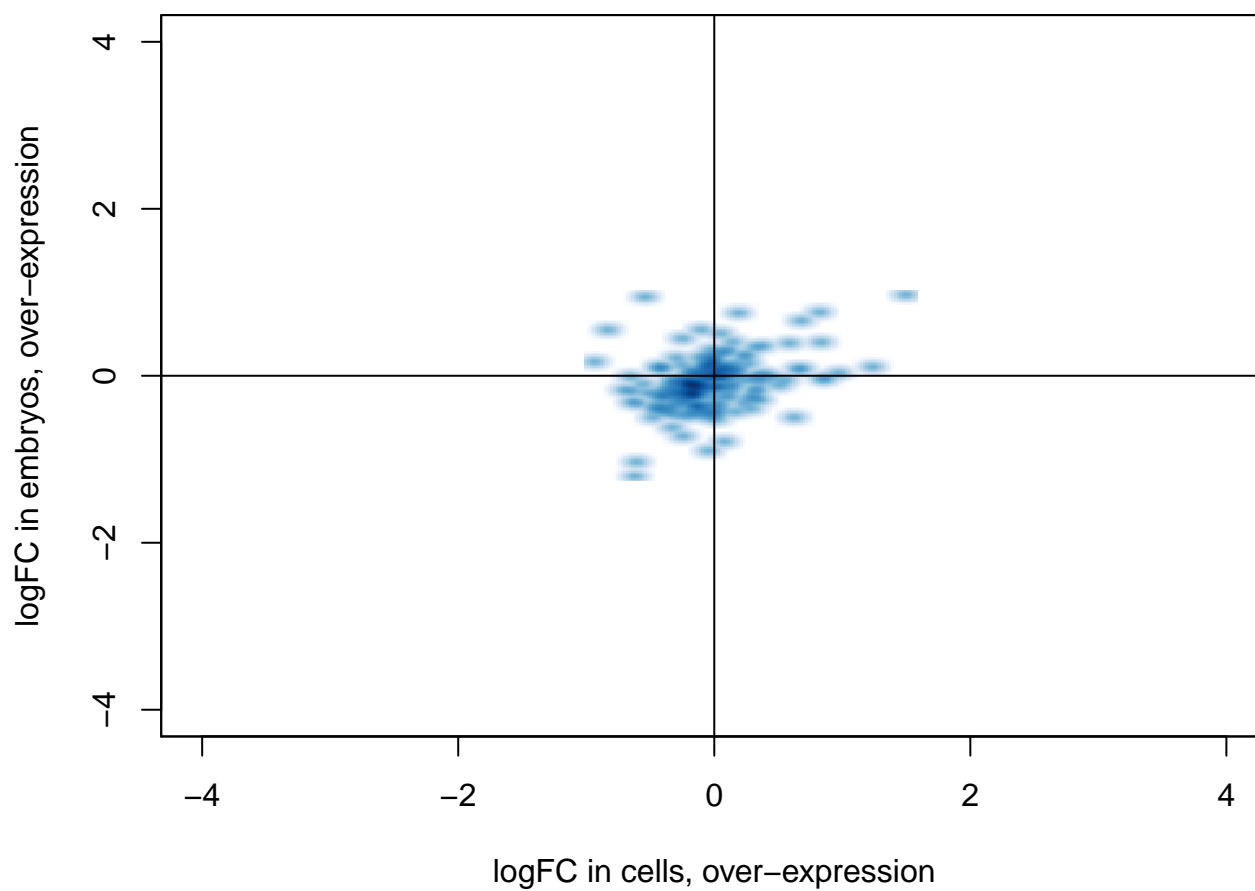

### genes in phagocytosis, engulfment

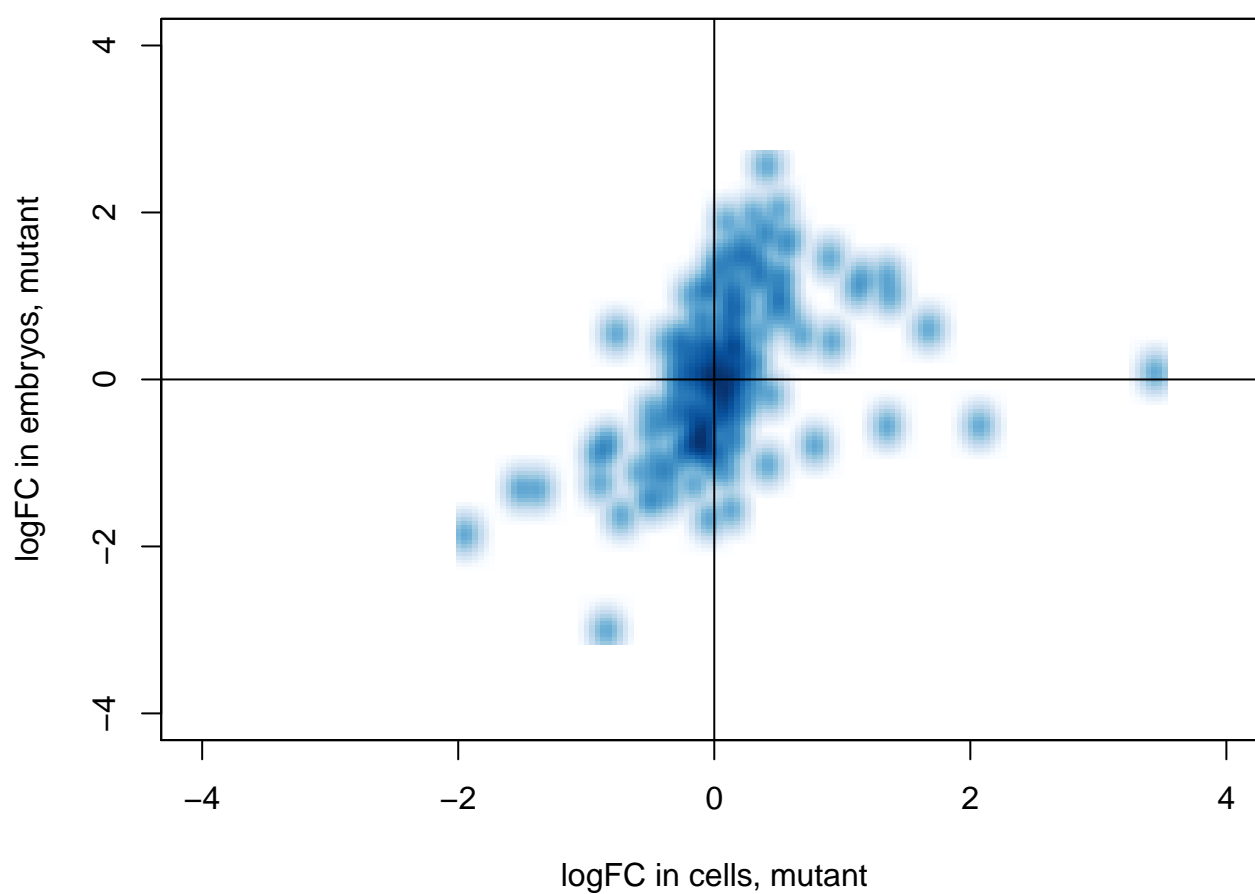

Supplement: File S1 — Archive (gzip2 compressed tar ball format) with 82 PDF files showing scatter plots for GO biological process terms with more than 50 annotated genes. Legend analogous to Figure 2. (BZ2) [file pone.0028985.s013.bz2 › scatterplots for 82 GO process terms with more than 50 genes/phagocytosis, engulfment-results.pdf]

### genes in protein dephosphorylation

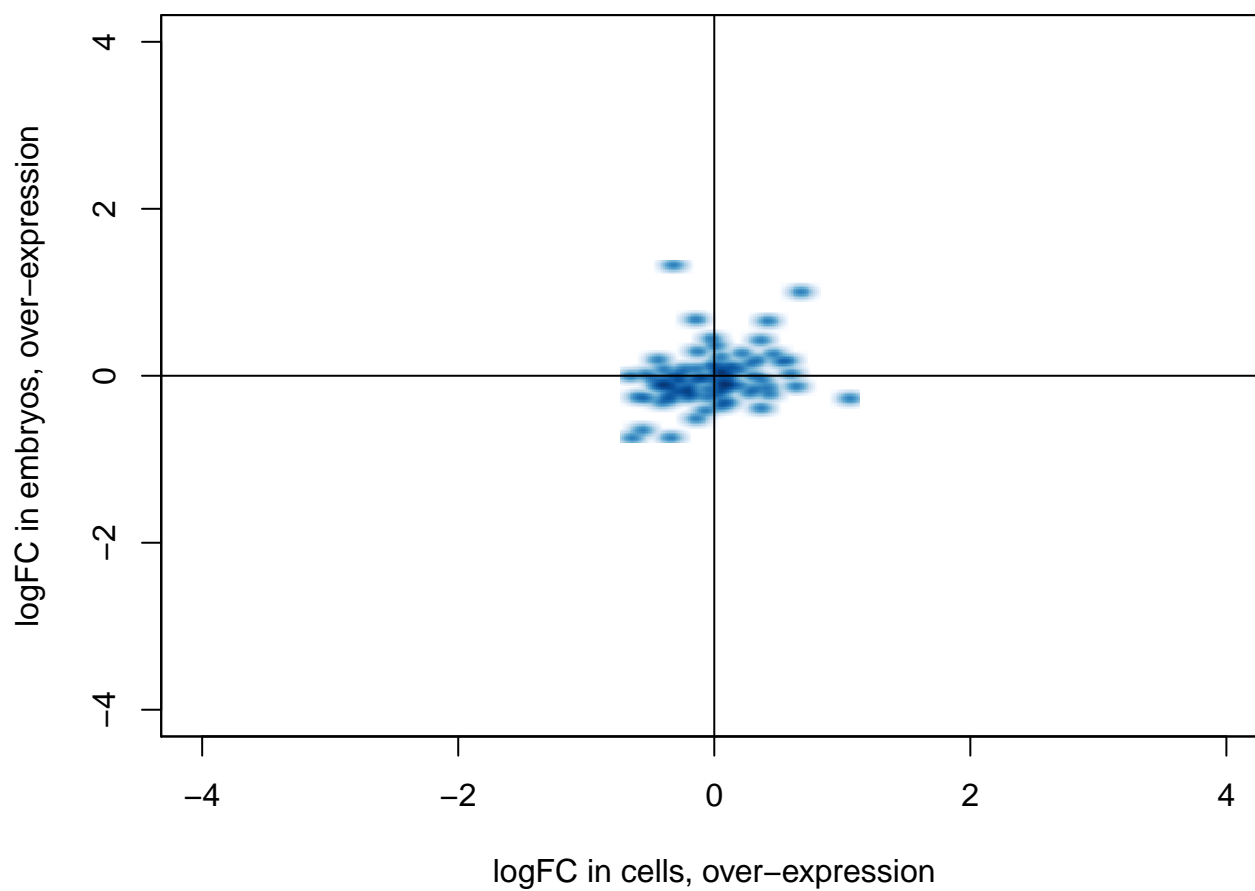

### genes in protein dephosphorylation

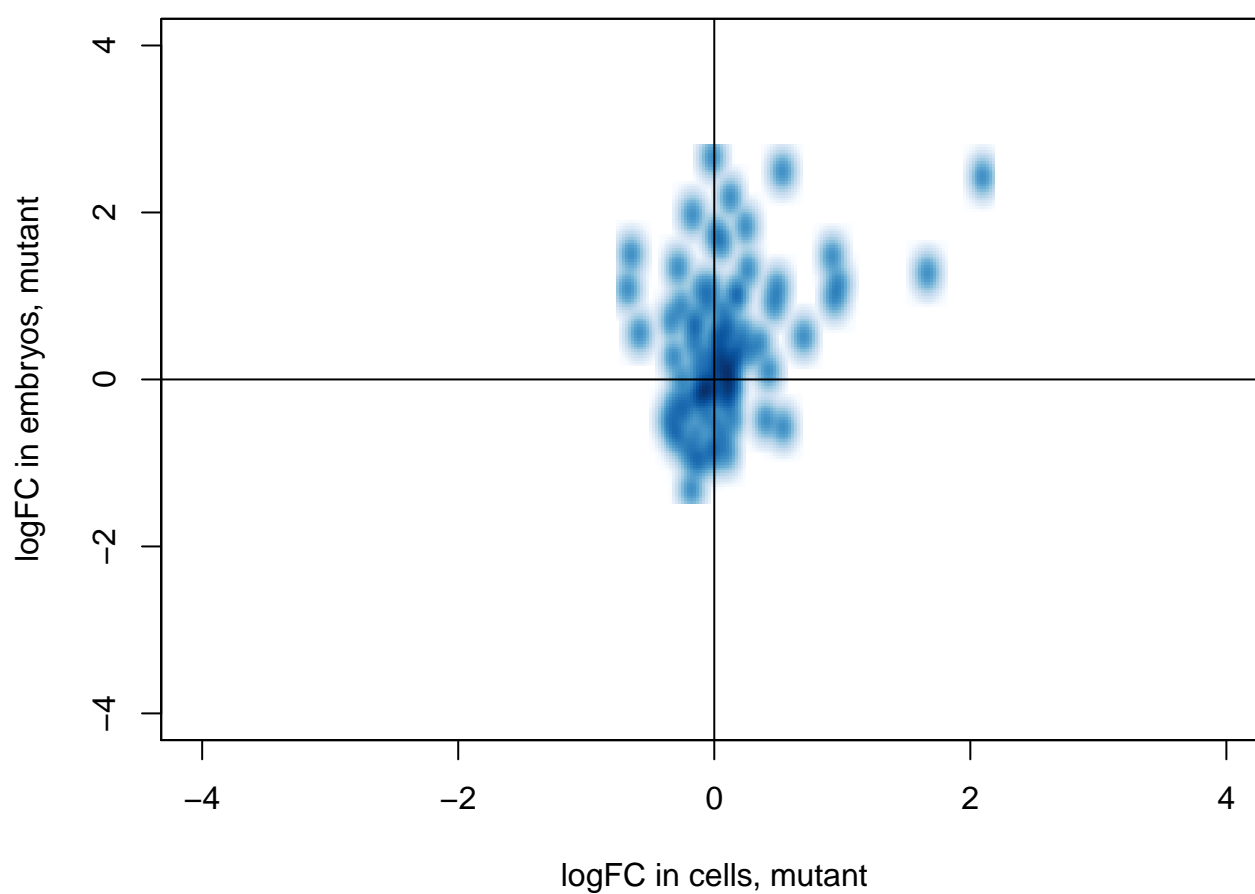

Supplement: File S1 — Archive (gzip2 compressed tar ball format) with 82 PDF files showing scatter plots for GO biological process terms with more than 50 annotated genes. Legend analogous to Figure 2. (BZ2) [file pone.0028985.s013.bz2 › scatterplots for 82 GO process terms with more than 50 genes/protein dephosphorylation-results.pdf]

### genes in protein folding

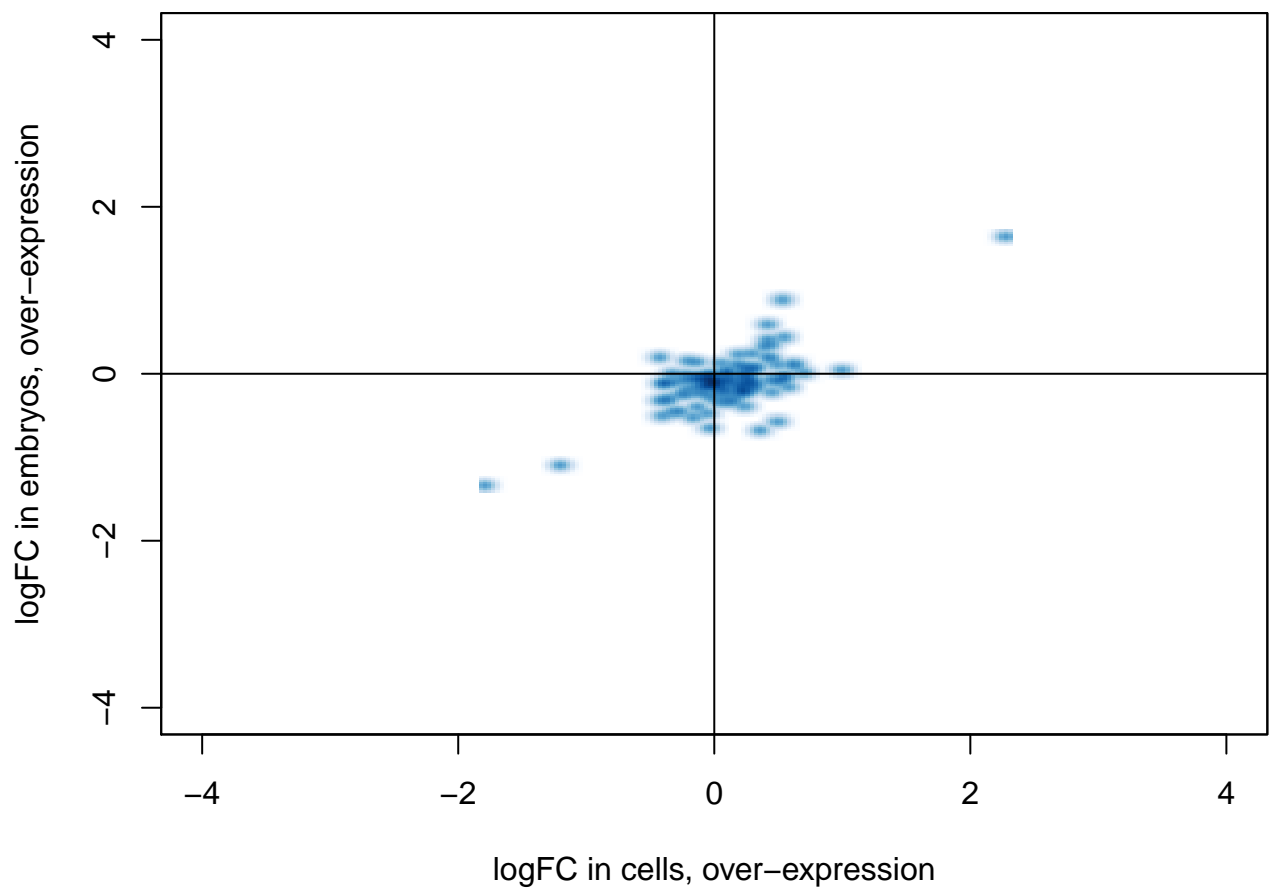

### genes in protein folding

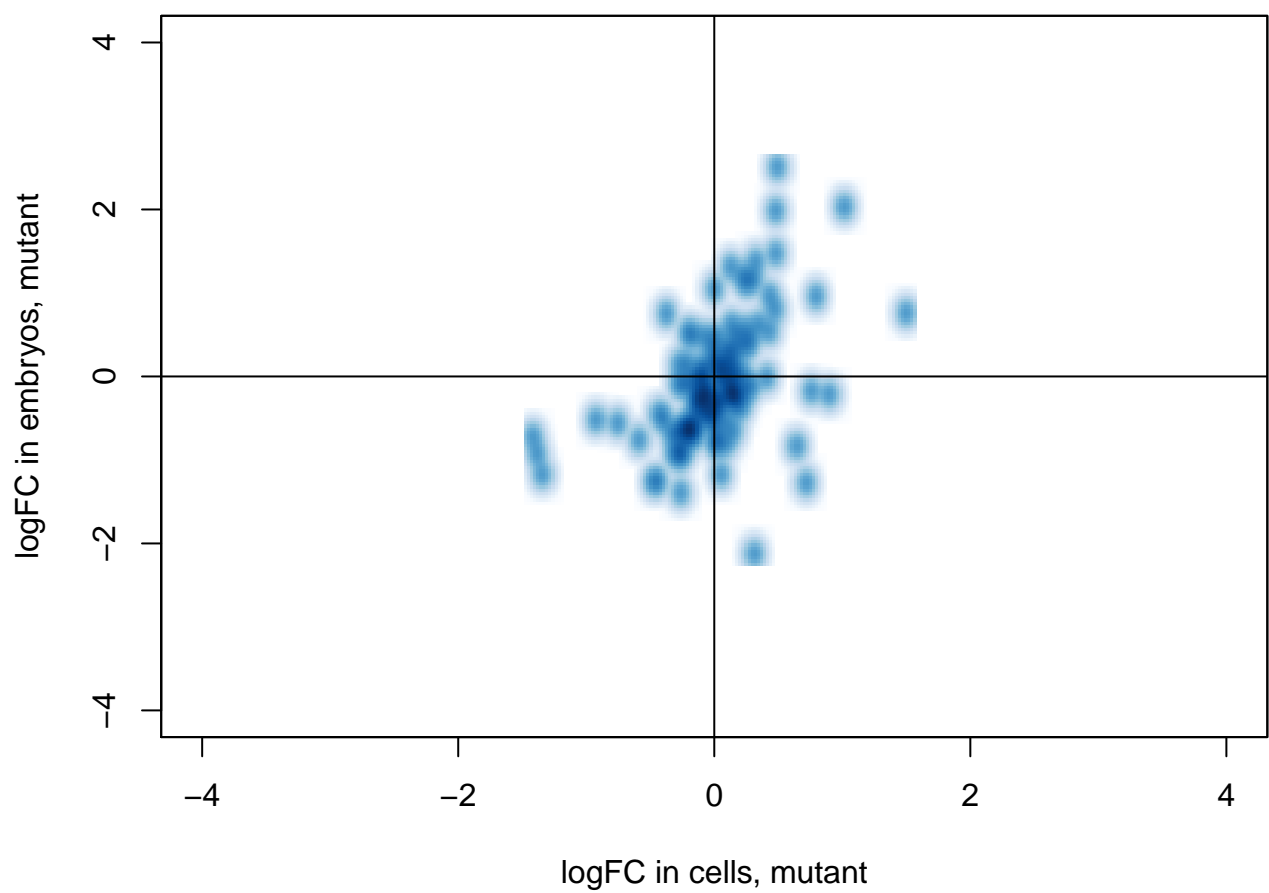

Supplement: File S1 — Archive (gzip2 compressed tar ball format) with 82 PDF files showing scatter plots for GO biological process terms with more than 50 annotated genes. Legend analogous to Figure 2. (BZ2) [file pone.0028985.s013.bz2 › scatterplots for 82 GO process terms with more than 50 genes/protein folding-results.pdf]

**genes in protein localization**

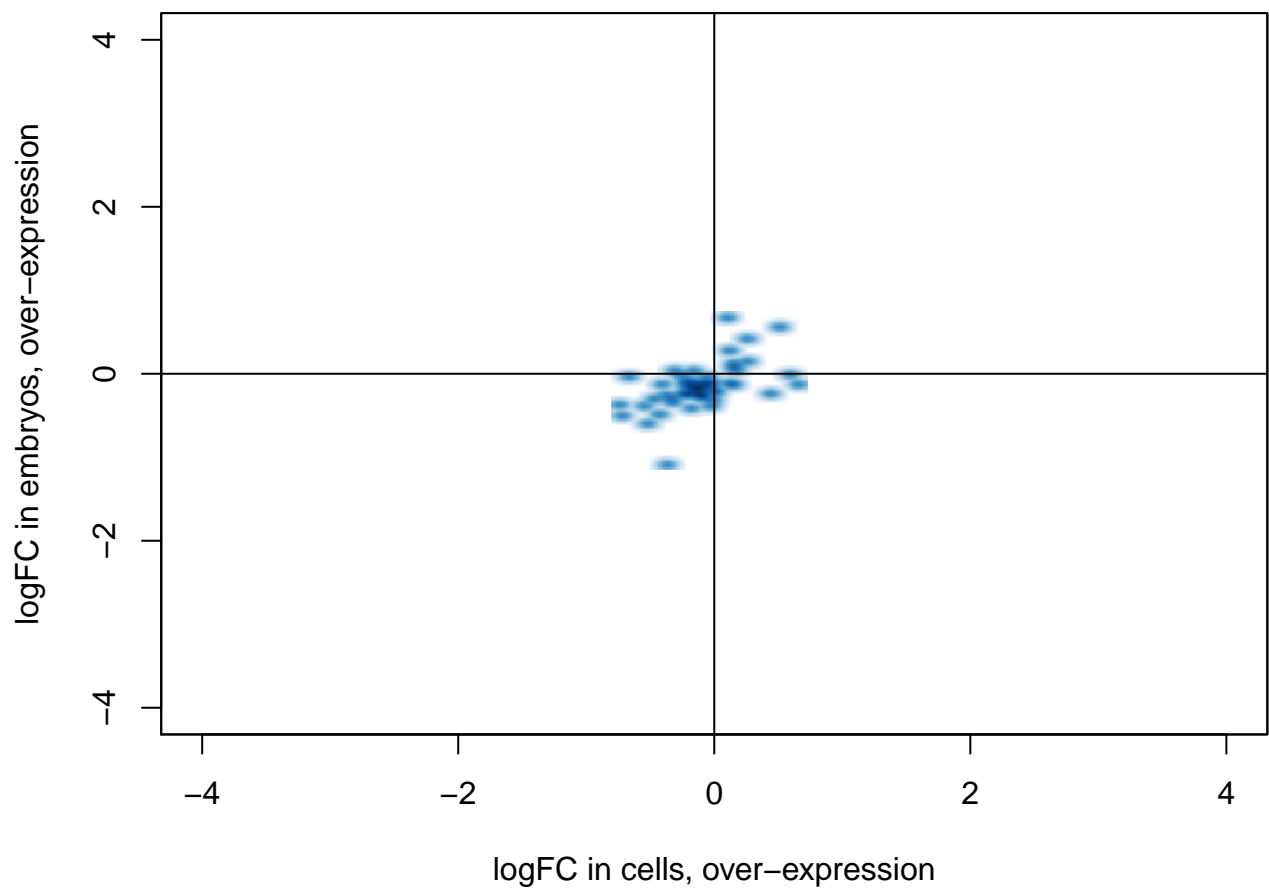

**genes in protein localization**

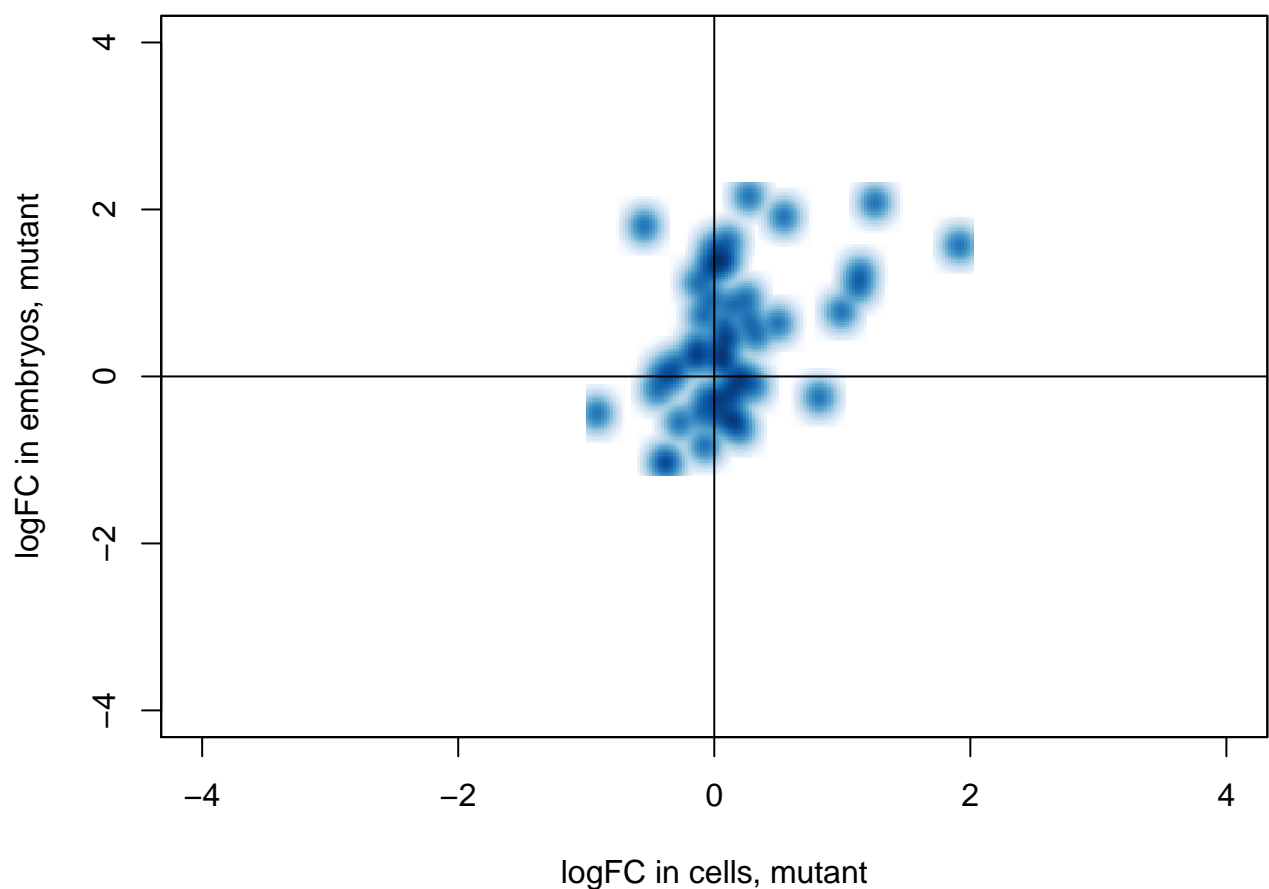

Supplement: File S1 — Archive (gzip2 compressed tar ball format) with 82 PDF files showing scatter plots for GO biological process terms with more than 50 annotated genes. Legend analogous to Figure 2. (BZ2) [file pone.0028985.s013.bz2 › scatterplots for 82 GO process terms with more than 50 genes/protein localization-results.pdf]

### genes in protein phosphorylation

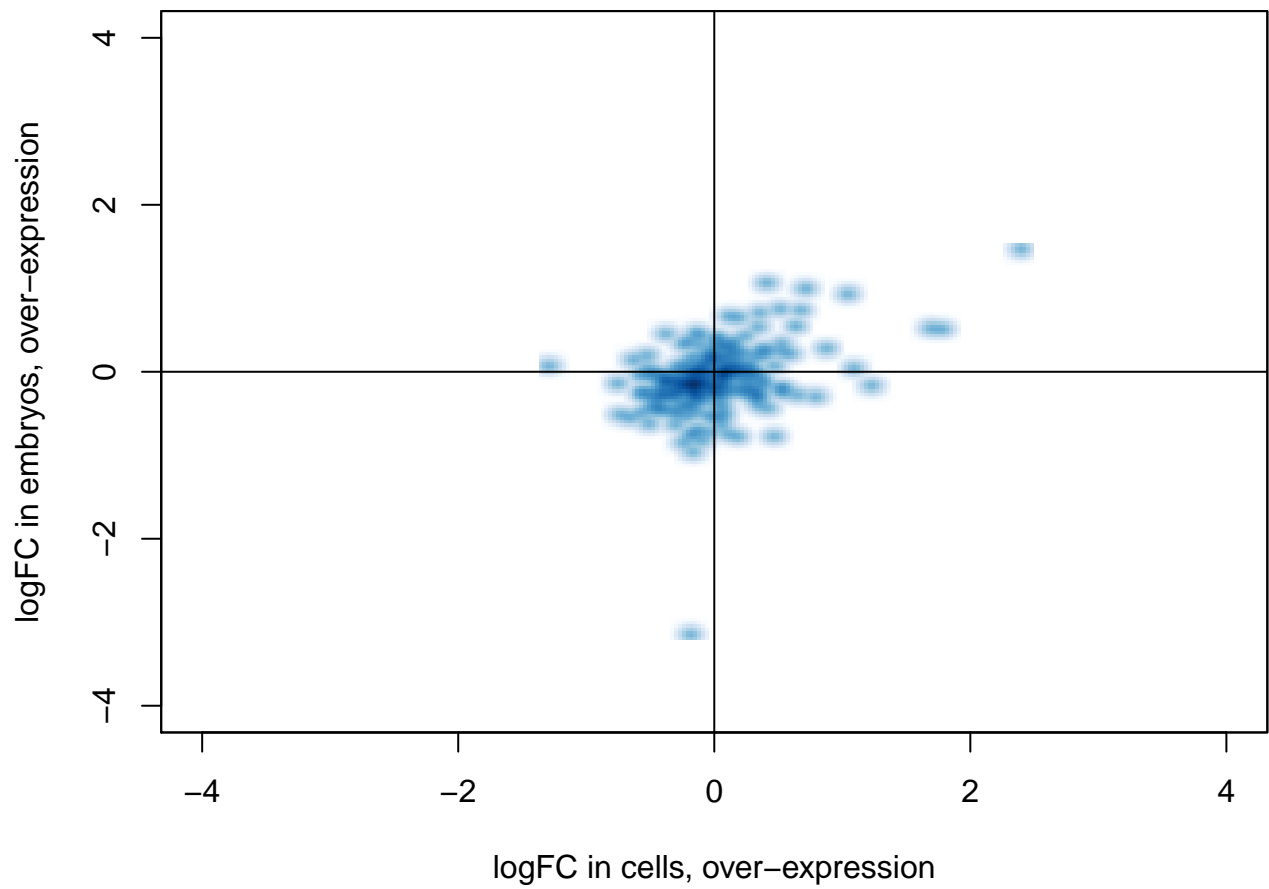

### genes in protein phosphorylation

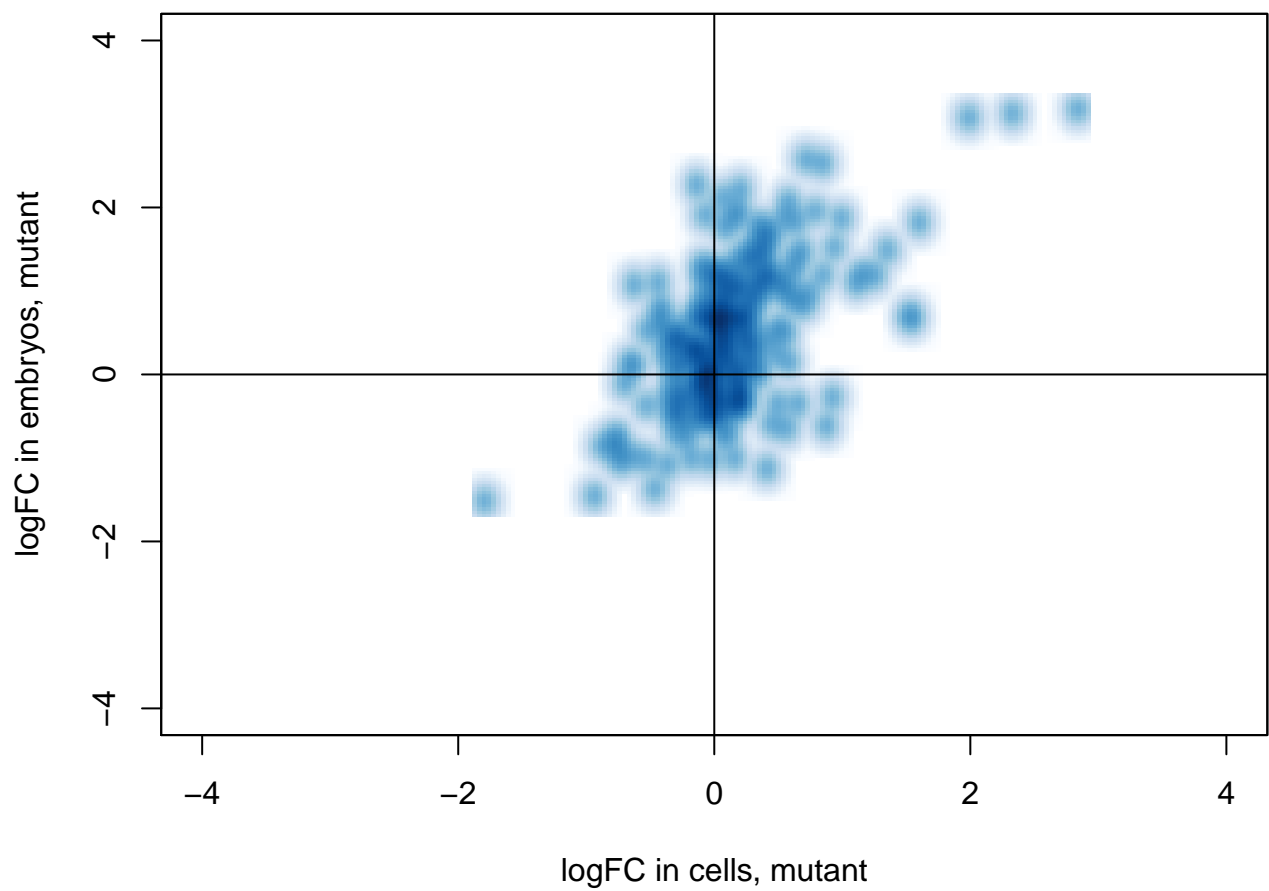

Supplement: File S1 — Archive (gzip2 compressed tar ball format) with 82 PDF files showing scatter plots for GO biological process terms with more than 50 annotated genes. Legend analogous to Figure 2. (BZ2) [file pone.0028985.s013.bz2 › scatterplots for 82 GO process terms with more than 50 genes/protein phosphorylation-results.pdf]

### genes in protein transport

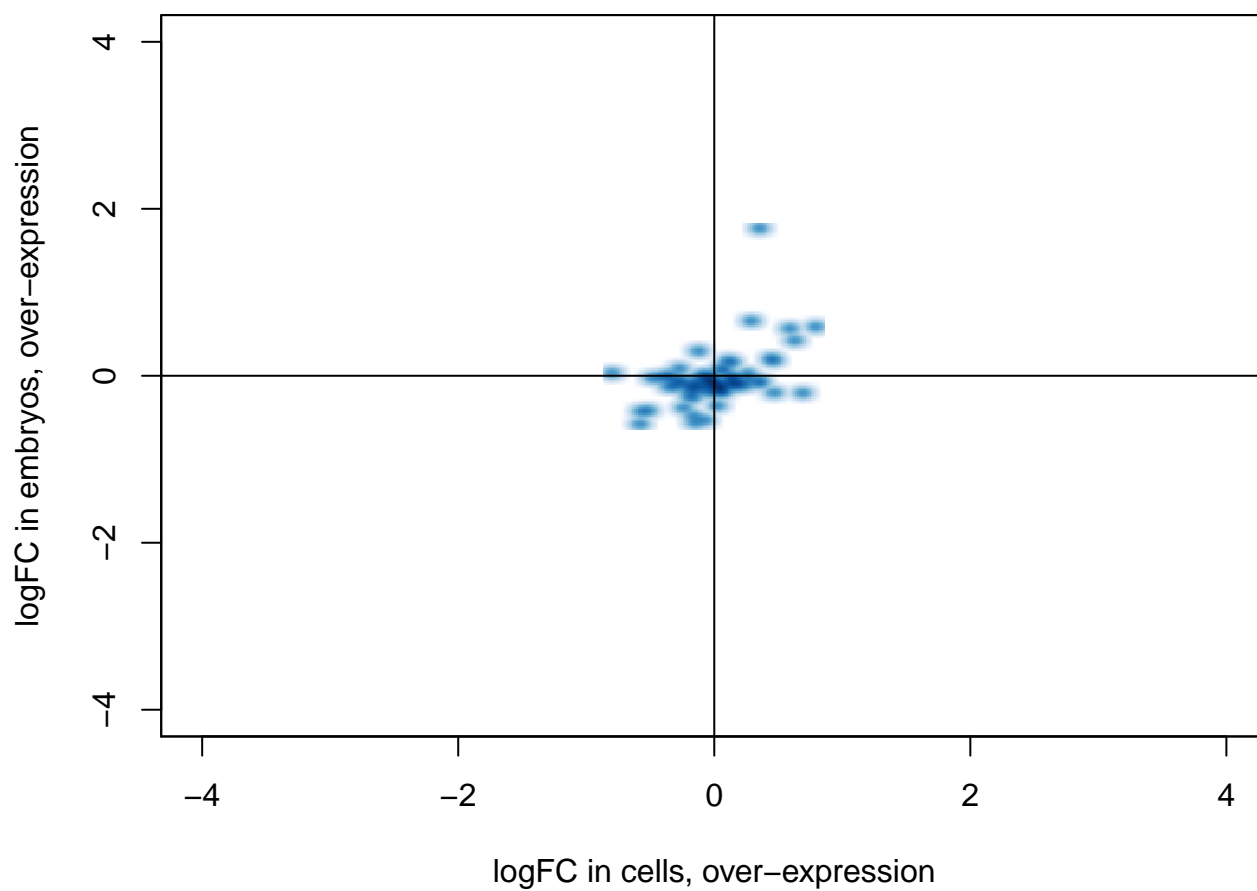

### genes in protein transport

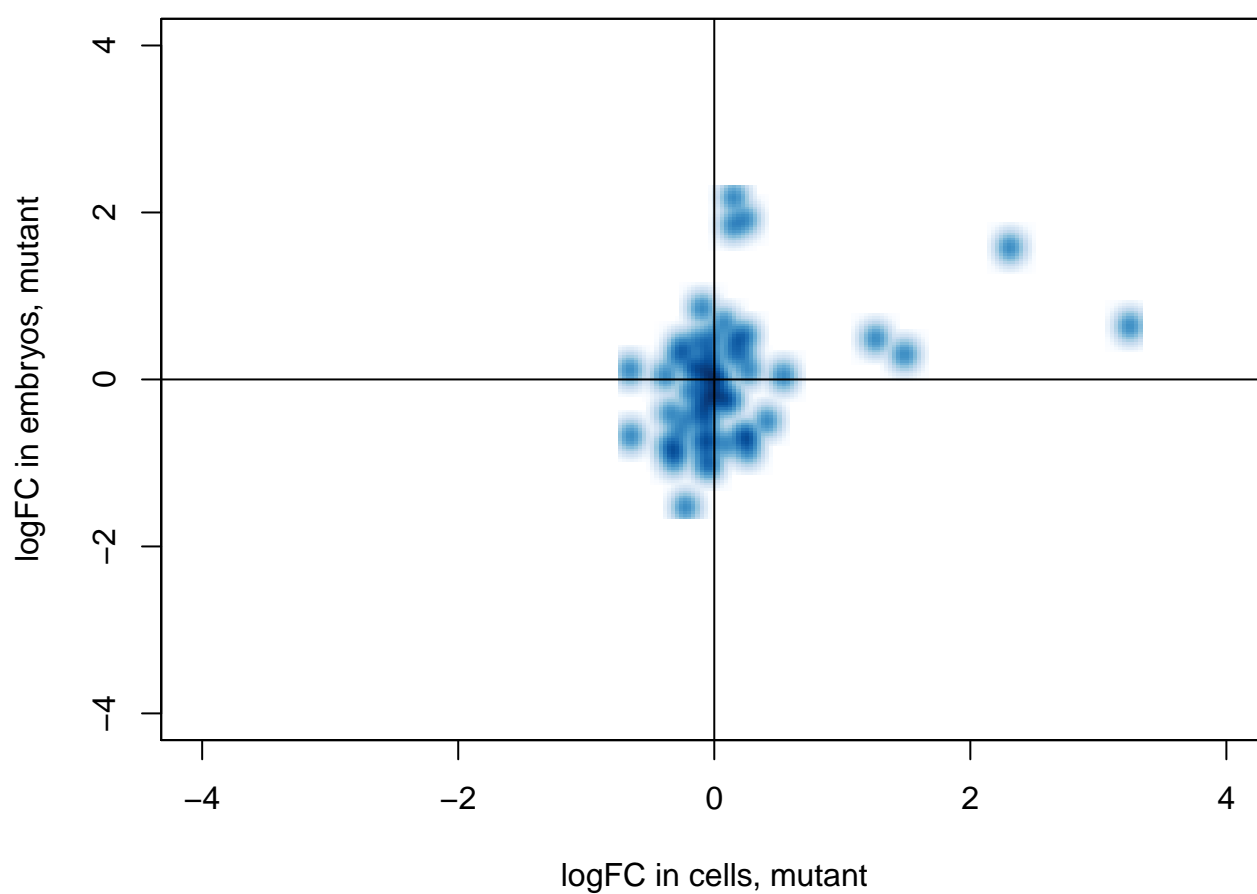

Supplement: File S1 — Archive (gzip2 compressed tar ball format) with 82 PDF files showing scatter plots for GO biological process terms with more than 50 annotated genes. Legend analogous to Figure 2. (BZ2) [file pone.0028985.s013.bz2 › scatterplots for 82 GO process terms with more than 50 genes/protein transport-results.pdf]

### genes in proteolysis

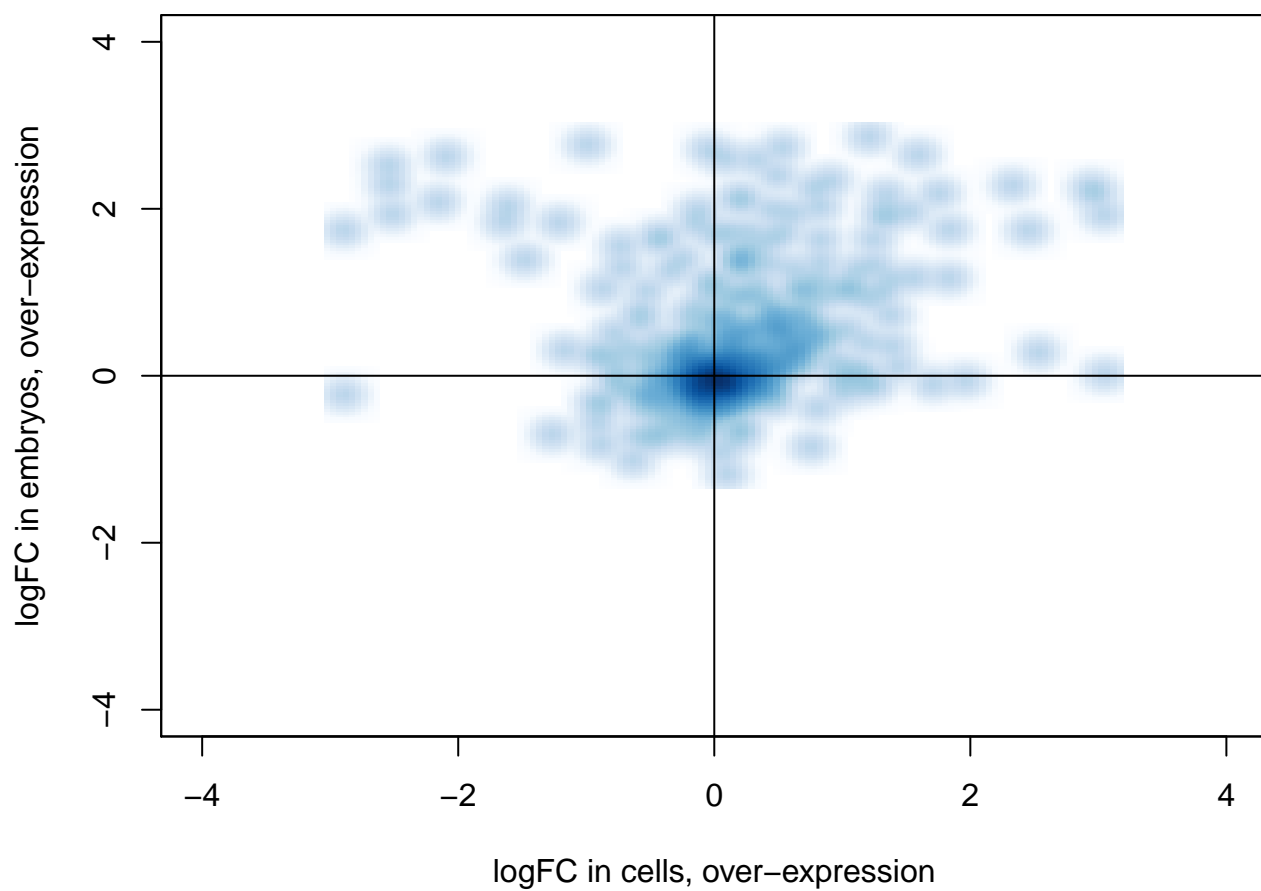

### genes in proteolysis

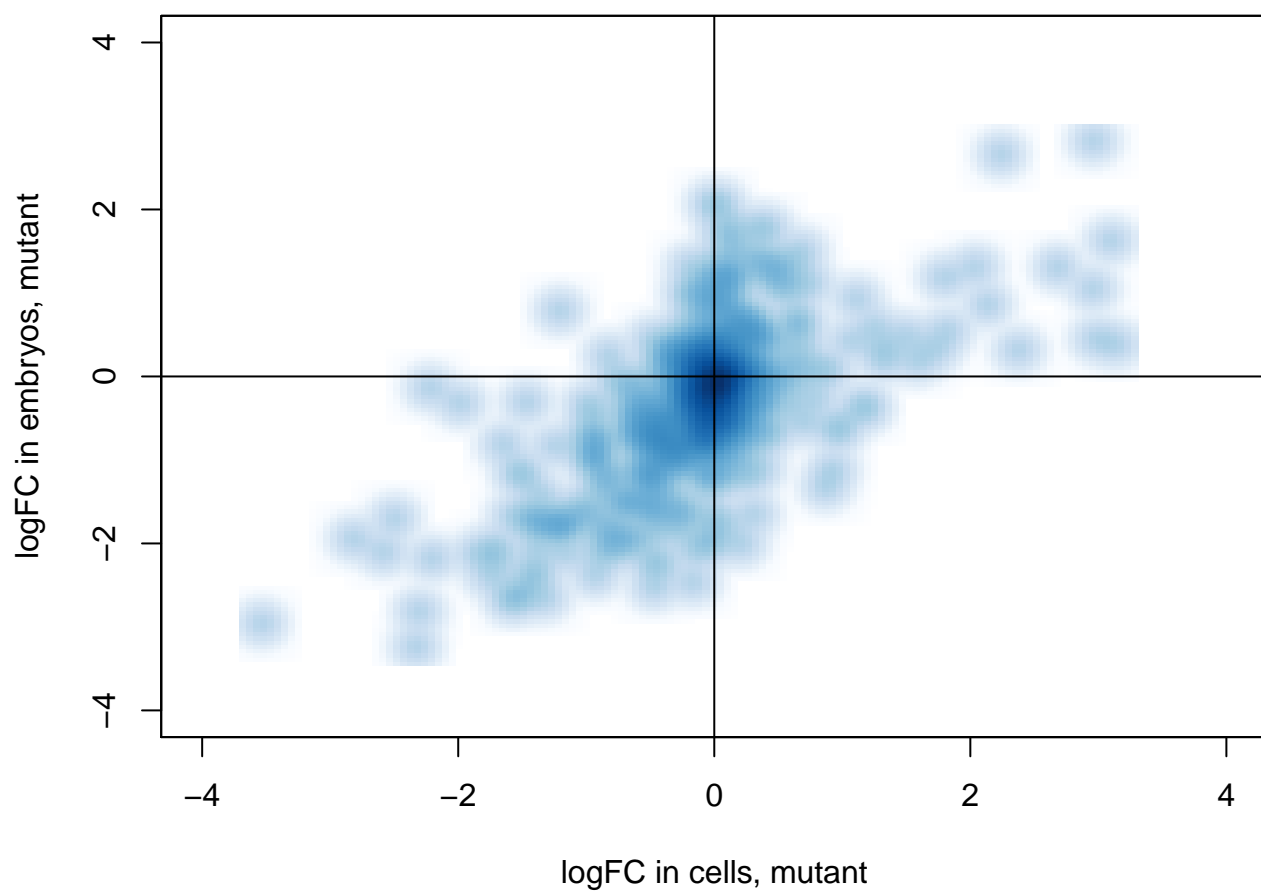

Supplement: File S1 — Archive (gzip2 compressed tar ball format) with 82 PDF files showing scatter plots for GO biological process terms with more than 50 annotated genes. Legend analogous to Figure 2. (BZ2) [file pone.0028985.s013.bz2 › scatterplots for 82 GO process terms with more than 50 genes/proteolysis-results.pdf]

### genes in regulation of cell shape

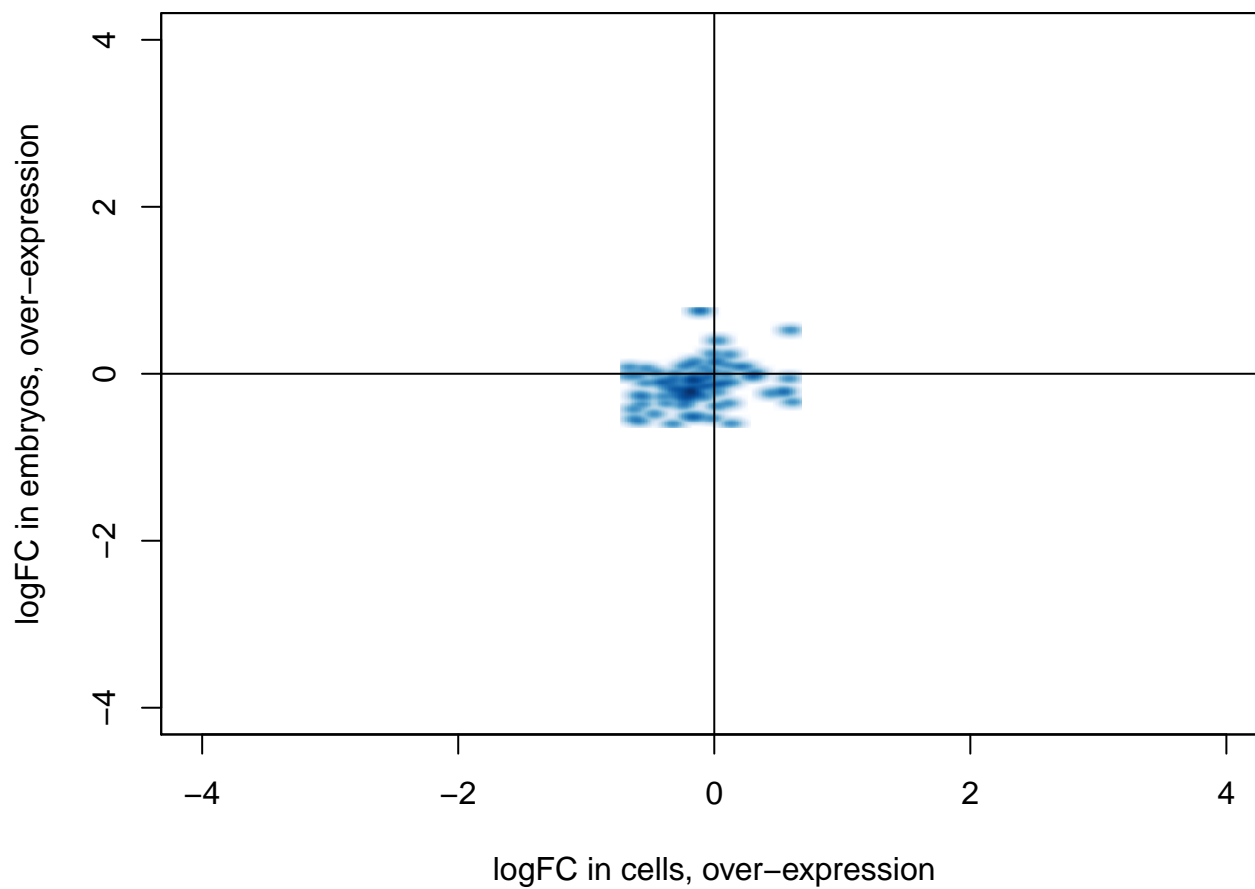

### genes in regulation of cell shape

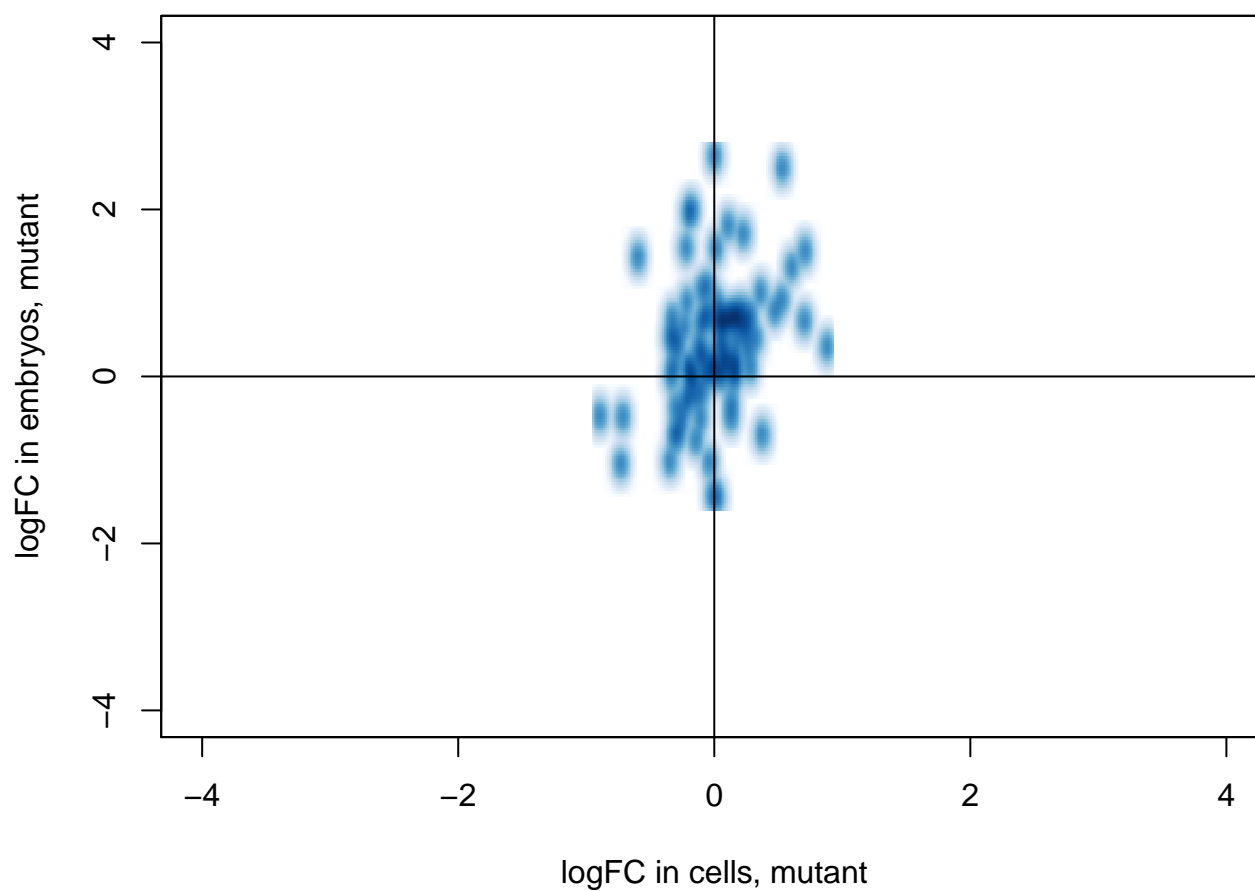

Supplement: File S1 — Archive (gzip2 compressed tar ball format) with 82 PDF files showing scatter plots for GO biological process terms with more than 50 annotated genes. Legend analogous to Figure 2. (BZ2) [file pone.0028985.s013.bz2 › scatterplots for 82 GO process terms with more than 50 genes/regulation of cell shape-results.pdf]

### genes in response to DNA damage stimulus

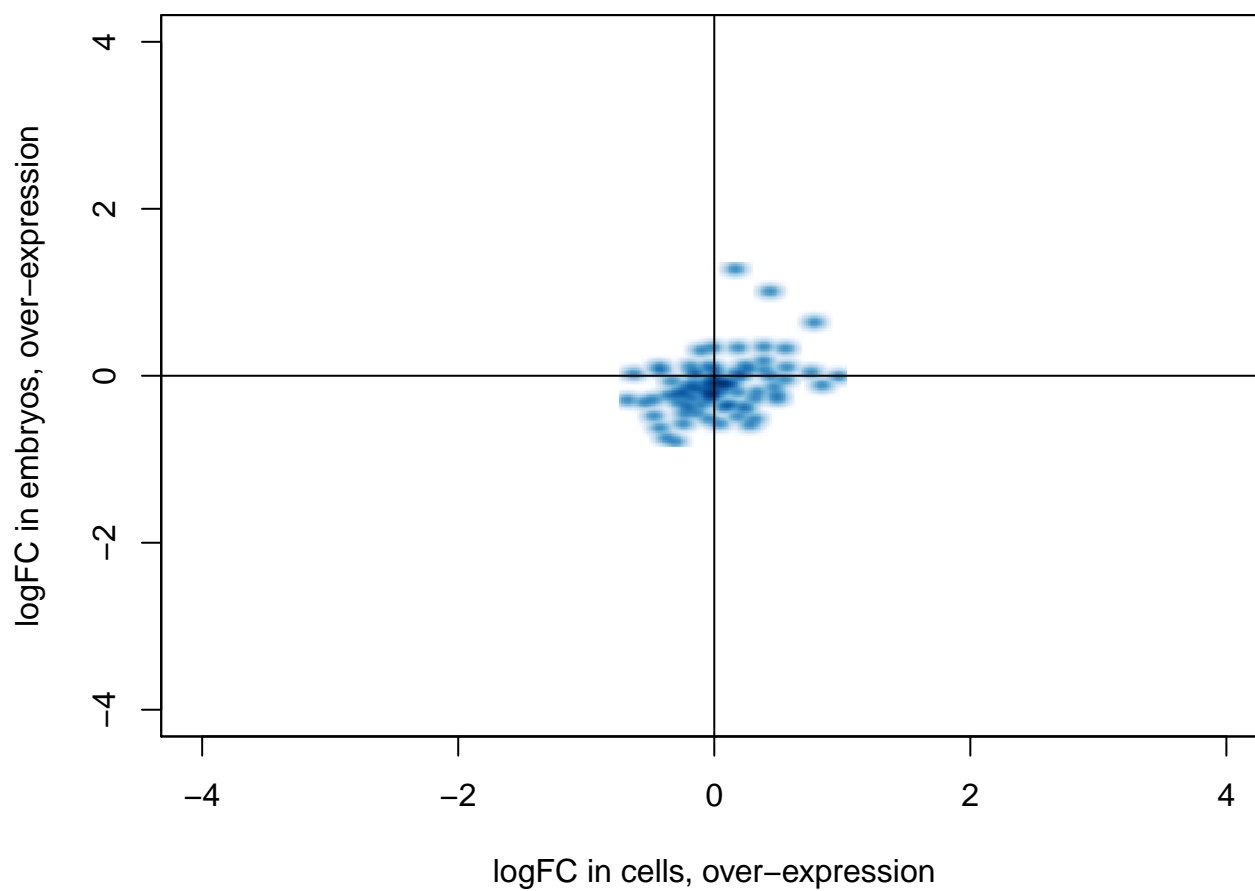

### genes in response to DNA damage stimulus

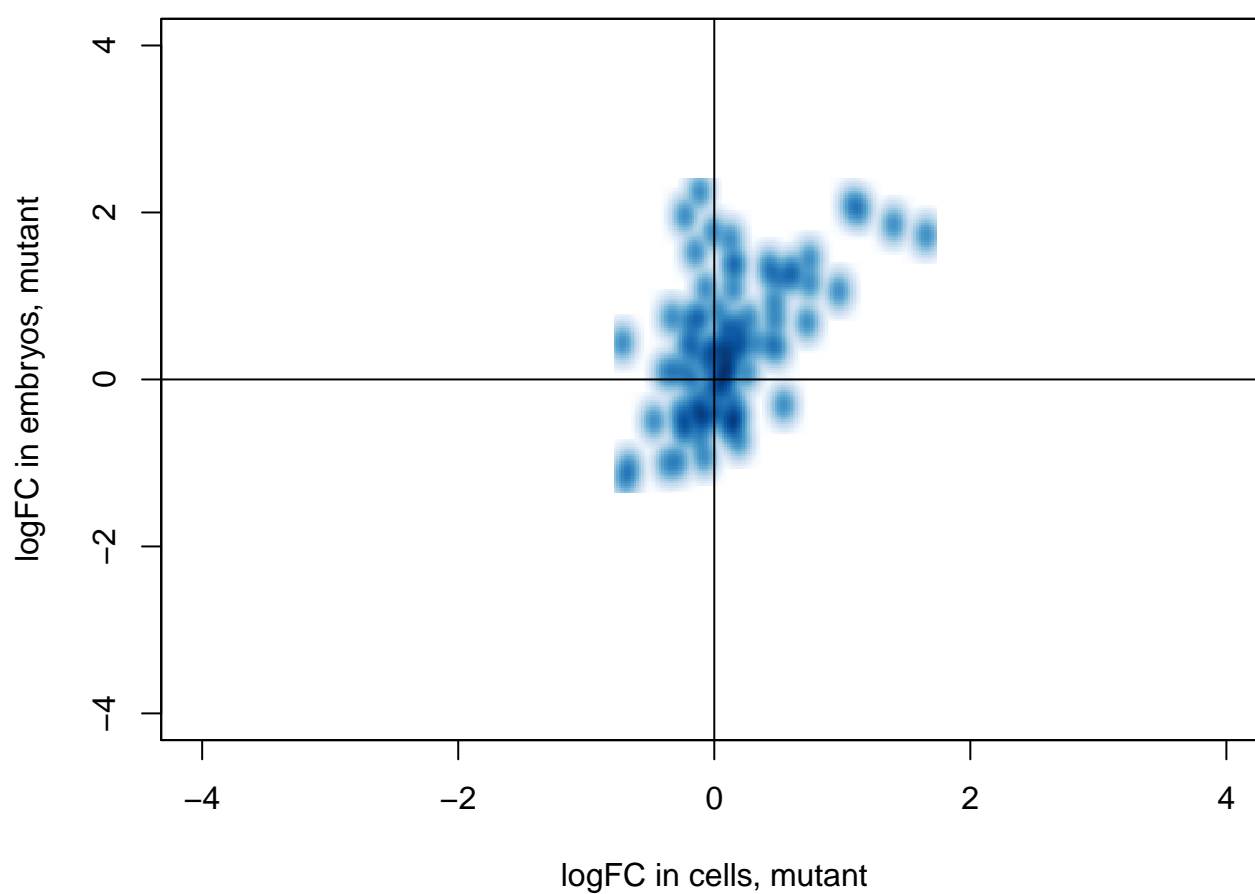

Supplement: File S1 — Archive (gzip2 compressed tar ball format) with 82 PDF files showing scatter plots for GO biological process terms with more than 50 annotated genes. Legend analogous to Figure 2. (BZ2) [file pone.0028985.s013.bz2 › scatterplots for 82 GO process terms with more than 50 genes/response to DNA damage stimulus-results.pdf]

### genes in response to heat

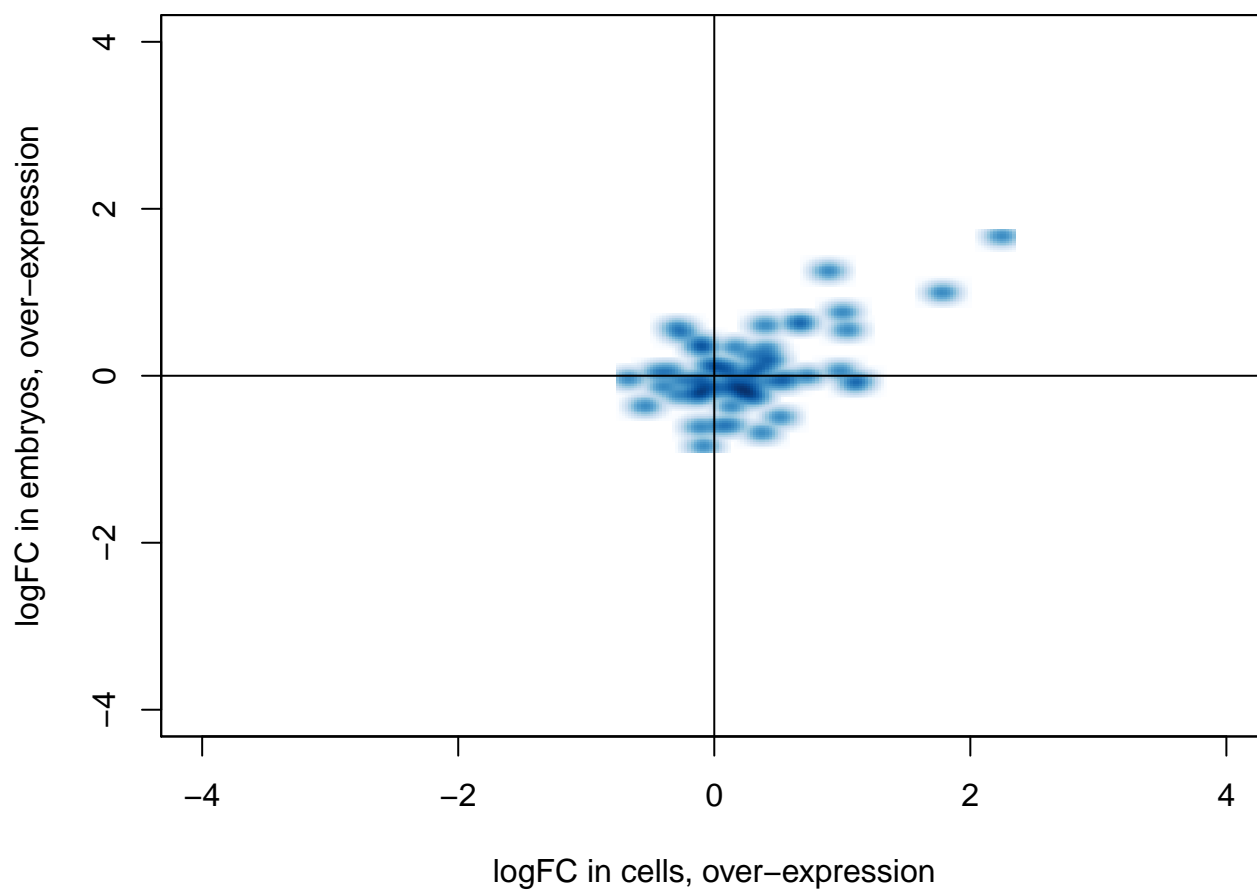

### genes in response to heat

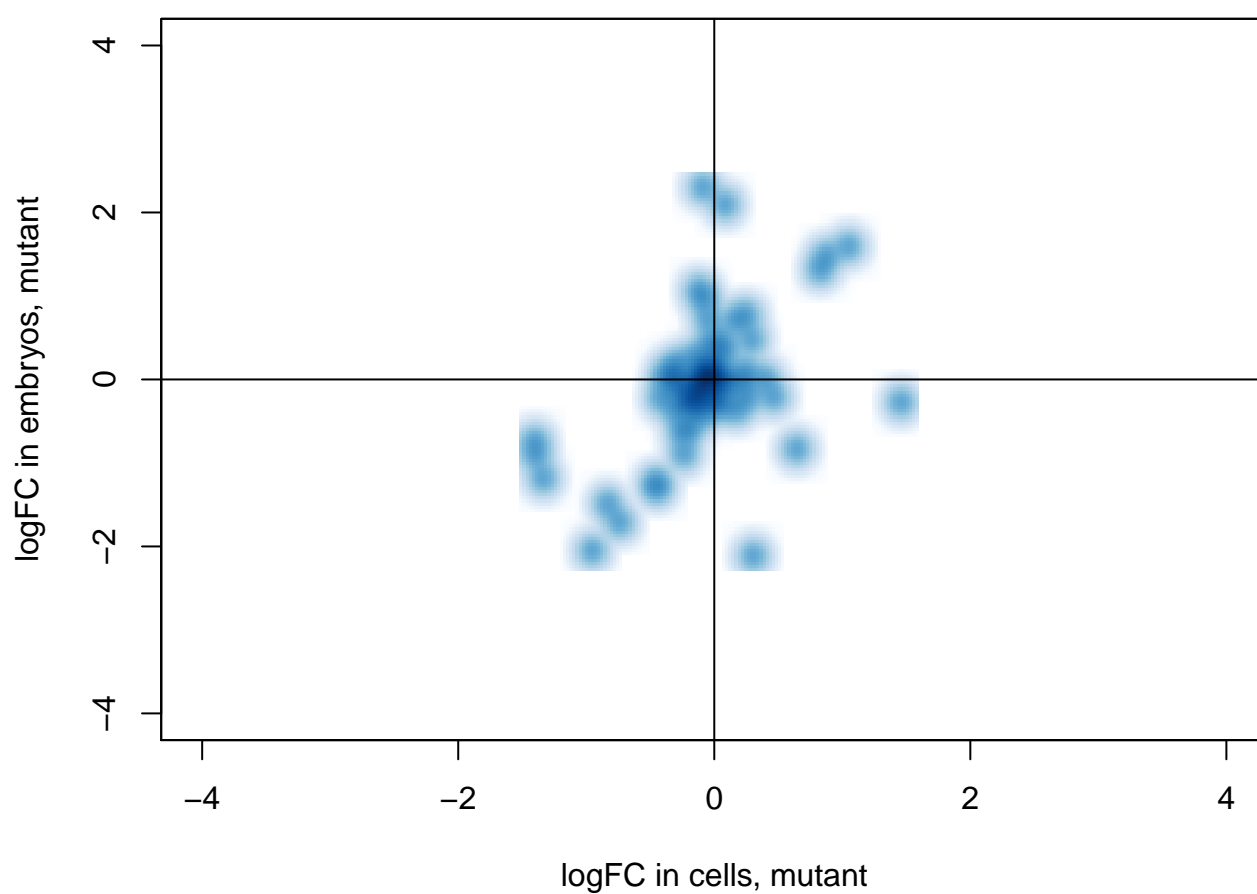

Supplement: File S1 — Archive (gzip2 compressed tar ball format) with 82 PDF files showing scatter plots for GO biological process terms with more than 50 annotated genes. Legend analogous to Figure 2. (BZ2) [file pone.0028985.s013.bz2 › scatterplots for 82 GO process terms with more than 50 genes/response to heat-results.pdf]

### genes in salivary gland morphogenesis

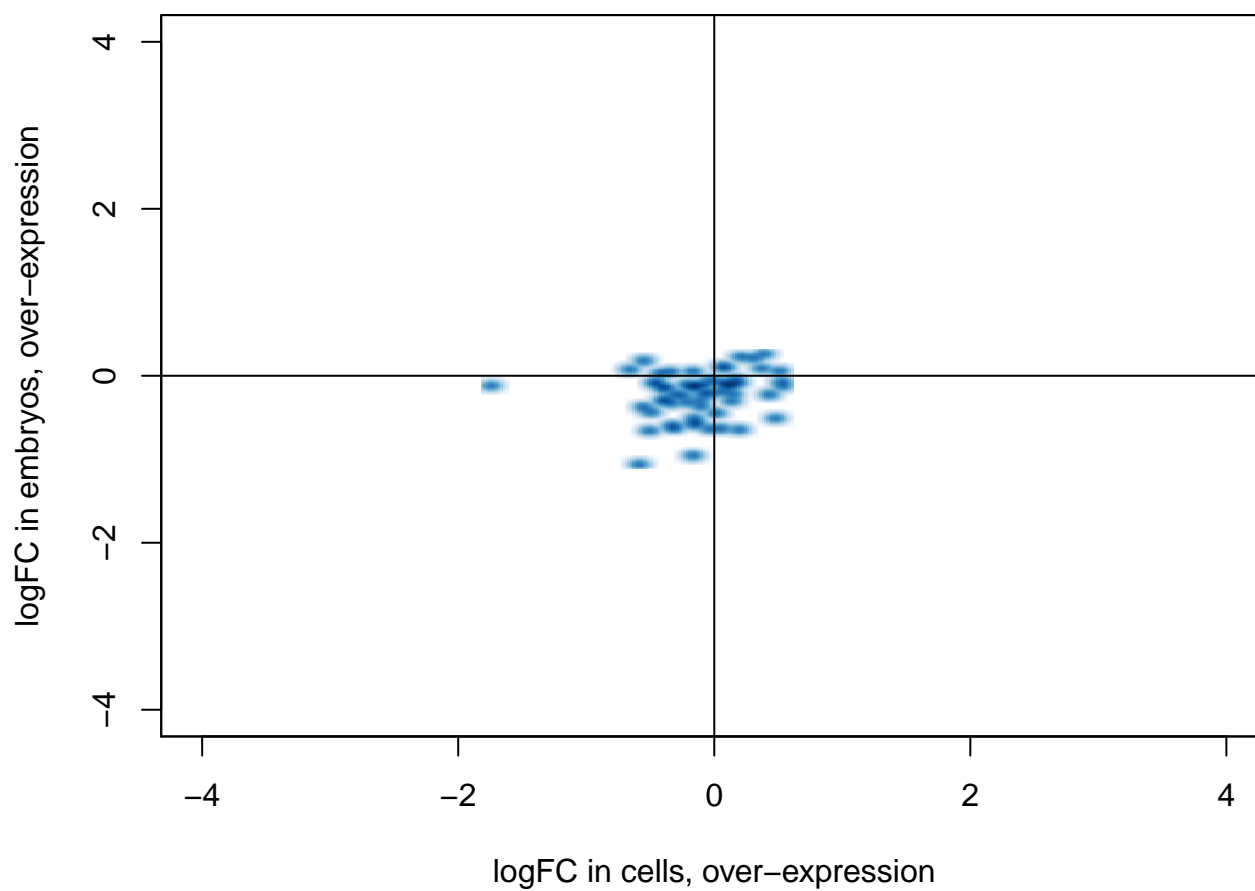

### genes in salivary gland morphogenesis

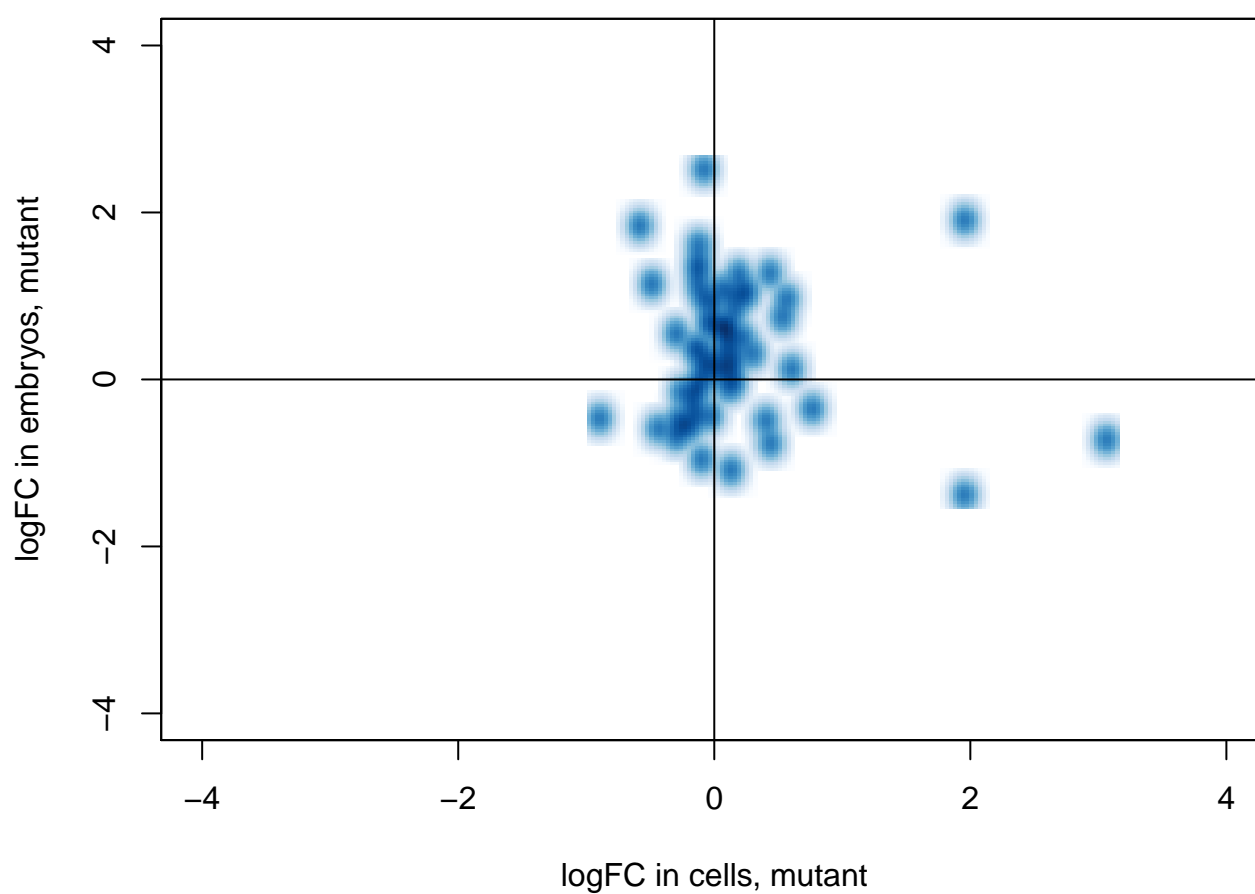

Supplement: File S1 — Archive (gzip2 compressed tar ball format) with 82 PDF files showing scatter plots for GO biological process terms with more than 50 annotated genes. Legend analogous to Figure 2. (BZ2) [file pone.0028985.s013.bz2 › scatterplots for 82 GO process terms with more than 50 genes/salivary gland morphogenesis-results.pdf]

### genes in sensory organ development

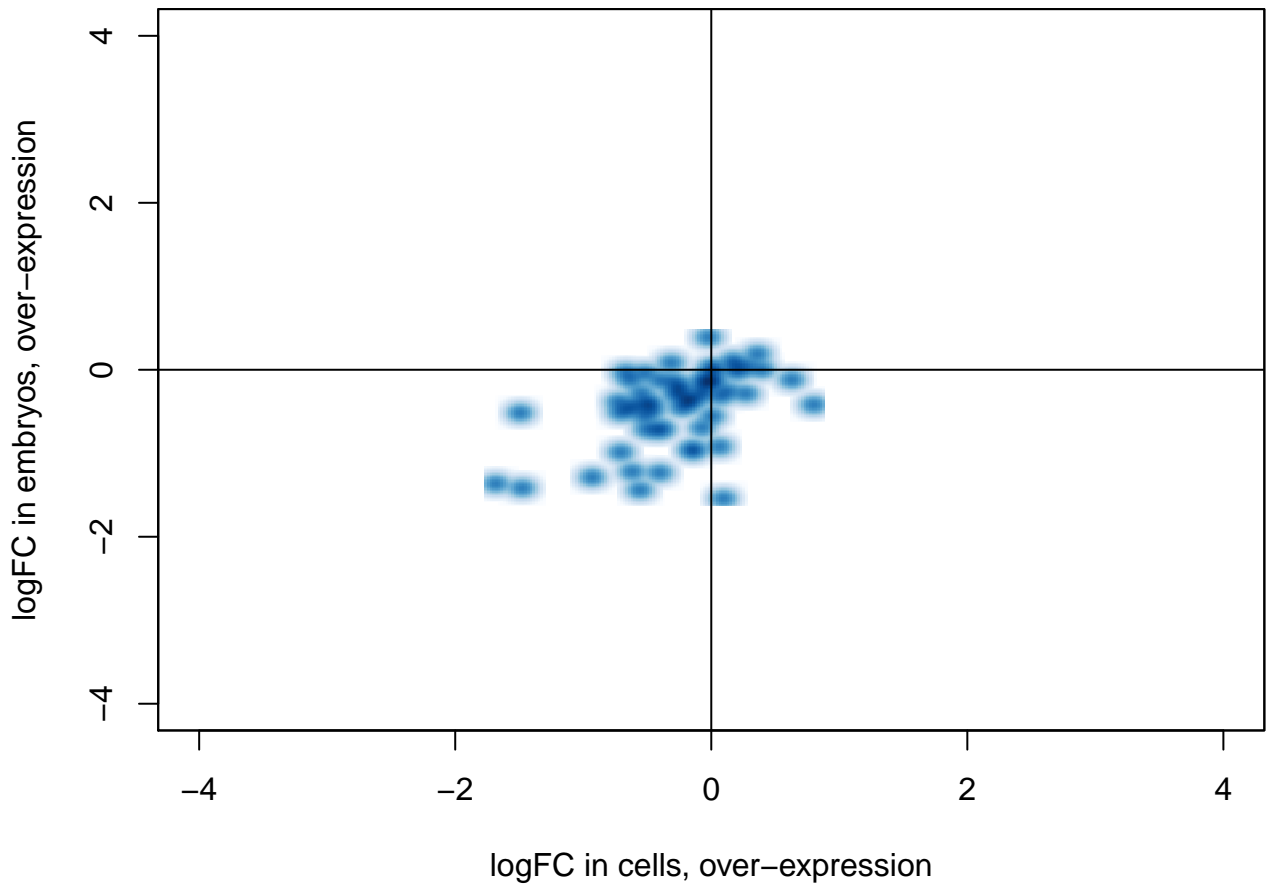

### genes in sensory organ development

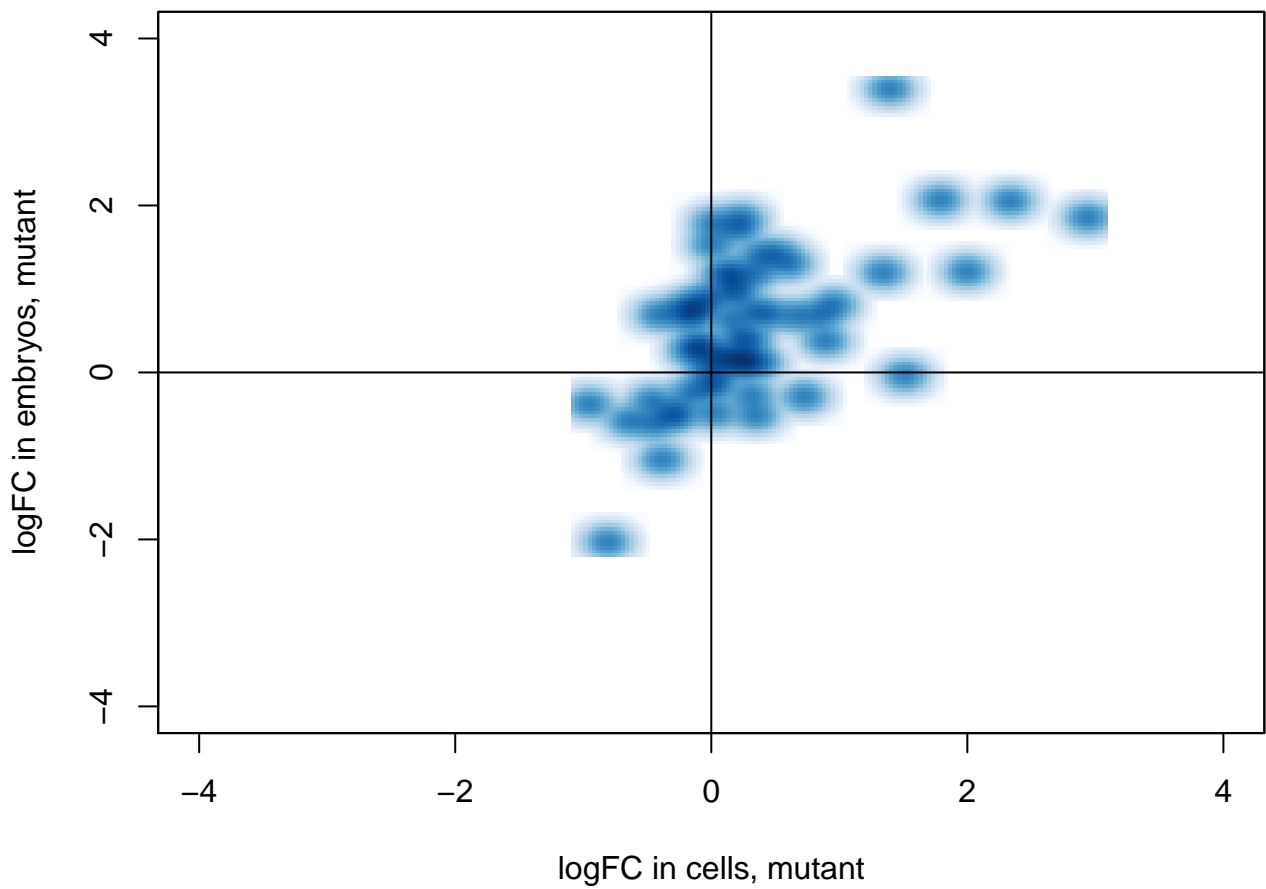

Supplement: File S1 — Archive (gzip2 compressed tar ball format) with 82 PDF files showing scatter plots for GO biological process terms with more than 50 annotated genes. Legend analogous to Figure 2. (BZ2) [file pone.0028985.s013.bz2 › scatterplots for 82 GO process terms with more than 50 genes/sensory organ development-results.pdf]

### genes in sensory perception of smell

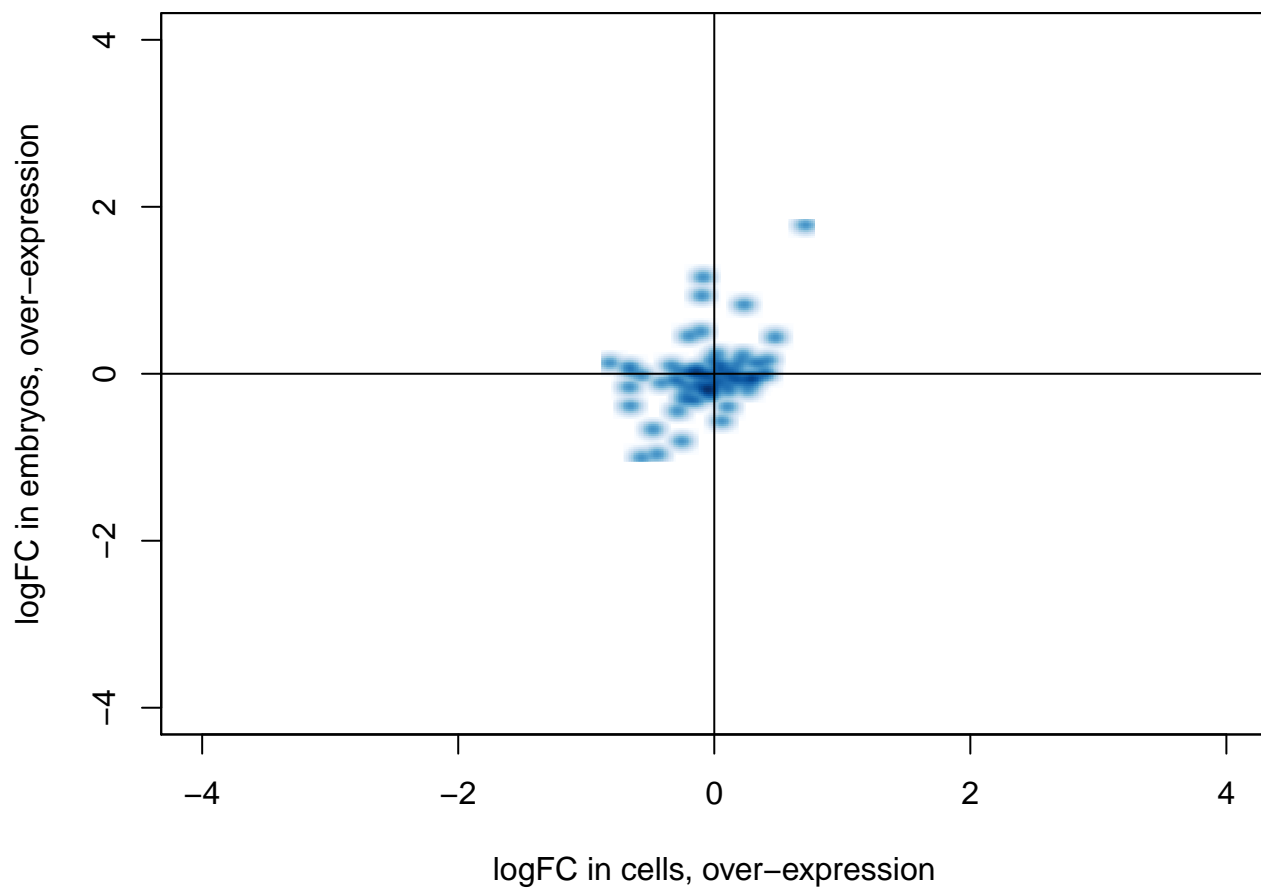

### genes in sensory perception of smell

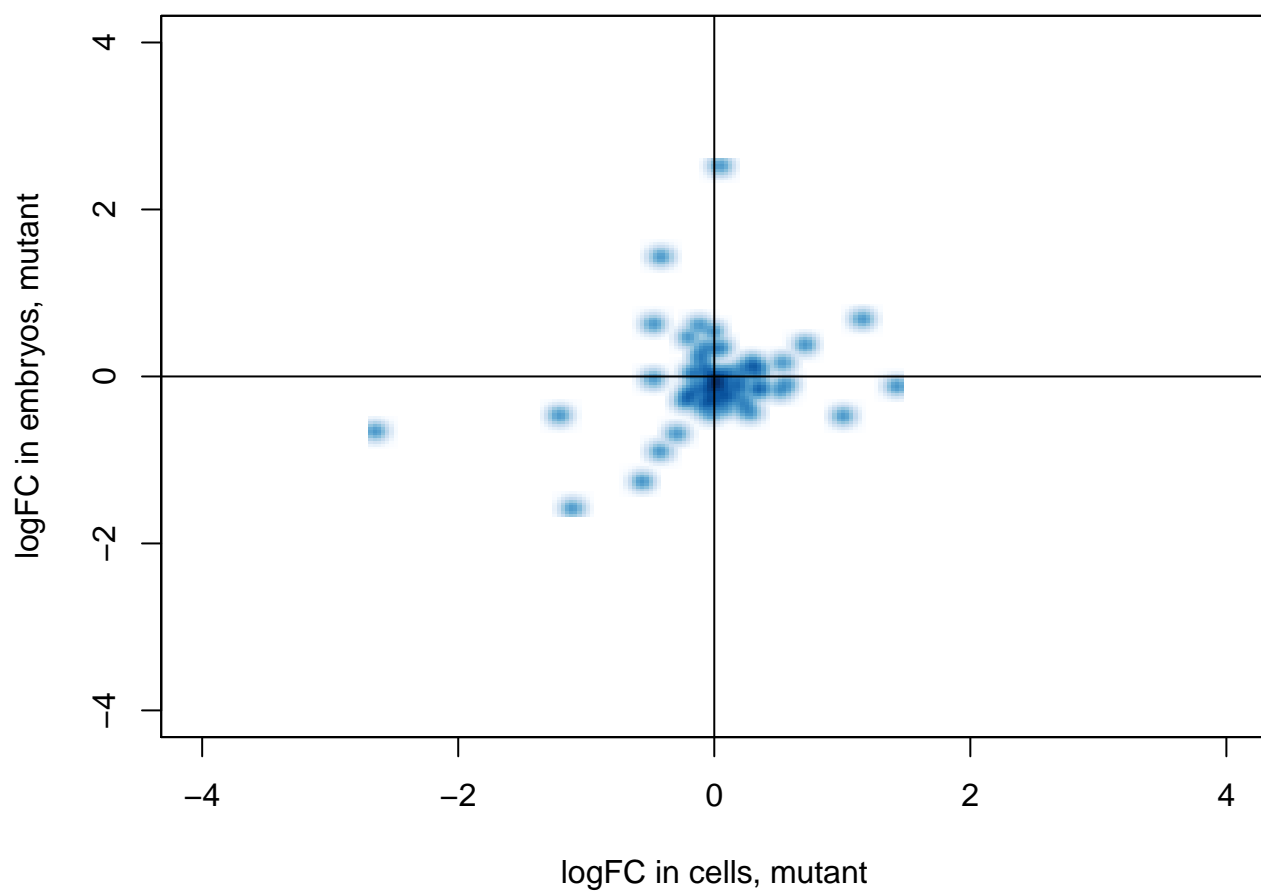

Supplement: File S1 — Archive (gzip2 compressed tar ball format) with 82 PDF files showing scatter plots for GO biological process terms with more than 50 annotated genes. Legend analogous to Figure 2. (BZ2) [file pone.0028985.s013.bz2 › scatterplots for 82 GO process terms with more than 50 genes/sensory perception of smell-results.pdf]

### genes in sensory perception of taste

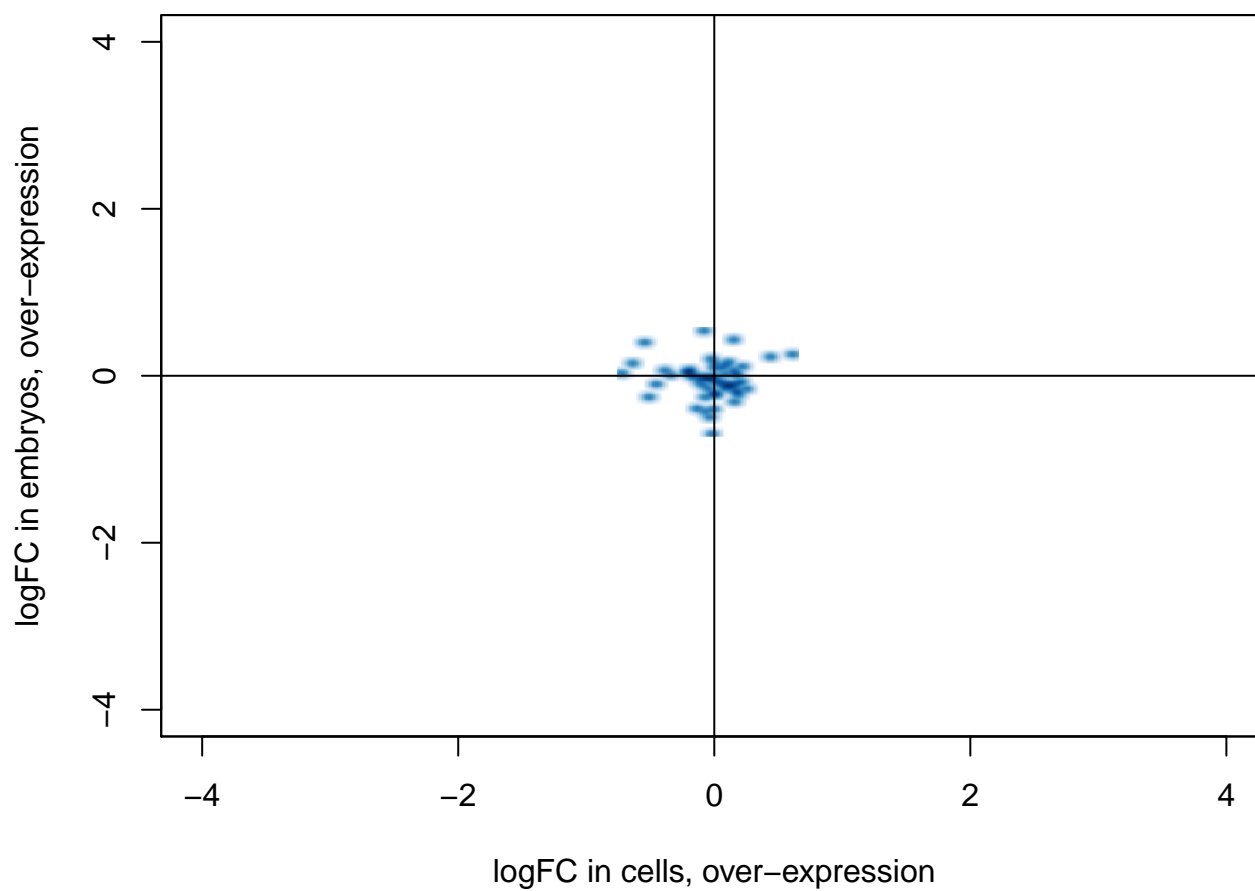

### genes in sensory perception of taste

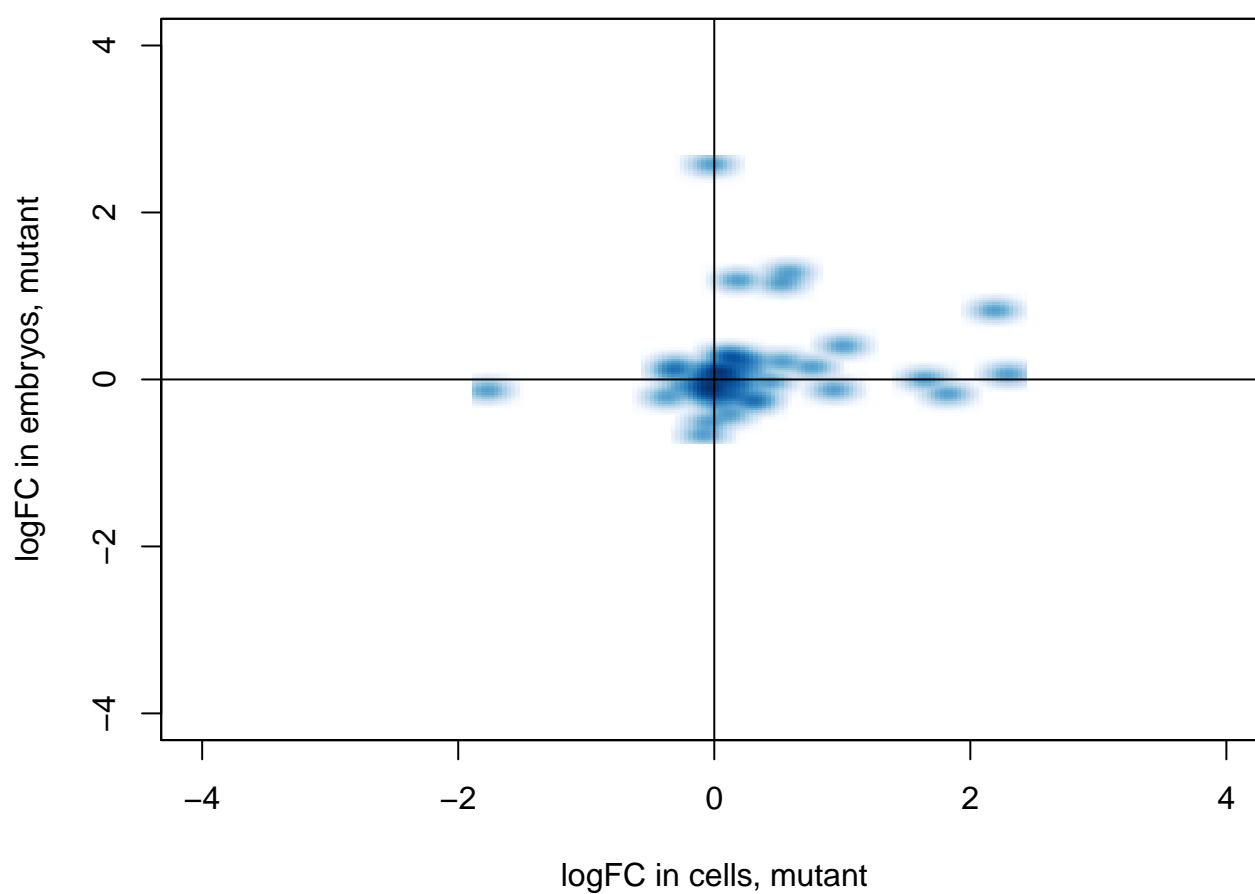

Supplement: File S1 — Archive (gzip2 compressed tar ball format) with 82 PDF files showing scatter plots for GO biological process terms with more than 50 annotated genes. Legend analogous to Figure 2. (BZ2) [file pone.0028985.s013.bz2 › scatterplots for 82 GO process terms with more than 50 genes/sensory perception of taste-results.pdf]

### genes in signal transduction

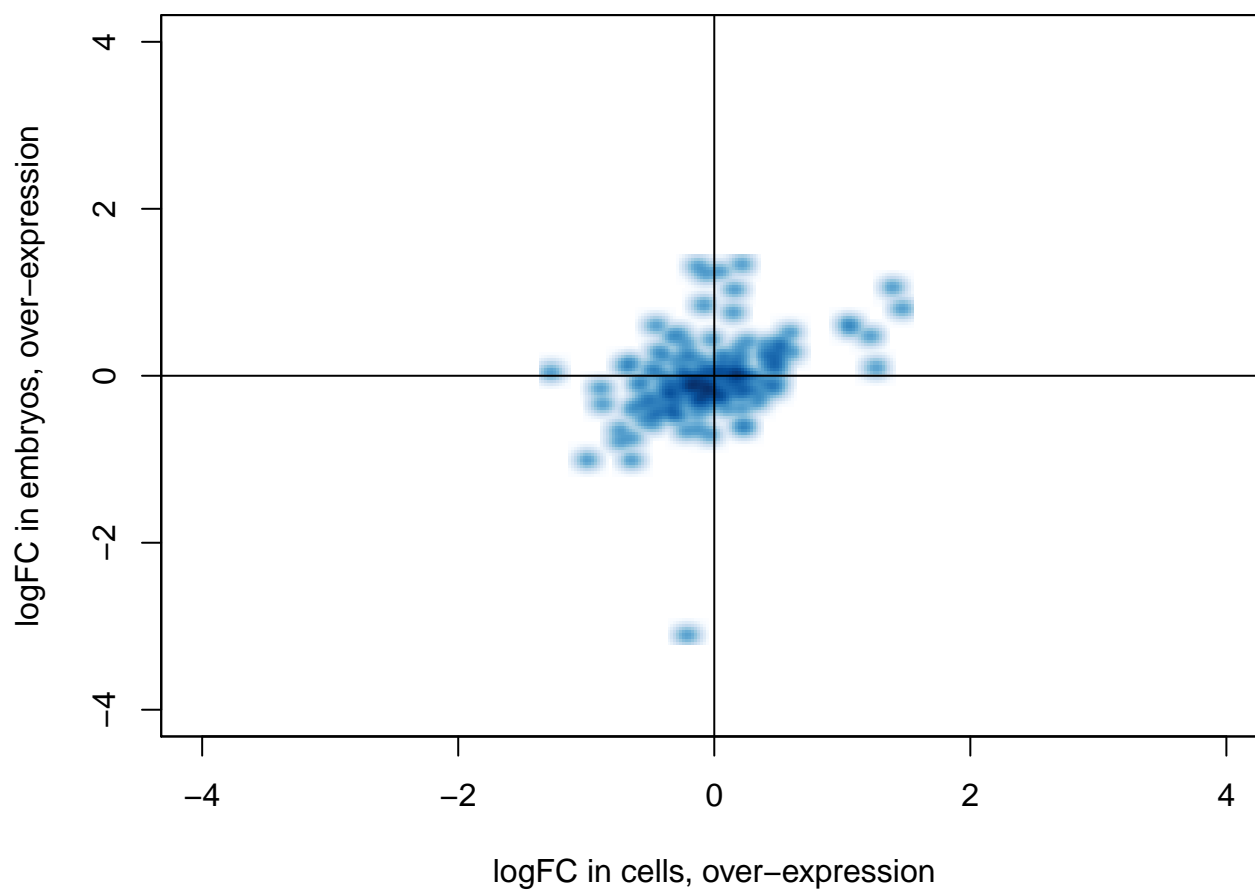

### genes in signal transduction

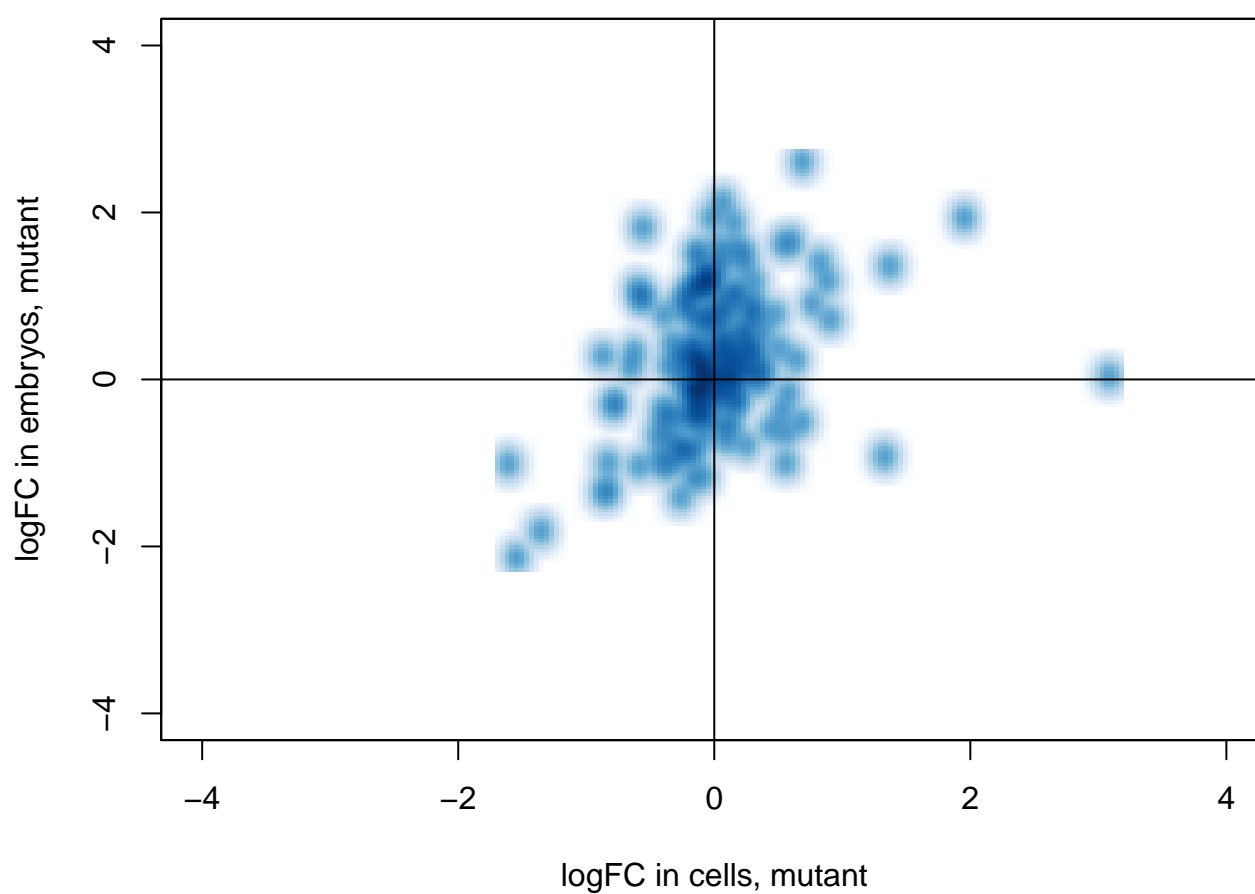

Supplement: File S1 — Archive (gzip2 compressed tar ball format) with 82 PDF files showing scatter plots for GO biological process terms with more than 50 annotated genes. Legend analogous to Figure 2. (BZ2) [file pone.0028985.s013.bz2 › scatterplots for 82 GO process terms with more than 50 genes/signal transduction-results.pdf]

**genes in sodium ion transport**

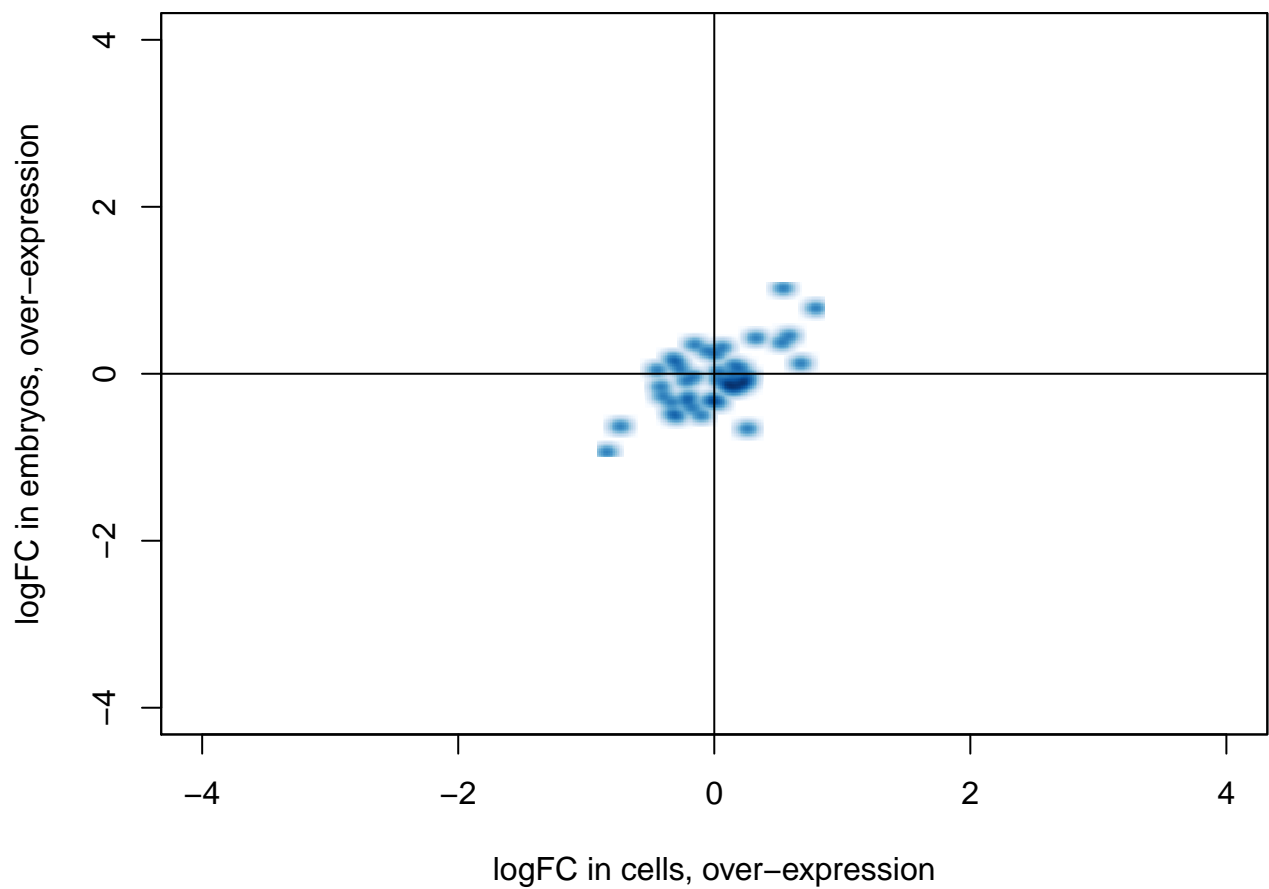

**genes in sodium ion transport**

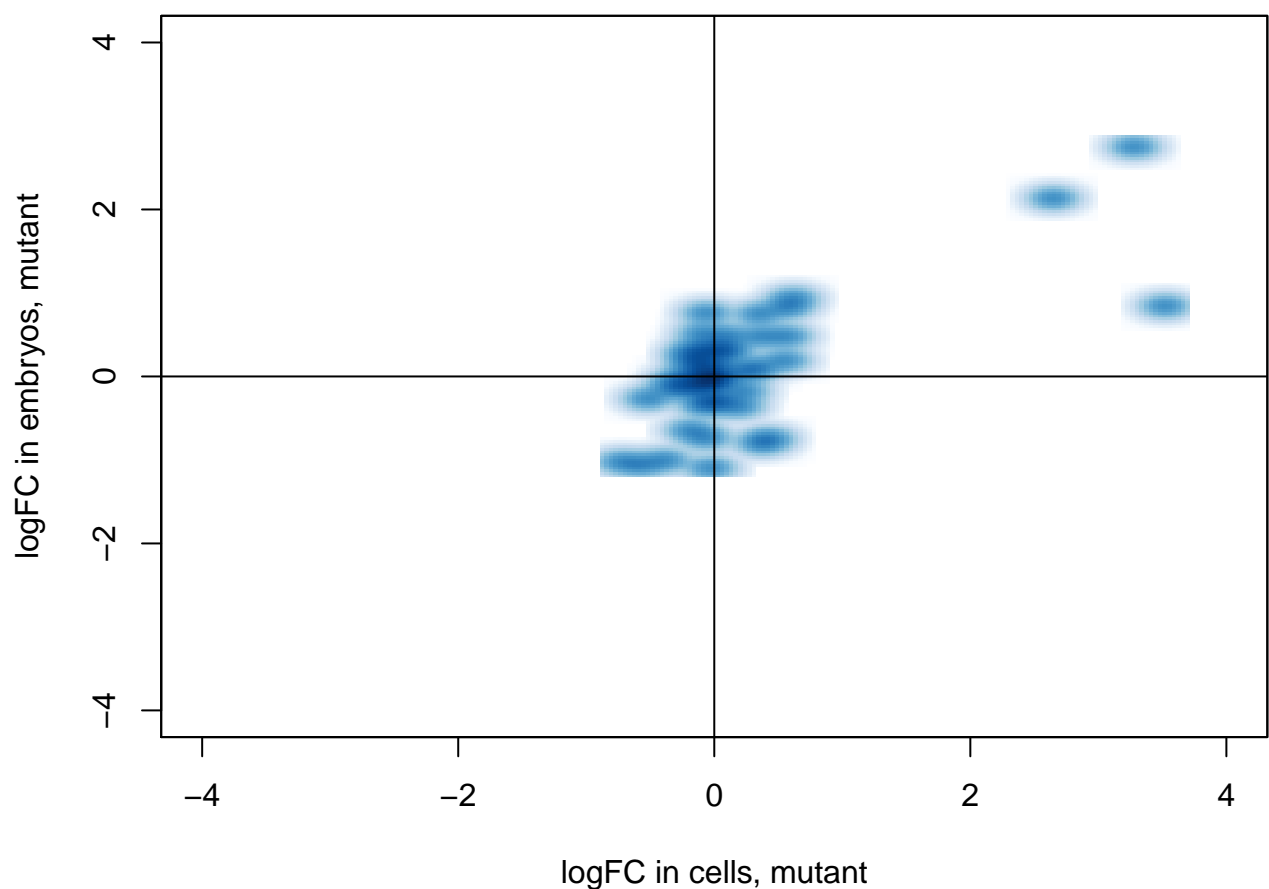

Supplement: File S1 — Archive (gzip2 compressed tar ball format) with 82 PDF files showing scatter plots for GO biological process terms with more than 50 annotated genes. Legend analogous to Figure 2. (BZ2) [file pone.0028985.s013.bz2 › scatterplots for 82 GO process terms with more than 50 genes/sodium ion transport-results.pdf]

### genes in spermatogenesis

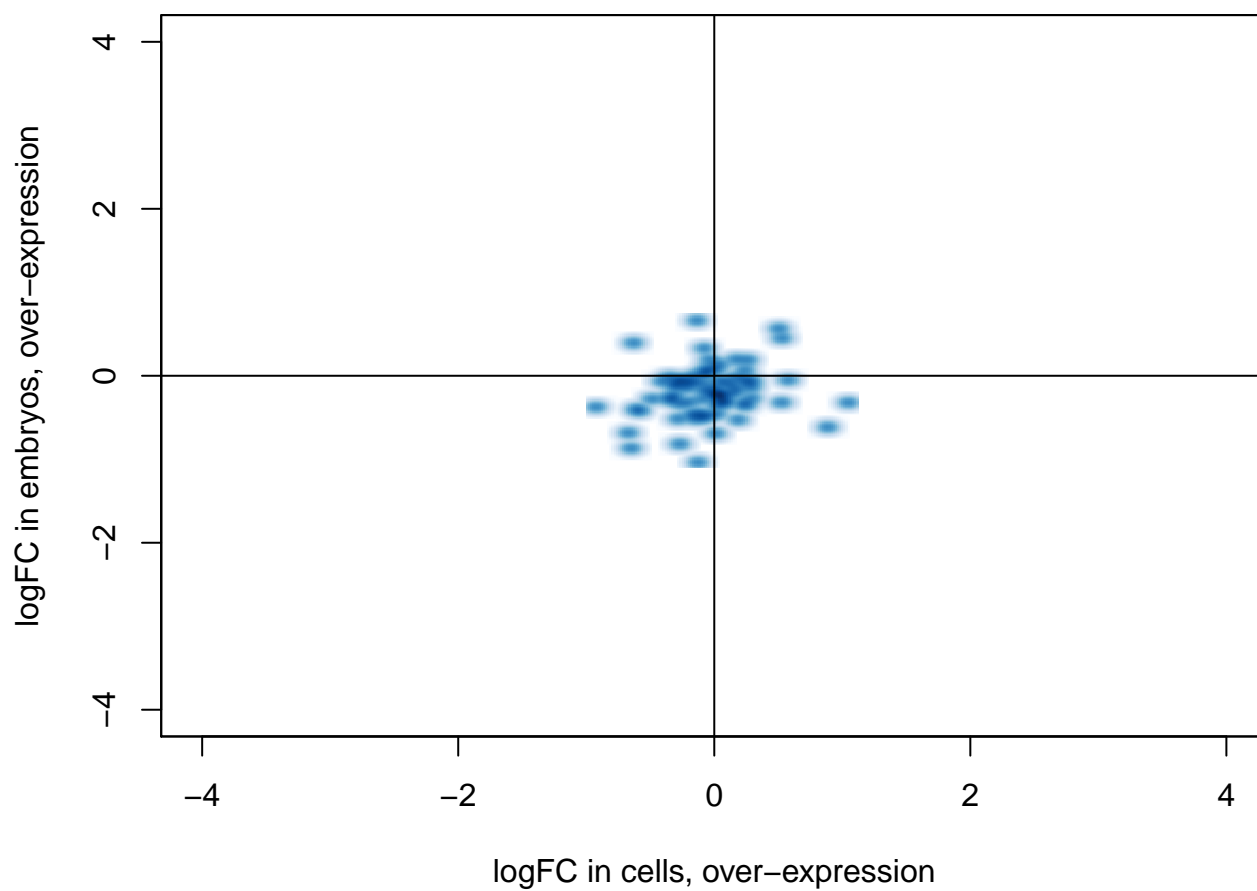

### genes in spermatogenesis

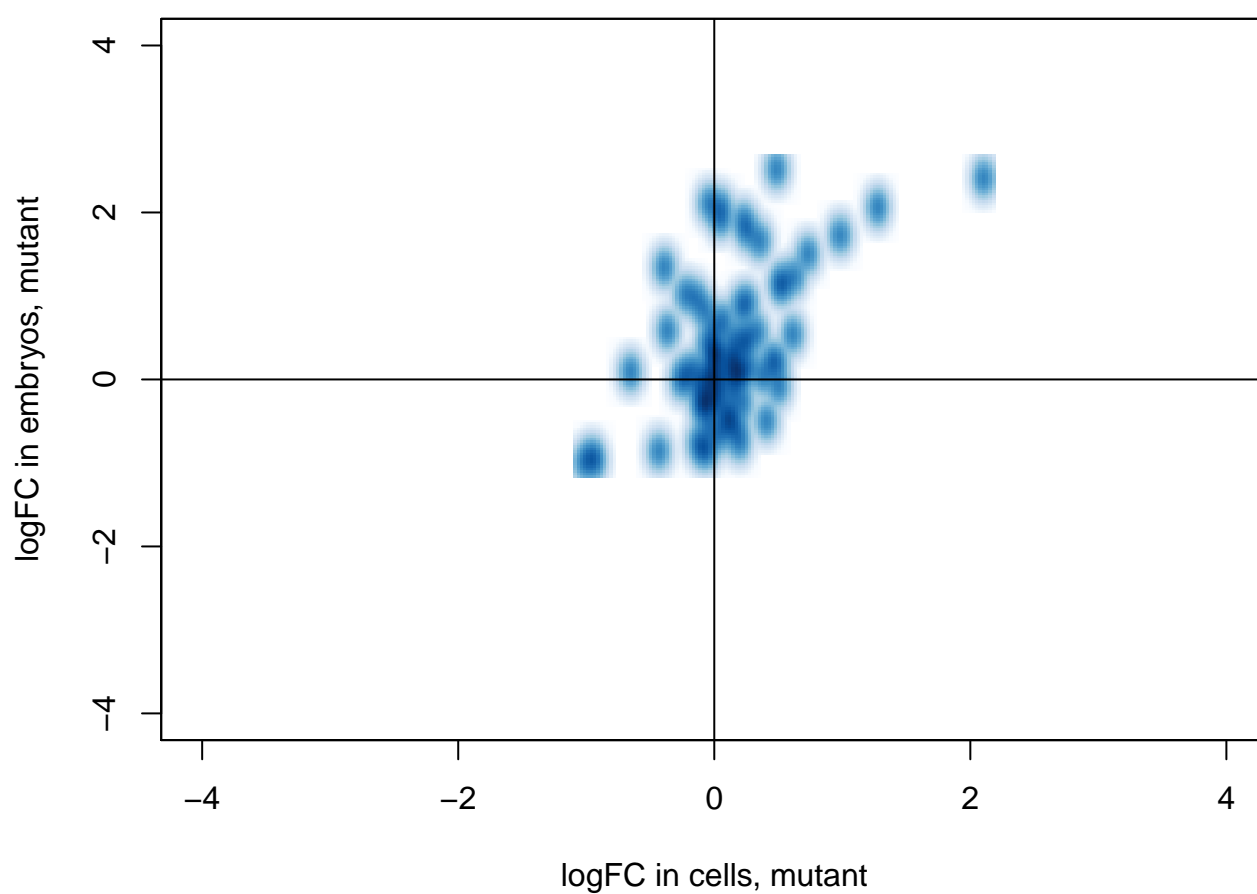

Supplement: File S1 — Archive (gzip2 compressed tar ball format) with 82 PDF files showing scatter plots for GO biological process terms with more than 50 annotated genes. Legend analogous to Figure 2. (BZ2) [file pone.0028985.s013.bz2 › scatterplots for 82 GO process terms with more than 50 genes/spermatogenesis-results.pdf]

### genes in translation

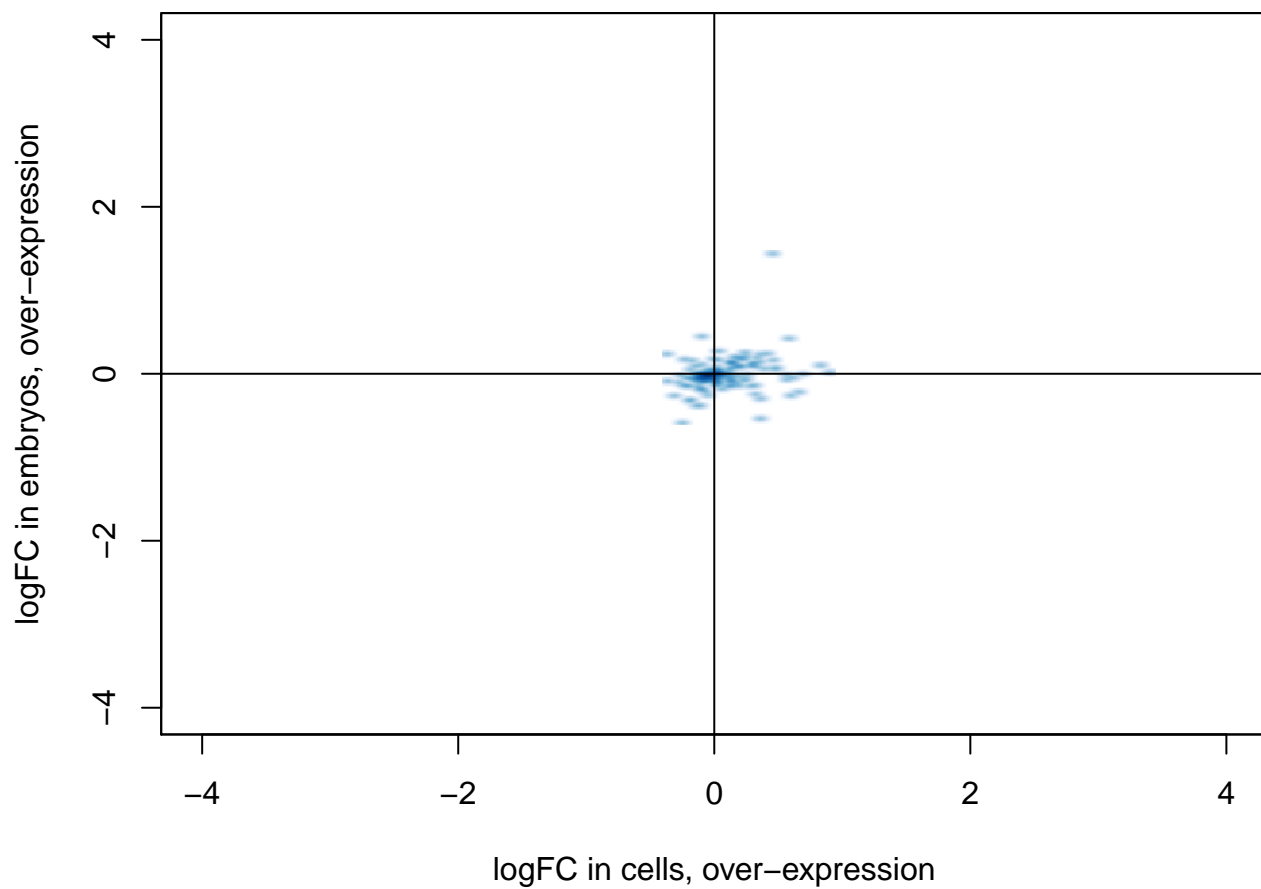

### genes in translation

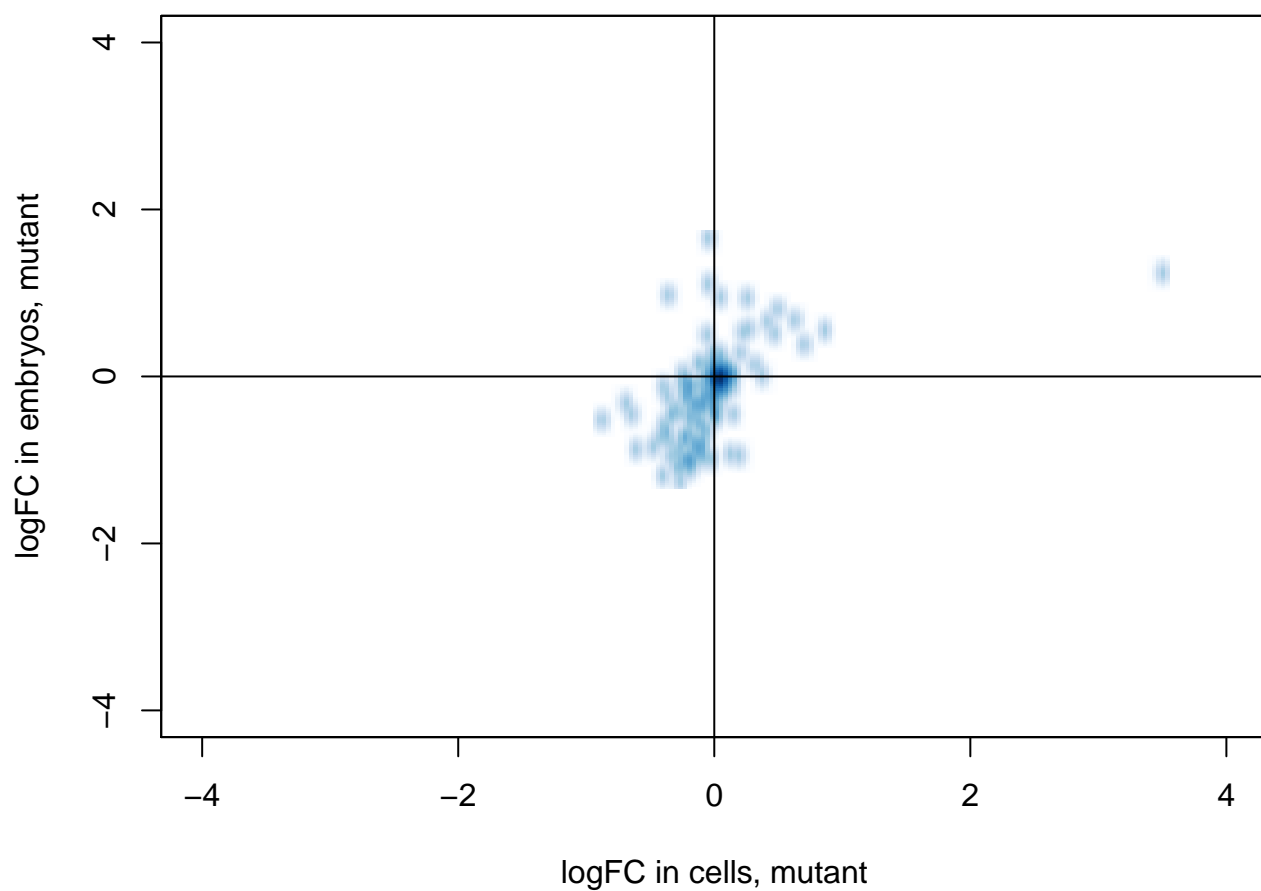

Supplement: File S1 — Archive (gzip2 compressed tar ball format) with 82 PDF files showing scatter plots for GO biological process terms with more than 50 annotated genes. Legend analogous to Figure 2. (BZ2) [file pone.0028985.s013.bz2 › scatterplots for 82 GO process terms with more than 50 genes/translation-results.pdf]

### genes in translational initiation

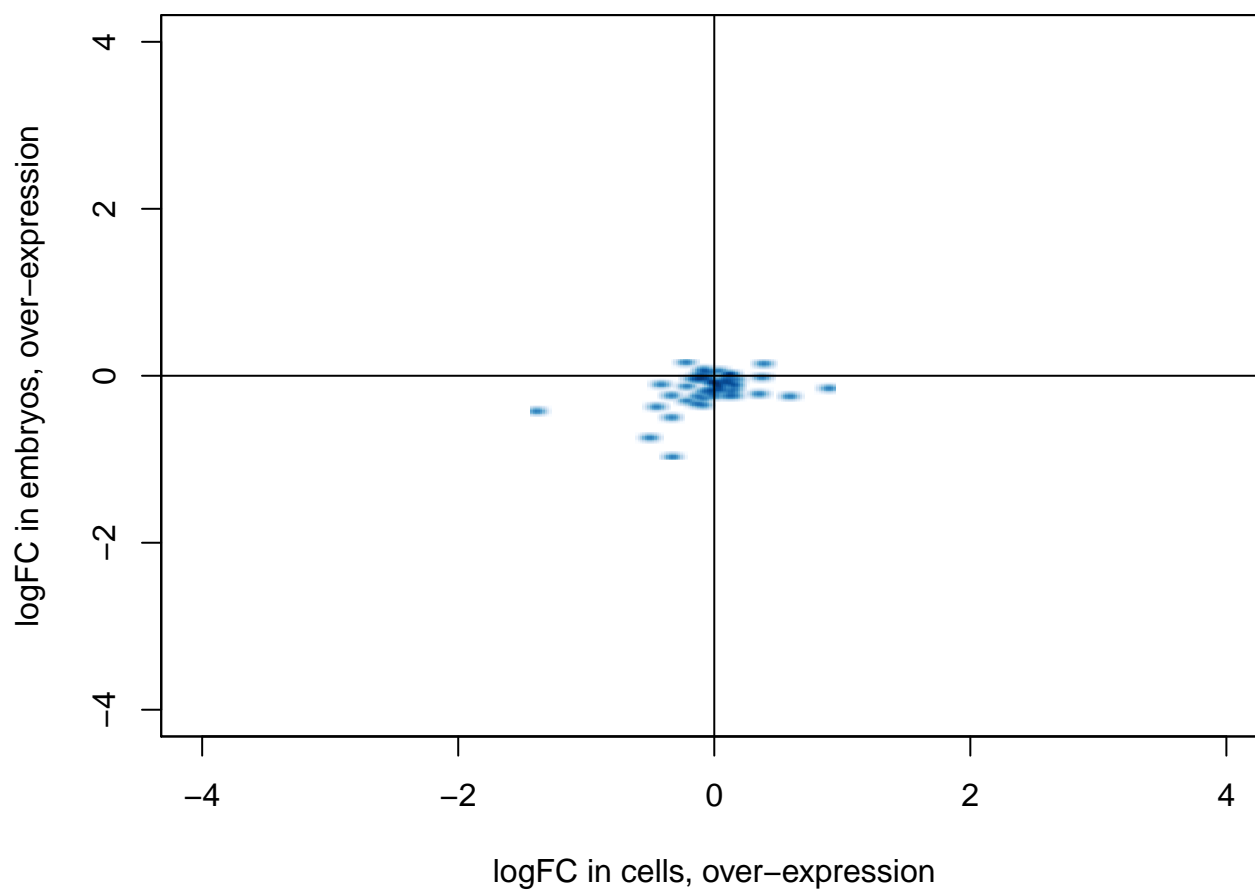

### genes in translational initiation

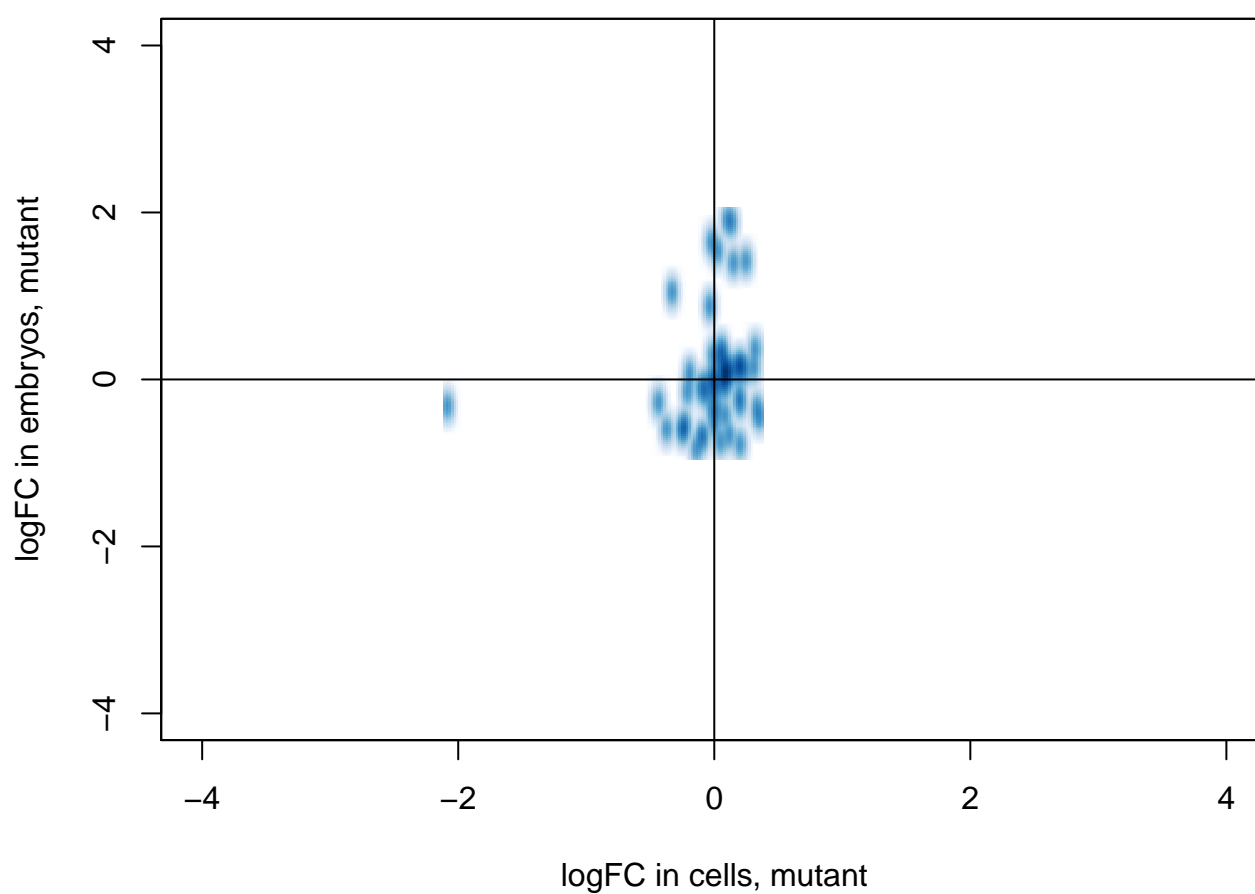

Supplement: File S1 — Archive (gzip2 compressed tar ball format) with 82 PDF files showing scatter plots for GO biological process terms with more than 50 annotated genes. Legend analogous to Figure 2. (BZ2) [file pone.0028985.s013.bz2 › scatterplots for 82 GO process terms with more than 50 genes/translational initiation-results.pdf]

### genes in transmembrane transport

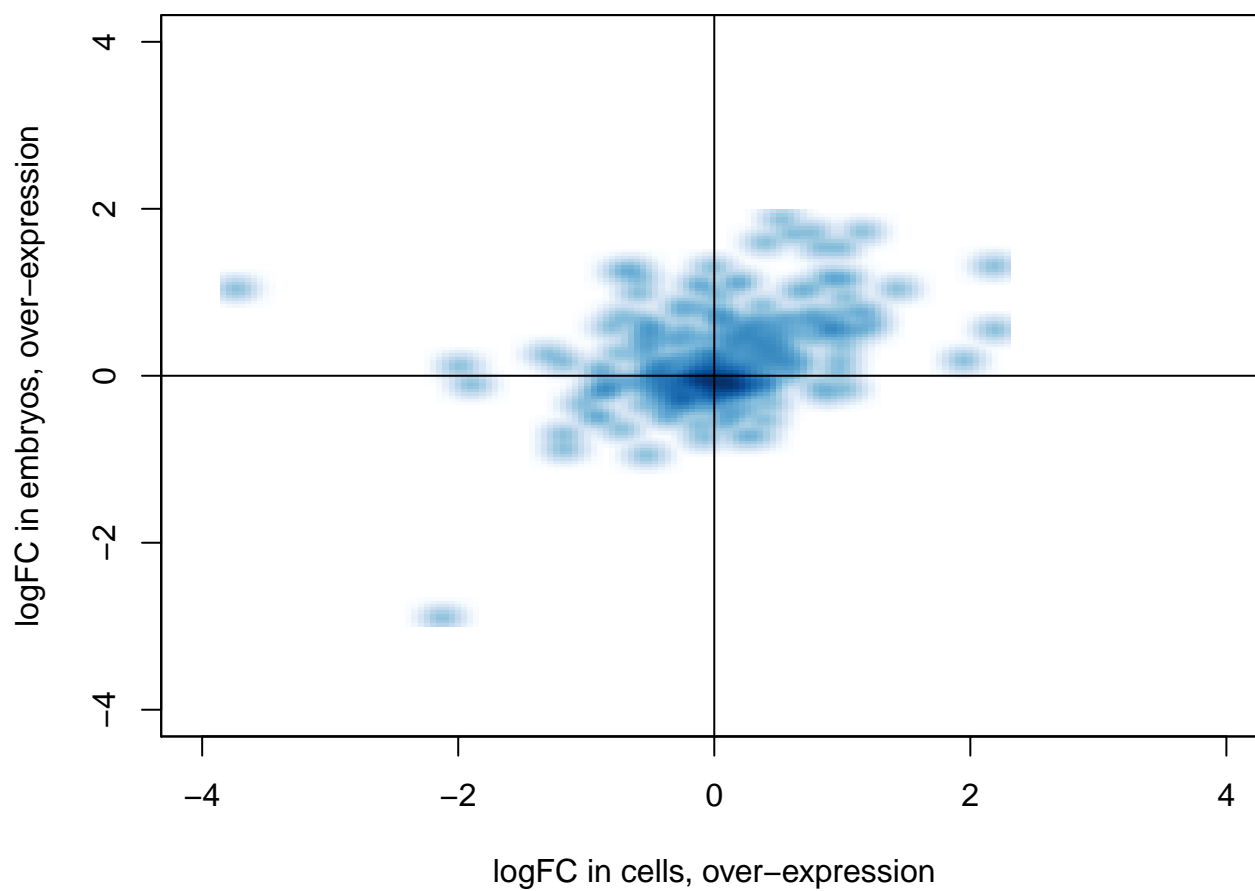

### genes in transmembrane transport

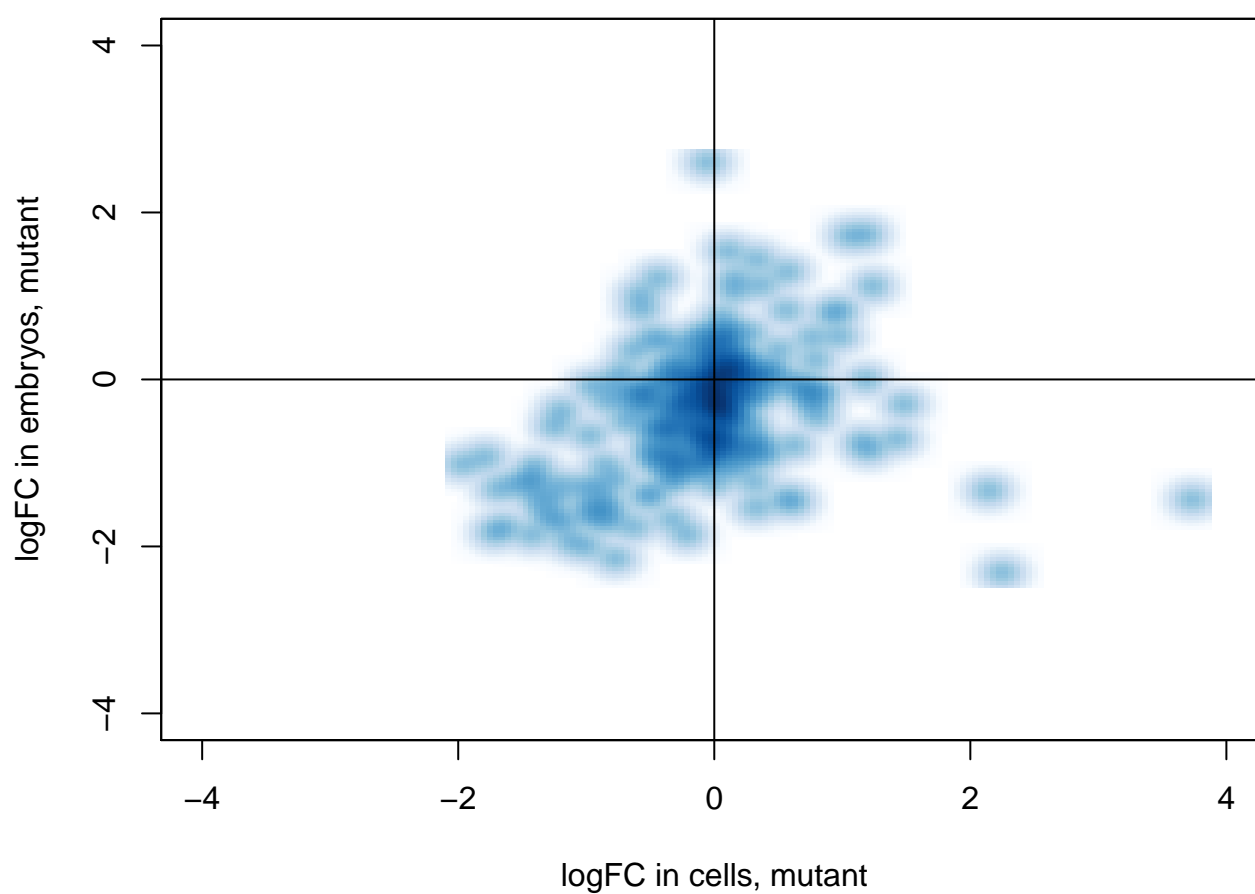

Supplement: File S1 — Archive (gzip2 compressed tar ball format) with 82 PDF files showing scatter plots for GO biological process terms with more than 50 annotated genes. Legend analogous to Figure 2. (BZ2) [file pone.0028985.s013.bz2 › scatterplots for 82 GO process terms with more than 50 genes/transmembrane transport-results.pdf]

### genes in transport

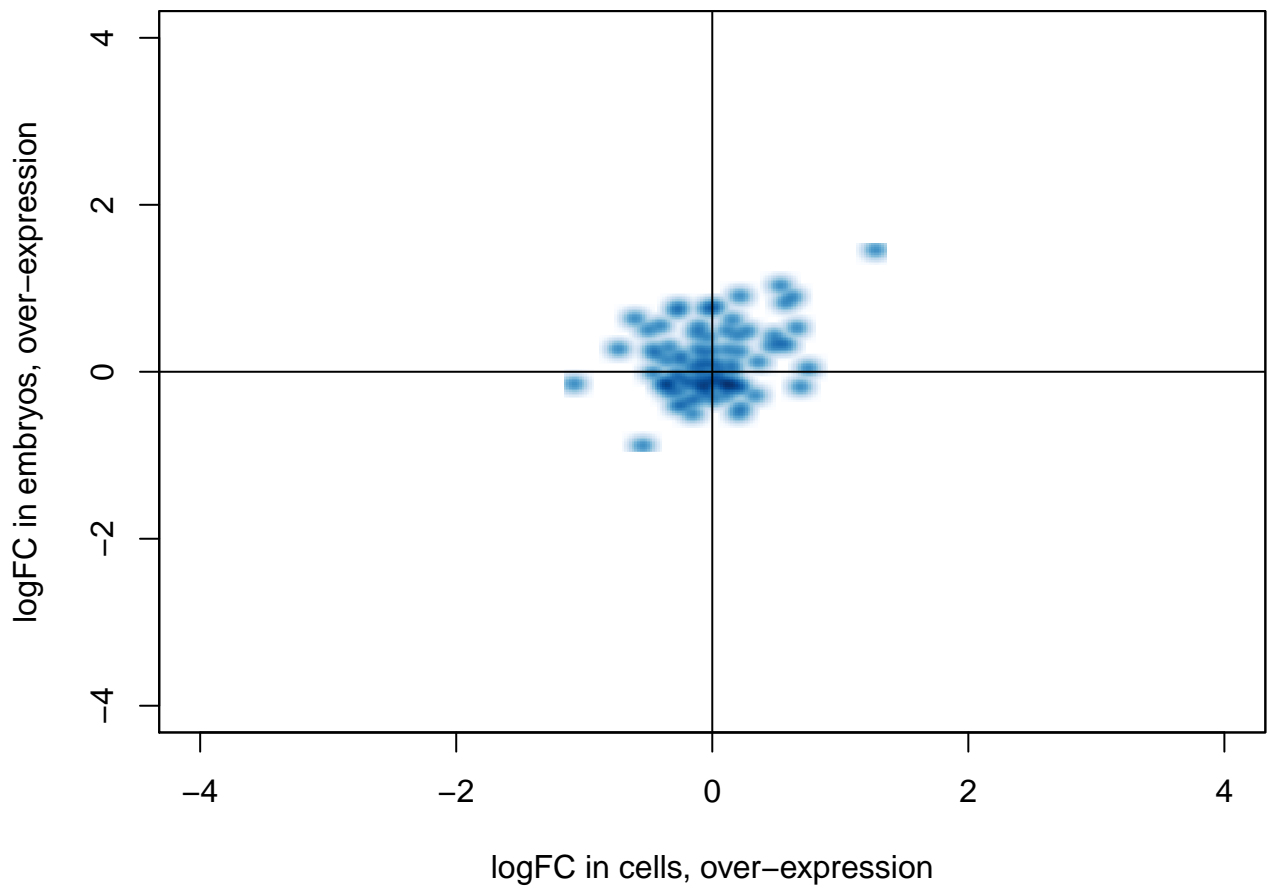

### genes in transport

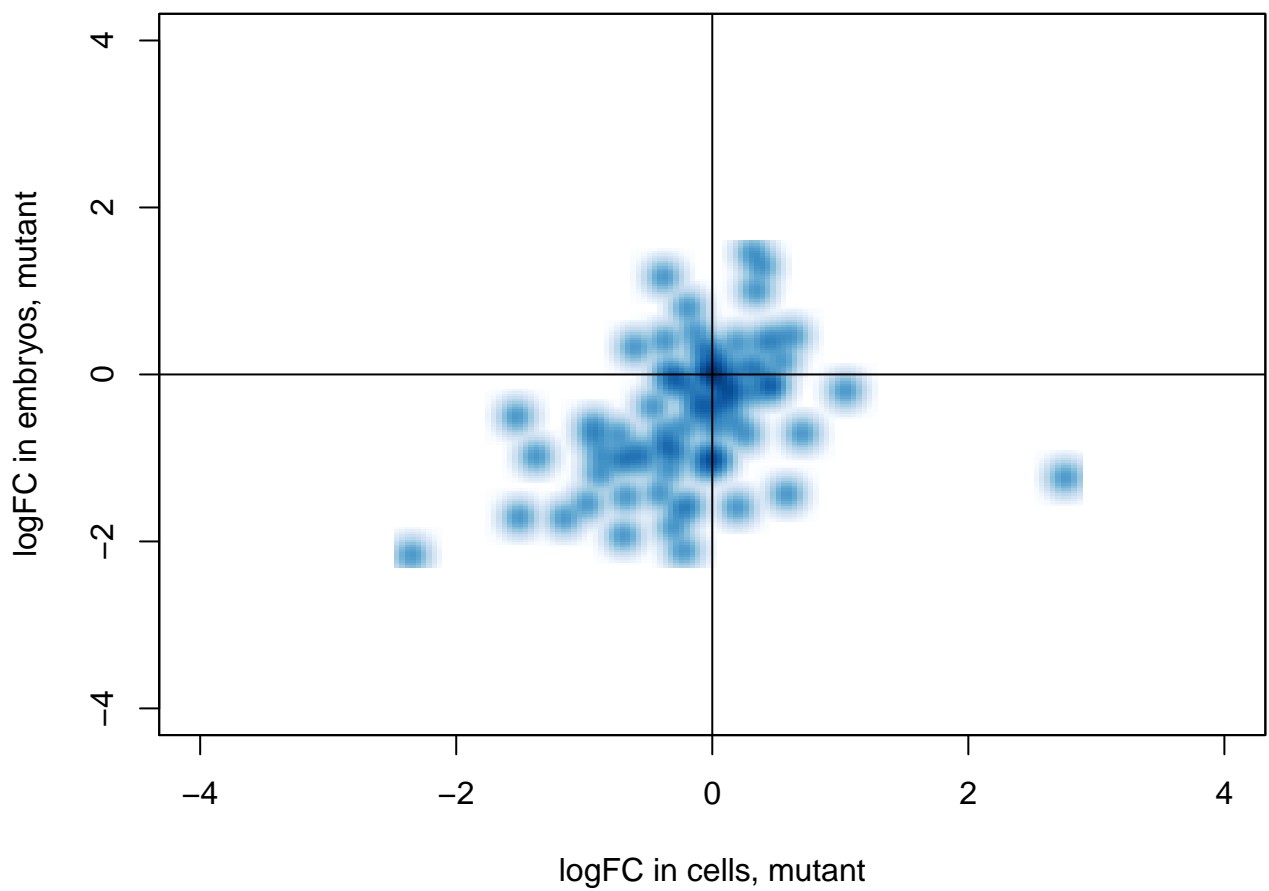

Supplement: File S1 — Archive (gzip2 compressed tar ball format) with 82 PDF files showing scatter plots for GO biological process terms with more than 50 annotated genes. Legend analogous to Figure 2. (BZ2) [file pone.0028985.s013.bz2 › scatterplots for 82 GO process terms with more than 50 genes/transport-results.pdf]

### genes in vesicle-mediated transport

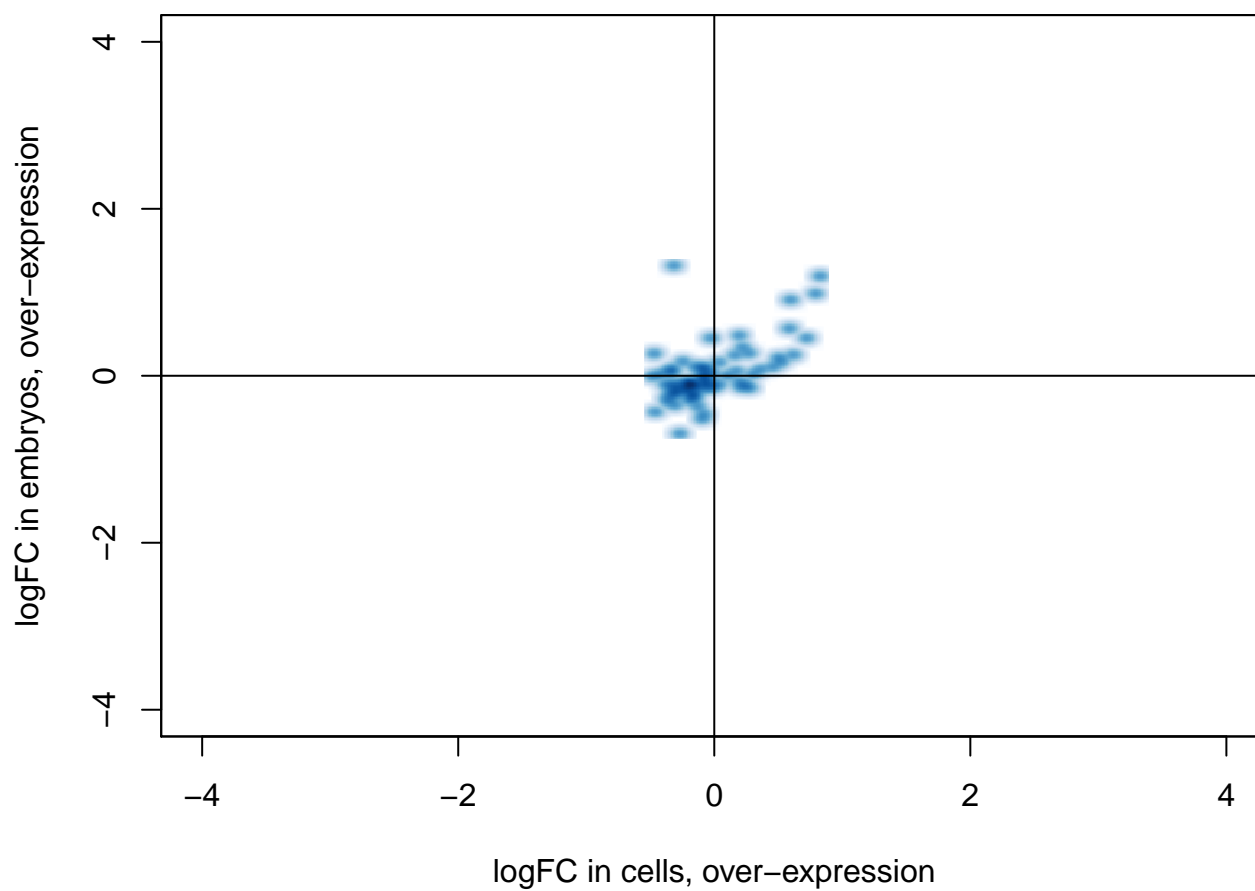

### genes in vesicle-mediated transport

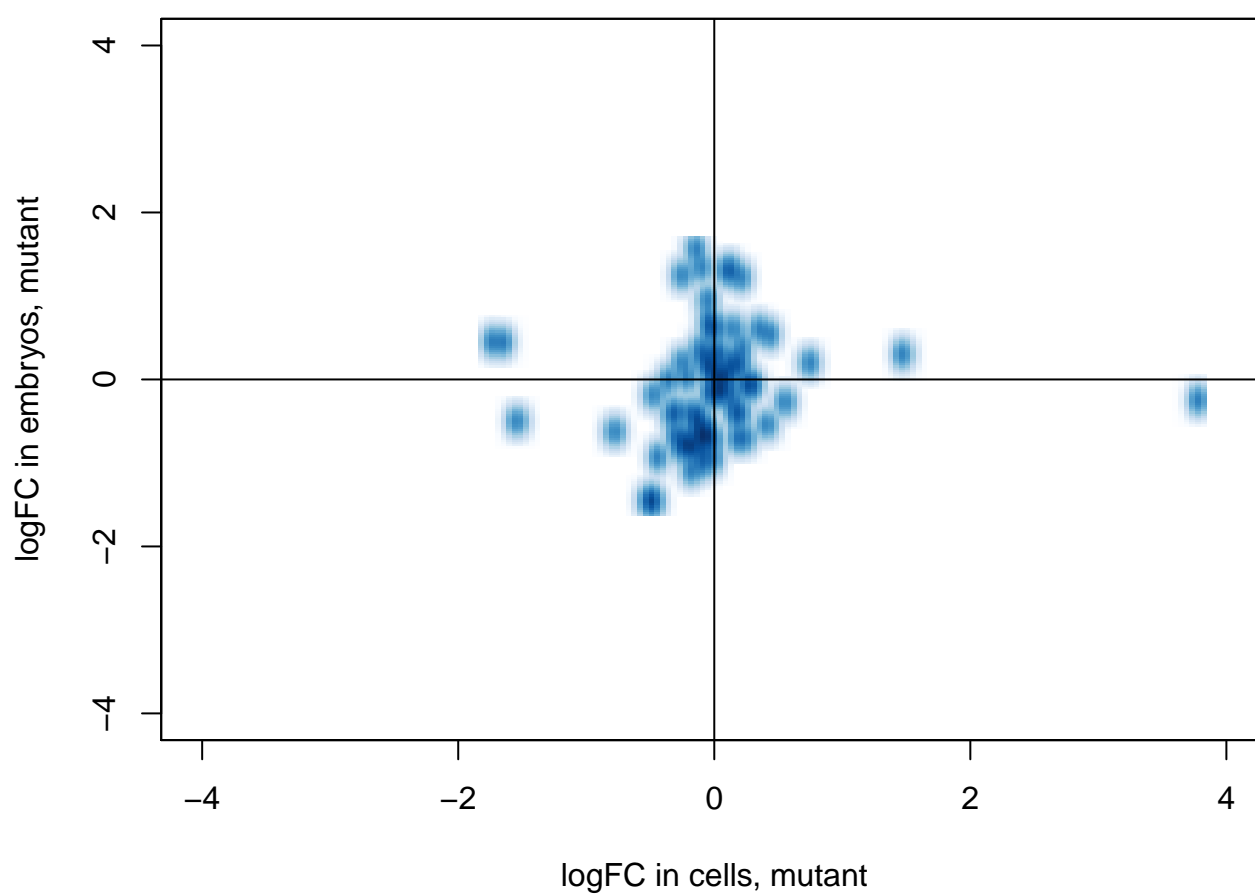

Supplement: File S1 — Archive (gzip2 compressed tar ball format) with 82 PDF files showing scatter plots for GO biological process terms with more than 50 annotated genes. Legend analogous to Figure 2. (BZ2) [file pone.0028985.s013.bz2 › scatterplots for 82 GO process terms with more than 50 genes/vesicle-mediated transport-results.pdf]

### genes in wing disc development

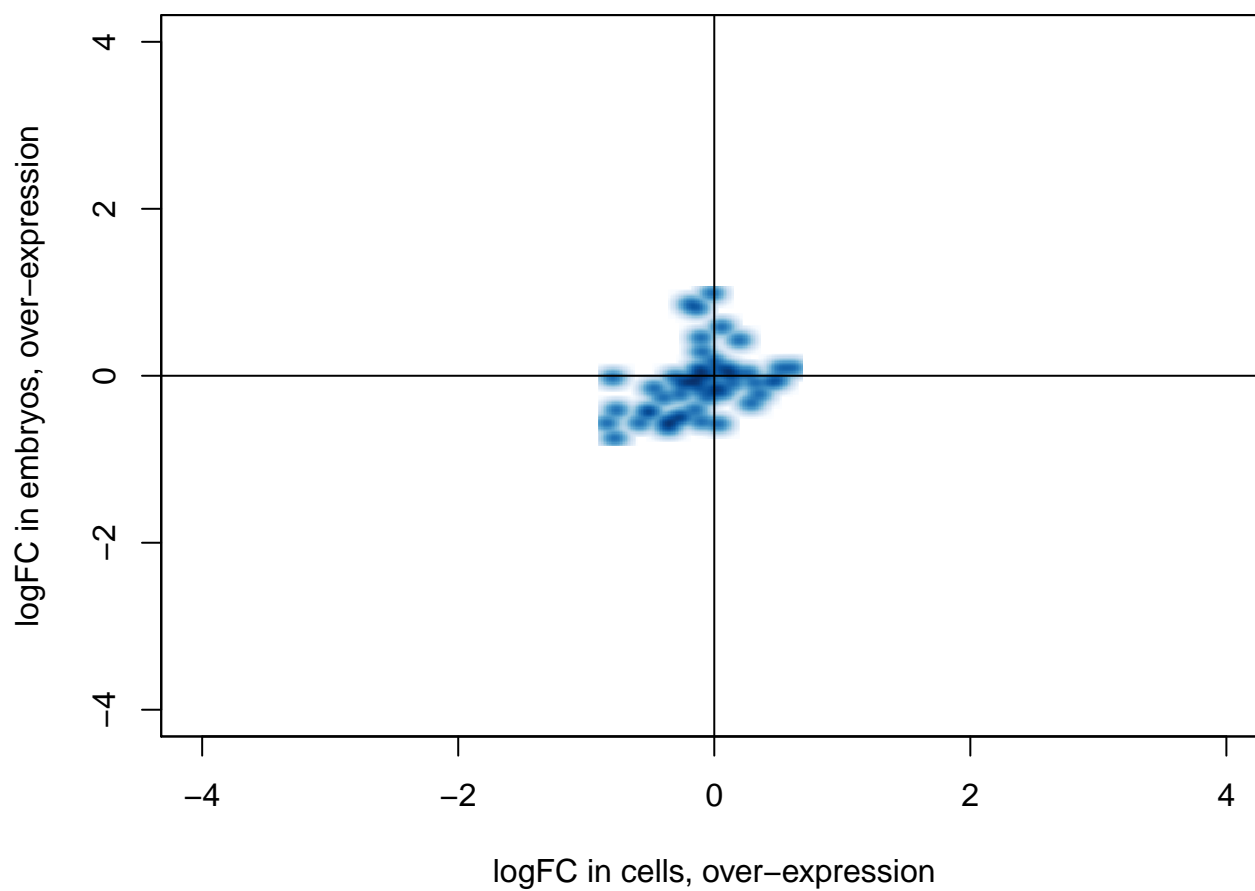

### genes in wing disc development

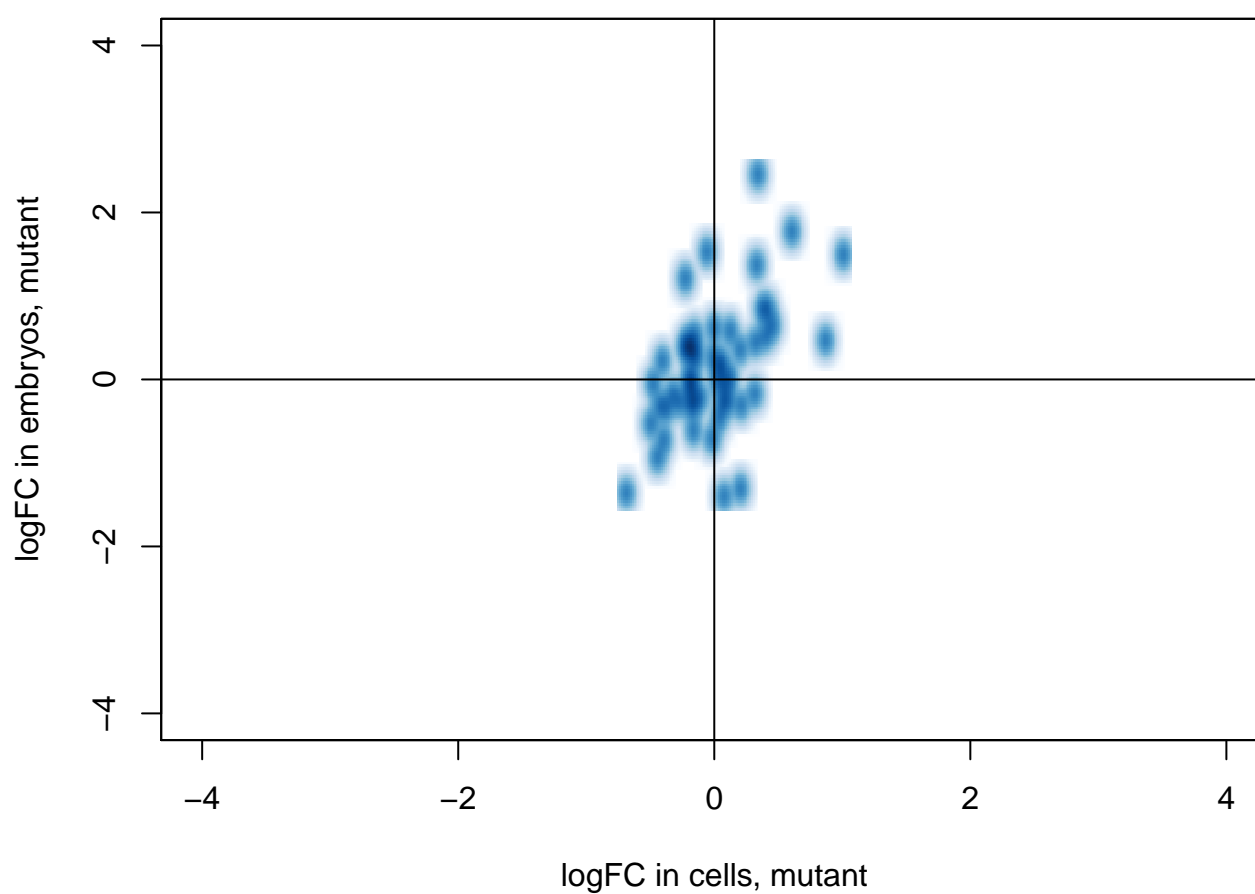

Supplement: File S1 — Archive (gzip2 compressed tar ball format) with 82 PDF files showing scatter plots for GO biological process terms with more than 50 annotated genes. Legend analogous to Figure 2. (BZ2) [file pone.0028985.s013.bz2 › scatterplots for 82 GO process terms with more than 50 genes/wing disc development-results.pdf]

### genes in Wnt receptor signaling pathway

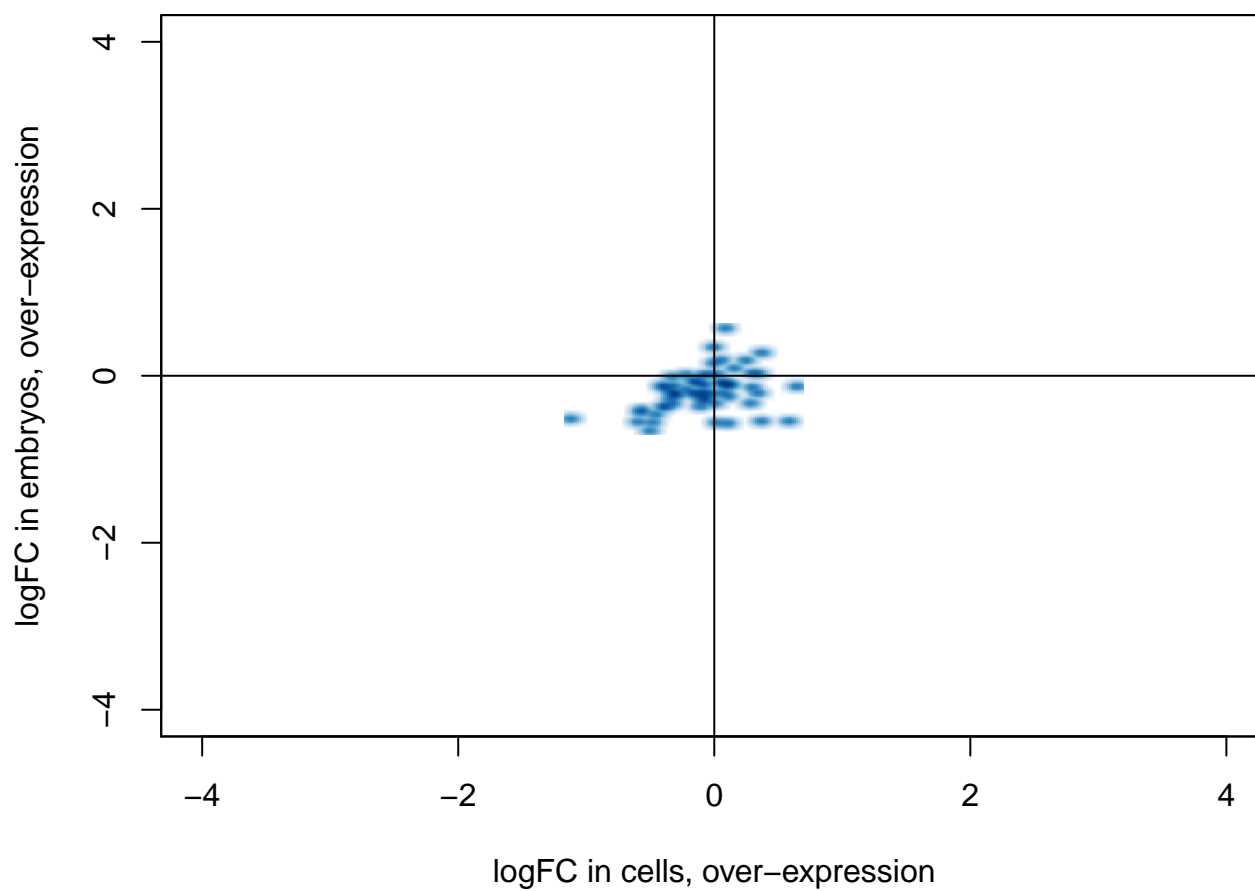

### genes in Wnt receptor signaling pathway

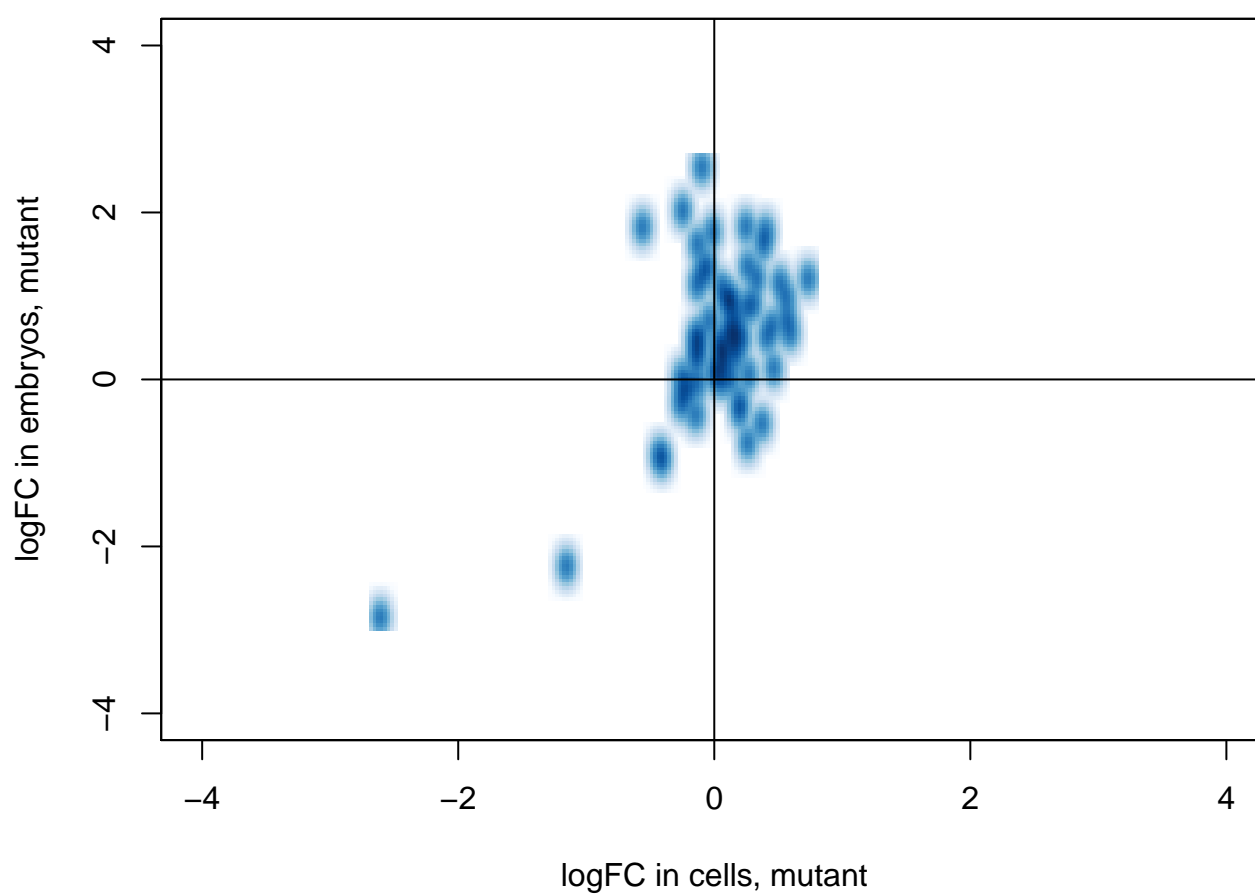

Supplement: File S1 — Archive (gzip2 compressed tar ball format) with 82 PDF files showing scatter plots for GO biological process terms with more than 50 annotated genes. Legend analogous to Figure 2. (BZ2) [file pone.0028985.s013.bz2 › scatterplots for 82 GO process terms with more than 50 genes/Wnt receptor signaling pathway-results.pdf]
