# Supplementary material for: Tramtrack Is Genetically Upstream of Genes Controlling Tracheal Tube Size in Drosophila
Source: PLoS One. 2011 Dec 22;6(12):e28985. doi: 10.1371/journal.pone.0028985 (PMC3245245; doi:10.1371/journal.pone.0028985)
Supplement: Table S5 — Real-time PCR primer sequences for target validation. (DOC) [file pone.0028985.s009.doc]

**Supplementary Table 5**

| **Real-time qPCR primer sequences** | | | | |
| --- | --- | --- | --- | --- |
| **Gene name** | **Gene symbol** | **FlyBase ID** | **Forward primer 5’-3’** | **Reverse primer 5’-3’** |
| *Chitinase 2* | *Cht2* | CG2054 | AACTATCCCCTTCTGCGGACC | GAATCTCGTTCTCGGAGTCATCC |
| *Cht5* | *Cht5* | CG9307 | ATCGGTCGCTATGGATTGGAG | AAACTTGCTGAAGCCACCCTG |
| *Chitin deacetylase-like4* | *Cda4* | CG32499 | TCACACATTGCCAACGGTAACTAC | TGGACAGCGGTTCAAATACGG |
| *Cuticular protein 78Cb* | *Cpr78Cb* | CG7663 | GCAAGTGTCCGTGAAGAAGGC | AGGGCAAGAAACATTTGGGC |
| *Imaginal disc growth factor 3* | *Idgf3* | CG4559 | GCTCCTGGAATCGGGACAAC | GCGTGGCTTATTTCTGGGAAG |
| *Cht9* | *Cht9* | CG10531 | ACTCGCTGTCCTCTGCCTTAGC | AACTTGCCATTGCCGCTGC |
| *CG7715* | *CG7715* | CG7715 | TACTATGTGTGCCCGAGCGTC | TCTGGAGGACAAAGGTGAAGACC |
| *CG8460* | *CG8460* | CG8460 | CGATTTGTTTTTGTGCGATTTATG | CAGTAGAAGCAGGAGAGCGACG |
| *Imaginal disc growth factor 1* | *Idgf1* | CG4472 | AGTTGTCGCAGAACGCATCC | CCATACGCCAAAATCTCCATTATC |
| *discs large 1* | *dlg1* | CG1725 | CATGCGCATCGAATCGGACAC | AGTCTGGGACCCGCTCCTCGA |
| *scribbled* | *scrib* | CG42614 | CAGTTGCTGTATCTGCCCTACTCG | ACACCTGTTCGCCCGTTTCC |
| *varicose* | *vari* | CG9326 | CCAGAAACAACAAAGTGAATGGC | CATCGTGGTCGGATAGGTCAGTC |
| *headcase* | *hdc* | CG15532 | TTCTTCAGGCGTCCTAATGGC | CCAGTTTGCGACAGTCTCCG |
| *scute* | *sc* | CG3827 | GCAATCAAAATCAACCCGCTG | TGGTCAGTGCCATACCCCTTG |
| *achaete* | *ac* | CG3796 | GCAATCTCCAACTGGCAGCAC | CGGTAGTCTTCAAAACTGGCTTCC |
| *Hairless* | *H* | CG5460 | CGGTTAGCGATGATAGCGAGTC | CATTCGTGATTCTGGGCAGC |
| *CG13167* | *CG13167* | CG13167 | CCTGCCCATCTTTCCATCCC | TTCCGCTTGAGGAGACCCAC |
| *branchless* | *bnl* | CG4608 | CGGTGTTCCCTACGGCTCGAAAG | GGCACCGTCTCCGTGATGGC |
