# Supplementary material for: Tramtrack Is Genetically Upstream of Genes Controlling Tracheal Tube Size in Drosophila
Source: PLoS One. 2011 Dec 22;6(12):e28985. doi: 10.1371/journal.pone.0028985 (PMC3245245; doi:10.1371/journal.pone.0028985)
Supplement: Table S7 — Analysis of stage-specific regulation by quantitative PCR. (DOC) [file pone.0028985.s011.doc]

**Supplementary Table 7: Analysis of stage-specific regulation by quantitative PCR**

The table shows the values obtained in the microarray (embryos at stages 11-16) and in the stage-controlled qPCR experiments (embryos at stages 11-13 and at stages 14-16). The analysis was performed on selected genes in whole embryo conditions in *ttk*^D2-50^ mutants versus control. Note the resolution of the stage-specific qPCR experiments.

| **Gene symbol** | **Microarray logFC**  **(stage 11-16)** | **p-value** | **qPCR**  **(stage 11-13)** | **p-value** | **qPCR**  **(stage 14-16)** | **p-value** |
| --- | --- | --- | --- | --- | --- | --- |
| *ac* | 1.21 | 3.12E-01 | -5.20 | <0,05 | 3.83 | <0,05 |
| *bnl* | -0.27 | 1.82 E^-01^ | -1.62 | <0,05 | -2.32 | <0,05 |
| *Cda4* | -2.03 | 1.59 E^-01^ | -1.19 | <0,05 | -1.30 | <0,05 |
