# Supplementary material for: Tramtrack Is Genetically Upstream of Genes Controlling Tracheal Tube Size in Drosophila
Source: PLoS One. 2011 Dec 22;6(12):e28985. doi: 10.1371/journal.pone.0028985 (PMC3245245; doi:10.1371/journal.pone.0028985)
Supplement: Text S1 — Quality control of microarrays. (DOC) [file pone.0028985.s012.doc]

**Text S1: Quality Control**

Mutant data

For the cell isolates it was expected that only a subset of the genes is expressed (of those which can be found in the whole embryo), and on the other hand genes specific to the purified tissues will appear to be expressed stronger, due to less dilution. Taking this into account, early normalization between groups was avoided, as this usually assumes that most genes are expressed at the same level. To judge overall microarray quality, metrics were preferred which averaged over large groups of genes. On the basis of these metrics we excluded two microarrays for the whole embryo material (arrays *BRJune09-474-2009-CON290508.CEL* and *BRJune09-475-TTK290509.CEL*) and one array from the cell specific material (*BRJune09-309-2009-TTK080509.CEL*) (data not shown). The remaining arrays were comparable within groups, with remarkably little difference between experiments in the histograms and the MA plots, except for lower signal values in the group of cell isolates, where there was considerably more noise than with whole embryos (see Supplementary Figure 1). This might be due to lower amounts of input material and thus stronger amplification. Also the cell isolates produced a somewhat weaker average signal than the whole embryos.

Over-expression data

Data from the IBMB Core Facility comprised 3 microarrays for each embryonic control and sample, and 2 microarrays for each sorted cell population (control and sample).

No arrays were discarded from the over-expression experiments. The arrays showed comparable similarity within and between groups to the arrays in the mutant experiments.
