# Supplementary material for: Tramtrack Is Genetically Upstream of Genes Controlling Tracheal Tube Size in Drosophila
Source: PLoS One. 2011 Dec 22;6(12):e28985. doi: 10.1371/journal.pone.0028985 (PMC3245245; doi:10.1371/journal.pone.0028985)
Supplement: File S1 — Archive (gzip2 compressed tar ball format) with 82 PDF files showing scatter plots for GO biological process terms with more than 50 annotated genes. Legend analogous to Figure 2. (BZ2) [file pone.0028985.s013.bz2 › scatterplots for 82 GO process terms with more than 50 genes/regulation of alternative nuclear mRNA splicing, via spliceosome-results.pdf]

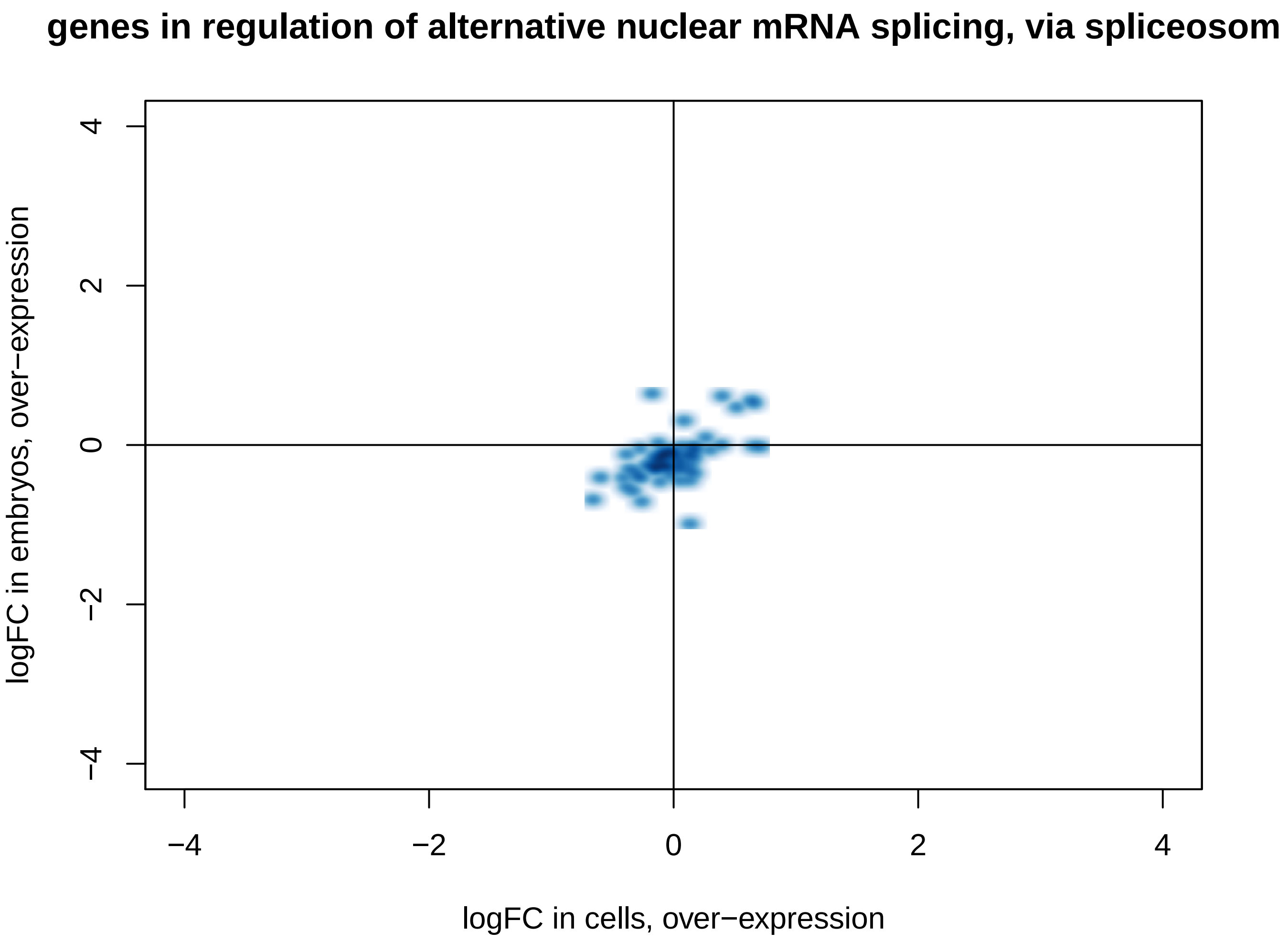

genes in regulation of alternative nuclear mRNA splicing, via spliceosom
logFC in embryos, over−expression
logFC in cells, over−expression
−4
−2
0
2
4


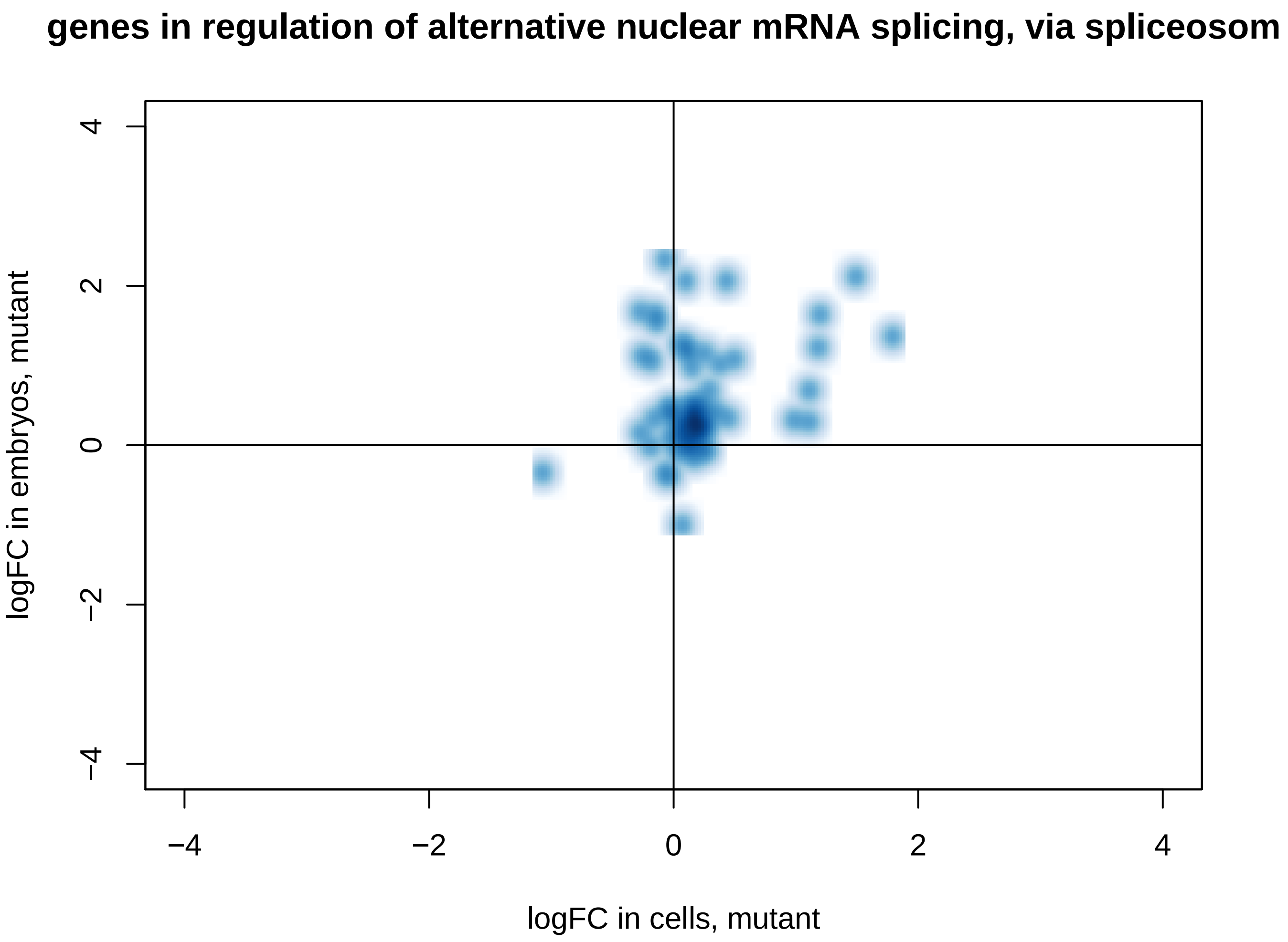

genes in regulation of alternative nuclear mRNA splicing, via spliceosom
logFC in embryos, mutant
logFC in cells, mutant
−4
−2
0
2
4
